# Origin of worldwide cultivated barley revealed by *NAM-1* gene and grain protein content

**DOI:** 10.3389/fpls.2015.00803

**Published:** 2015-09-30

**Authors:** Yonggang Wang, Xifeng Ren, Dongfa Sun, Genlou Sun

**Affiliations:** ^1^College of Plant Science and Technology, Huazhong Agricultural UniversityWuhan, China; ^2^Department of Biology, Saint Mary’s University, HalifaxNS, Canada

**Keywords:** origin, spread, barley, *NAM-1* gene, grain protein content

## Abstract

The origin, evolution, and distribution of cultivated barley provides powerful insights into the historic origin and early spread of agrarian culture. Here, population-based genetic diversity and phylogenetic analyses were performed to determine the evolution and origin of barley and how domestication and subsequent introgression have affected the genetic diversity and changes in cultivated barley on a worldwide scale. A set of worldwide cultivated and wild barleys from Asia and Tibet of China were analyzed using the sequences for *NAM-1* gene and gene-associated traits-grain protein content (GPC). Our results showed Tibetan wild barley distinctly diverged from Near Eastern barley, and confirmed that Tibet is one of the origin and domestication centers for cultivated barley, and in turn supported a polyphyletic origin of domesticated barley. Comparison of haplotype composition among geographic regions revealed gene flow between Eastern and Western barley populations, suggesting that the Silk Road might have played a crucial role in the spread of genes. The GPC in the 118 cultivated and 93 wild barley accessions ranged from 6.73 to 12.35% with a mean of 9.43%. Overall, wild barley had higher averaged GPC (10.44%) than cultivated barley. Two unique haplotypes (Hap2 and Hap7) caused by a base mutations (at position 544) in the coding region of the *NAM-1* gene might have a significant impact on the GPC. Single nucleotide polymorphisms and haplotypes of *NAM-1* associated with GPC in barley could provide a useful method for screening GPC in barley germplasm. The Tibetan wild accessions with lower GPC could be useful for malt barley breeding.

## Introduction

Wild barley, *Hordeum spontaneum* C. Koch, is the progenitor of cultivated barley, *Hordeum vulgare* L. As one of the earliest domesticated crops, barley has been one of most important staple crops in old world Neolithic agriculture upon which early agriculture was built (Harlan and Zohary, 1966; Zohary and Hopf, 2000). The domestication of barley is fundamental to understanding the origin and early diffusion of agrarian culture (Morrell and Clegg, 2007).

The geographic range of *H. spontaneum* was clearly defined, and the Fertile Crescent has been suggested as the only location where barley was domesticated by a large number of researchers (Harlan and Zohary, 1966; Nevo et al., 1984, 1986; Pakniyat et al., 1997; Badr et al., 2000; Nevo, 2006; Zohary et al., 2012). However, the centre of origin of barley has not been fully resolved. *H. spontaneum*, the wild ancestor of cultivated barley, has been discovered in several geographically distinct locations other than the Fertile Crescent, such as Morocco, Algeria, Libya, Egypt, Crete, Ethiopia, and Tibet, leading to the proposal of a multicentric origin for this crop (Åberg, 1938; Bekele, 1983; Molina-Cano et al., 1987; Von Bothmer et al., 1995; Molina-Cano et al., 2002; Fuller et al., 2011; Von Bothmer and Komatsuda, 2011), and was supported by additional studies (Takahashi and Hayashi, 1964; Azhaguvel and Komatsuda, 2007; Fuller et al., 2011). For instance, archeological evidence suggests a diffuse “center” of origin for barley (Fuller et al., 2011), and non-brittle rachis in oriental and occidental lines is controlled by two distinct genetic loci, indicating independent origins of oriental and occidental barleys (Takahashi and Hayashi, 1964; Azhaguvel and Komatsuda, 2007). In addition, numerous studies have reported clear genetic differentiation among barley populations from Eastern and Southern Asia and those from Western Asia, Europe, and North Africa (Azhaguvel and Komatsuda, 2007; Saisho and Purugganan, 2007; Fu and Peterson, 2011; Morrell et al., 2013). Recent molecular evidence indicated that an additional center of wild barley domestication event may occur in Central Asia at the eastern edge of the Iranian Plateau. It is assumed that this area constitutes the center of origin for cultivated barley from South and East Asia (Morrell and Clegg, 2007; Jones et al., 2008). Most noticeably, the Qinghai–Tibetan Plateau has been proposed as one of the centers of origin of cultivated barley. Recent Diversity array technology (DArT) data and transcriptome profiling suggested that the Tibetan Plateau and its vicinity was one of the centers of barley domestication, and further confirmed the multiple origins of cultivated barley (Dai et al., 2012, 2014).

The resequencing of candidate gene loci within diverse populations has implication for understanding the origin of barley domestication (Morrell and Clegg, 2007; Jones et al., 2008). Domestication has genetically not only transformed the brittle rachis, tenacious glume, and non-free thresh-ability, but also modified yield and yield components in many crops (Peng et al., 2011; Yan et al., 2015). Grain protein content (GPC) is a very important quality determinant in many cereals. In barley, GPC is closely related to feeding quality as well as malting and brewing processes. High protein concentration is attributed to feeding quality, while low protein content is favorable for barley malt and beer production (See et al., 2002). However, it is difficult to improve simultaneously grain yield (GY) and grain protein concentration (Bogard et al., 2010). Previous studies have demonstrated a strong genetic negative correlation between GPC and yield in various cereals (Oury and Godin, 2007; Bogard et al., 2011; Blanco et al., 2012; Martre et al., 2015). The content of grain protein (GPC) is determined by numerous factors including environmental effects, the elements determined yield, and complex genetic system (Simmonds, 1995). Whole-plant senescence processes overlap with grain filling, and the synchronization of these two processes is important for affecting yield and quality characteristics such as grain protein content (GPC; Distelfeld et al., 2014).

The NAC transcription factor family plays a central role in regulating organ and organism senescence (Uauy et al., 2006; Balazadeh et al., 2010; Zhu et al., 2012; Jensen and Skriver, 2014). It has been shown that a NAC transcription factor (*TtNAM-B1*) was related to the GPC of wheat (Uauy et al., 2006; Peng et al., 2013). In barley, the relationships between plant senescence, NAC gene expression and GPC were also studied (Cai et al., 2013). Two orthologs genes of a NAC transcription factor, *HvNAM-1* and *HvNAM-2* (GenBank accession number DQ869678 and DQ869679) have been mapped on chromosomes 6H and 2H, respectively (Uauy et al., 2006; Distelfeld et al., 2008). Allelic variation of the *NAM-1* gene seems to be related to protein content (Distelfeld et al., 2008; Jukanti and Fischer, 2008; Jamar et al., 2010). Recently, a genome-wide association study (GWAS) and a multi-platform candidate gene-based association analysis for cultivated and wild barley found that the two *HvNAM* genes might play a role in controlling GPC in barley (Cai et al., 2013).

The origin and domestication of cultivated barley have been widely discussed; however, the debate on these subjects still remains. To provide further evidence to determine the evolution and origin of barley and how barley domestication and subsequent introgression have affected the genetic diversity of cultivated barley, we characterized nucleotide diversity of the *NAM-1* gene and gene-associated traits-GPC in wild barley from Southwest Asia, Central Asia, Tibet of China, and cultivated barley from different parts of the world. Our primary goals were (i) to examine genetic differentiation between cultivated barley and wild-barley populations; (ii) to elucidate the origin and spread of worldwide cultivated barley; and (iii) to investigate the GPC in barley populations and its relationship to *NAM-1* gene.

## Materials and Methods

### Plant Materials

Total of 218 barley accessions were used in this study, including 94 accessions of wild barley (*H. spontaneum*) and 124 lines of cultivated barley (*H. vulgare*). The wild barley populations included: 53 wild barley accessions from the Southwest Asia (Israel, Jordan, Ethiopia, Lebanon, Azerbaijan, Syria, Turkey, and Iraq); 21 wild barley from Central Asia (Iran, Afghanistan, Tajikistan, and Pakistan), and 20 wild barley from Tibet of China. One hundred and twenty-four cultivated barley (*H. vulgare*) accessions were from 18 countries (61 form Eastern Asia, 8 from South America, 18 form North America, 10 from Mediterranean coast, 5 form Australia, and 22 from Europe). Those materials were provided by USDA (United States Department of Agriculture) and the Huazhong Agricultural University barley germplasm collection. The accessions names and their geographical origin were given in the Supplementary Table S1.

### DNA Extraction, *NAM-1* Gene Amplification, and Sequencing

The seeds were planted in pots with nutrient soil, and grown in a growth chamber with 14 h of light at 22°C and 10 h of darkness at 18°C prior to DNA extraction. Young leaves were collected from 5 to 10 plants of each accession and ground to a fine powder with liquid nitrogen and stored at –80°C until DNA extraction. DNA was extracted according to the method of Stein et al. (2001).

The *NAM-1* gene sequence was amplified using polymerase chain reaction (PCR). The forward and reverse primer sequences are Nam-1HF: 5′-TATCAAGCGCCGTAATTTCC-3′ and Nam-1HR: 5′-ATACTGCCGACGTTTCTGCT-3′, respectively (Ren et al., 2013). Amplification of DNA was carried out in 40 μl reaction mixture containing 60 ng template DNA, 0.2 μM of each primer, 1.5 mM MgCl_2_, 0.2 mM of each deoxynucleoside triphosphate (dNTP), and 1.5 unit of high-fidelity polymerase Ex*Taq* (TaKaRa, Dalian, China). PCR amplification was performed with an initial denaturing of 4 min at 95°C, followed by 40 cycles of 1 min at 95°C, 1 min at 52°C, 2 min at 72°C, and ending with an 8 min extension at 72°C.

Amplified products were electrophoresed in 1.0% agarose gel, and purified using the QIAquick^TM^ PCR purification kit (QIAGEN Inc) according to the manufacturer’s protocol. DNA was commercially sequenced at the Beijing TsingKe BioTech Co., Ltd (Beijing, China). The amplification and sequencing were repeated three times to exclude sequencing errors introduced by *Taq*DNA polymerase during PCR amplification. In addition, both forward and reverse strands were sequenced independently, and further checked for data quality using Chromas 2.32 (Technelysium Pty. Ltd.). Sequences of *NAM-1* genes in 214 barley accessions were shown in Supplementary Data Sheet S1.

### Grain Protein Content (GPC) Measurement

The GPC in 211 barley accessions was determined. All barley accessions were planted at the experimental field of Ezhou (Hubei, China, 114.41°E. 30.06°N) in the early winter of 2013, and were cultivated in the same conditions with identical agronomic managements until maturity and harvested. Mature grains were fully ground and passed through a 0.5-mm screen after dehydration until constant mass is reached. The GPC was measured using the method of Kjeldahl (1883), three measurements were done for each sample. Protein content is calculated with a factor of 6.25 for N content (Mariotti et al., 2008).

### Data Analysis

Multiple sequences were aligned using ClustalX (Thompson et al., 1997). Genetic diversity was estimated by Tajima’s (1989) π and Watterson’s (1975) statistics using DnaSP version 5.0 (Librado and Rozas, 2009), and the tests of neutral evolution were performed as described by Tajima (1989) and Fu and Li (1993). Phylogenetic analysis was performed with the computer program MEGA 6 (Tamura et al., 2013). The phylogenetic tree of the 214 accessions was constructed using neighbor-joining (Saitou and Nei, 1987) methods with Tajima–Nei model. The confidence of each clade was evaluated by the bootstrap values with 1,000 replicates. Statistical analyses were performed by using SAS 9.0 software (SAS Institute, Cary, North Carolina, USA). Significance between groups was evaluated by one-way analysis of variance (ANOVA) followed by a Newman–Keuls *post hoc* test, a *P* value of <0.05 was considered statistically significant.

## Results

### Haplotype Frequency Analysis in Barley Populations

Eight Single nucleotide polymorphisms (SNPs) and 10 distinguishable haplotypes were identified in 214 barley accessions (**Table 1**). Haplotypes in three wild barley populations and six cultivated barley populations were compared and are shown in **Table 2**. Ten haplotypes were detected across 94 wild barley accessions. Of these, seven haplotypes were detected in the Southwest Asian wild population, five in the Tibetan wild barley population, and two in the Central Asian wild barley population. Moreover, seven haplotypes were population-specific, four specific to the Southwest Asian wild population, and three specific to the Tibetan wild barley population. In contrast, only three haplotypes were observed in cultivars, with all three present in the North American and European cultivated barley populations, and two of them in the other four cultivar populations. No population-specific haplotype was found in the cultivated barley populations. The three haplotypes identified in cultivars were also identified in both Southwest Asian and Tibetan wild barley populations, suggesting that two major wild-barley populations, the Near East Fertile Crescent and Tibetan Plateau populations, might have contributed to the origin of cultivated barley.

**Table 1:** Distribution of polymorphic SNPs across ten *NAM-1* haplotypes.

Haplotypes	SNP position								Total number of accessions
	375	473	507	544	616	823	1190	1253	
Hap1	T	G	C	C	C	A	G	T	17
Hap2	T	**A**	C	C	C	A	G	T	96
Hap3	T	**A**	C	C	C	A	G	**C**	4
Hap4	T	**A**	**T**	C	C	A	G	T	4
Hap5	T	**A**	C	C	**A**	A	G	T	3
Hap6	T	**A**	C	C	C	**C**	G	T	3
Hap7	T	**A**	C	**G**	C	A	G	T	80
Hap8	**A**	**A**	C	**G**	C	A	G	T	2
Hap9	T	**A**	C	C	C	A	**A**	T	4
Hap10	**A**	**A**	C	C	C	A	**A**	T	1

*SNPs relative to the haplotype Hap1 are indicated in bold. The number of SNP positions is relative to the sequence on GenBank accession number DQ869678.*

**Table 2:** Haplotype frequencies of *NAM-1* gene in three wild barley populations and six landrace barley populations.

NAM	Wb-T (20)	Wb-C (21)	Wb-S (53)	Lb-EA (61)	Lb-NA (16)	Lb-SA (8)	Lb-MA (10)	Lb-EU (20)	Lb-AU (5)	Overall (214)
Hap1	0	0	0.264 (14)	0	0.063 (1)	0	0	0.10 (2)	0	0.079 (17)
Hap2	0.50 (10)	0.905 (19)	0.453 (24)	0.525 (32)	0.063 (1)	0.25 (2)	0.20 (2)	0.25 (5)	0.20 (1)	0.449 (96)
Hap3	0	0	0.075 (4)	0	0	0	0	0	0	0.019 (4)
Hap4	0	0.095 (2)	0.038 (2)	0	0	0	0	0	0	0.019 (4)
Hap5	0	0	0.057 (3)	0	0	0	0	0	0	0.014 (3)
Hap6	0	0	0.057 (3)	0	0	0	0	0	0	0.014 (3)
Hap7	0.30 (6)	0	0	0.475 (29)	0.875 (14)	0.75 (6)	0.80 (8)	0.65 (13)	0.80 (4)	0.374 (80)
Hap8	0.10 (2)	0	0	0	0	0	0	0	0	0.009 (2)
Hap9	0.05 (1)	0	0.057 (3)	0	0	0	0	0	0	0.019 (4)
Hap10	0.05 (1)	0	0	0	0	0	0	0	0	0.005 (1)

*The three wild populations are Wb-T (Wild barley of Tibet), Wb-C (Wild barley of Central Asia), and Wb-S (Wild barley of Southwest Asia), respectively; The six landrace populations as follows: Lb-EA (Landrace barley of East Asia), Lb-NA (Landrace barley of North America), Lb-SA (Landrace barley of South America), Lb-MA (Landrace barley of the Mediterranean Coast Areas), Lb-EU (Landrace barley of Europe), and Lb-AU (Landrace barley of Australia).*

**FIGURE 1:**
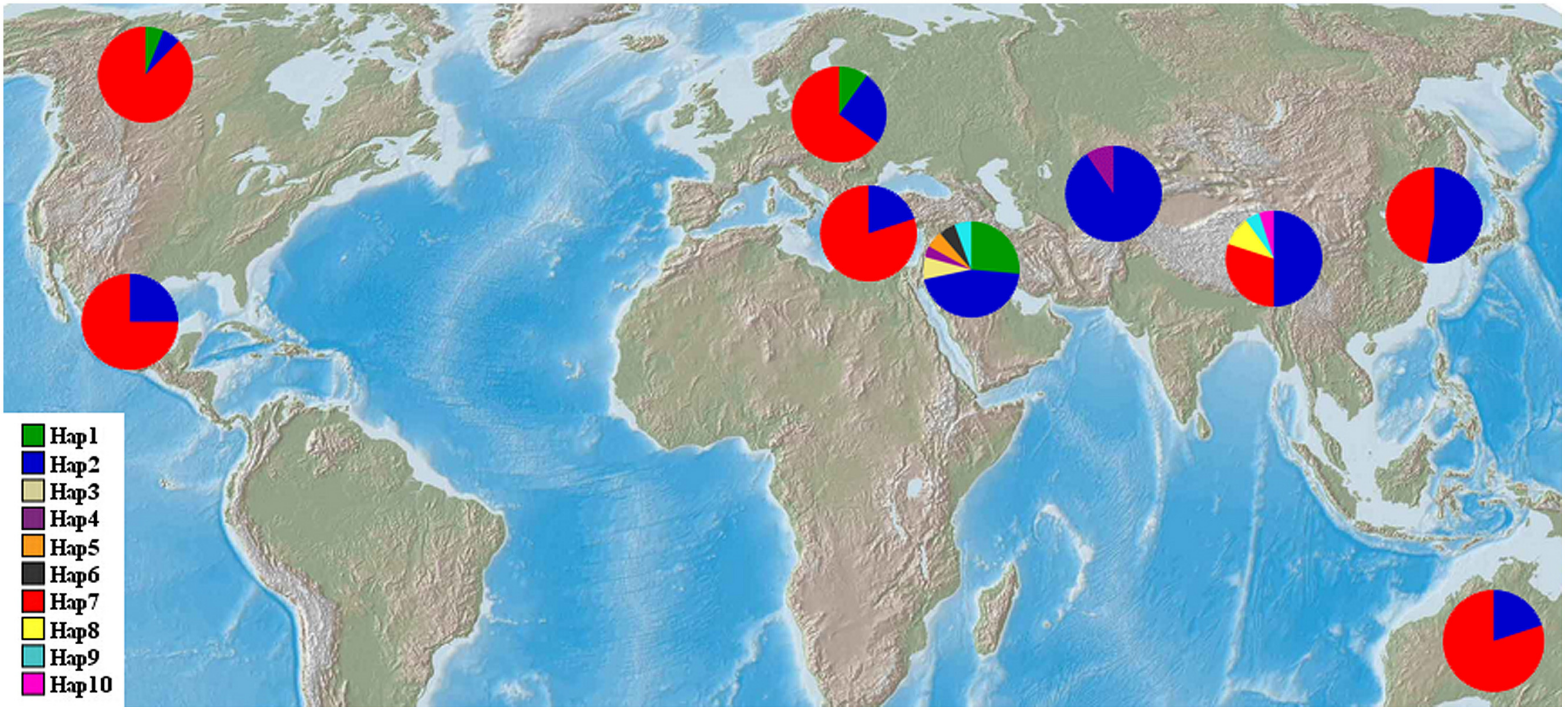
**Geographic distribution of wild barley populations and landrace populations.** *NAM-1* haplotype frequencies among nine geographic regions were displayed in pie diagrams and the exact proportions of each are given in percent by the corresponding color code.

The haplotype frequencies present in all accessions ranged from 0.005 to 0.449. Among all the haplotypes, three haplotypes were detected in 193 barley accessions. The haplotype Hap2 appeared in 96 accessions (44.9%), Hap7 was observed in 80 accessions (37.4%), and Hap1 was present in 17 accessions (7.9%). Of the seven haplotypes present in <2% of the accessions sampled, five were unique to the specific wild populations, i.e., Hap3 and Hap8 was unique to the Southwest Asian wild barley population and the Tibetan wild barley populations, respectively. The frequencies of *NAM-1* haplotypes differed markedly among different geographic populations. The haplotype Hap1 was the most frequent one in the Southwest Asian wild barley population (0.264), but rare in the North American and European landraces (0.063 and 0.10, respectively), and absent in the remaining six populations. The rare haplotypes either confined or occurred in specific geographic regions, i.e., the Hap2 was detected in a few landrace accessions from North America (0.063), and the Hap3 (0.075), Hap5 (0.057), and Hap6 (0.057) were population-specific to wild barley in Southwest Asia; Hap8 and Hap10 were unique to the wild population in Tibet (**Table 2**; **Figure 1**).

### Genetic Diversity Analysis and Neutrality Test

Genetic variation analyses revealed that wild barley, except Central Asian populations, showed higher haplotype diversity than landraces. The highest diversity was detected in the Southwest Asian wild barley population (0.722), followed by the Tibetan wild barley population (0.679). The pre-site nucleotide diversity ranged from 0.00023 ± 0.00023 (East Asian landrace population) to 0.00141 ± 0.00067 (Southwest Asian wild population). Higher values were also discovered in the Southwest Asian and Tibetan wild barley populations. These estimates corresponded well to the number of *NAM-1* haplotypes (7 and 5, respectively), as well as the diversity based on the number of segregating sites (0.00100 and 0.00103, respectively; **Table 3**).

**Table 3:** Estimates of nucleotide diversity per base pair and test statistics for *NAM-1* gene.

Population	No. of accessions	No. of haplotypes (H)	Haplotype diversity (Hd)	Theta (per site) from S (𝜃)	Nucleotide diversity (π)	Tajima’s *D* test	Fu and Li’s *D* test	Fu and Li’s *F* test
Wb-T	20	5	0.679	0.00090 ± 0.00058	0.00103	0.37128	1.00649	0.95750
Wb-C	21	2	0.181	0.00030 ± 0.00030	0.00019	-0.61772	0.64197	0.35117
Wb-S	53	7	0.722	0.00141 ± 0.00067	0.00100	-0.72915	1.17017	0.66550
Lb-EA	61	2	0.507	0.00023 ± 0.00023	0.00054	1.75537	0.52682	1.02603
Lb-NA	16	3	0.242	0.00064 ± 0.00048	0.00038	-1.03789	-0.50381	-0.73427
Lb-SA	8	2	0.429	0.00041 ± 0.00041	0.00046	0.33350	0.88779	0.82528
Lb-MA	10	2	0.356	0.00038 ± 0.00038	0.00038	0.01499	0.80424	0.68403
Lb-EU	20	3	0.532	0.00060 ± 0.00045	0.00071	0.43538	0.86615	0.86048
Lb-AU	5	2	0.400	0.00051 ± 0.00051	0.00043	-0.81650	-0.81650	-0.77152
All	214	10	0.654	0.00144 ± 0.00058	0.00088	-0.84723	1.20381	0.56801

*The three wild populations are Wb-T (Wild barley of Tibet), Wb-C (Wild barley of Central Asia), and Wb-S (Wild barley of Southwest Asia), respectively; The six landrace populations as follows: Lb-EA (Landrace barley of East Asia), Lb-NA (Landrace barley of North America), Lb-SA (Landrace barley of South America), Lb-MA (Landrace barley of the Mediterranean Coast Areas), Lb-EU (Landrace barley of Europe), and Lb-AU (Landrace barley of Australia).*

Tajima (1989) and Fu and Li’s (1993) neutrality tests were performed to determine whether the observed genetic diversity follows an equilibrium neutral model. Both positive values were obtained from the East Asian, South American, Mediterranean Coast and European landrace populations as well as Tibetan wild barley populations. In contrast, negative values for both tests were obtained from the North American and Australian landrace populations. Moreover, Tajima’s *D* values were negative for the Central Asian and Southwest Asian wild barley populations (–0.61772 and –0.72915, respectively), while the Fu and Li test values were positive for these two populations. None of the values were statistically significant (*P* = 0.05; **Table 3**).

### Phylogenetic Analysis of *NAM-1* Gene

**FIGURE 2:**
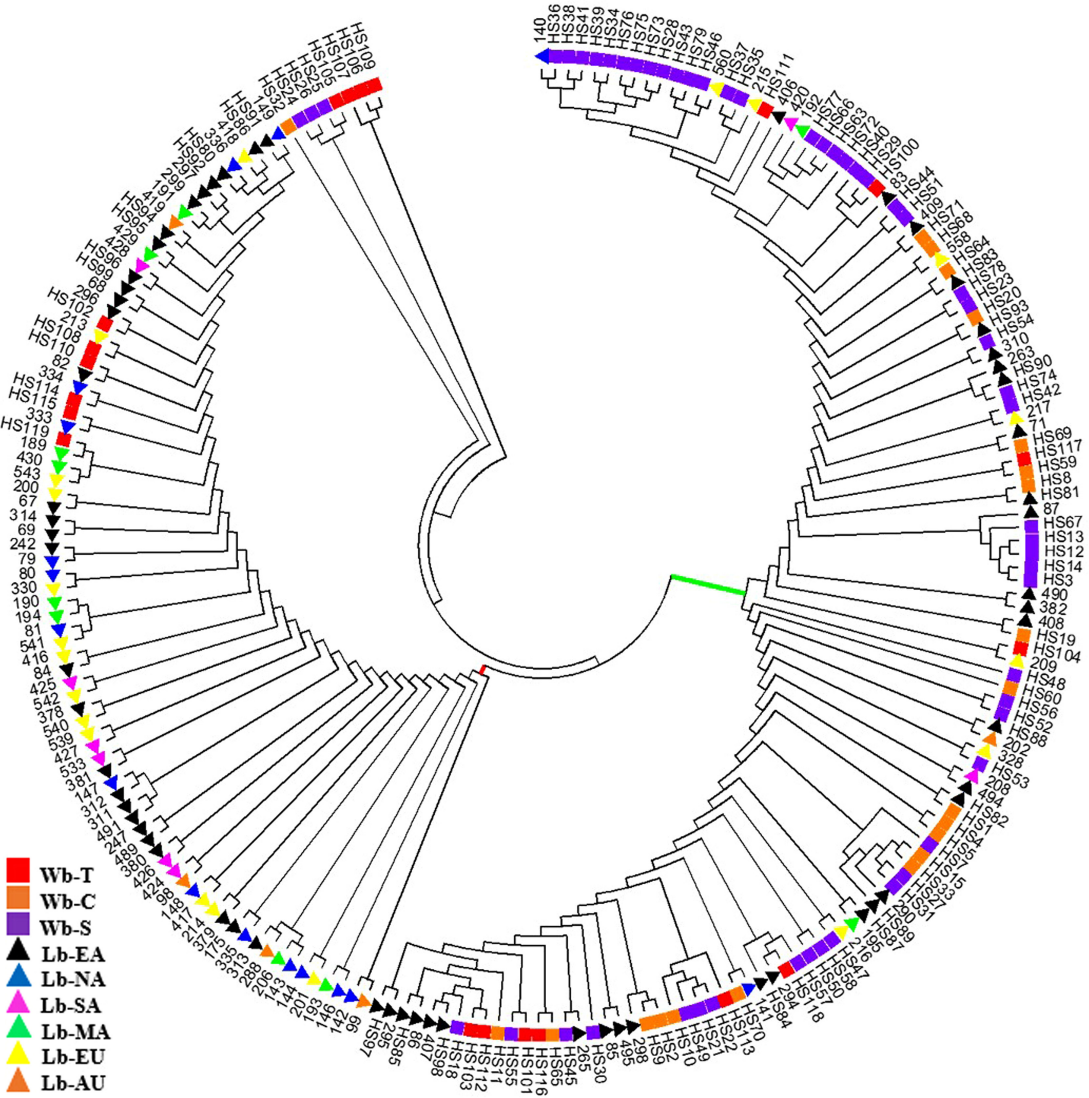
**Phylogenetic tree of 214 barley accessions based on the *NAM-1* gene.** Two major clusters, one comprised of a majority of wild barley accessions (represented in green bar) and another comprised of a majority of cultivated barley accessions (represented in red bar) are separated. The square stands for wild barley accessions: Tibet (Wb-T, red), Southwest Asia (Wb-S, purple), and Central Asia (Wb-C, orange), respectively; the triangle indicates landrace barleys: East Asia (Lb-EA, black), North America (Lb-NA, blue), South America (Lb-SA, pink), Mediterranean Coast Areas (Lb-MA, green), Europe (Lb-EU, yellow), and Australia (Lb-AU, orange).

A Phylogenetic tree was constructed to depict genetic relationships among the 214 samples based on the *NAM-1* gene (**Figure 2**). The neighbor-joining analysis placed these samples into two major clusters, one comprised of the majority of wild barley accessions (red bar in **Figure 2**) and another comprised of the majority of cultivated barley accessions (green bar in **Figure 2**). However, some Tibetan wild barleys were distinct from the Near Eastern and Central Asian wild barleys, and appeared in the cultivars-dominated cluster. The third cluster comprised of four accession of wild barley from Tibet, three accessions of wild barley from Southwest Asia, and one accession of wild barley from Central Asia.

### The Variation of Protein Content

**FIGURE 3:**
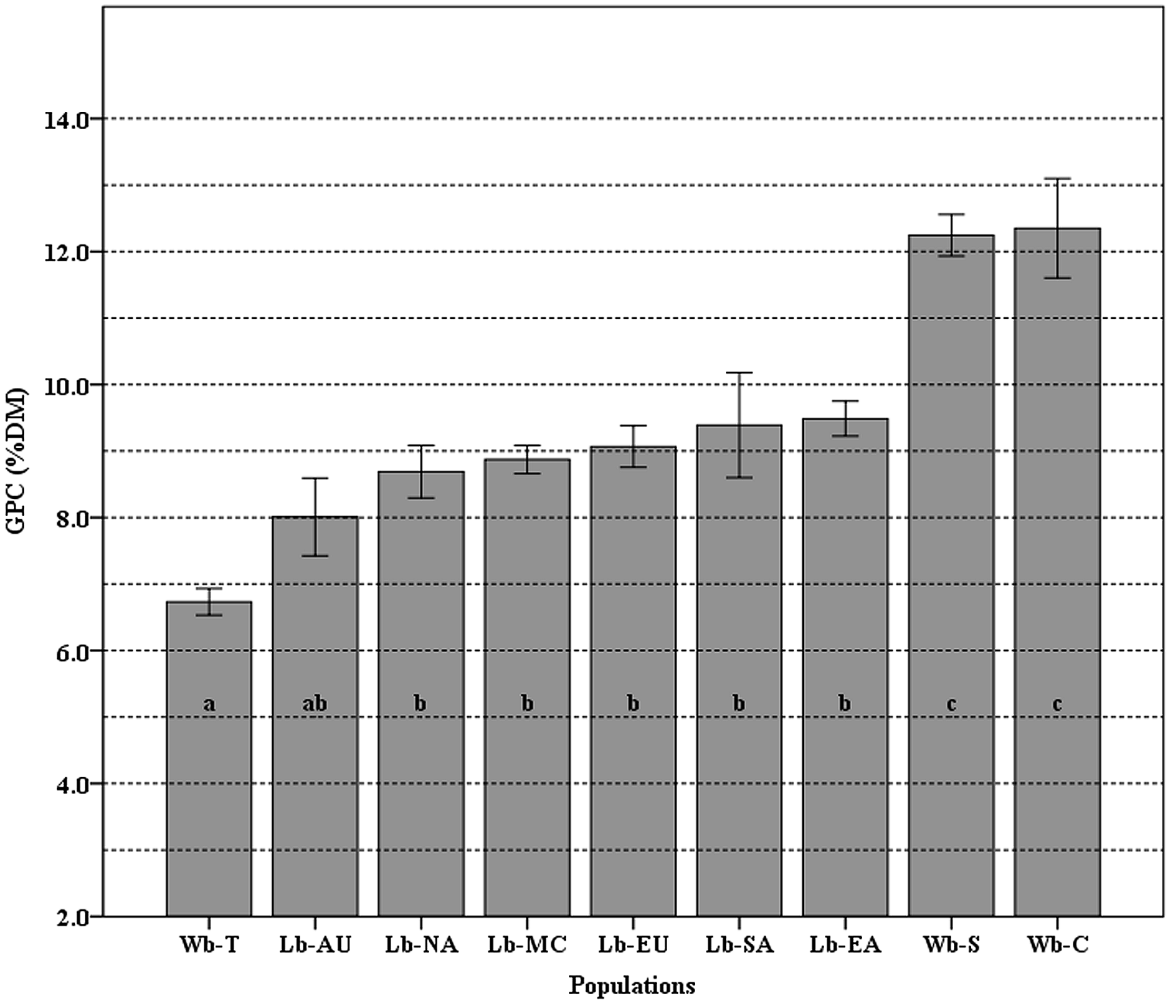
**The means of grain protein content (GPC) variation among nine populations.** Different letters (from a–c) on top of the histogram bars correspond to classes of which the population belongs, based on the Newman–Keuls test. Error bars indicate standard error. The populations used are Wb-T (Wild barley of Tibet), Wb-C (Wild barley of Central Asia), and Wb-S (Wild barley of Southwest Asia); Lb-EA (Landrace barley of East Asia), Lb-NA (Landraces of North America), Lb-SA (Landraces of South America), Lb-MA (Landraces of the Mediterranean Coast Areas), Lb-EU (Landraces of Europe), and Lb-AU (Landraces of Australia).

The mean value of GPC and the differences among populations are shown in **Figure 3**. The GPC in 118 cultivated and 93 wild barley accessions ranged from 6.73 to 12.35% with a mean of 9.43%. Overall, wild barley had higher averaged GPC (10.44%) than cultivated barley. A significantly statistical difference was found between the group of landraces and the group of Southwest Asian and Central Asian wild barleys; however, no significant difference was found within each of these.

### Association between SNP and GPC

**FIGURE 4:**
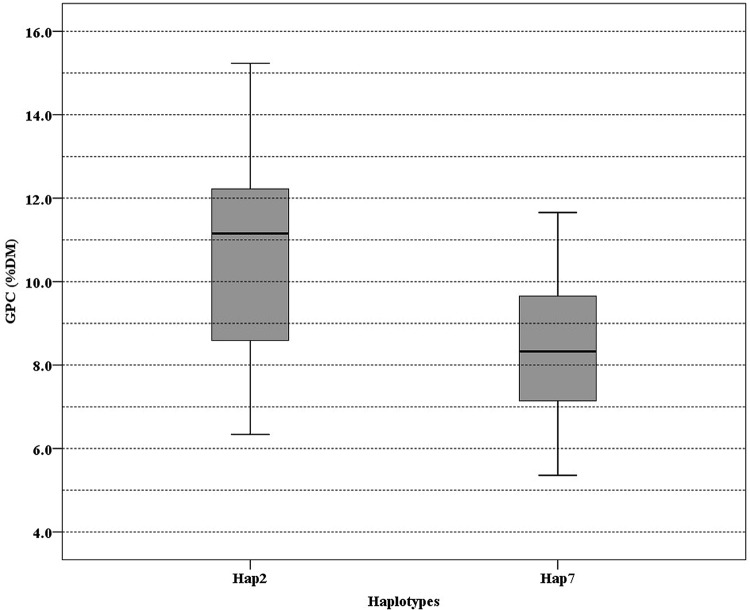
**Boxplot of grain protein content (GPC) variation among 176 barley accessions grouped according to the haplotype of Hap2 and Hap7.** Lines across the boxes depict the medians. Boxes indicate the interquartile range. Whiskers represent 95% confidence intervals.

To determine association between SNP of *NAM-1* and GPC among barley populations, a sequence from NCBI (accession number DQ869678) was used as a reference to identify SNP in our populations. The SNPs identified were summarized in **Table 4**. Three SNPs were recognized in the Tibetan wild barley population and are located at position 375, 544, and 1190, respectively; the SNPs at position 544 and 1190 are within the coding sequence. Seven SNPs were found in the Southwest Asian wild barley population, of which two SNPs (position 473 and 823) were located in a non-coding region, and five SNPs (position 507, 544, 616, 1190, and 1253) in coding; Only one SNP at position 544 within the coding sequence was obtained in the Central Asian wild barley population. Two SNPs were identified (position 507 and 544) from landraces, with one in a coding region. The haplotype 2 of *NAM-1* was associated with the highest GPC, while the haplotype 7 of *NAM-1* with lowest GPC (**Figure 4**).

**Table 4:** The single nucleotide polymorphism (SNP) and its positions relative to the reference sequence among distinct populations.

Population	No. SNP	375	473	507	544	616	823	1190	1253
Wb-T	3	T/A	–	–	G/C	–	–	G/A	–
Wb-S	7	–	A/G	C/T	C	C/A	A/C	G/A	T/C
Wb-C	2	–	–	C/T	C	–	–	–	–
Landraces	2	–	A/G	–	G/C	–	–	–	–

*The number and position of SNP relative to the reference sequence (DQ869678). A slash indicates two classes of bases in one population, the front one is same to reference and the behind is different; A horizontal dash indicates the absence of the SNP happens. The distinct populations as follows: Wb-T (Wild barley of Tibet), Wb-S (Wild barley of Southwest Asia), Wb-C (Wild barley of Central Asia), and worldwide landraces, respectively.*

## Discussion

### Tibetan Wild Barley Diverged from Southwest Asian Barley

Previous studies have reported clear genetic differentiation between oriental and occidental barleys (Kahler and Allard, 1981; Zhang et al., 1992a,b; Ma, 2002; Komatsuda et al., 2004; Azhaguvel and Komatsuda, 2007). Ma (2002) showed morphological difference between two-rowed wild barley of the Qinghai–Tibet Plateau and the Middle Eastern barley. Genome-wide diversity analysis has shown significant genetic differences between wild barley from the Near East and Tibet (Dai et al., 2012, 2014). Our *NAM-1* gene data revealed significant genetic differentiation among wild populations. Most dramatic differences in haplotype composition in wild barley occurred between the Tibetan and Southwest Asian barley, three haplotypes specific to the Tibetan wild barley population, and four haplotypes specific to the Southwest Asian wild barley population were detected (**Table 2**). No population-specific haplotype was found in wild barley from Central Asia. A higher genetic diversity and population-specific haplotype observed in the Tibetan wild barley, further supports that Tibetan wild barley is distinctly diverged from the Southwest Asian barley.

### The Origin of Cultivated Barley

It has been well recognized that the Near East Fertile Crescent (Harlan and Zohary, 1966; Badr et al., 2000; Zohary et al., 2012) and Central Asia (Morrell and Clegg, 2007; Saisho and Purugganan, 2007) are the primary evolutionary centers of wild barley as well as a domestication center of cultivated forms. However, increasing evidence suggested that Tibet of China is an additional domestication center of cultivated barley (Dai et al., 2012, 2014; Nevo, 2013). The present study not only supports the status of the Fertile Crescent in domestication of cultivated barley, but also reveals Tibet as one of the centers of domestication of cultivated barley, thus further supporting the concept of polyphyletic domestication of barley. Hypotheses concerning the origin of barley have suggested that the varieties growing in the original center generally contain large amounts of dominant genetic genes (Wang et al., 2009). The region with the highest level of genetic diversity in wild barley is also most likely the center of origin for cultivated barley (Wang et al., 2009). In our study, high levels of nucleotide diversity, haplotype diversity and number of haplotypes were detected in the Tibetan and Southwest Asian wild barley populations (**Table 3**). Furthermore, the haplotype analysis showed that the worldwide cultivars shared the same haplotypes with the Southwest Asia wild barley, and specially, the wild barley from the Tibet (**Table 2**; **Figure 1**). A close relationship between worldwide domesticated barley and the Tibetan wild barley was revealed in our study, suggesting that Tibetan wild barley is one of the ancestors of domesticated barley. Our results corroborated previous finding that cultivated barley is not only derived from wild-barley genotypes in the Fertile Crescent, but also from those in Tibet of China (Dai et al., 2014).

Where Chinese cultivated barley originated from still remains to be addressed. At present, two hypotheses have been suggested. One suggested the Chinese cultivated barley was introduced from the Near East (Harlan, 1971, 1976; Badr et al., 2000), and another indicated that Chinese cultivated barley might have originated from two-rowed or six-rowed wild barley from Tibet (Zhang et al., 1994; Feng et al., 2006). Recent genome-wide diversity array data suggested that Chinese hulless and six-rowed barleys were domesticated in the Tibetan Plateau and its vicinity (Dai et al., 2012). Our results showed that 29 out of 61 landraces shared the same haplotype (Hap7) with the Tibetan wild barley, and phylogenetic analysis also revealed a close relationship between them. Haplotype Hap2 in the rest of the Chinese landraces was not only present in the Tibetan wild barley population, but also in the Central Asian and Southwest Asian wild barley populations (**Table 2**). Thus, our results not only supported that the Tibetan wild barley is the ancestor of Chinese domesticated barley, but also suggested that the Near East Fertile Crescent wild barley might have contributed to the origin of Chinese cultivars. This is in agreement with previous findings that landraces with majority western ancestry were relatively commonly encountered among Asian samples (Morrell et al., 2013) and the Oriental landraces have high proportion of admixed ancestry (Morrell and Clegg, 2007).

### Implications for the Spread of Barley Cultivation in the World

Various hypotheses about the world spread of domesticated barley have been proposed. Badr et al. (2000) suggested that the border region between Israel and Jordan might be the region where barley was brought into cultivation and subsequently migrated to the area of the Himalayas. Some studies argued that barley was domesticated in this region and subsequently expanded westward into Europe and North Africa and eastward into Asia 8000 years ago (Von Bothmer et al., 2003). Morrell and Clegg (2007) proposed that the Fertile Crescent domestication contributed the majority of diversity in European and American cultivars, whereas the second domestication, 1500–3000 km farther east contributed most of the diversity in barley from Central Asia to the Far East. The trade of barley between the New World and Europe was supported by *eIF4E* gene data (Hofinger et al., 2011).

Our study provided interesting insights into historic global cultural / trade routes of barley. First, a haplotype that is private to the Southwest Asian wild barley population was also detected in the North American and European landrace barley populations (**Table 2**; **Figure 1**), corroborating assumptions made by Morrell and Clegg (2007) that Fertile Crescent domestication contributed the majority of diversity in European and American cultivars. In addition, we were surprised to see a haplotype that is exclusively found in Tibetan wild barley population is pervasive in all landrace populations (**Table 2**; **Figure 1**). It seems likely that the ancestral carrier(s) of this haplotype was initially introduced from the Tibet region to other geographic regions, which might explain the high levels of similarity between Eastern malting barley and European cultivars reported by Ordon et al. (1997). Our results suggested that the gene pool of Tibetan wild barley has been widely circulated, and has significantly contributed to the gene pool of global cultivated barley. Moreover, it may be assumed that Central Asia is the sole route for wild barley migration between the Near East and the Qinghai–Tibetan Plateau (Dai et al., 2012), as deduced in our study from haplotype Hap2 that was widespread and frequently found in Southwest Asia, Central Asia, and Tibetan wild barley populations, and worldwide cultivars (**Table 2**; **Figure 1**). Thus, our results supported the most likely scenario that the gene pool of the cultivated barley includes contributions of wild barleys from both the Near East and Tibet (Dai et al., 2012, 2014). Meanwhile, we suggest that the gene flow between Eastern and Western cultivars has occurred via the Silk Road, which started from China and moved westward, through the Eurasian civilization zones, Central Asia, and the Roman empire to Europe (Ma, 1998). The Silk Road might be an important barley transition route between the Orient and the Occident as previously proposed (Harold, 2007; Dai et al., 2012).

The spread of agriculture from domestication region involved the dispersal of crop plants well beyond their progenitors’ native range, and may have required adaptation to new environments (Jones et al., 2008). Ecologically, Tibetan wild barley is adapted to cold and dry environments, these characteristics may also be an important reason for its successfully spread all over the world (Dai et al., 2012).

### Natural Variation in Barley Population

Crop domestication is the outcome of complex independent or combined processes of artificial and natural selection that lead to plants adapted to cultivation and to meet the requirements of human consumption (Dai et al., 2014). Gene pools undergoing domestication experienced dramatic changes in allele frequencies due to genetic drift or selection, and some allelic combinations may be lost (Wang et al., 2014). In our studies, a total of 10 distinct haplotypes were discovered, only 3 haplotypes were detected in the diverse set of 120 domesticated barleys from across the world, while more haplotypes occurred in wild barley accessions. This result agreed with previous reports (Kilian et al., 2006; Jakob et al., 2014) and further confirmed that most alleles in wild types have been lost in the domesticated forms. In addition, reduction in haplotype diversity, nucleotide diversity, and pre-site nucleotide diversity in domesticated lines was in accord with previous findings that *H. spontaneum* has a higher genetic diversity than *H. vulgare* landraces (Russell et al., 2004; Jin et al., 2011; Jakob et al., 2014), which might be caused by genetic bottlenecks acting on neutrally evolving loci either during the domestication process or during subsequent breeding, or both (Badr and El-Shazly, 2012). A shift toward more positive values of Tajima’s *D* in the domesticated relative to wild populations is indicative of reduced genetic diversity in the domesticated forms (Hufford et al., 2012; Morrell et al., 2013). A similar pattern was observed in our study, where Tajima’s *D* values are negative in wild barley populations of Southwest Asia, while they are more positive in domesticated barley populations of East Asia, South America, Mediterranean Coast Areas, and Europe. This similar shift from negative Tajima’s *D* at the majority of loci in the wild toward positive values in landraces, was also mentioned by Morrell et al. (2013). However, a positive Tajima’s *D* observed in Tibetan wild barley populations (**Table 3**) might be due to the fact that a rare-allele advantage resulted in an accumulating allelic frequency up to an intermediate level may have been involved in balancing selection, thus causing a positive value of Tajima’s *D*, as suggested by Chung et al. (2010). In general, deviation from neutrality with Tajima’s *D* was not significant (at *P* > 0.05) for any barley populations in our study. Domesticated barley populations of North America and Australia showed high negative Tajima’s *D* values (**Table 3**), suggesting that purifying selection might act on these populations. Insignificance may be attributed to the low number of SNPs (**Table 4**) observed, which weakens the neutrality test (Xia et al., 2013).

### Association between GPC and *NAM-1* Gene

GPC, as a key factor for quality in cereals, is influenced to a large extent by both genotype and environment (Smith, 1990; Jaradat, 1991). In our study, GPC was significantly different between domesticated and the wild barley population of Southwest Asia and Central Asia. Our findings support the studies that showed *H. spontaneum* with higher GPC values than cultivated barley (Jaradat, 1991; Jamar et al., 2010). However, wild barley population of Tibet had a lower GPC, which contradicts the study by Cai et al. (2013).

GPC in barley is influenced by both genetic and environmental factors (Bertholdsson, 1999; Distelfeld et al., 2008; Jamar et al., 2010; Cai et al., 2013). That allelic variation of the *NAM-1* gene is an important genetic factor was demonstrated by Distelfeld et al. (2008), who found that two amino acid (aa) substitutions in *HvNAM-1* might be associated with the GPC in barley. Jamar et al. (2010) suggested that allelic variation of *NAM-1* gene might be associated with GPC variation in the genus *Hordeum.* Recent GWAS showed a significant correlation between haplotypes of *HvNAM1, HvNAM2*, and GPC in barley (Cai et al., 2013). In our study, two unique haplotypes (Hap2 and Hap7) might have a significant impact on the GPC (**Table 4**; **Figure 4**; Supplementary Data Sheet S2). The SNP at position 544 is within the coding region and causes a non-synonymous change with aa substitution between Alanine (A) and Proline (P). This substitution occurred in the C subdomain of NAC domain in the N-terminal, and may have an impact on protein folding (Jamar et al., 2010). The SNP at position 544 was also identified by Cai et al. (2013), in which the 102th aa proline (P) was replaced by alanine (A), resulting in low GPC. Therefore, we suggested that aa substitution in Hap2 and Hap7 might have an impact on DNA-binding ability of *NAM-1* gene, and further affect GPC. In addition, the differences in GPC of a particular group with no polymorphisms at the *NAM-1* gene might suggest that expression of the *NAM-1* gene or other genes are also important (Jamar et al., 2010).

In summary, our results showed significant genetic differentiation among wild populations. Our data supported that Tibet is a center of origin and domestication centre for cultivated barleys, and suggested that the Silk Road might have played an important role in gene flow between Eastern and Western barley. Moreover, SNPs and haplotypes of *NAM-1* associated with GPC in barley could provide a useful method for screening GPC in barley germplasm. The Tibetan wild accessions with lower GPC could be useful for malt barley breeding.

## Author Contributions

Conceived and designed the experiments: DS, GS. Performed the experiments: YW, XR. Analyzed the data: YW, XR, DS, and GS. Contributed reagents/materials/analysis tools: YW, XR, DS, and GS. Wrote the paper: YW, XR, DS, and GS.

## Conflict of Interest Statement

The authors declare that the research was conducted in the absence of any commercial or financial relationships that could be construed as a potential conflict of interest.
