# Supplementary material for: Origin of worldwide cultivated barley revealed by NAM-1 gene and grain protein content
Source: Front Plant Sci. 2015 Sep 30;6:803. doi: 10.3389/fpls.2015.00803 (PMC4588695; doi:10.3389/fpls.2015.00803)
Supplement: Supplementary file 1 [file Table_1.DOC]

Table S1

| Species |  | Accession No. | Origin (Country) | GenBank Accession No. |
| --- | --- | --- | --- | --- |
|  | HS1 | PI212305 | Afghanistan | KT779934 |
|  | HS2 | PI212306 | Afghanistan | KT779935 |
|  | HS3 | PI219796 | Iraq | KT779936 |
|  | HS4 | PI220664 | Afghanistan | KT779937 |
|  | HS5 | PI227019 | Iran | KT779938 |
|  | HS7 | PI236386 | Syria | KT779939 |
|  | HS8 | PI244772 | Pakistan | KT779940 |
|  | HS9 | PI244774 | Afghanistan | KT779941 |
|  | HS10 | PI244776 | Afghanistan | KT779942 |
|  | HS11 | PI244777 | Afghanistan | KT779943 |
|  | HS12 | PI245739 | Turkey | KT779944 |
|  | HS13 | PI253933 | Iraq | KT779945 |
|  | HS14 | PI254894 | Iraq | KT779946 |
|  | HS15 | PI268243 | Iran | KT779947 |
|  | HS18 | PI284752 | Israel | KT779948 |
|  | HS19 | PI293411 | Tajikistan | KT779949 |
|  | HS20 | PI293412 | Tajikistan | KT779950 |
|  | HS21 | PI296413 | Azerbaijan | KT779951 |
|  | HS22 | PI293414 | Azerbaijan | KT779952 |
|  | HS23 | PI296792 | Israel | KT779953 |
|  | HS24 | PI296849 | Israel | KT779954 |
|  | HS25 | PI296862 | Israel | KT779955 |
|  | HS26 | PI296878 | Israel | KT779956 |
|  | HS27 | PI296908 | Israel | KT779957 |
|  | HS28 | PI354948 | Israel | KT779958 |
|  | HS29 | PI356061 | Ethiopia | KT779959 |
|  | HS30 | PI356209 | Ethiopia | KT779960 |
|  | HS31 | PI391100 | Israel | KT779961 |
|  | HS32 | PI401368 | Iran | KT779962 |
|  | HS33 | PI401371 | Iran | KT779963 |
|  | HS34 | PI420911 | Jordan | KT779964 |
|  | HS35 | PI420912 | Jordan | KT779965 |
|  | HS36 | PI420913 | Jordan | KT779966 |
|  | HS37 | PI420915 | Jordan | KT779967 |
|  | HS38 | PI420916 | Jordan | KT779968 |
|  | HS39 | PI420917 | Jordan | KT779969 |
|  | HS40 | PI466040 | Syria | KT779970 |
|  | HS41 | PI466048 | Syria | KT779971 |
|  | HS42 | PI466060 | Syria | KT779972 |
|  | HS43 | PI466086 | Syria | KT779973 |
|  | HS44 | PI466118 | Syria | KT779974 |
|  | HS45 | PI466130 | Syria | KT779975 |
|  | HS46 | PI466178 | Syria | KT779976 |
|  | HS47 | PI466206 | Syria | KT779977 |
|  | HS48 | PI466238 | Syria | KT779978 |
|  | HS49 | PI466249 | Lebanon | KT779979 |
|  | HS50 | PI466256 | Lebanon | KT779980 |
|  | HS51 | PI466264 | Lebanon | KT779981 |
|  | HS52 | PI466296 | Israel | KT779982 |
|  | HS53 | PI466328 | Israel | KT779983 |
|  | HS54 | PI466388 | Israel | KT779984 |
|  | HS55 | PI466498 | Israel | KT779985 |
|  | HS56 | PI466524 | Israel | KT779986 |
|  | HS57 | PI466554 | Israel | KT779987 |
|  | HS58 | PI466586 | Israel | KT779988 |
|  | HS59 | PI466605 | Iran | KT779989 |
|  | HS60 | PI466618 | Iran | KT779990 |
|  | HS63 | PI554426 | Turkey | KT779991 |
|  | HS64 | PI466632 | Iran | KT779992 |
|  | HS65 | PI466699 | Iran | KT779993 |
|  | HS66 | PI554428 | Turkey | KT779994 |
|  | HS67 | PI559556 | Turkey | KT779995 |
|  | HS68 | PI662052 | Tajikistan | KT779996 |
|  | HS69 | PI662080 | Tajikistan | KT779997 |
|  | HS70 | PI662109 | Tajikistan | KT779998 |
|  | HS71 | PI662118 | Tajikistan | KT779999 |
|  | HS72 | PI662138 | Turkey | KT780000 |
|  | HS73 | PI662158 | Turkey | KT780001 |
|  | HS74 | PI662170 | Turkey | KT780002 |
|  | HS75 | PI662178 | Turkey | KT780003 |
|  | HS76 | PI662188 | Turkey | KT780004 |
|  | HS77 | PI662204 | Turkey | KT780005 |
|  | HS78 | PI662214 | Turkey | KT780006 |
|  | HS79 | PI662218 | Turkey | KT780007 |
|  | HS80 |  | China | KT780008 |
|  | HS81 |  | China | KT780009 |
|  | HS82 |  | China | KT780010 |
|  | HS83 |  | China | KT780011 |
|  | HS84 |  | China | KT780012 |
|  | HS85 |  | China | KT780013 |
|  | HS86 |  | China | KT780014 |
|  | HS87 |  | China | KT780015 |
|  | HS88 |  | China | KT780016 |
|  | HS89 |  | China | KT780017 |
|  | HS90 |  | China | KT780018 |
|  | HS91 |  | China | KT780019 |
|  | HS92 |  | China | KT780020 |
|  | HS93 |  | China | KT780021 |
|  | HS94 |  | China | KT780022 |
|  | HS95 |  | China | KT780023 |
|  | HS96 |  | China | KT780024 |
|  | HS97 |  | China | KT780025 |
|  | HS98 |  | China | KT780026 |
|  | HS99 |  | China | KT780027 |
|  | HS100 |  | China | KT780028 |
|  | HS101 |  | China | KT780029 |
|  | HS102 |  | China | KT780030 |
|  | HS103 |  | China | KT780031 |
|  | HS104 |  | China | KT780032 |
|  | HS105 |  | China | KT780033 |
|  | HS106 |  | China | KT780034 |
|  | HS107 |  | China | KT780035 |
|  | HS108 |  | China | KT780036 |
|  | HS109 |  | China | KT780037 |
|  | HS110 |  | China | KT780038 |
|  | HS111 |  | China | KT780039 |
|  | HS112 |  | China | KT780040 |
|  | HS113 |  | China | KT780041 |
|  | HS114 |  | China | KT780042 |
|  | HS115 |  | China | KT780043 |
|  | HS116 |  | China | KT780044 |
|  | HS117 |  | China | KT780045 |
|  | HS118 |  | China | KT780046 |
|  | HS119  hzd67  hzd68  hzd69  hzd71  hzd79  hzd80  hzd81  hzd82  hzd83  hzd84  hzd85  hzd86  hzd87  hzd98  hzd99  hzd140  hzd141  hzd142  hzd143  hzd144  hzd145  hzd146  hzd147  hzd148  hzd149  hzd175  hzd189  hzd190  hzd191  hzd192  hzd193  hzd194  hzd195  hzd200  hzd201  hzd202  hzd206  hzd208  hzd209  hzd210  hzd213  hzd214  hzd215  hzd216  hzd217  hzd242  hzd247  hzd263  hzd265  hzd288  hzd290  hzd294  hzd295  hzd296  hzd297  hzd298  hzd299  hzd310  hzd311  hzd312  hzd313  hzd314  hzd328  hzd329  hzd330  hzd332  hzd333  hzd334  hzd335  hzd336  hzd378  hzd379  hzd380  hzd381  hzd382  hzd406  hzd407  hzd408  hzd409  hzd416  hzd417  hzd418  hzd419  hzd420  hzd424  hzd425  hzd426  hzd427  hzd428  hzd429  hzd430  hzd489  hzd490  hzd491  hzd494  hzd495  hzd533  hzd539  hzd540  hzd541  hzd542  hzd543  hzd558  hzd560 |  | China  China  China  China  China  Canada  Canada  Canada  China  China  China  China  China  China  Australia  Australia  U.S.A  U.S.A  U.S.A  U.S.A  U.S.A  U.S.A  U.S.A  U.S.A  U.S.A  U.S.A  China  Syria  Syria  Syria  Syria  Syria  Syria  Syria  Rumania  Rumania  Australia  Egypt  Columbia  U.K  France  Sweden  Sweden  Sweden  Sweden  Sweden  China  China  China  China  Australia  China  China  Japan  Japan  Japan  Japan  Japan  China  China  China  China  China  Netherland  Netherland  Netherland  Canada  Canada  Canada  Canada  Canada  China  China  China  China  China  China  China  China  China  Russia  Russia  Russia  Australia  Mexico  Mexico  Mexico  Mexico  Mexico  Mexico  Turkey  Turkey  China  China  China  China  China  Mexico  Germany  Germany  Germany  Germany  Germany  France  Hungary | KT780047  KT780048  KT780049  KT780050  KT780051  KT780052  KT780053  KT780054  KT780055  KT780056  KT780057  KT780058  KT780059  KT780060  KT780061  KT780062  KT780063  KT780064  KT780065  KT780066  KT780067  KT780068  KT780069  KT780070  KT780071  KT780072  KT780073  KT780074  KT780075  KT780076  KT780077  KT780078  KT780079  KT780080  KT780081  KT780082  KT780083  KT780084  KT780085  KT780086  KT780087  KT780088  KT780089  KT780090  KT780091  KT780092  KT780093  KT780094  KT780095  KT780096  KT780097  KT780098  KT780099  KT780100  KT780101  KT780102  KT780103  KT780104  KT780105  KT780106  KT780107  KT780108  KT780109  KT780110  KT780111  KT780112  KT780113  KT780114  KT780115  KT780116  KT780117  KT780118  KT780119  KT780120  KT780121  KT780122  KT780123  KT780124  KT780125  KT780126  KT780127  KT780128  KT780129  KT780130  KT780131  KT780132  KT780133  KT780134  KT780135  KT780136  KT780137  KT780138  KT780139  KT780140  KT780141  KT780142  KT780143  KT780144  KT780145  KT780146  KT780147 |
