# Supplementary material for: Origin of worldwide cultivated barley revealed by NAM-1 gene and grain protein content
Source: Front Plant Sci. 2015 Sep 30;6:803. doi: 10.3389/fpls.2015.00803 (PMC4588695; doi:10.3389/fpls.2015.00803)
Supplement: Supplementary file 2 [file Data_Sheet_1.DOCX]

***Supplementary Material***

**Origin of worldwide cultivated barley revealed by *NAM-1* gene and grain protein content**

**Yonggang Wang, Xifeng Ren, Dongfa Sun*, Genlou Sun***

*** Correspondence:**

Dongfa Sun: [sundongfa1@mail.hzau.edu.cn](mailto:sundongfa1@mail.hzau.edu.cn)

Genlou Sun: [genlou.sun@smu.ca](mailto:genlou.sun@smu.ca)

**Supplementary Data Sheet 1:** **Partial sequences of *NAM-1* genes in 214 barley accessions.** All available information of codes, accession numbers and geographical origins is described in Supplementary Table S1.

>HS1_*NAM-1*

GCTTTTTATTATACTGTGCACAAGTATTTTTATATTCTTCCAGTAAGTACAGCGCATGTATGTGATCCTGTCGTCGTGCTTGTTCATGCGCTCGGGCGGGATCATCATCCATCAGAGAAGGCGACCTTCGGGGAGCACGAGTGGTACTTCTTCAGCCCGCGCGACCGCAAGTACCCCAACGGCGCGCGGCCGAACCGGGCGGCGACGTCGGGCTACTGGAAGGCCACCGGCACGGACAAGCCTATCCTGGCCTCGGCCACCGGGTGCGGCCGGGAGAAGGTCGGCGTCAAGAAGGCGCTCGTCTTCTACCGCGGGAAGCCGCCCAGGGGCCTCAAGACCAACTGGATCATGCATGAGTACCGCCTCACCGGAGCCTCTGCTGGCTCCACCACCACCAGCCGGCCGCCGCCGGTGACCGGCGGGAGCAGGGCCCCGGCCTCTCTCAGGGTACGTACTTACACGTGTCCATCGCACGGTCTATCAGTATTTATTTATTAACTACTCTCGAGCTTAATTATGGTATTGTTGATAGTTGATGAAGTTAATTATTGTACGCCGTCTCATCGATCAGTTGGACGACTGGGTGCTGTGCCGCATCTACAAGAAGACCAGCAAGGCCGCGGCCGCGGTCGGAGATGAGCAGAGGAGCATGGAGTGCGAGGACTCCGTGGAGGACGCGGTCACCGCGTACCCGCCCTACGCCACGGCGGGCATGGCCGGCGCAGGTGCGCATGGCAGCAACTACGTTCAACTGCTCCATCATCACGACAGCCACGAGGACAACTTCCAGCTAGACGGCCTGCTCACAGAACACGACGTCGGCCTCTCGGCGGGCGCCGCCTCGCTGGGCCACCTTGCCGCGGCGGCGAGGGCCACCAAACAGTTCCTCGCCCCGTCGTCCTCAACCCCGTTCAACTGGCTCGAGGCGTCAACCGG

>HS2_*NAM-1*

GCTTTTTATTATACTGTGCACAAGTATTTTTATATTCTTCCAGTAAGTACAGCGCATGTATGTGATCCTGTCGTCGTGCTTGTTCATGCGCTCGGGCGGGATCATCATCCATCAGAGAAGGCGACCTTCGGGGAGCACGAGTGGTACTTCTTCAGCCCGCGCGACCGCAAGTACCCCAACGGCGCGCGGCCGAACCGGGCGGCGACGTCGGGCTACTGGAAGGCCACCGGCACGGACAAGCCTATCCTGGCCTCGGCCACCGGGTGCGGCCGGGAGAAGGTCGGCGTCAAGAAGGCGCTCGTCTTCTACCGCGGGAAGCCGCCCAGGGGCCTCAAGACCAACTGGATCATGCATGAGTACCGCCTCACCGGAGCCTCTGCTGGCTCCACCACCACCAGCCGGCCGCCGCCGGTGACCGGCGGGAGCAGGGCCCCGGCCTCTCTCAGGGTACGTACTTACACGTGTCCATCGCACGGTCTATCAGTATTTATTTATTAACTACTCTCGAGCTTAATTATGGTATTGTTGATAGTTGATGAAGTTAATTATTGTACGCCGTCTCATCGATCAGTTGGACGACTGGGTGCTGTGCCGCATCTACAAGAAGACCAGCAAGGCCGCGGCCGCGGTCGGAGATGAGCAGAGGAGCATGGAGTGCGAGGACTCCGTGGAGGACGCGGTCACCGCGTACCCGCCCTACGCCACGGCGGGCATGGCCGGCGCAGGTGCGCATGGCAGCAACTACGTTCAACTGCTCCATCATCACGACAGCCACGAGGACAACTTCCAGCTAGACGGCCTGCTCACAGAACACGACGTCGGCCTCTCGGCGGGCGCCGCCTCGCTGGGCCACCTTGCCGCGGCGGCGAGGGCCACCAAACAGTTCCTCGCCCCGTCGTCCTCAACCCCGTTCAACTGGCTCGAGGCGTCAACCGG

>HS3*_NAM-1*

GCTTTTTATTATACTGTGCACAAGTATTTTTATATTCTTCCAGTAAGTACAGCGCATGTATGTGATCCTGTCGTCGTGCTTGTTCATGCGCTCGGGCGGGATCATCATCCATCAGAGAAGGCGACCTTCGGGGAGCACGAGTGGTACTTCTTCAGCCCGCGCGACCGCAAGTACCCCAACGGCGCGCGGCCGAACCGGGCGGCGACGTCGGGCTACTGGAAGGCCACCGGCACGGACAAGCCTATCCTGGCCTCGGCCACCGGGTGCGGCCGGGAGAAGGTCGGCGTCAAGAAGGCGCTCGTCTTCTACCGCGGGAAGCCGCCCAGGGGCCTCAAGACCAACTGGATCATGCATGAGTACCGCCTCACCGGAGCCTCTGCTGGCTCCACCACCACCAGCCGGCCGCCGCCGGTGACCGGCGGGAGCAGGGCCCCGGCCTCTCTCAGGGTACGTACTTACACGTGTCCATCGCACGGTCTATCAGTATTTATTTATTAACTACTCTCGAGCTTAATTATGGTATTGTTGATAGTTGATGAAGTTAATTATTGTACGCCGTCTCATCGATCAGTTGGACGACTGGGTGCTGTGCCGCATCTACAAGAAGACCAGCAAGGCCGCGGCCGCGGTCGGAGATGAGCAGAGGAGCATGGAGTGCGAGGACTCCGTGGAGGACGCGGTCACCGCGTACCCGCCCTACGCCACGGCGGGCATGGCCGGCGCAGGTGCGCATGGCAGCAACTACGTTCAACTGCTCCATCATCACGACAGCCACGAGGACAACTTCCAGCTAGACGGCCTGCTCACAGAACACGACGTCGGCCTCTCGGCGGGCGCCGCCTCGCTGGGCCACCTTGCCGCGGCGGCGAGGGCCACCAAACAGCTCCTCGCCCCGTCGTCCTCAACCCCGTTCAACTGGCTCGAGGCGTCAACCGG

>HS4*_NAM-1*

GCTTTTTATTATACTGTGCACAAGTATTTTTATATTCTTCCAGTAAGTACAGCGCATGTATGTGATCCTGTCGTCGTGCTTGTTCATGCGCTCGGGCGGGATCATCATCCATCAGAGAAGGCGACCTTCGGGGAGCACGAGTGGTACTTCTTCAGCCCGCGCGACCGCAAGTACCCCAACGGCGCGCGGCCGAACCGGGCGGCGACGTCGGGCTACTGGAAGGCCACCGGCACGGACAAGCCTATCCTGGCCTCGGCCACCGGGTGCGGCCGGGAGAAGGTCGGCGTCAAGAAGGCGCTCGTCTTCTACCGCGGGAAGCCGCCCAGGGGCCTCAAGACCAACTGGATCATGCATGAGTACCGCCTCACCGGAGCCTCTGCTGGCTCCACCACCACCAGCCGGCCGCCGCCGGTGACCGGCGGGAGCAGGGCCCCGGCCTCTCTCAGGGTACGTACTTACACGTGTCCATCGCACGGTCTATCAGTATTTATTTATTAACTACTCTCGAGCTTAATTATGGTATTGTTGATAGTTGATGAAGTTAATTATTGTACGCCGTCTCATCGATCAGTTGGACGACTGGGTGCTGTGCCGCATCTACAAGAAGACCAGCAAGGCCGCGGCCGCGGTCGGAGATGAGCAGAGGAGCATGGAGTGCGAGGACTCCGTGGAGGACGCGGTCACCGCGTACCCGCCCTACGCCACGGCGGGCATGGCCGGCGCAGGTGCGCATGGCAGCAACTACGTTCAACTGCTCCATCATCACGACAGCCACGAGGACAACTTCCAGCTAGACGGCCTGCTCACAGAACACGACGTCGGCCTCTCGGCGGGCGCCGCCTCGCTGGGCCACCTTGCCGCGGCGGCGAGGGCCACCAAACAGTTCCTCGCCCCGTCGTCCTCAACCCCGTTCAACTGGCTCGAGGCGTCAACCGG

>HS5*_NAM-1*

GCTTTTTATTATACTGTGCACAAGTATTTTTATATTCTTCCAGTAAGTACAGCGCATGTATGTGATCCTGTCGTCGTGCTTGTTCATGCGCTCGGGCGGGATCATCATCCATCAGAGAAGGCGACCTTCGGGGAGCACGAGTGGTACTTCTTCAGCCCGCGCGACCGCAAGTACCCCAACGGCGCGCGGCCGAACCGGGCGGCGACGTCGGGCTACTGGAAGGCCACCGGCACGGACAAGCCTATCCTGGCCTCGGCCACCGGGTGCGGCCGGGAGAAGGTCGGCGTCAAGAAGGCGCTCGTCTTCTACCGCGGGAAGCCGCCCAGGGGCCTCAAGACCAACTGGATCATGCATGAGTACCGCCTCACCGGAGCCTCTGCTGGCTCCACCACCACCAGCCGGCCGCCGCCGGTGACCGGCGGGAGCAGGGCCCCGGCCTCTCTCAGGGTACGTACTTACACGTGTCCATCGCACGGTCTATCAGTATTTATTTATTAACTACTCTCGAGCTTAATTATGGTATTGTTGATAGTTGATGAAGTTAATTATTGTACGCCGTCTCATCGATCAGTTGGACGACTGGGTGCTGTGCCGCATCTACAAGAAGACCAGCAAGGCCGCGGCCGCGGTCGGAGATGAGCAGAGGAGCATGGAGTGCGAGGACTCCGTGGAGGACGCGGTCACCGCGTACCCGCCCTACGCCACGGCGGGCATGGCCGGCGCAGGTGCGCATGGCAGCAACTACGTTCAACTGCTCCATCATCACGACAGCCACGAGGACAACTTCCAGCTAGACGGCCTGCTCACAGAACACGACGTCGGCCTCTCGGCGGGCGCCGCCTCGCTGGGCCACCTTGCCGCGGCGGCGAGGGCCACCAAACAGTTCCTCGCCCCGTCGTCCTCAACCCCGTTCAACTGGCTCGAGGCGTCAACCGG

>HS7*_NAM-1*

GCTTTTTATTATACTGTGCACAAGTATTTTTATATTCTTCCAGTAAGTACAGCGCATGTATGTGATCCTGTCGTCGTGCTTGTTCATGCGCTCGGGCGGGATCATCATCCATCAGAGAAGGCGACCTTCGGGGAGCACGAGTGGTACTTCTTCAGCCCGCGCGACCGCAAGTACCCCAACGGCGCGCGGCCGAACCGGGCGGCGACGTCGGGCTACTGGAAGGCCACCGGCACGGACAAGCCTATCCTGGCCTCGGCCACCGGGTGCGGCCGGGAGAAGGTCGGCGTCAAGAAGGCGCTCGTCTTCTACCGCGGGAAGCCGCCCAGGGGCCTCAAGACCAACTGGATCATGCATGAGTACCGCCTCACCGGAGCCTCTGCTGGCTCCACCACCACCAGCCGGCCGCCGCCGGTGACCGGCGGGAGCAGGGCCCCGGCCTCTCTCAGGGTACGTACTTACACGTGTCCATCGCACGGTCTATCAGTATTTATTTATTAACTACTCTCGAGCTTAATTATGGTATTGTTGATAGTTGATGAAGTTAATTATTGTACGCCGTCTCATCGATCAGTTGGACGACTGGGTGCTGTGCCGCATCTACAAGAAGACCAGCAAGGCCGCGGCCGCGGTCGGAGATGAGCAGAGGAGCATGGAGTGCGAGGACTCCGTGGAGGACGCGGTCACCGCGTACCCGCCCTACGCCACGGCGGGCATGGCCGGCGCAGGTGCGCATGGCAGCAACTACGTTCAACTGCTCCATCATCACGACAGCCACGAGGACAACTTCCAGCTAGACGGCCTGCTCACAGAACACGACGTCGGCCTCTCGGCGGGCGCCGCCTCGCTGGGCCACCTTGCCGCGGCGGCGAGGGCCACCAAACAGTTCCTCGCCCCGTCGTCCTCAACCCCGTTCAACTGGCTCGAGGCGTCAACCGG

>HS8*_NAM-1*

GCTTTTTATTATACTGTGCACAAGTATTTTTATATTCTTCCAGTAAGTACAGCGCATGTATGTGATCCTGTCGTCGTGCTTGTTCATGCGCTCGGGCGGGATCATCATCCATCAGAGAAGGCGACCTTCGGGGAGCACGAGTGGTACTTCTTCAGCCCGCGCGACCGCAAGTACCCCAACGGCGCGCGGCCGAACCGGGCGGCGACGTCGGGCTACTGGAAGGCCACCGGCACGGACAAGCCTATCCTGGCCTCGGCCACCGGGTGCGGCCGGGAGAAGGTCGGCGTCAAGAAGGCGCTCGTCTTCTACCGCGGGAAGCCGCCCAGGGGCCTCAAGACCAACTGGATCATGCATGAGTACCGCCTCACCGGAGCCTCTGCTGGCTCCACCACCACCAGCCGGCCGCCGCCGGTGACCGGCGGGAGCAGGGCCCCGGCCTCTCTCAGGGTACGTACTTACACGTGTCCATCGCACGGTCTATCAGTATTTATTTATTAACTACTCTCGAGCTTAATTATGGTATTGTTGATAGTTGATGAAGTTAATTATTGTACGCCGTCTCATCGATCAGTTGGACGACTGGGTGCTGTGCCGCATCTACAAGAAGACCAGCAAGGCCGCGGCCGCGGTCGGAGATGAGCAGAGGAGCATGGAGTGCGAGGACTCCGTGGAGGACGCGGTCACCGCGTACCCGCCCTACGCCACGGCGGGCATGGCCGGCGCAGGTGCGCATGGCAGCAACTACGTTCAACTGCTCCATCATCACGACAGCCACGAGGACAACTTCCAGCTAGACGGCCTGCTCACAGAACACGACGTCGGCCTCTCGGCGGGCGCCGCCTCGCTGGGCCACCTTGCCGCGGCGGCGAGGGCCACCAAACAGTTCCTCGCCCCGTCGTCCTCAACCCCGTTCAACTGGCTCGAGGCGTCAACCGG

>HS9*_NAM-1*

GCTTTTTATTATACTGTGCACAAGTATTTTTATATTCTTCCAGTAAGTACAGCGCATGTATGTGATCCTGTCGTCGTGCTTGTTCATGCGCTCGGGCGGGATCATCATCCATCAGAGAAGGCGACCTTCGGGGAGCACGAGTGGTACTTCTTCAGCCCGCGCGACCGCAAGTACCCCAACGGCGCGCGGCCGAACCGGGCGGCGACGTCGGGCTACTGGAAGGCCACCGGCACGGACAAGCCTATCCTGGCCTCGGCCACCGGGTGCGGCCGGGAGAAGGTCGGCGTCAAGAAGGCGCTCGTCTTCTACCGCGGGAAGCCGCCCAGGGGCCTCAAGACCAACTGGATCATGCATGAGTACCGCCTCACCGGAGCCTCTGCTGGCTCCACCACCACCAGCCGGCCGCCGCCGGTGACCGGCGGGAGCAGGGCCCCGGCCTCTCTCAGGGTACGTACTTACACGTGTCCATCGCACGGTCTATCAGTATTTATTTATTAACTACTCTCGAGCTTAATTATGGTATTGTTGATAGTTGATGAAGTTAATTATTGTACGCCGTCTCATCGATCAGTTGGACGACTGGGTGCTGTGCCGCATCTACAAGAAGACCAGCAAGGCCGCGGCCGCGGTCGGAGATGAGCAGAGGAGCATGGAGTGCGAGGACTCCGTGGAGGACGCGGTCACCGCGTACCCGCCCTACGCCACGGCGGGCATGGCCGGCGCAGGTGCGCATGGCAGCAACTACGTTCAACTGCTCCATCATCACGACAGCCACGAGGACAACTTCCAGCTAGACGGCCTGCTCACAGAACACGACGTCGGCCTCTCGGCGGGCGCCGCCTCGCTGGGCCACCTTGCCGCGGCGGCGAGGGCCACCAAACAGTTCCTCGCCCCGTCGTCCTCAACCCCGTTCAACTGGCTCGAGGCGTCAACCGG

>HS10*_NAM-1*

GCTTTTTATTATACTGTGCACAAGTATTTTTATATTCTTCCAGTAAGTACAGCGCATGTATGTGATCCTGTCGTCGTGCTTGTTCATGCGCTCGGGCGGGATCATCATCCATCAGAGAAGGCGACCTTCGGGGAGCACGAGTGGTACTTCTTCAGCCCGCGCGACCGCAAGTACCCCAACGGCGCGCGGCCGAACCGGGCGGCGACGTCGGGCTACTGGAAGGCCACCGGCACGGACAAGCCTATCCTGGCCTCGGCCACCGGGTGCGGCCGGGAGAAGGTCGGCGTCAAGAAGGCGCTCGTCTTCTACCGCGGGAAGCCGCCCAGGGGCCTCAAGACCAACTGGATCATGCATGAGTACCGCCTCACCGGAGCCTCTGCTGGCTCCACCACCACCAGCCGGCCGCCGCCGGTGACCGGCGGGAGCAGGGCCCCGGCCTCTCTCAGGGTACGTACTTACACGTGTCCATCGCACGGTCTATCAGTATTTATTTATTAACTACTCTCGAGCTTAATTATGGTATTGTTGATAGTTGATGAAGTTAATTATTGTACGCCGTCTCATCGATCAGTTGGACGACTGGGTGCTGTGCCGCATCTACAAGAAGACCAGCAAGGCCGCGGCCGCGGTCGGAGATGAGCAGAGGAGCATGGAGTGCGAGGACTCCGTGGAGGACGCGGTCACCGCGTACCCGCCCTACGCCACGGCGGGCATGGCCGGCGCAGGTGCGCATGGCAGCAACTACGTTCAACTGCTCCATCATCACGACAGCCACGAGGACAACTTCCAGCTAGACGGCCTGCTCACAGAACACGACGTCGGCCTCTCGGCGGGCGCCGCCTCGCTGGGCCACCTTGCCGCGGCGGCGAGGGCCACCAAACAGTTCCTCGCCCCGTCGTCCTCAACCCCGTTCAACTGGCTCGAGGCGTCAACCGG

>HS11*_NAM-1*

GCTTTTTATTATACTGTGCACAAGTATTTTTATATTCTTCCAGTAAGTACAGCGCATGTATGTGATCCTGTCGTCGTGCTTGTTCATGCGCTCGGGCGGGATCATCATCCATCAGAGAAGGCGACCTTCGGGGAGCACGAGTGGTACTTCTTCAGCCCGCGCGACCGCAAGTACCCCAACGGCGCGCGGCCGAACCGGGCGGCGACGTCGGGCTACTGGAAGGCCACCGGCACGGACAAGCCTATCCTGGCCTCGGCCACCGGGTGCGGCCGGGAGAAGGTCGGCGTCAAGAAGGCGCTCGTCTTCTACCGCGGGAAGCCGCCCAGGGGCCTCAAGACCAACTGGATCATGCATGAGTACCGCCTCACCGGAGCCTCTGCTGGCTCCACCACCACCAGCCGGCCGCCGCCGGTGACCGGCGGGAGCAGGGCCCCGGCCTCTCTCAGGGTACGTACTTACACGTGTCCATCGCACGGTCTATCAGTATTTATTTATTAACTACTCTCGAGCTTAATTATGGTATTGTTGATAGTTGATGAAGTTAATTATTGTACGCCGTCTCATCGATCAGTTGGACGACTGGGTGCTGTGCCGCATCTACAAGAAGACCAGCAAGGCCGCGGCCGCGGTCGGAGATGAGCAGAGGAGCATGGAGTGCGAGGACTCCGTGGAGGACGCGGTCACCGCGTACCCGCCCTACGCCACGGCGGGCATGGCCGGCGCAGGTGCGCATGGCAGCAACTACGTTCAACTGCTCCATCATCACGACAGCCACGAGGACAACTTCCAGCTAGACGGCCTGCTCACAGAACACGACGTCGGCCTCTCGGCGGGCGCCGCCTCGCTGGGCCACCTTGCCGCGGCGGCGAGGGCCACCAAACAGTTCCTCGCCCCGTCGTCCTCAACCCCGTTCAACTGGCTCGAGGCGTCAACCGG

>HS12_*NAM-1*

GCTTTTTATTATACTGTGCACAAGTATTTTTATATTCTTCCAGTAAGTACAGCGCATGTATGTGATCCTGTCGTCGTGCTTGTTCATGCGCTCGGGCGGGATCATCATCCATCAGAGAAGGCGACCTTCGGGGAGCACGAGTGGTACTTCTTCAGCCCGCGCGACCGCAAGTACCCCAACGGCGCGCGGCCGAACCGGGCGGCGACGTCGGGCTACTGGAAGGCCACCGGCACGGACAAGCCTATCCTGGCCTCGGCCACCGGGTGCGGCCGGGAGAAGGTCGGCGTCAAGAAGGCGCTCGTCTTCTACCGCGGGAAGCCGCCCAGGGGCCTCAAGACCAACTGGATCATGCATGAGTACCGCCTCACCGGAGCCTCTGCTGGCTCCACCACCACCAGCCGGCCGCCGCCGGTGACCGGCGGGAGCAGGGCCCCGGCCTCTCTCAGGGTACGTACTTACACGTGTCCATCGCACGGTCTATCAGTATTTATTTATTAACTACTCTCGAGCTTAATTATGGTATTGTTGATAGTTGATGAAGTTAATTATTGTACGCCGTCTCATCGATCAGTTGGACGACTGGGTGCTGTGCCGCATCTACAAGAAGACCAGCAAGGCCGCGGCCGCGGTCGGAGATGAGCAGAGGAGCATGGAGTGCGAGGACTCCGTGGAGGACGCGGTCACCGCGTACCCGCCCTACGCCACGGCGGGCATGGCCGGCGCAGGTGCGCATGGCAGCAACTACGTTCAACTGCTCCATCATCACGACAGCCACGAGGACAACTTCCAGCTAGACGGCCTGCTCACAGAACACGACGTCGGCCTCTCGGCGGGCGCCGCCTCGCTGGGCCACCTTGCCGCGGCGGCGAGGGCCACCAAACAGCTCCTCGCCCCGTCGTCCTCAACCCCGTTCAACTGGCTCGAGGCGTCAACCGG

>HS13_*NAM-1*

GCTTTTTATTATACTGTGCACAAGTATTTTTATATTCTTCCAGTAAGTACAGCGCATGTATGTGATCCTGTCGTCGTGCTTGTTCATGCGCTCGGGCGGGATCATCATCCATCAGAGAAGGCGACCTTCGGGGAGCACGAGTGGTACTTCTTCAGCCCGCGCGACCGCAAGTACCCCAACGGCGCGCGGCCGAACCGGGCGGCGACGTCGGGCTACTGGAAGGCCACCGGCACGGACAAGCCTATCCTGGCCTCGGCCACCGGGTGCGGCCGGGAGAAGGTCGGCGTCAAGAAGGCGCTCGTCTTCTACCGCGGGAAGCCGCCCAGGGGCCTCAAGACCAACTGGATCATGCATGAGTACCGCCTCACCGGAGCCTCTGCTGGCTCCACCACCACCAGCCGGCCGCCGCCGGTGACCGGCGGGAGCAGGGCCCCGGCCTCTCTCAGGGTACGTACTTACACGTGTCCATCGCACGGTCTATCAGTATTTATTTATTAACTACTCTCGAGCTTAATTATGGTATTGTTGATAGTTGATGAAGTTAATTATTGTACGCCGTCTCATCGATCAGTTGGACGACTGGGTGCTGTGCCGCATCTACAAGAAGACCAGCAAGGCCGCGGCCGCGGTCGGAGATGAGCAGAGGAGCATGGAGTGCGAGGACTCCGTGGAGGACGCGGTCACCGCGTACCCGCCCTACGCCACGGCGGGCATGGCCGGCGCAGGTGCGCATGGCAGCAACTACGTTCAACTGCTCCATCATCACGACAGCCACGAGGACAACTTCCAGCTAGACGGCCTGCTCACAGAACACGACGTCGGCCTCTCGGCGGGCGCCGCCTCGCTGGGCCACCTTGCCGCGGCGGCGAGGGCCACCAAACAGCTCCTCGCCCCGTCGTCCTCAACCCCGTTCAACTGGCTCGAGGCGTCAACCGG

>HS14_*NAM-1*

GCTTTTTATTATACTGTGCACAAGTATTTTTATATTCTTCCAGTAAGTACAGCGCATGTATGTGATCCTGTCGTCGTGCTTGTTCATGCGCTCGGGCGGGATCATCATCCATCAGAGAAGGCGACCTTCGGGGAGCACGAGTGGTACTTCTTCAGCCCGCGCGACCGCAAGTACCCCAACGGCGCGCGGCCGAACCGGGCGGCGACGTCGGGCTACTGGAAGGCCACCGGCACGGACAAGCCTATCCTGGCCTCGGCCACCGGGTGCGGCCGGGAGAAGGTCGGCGTCAAGAAGGCGCTCGTCTTCTACCGCGGGAAGCCGCCCAGGGGCCTCAAGACCAACTGGATCATGCATGAGTACCGCCTCACCGGAGCCTCTGCTGGCTCCACCACCACCAGCCGGCCGCCGCCGGTGACCGGCGGGAGCAGGGCCCCGGCCTCTCTCAGGGTACGTACTTACACGTGTCCATCGCACGGTCTATCAGTATTTATTTATTAACTACTCTCGAGCTTAATTATGGTATTGTTGATAGTTGATGAAGTTAATTATTGTACGCCGTCTCATCGATCAGTTGGACGACTGGGTGCTGTGCCGCATCTACAAGAAGACCAGCAAGGCCGCGGCCGCGGTCGGAGATGAGCAGAGGAGCATGGAGTGCGAGGACTCCGTGGAGGACGCGGTCACCGCGTACCCGCCCTACGCCACGGCGGGCATGGCCGGCGCAGGTGCGCATGGCAGCAACTACGTTCAACTGCTCCATCATCACGACAGCCACGAGGACAACTTCCAGCTAGACGGCCTGCTCACAGAACACGACGTCGGCCTCTCGGCGGGCGCCGCCTCGCTGGGCCACCTTGCCGCGGCGGCGAGGGCCACCAAACAGCTCCTCGCCCCGTCGTCCTCAACCCCGTTCAACTGGCTCGAGGCGTCAACCGG

>HS15_*NAM-1*

GCTTTTTATTATACTGTGCACAAGTATTTTTATATTCTTCCAGTAAGTACAGCGCATGTATGTGATCCTGTCGTCGTGCTTGTTCATGCGCTCGGGCGGGATCATCATCCATCAGAGAAGGCGACCTTCGGGGAGCATGAGTGGTACTTCTTCAGCCCGCGCGACCGCAAGTACCCCAACGGCGCGCGGCCGAACCGGGCGGCGACGTCGGGCTACTGGAAGGCCACCGGCACGGACAAGCCTATCCTGGCCTCGGCCACCGGGTGCGGCCGGGAGAAGGTCGGCGTCAAGAAGGCGCTCGTCTTCTACCGCGGGAAGCCGCCCAGGGGCCTCAAGACCAACTGGATCATGCATGAGTACCGCCTCACCGGAGCCTCTGCTGGCTCCACCACCACCAGCCGGCCGCCGCCGGTGACCGGCGGGAGCAGGGCCCCGGCCTCTCTCAGGGTACGTACTTACACGTGTCCATCGCACGGTCTATCAGTATTTATTTATTAACTACTCTCGAGCTTAATTATGGTATTGTTGATAGTTGATGAAGTTAATTATTGTACGCCGTCTCATCGATCAGTTGGACGACTGGGTGCTGTGCCGCATCTACAAGAAGACCAGCAAGGCCGCGGCCGCGGTCGGAGATGAGCAGAGGAGCATGGAGTGCGAGGACTCCGTGGAGGACGCGGTCACCGCGTACCCGCCCTACGCCACGGCGGGCATGGCCGGCGCAGGTGCGCATGGCAGCAACTACGTTCAACTGCTCCATCATCACGACAGCCACGAGGACAACTTCCAGCTAGACGGCCTGCTCACAGAACACGACGTCGGCCTCTCGGCGGGCGCCGCCTCGCTGGGCCACCTTGCCGCGGCGGCGAGGGCCACCAAACAGTTCCTCGCCCCGTCGTCCTCAACCCCGTTCAACTGGCTCGAGGCGTCAACCGG

>HS18_*NAM-1*

GCTTTTTATTATACTGTGCACAAGTATTTTTATATTCTTCCAGTAAGTACAGCGCATGTATGTGATCCTGTCGTCGTGCTTGTTCATGCGCTCGGGCGGGATCATCATCCATCAGAGAAGGCGACCTTCGGGGAGCACGAGTGGTACTTCTTCAGCCCGCGCGACCGCAAGTACCCCAACGGCGCGCGGCCGAACCGGGCGGCGACGTCGGGCTACTGGAAGGCCACCGGCACGGACAAGCCTATCCTGGCCTCGGCCACCGGGTGCGGCCGGGAGAAGGTCGGCGTCAAGAAGGCGCTCGTCTTCTACCGCGGGAAGCCGCCCAGGGGCCTCAAGACCAACTGGATCATGCATGAGTACCGCCTCACCGGAGCCTCTGCTGGCTCCACCACCACCAGCCGGCCGCCGCCGGTGACCGGCGGGAGCAGGGCCCCGGCCTCTCTCAGGGTACGTACTTACACGTGTCCATCGCACGGTCTATCAGTATTTATTTATTAACTACTCTCGAGCTTAATTATGGTATTGTTGATAGTTGATGAAGTTAATTATTGTACGCCGTCTCATCGATCAGTTGGACGACTGGGTGCTGTGCCGCATCTACAAGAAGACCAGCAAGGCCGCGGCCGCGGTCGGAGATGAGCAGAGGAGCATGGAGTGCGAGGACTCCGTGGAGGACGCGGTCACCGCGTACCCGCCCTACGCCACGGCGGGCATGGCCGGCGCAGGTGCGCATGGCAGCAACTACGTTCAACTGCTCCATCATCACGACAGCCACGAGGACAACTTCCAGCTAGACGGCCTGCTCACAGAACACGACGTCGGCCTCTCGGCGGGCGCCGCCTCGCTGGGCCACCTTGCCGCGGCGGCGAGGGCCACCAAACAGTTCCTCGCCCCGTCGTCCTCAACCCCGTTCAACTGGCTCGAGGCGTCAACCGG

>HS19_*NAM-1*

GCTTTTTATTATACTGTGCACAAGTATTTTTATATTCTTCCAGTAAGTACAGCGCATGTATGTGATCCTGTCGTCGTGCTTGTTCATGCGCTCGGGCGGGATCATCATCCATCAGAGAAGGCGACCTTCGGGGAGCACGAGTGGTACTTCTTCAGCCCGCGCGACCGCAAGTACCCCAACGGCGCGCGGCCGAACCGGGCGGCGACGTCGGGCTACTGGAAGGCCACCGGCACGGACAAGCCTATCCTGGCCTCGGCCACCGGGTGCGGCCGGGAGAAGGTCGGCGTCAAGAAGGCGCTCGTCTTCTACCGCGGGAAGCCGCCCAGGGGCCTCAAGACCAACTGGATCATGCATGAGTACCGCCTCACCGGAGCCTCTGCTGGCTCCACCACCACCAGCCGGCCGCCGCCGGTGACCGGCGGGAGCAGGGCCCCGGCCTCTCTCAGGGTACGTACTTACACGTGTCCATCGCACGGTCTATCAGTATTTATTTATTAACTACTCTCGAGCTTAATTATGGTATTGTTGATAGTTGATGAAGTTAATTATTGTACGCCGTCTCATCGATCAGTTGGACGACTGGGTGCTGTGCCGCATCTACAAGAAGACCAGCAAGGCCGCGGCCGCGGTCGGAGATGAGCAGAGGAGCATGGAGTGCGAGGACTCCGTGGAGGACGCGGTCACCGCGTACCCGCCCTACGCCACGGCGGGCATGGCCGGCGCAGGTGCGCATGGCAGCAACTACGTTCAACTGCTCCATCATCACGACAGCCACGAGGACAACTTCCAGCTAGACGGCCTGCTCACAGAACACGACGTCGGCCTCTCGGCGGGCGCCGCCTCGCTGGGCCACCTTGCCGCGGCGGCGAGGGCCACCAAACAGTTCCTCGCCCCGTCGTCCTCAACCCCGTTCAACTGGCTCGAGGCGTCAACCGG

>HS20_*NAM-1*

GCTTTTTATTATACTGTGCACAAGTATTTTTATATTCTTCCAGTAAGTACAGCGCATGTATGTGATCCTGTCGTCGTGCTTGTTCATGCGCTCGGGCGGGATCATCATCCATCAGAGAAGGCGACCTTCGGGGAGCACGAGTGGTACTTCTTCAGCCCGCGCGACCGCAAGTACCCCAACGGCGCGCGGCCGAACCGGGCGGCGACGTCGGGCTACTGGAAGGCCACCGGCACGGACAAGCCTATCCTGGCCTCGGCCACCGGGTGCGGCCGGGAGAAGGTCGGCGTCAAGAAGGCGCTCGTCTTCTACCGCGGGAAGCCGCCCAGGGGCCTCAAGACCAACTGGATCATGCATGAGTACCGCCTCACCGGAGCCTCTGCTGGCTCCACCACCACCAGCCGGCCGCCGCCGGTGACCGGCGGGAGCAGGGCCCCGGCCTCTCTCAGGGTACGTACTTACACGTGTCCATCGCACGGTCTATCAGTATTTATTTATTAACTACTCTCGAGCTTAATTATGGTATTGTTGATAGTTGATGAAGTTAATTATTGTACGCCGTCTCATCGATCAGTTGGACGACTGGGTGCTGTGCCGCATCTACAAGAAGACCAGCAAGGCCGCGGCCGCGGTCGGAGATGAGCAGAGGAGCATGGAGTGCGAGGACTCCGTGGAGGACGCGGTCACCGCGTACCCGCCCTACGCCACGGCGGGCATGGCCGGCGCAGGTGCGCATGGCAGCAACTACGTTCAACTGCTCCATCATCACGACAGCCACGAGGACAACTTCCAGCTAGACGGCCTGCTCACAGAACACGACGTCGGCCTCTCGGCGGGCGCCGCCTCGCTGGGCCACCTTGCCGCGGCGGCGAGGGCCACCAAACAGTTCCTCGCCCCGTCGTCCTCAACCCCGTTCAACTGGCTCGAGGCGTCAACCGG

>HS21_*NAM-1*

GCTTTTTATTATACTGTGCACAAGTATTTTTATATTCTTCCAGTAAGTACAGCGCATGTATGTGATCCTGTCGTCGTGCTTGTTCATGCGCTCGGGCGGGATCATCATCCATCAGAGAAGGCGACCTTCGGGGAGCACGAGTGGTACTTCTTCAGCCCGCGCGACCGCAAGTACCCCAACGGCGCGCGGCCGAACCGGGCGGCGACGTCGGGCTACTGGAAGGCCACCGGCACGGACAAGCCTATCCTGGCCTCGGCCACCGGGTGCGGCCGGGAGAAGGTCGGCGTCAAGAAGGCGCTCGTCTTCTACCGCGGGAAGCCGCCCAGGGGCCTCAAGACCAACTGGATCATGCATGAGTACCGCCTCACCGGAGCCTCTGCTGGCTCCACCACCACCAGCCGGCCGCCGCCGGTGACCGGCGGGAGCAGGGCCCCGGCCTCTCTCAGGGTACGTCCTTACACGTGTCCATCGCACGGTCTATCAGTATTTATTTATTAACTACTCTCGAGCTTAATTATGGTATTGTTGATAGTTGATGAAGTTAATTATTGTACGCCGTCTCATCGATCAGTTGGACGACTGGGTGCTGTGCCGCATCTACAAGAAGACCAGCAAGGCCGCGGCCGCGGTCGGAGATGAGCAGAGGAGCATGGAGTGCGAGGACTCCGTGGAGGACGCGGTCACCGCGTACCCGCCCTACGCCACGGCGGGCATGGCCGGCGCAGGTGCGCATGGCAGCAACTACGTTCAACTGCTCCATCATCACGACAGCCACGAGGACAACTTCCAGCTAGACGGCCTGCTCACAGAACACGACGTCGGCCTCTCGGCGGGCGCCGCCTCGCTGGGCCACCTTGCCGCGGCGGCGAGGGCCACCAAACAGTTCCTCGCCCCGTCGTCCTCAACCCCGTTCAACTGGCTCGAGGCGTCAACCGG

>HS22_*NAM-1*

GCTTTTTATTATACTGTGCACAAGTATTTTTATATTCTTCCAGTAAGTACAGCGCATGTATGTGATCCTGTCGTCGTGCTTGTTCATGCGCTCGGGCGGGATCATCATCCATCAGAGAAGGCGACCTTCGGGGAGCACGAGTGGTACTTCTTCAGCCCGCGCGACCGCAAGTACCCCAACGGCGCGCGGCCGAACCGGGCGGCGACGTCGGGCTACTGGAAGGCCACCGGCACGGACAAGCCTATCCTGGCCTCGGCCACCGGGTGCGGCCGGGAGAAGGTCGGCGTCAAGAAGGCGCTCGTCTTCTACCGCGGGAAGCCGCCCAGGGGCCTCAAGACCAACTGGATCATGCATGAGTACCGCCTCACCGGAGCCTCTGCTGGCTCCACCACCACCAGCCGGCCGCCGCCGGTGACCGGCGGGAGCAGGGCCCCGGCCTCTCTCAGGGTACGTCCTTACACGTGTCCATCGCACGGTCTATCAGTATTTATTTATTAACTACTCTCGAGCTTAATTATGGTATTGTTGATAGTTGATGAAGTTAATTATTGTACGCCGTCTCATCGATCAGTTGGACGACTGGGTGCTGTGCCGCATCTACAAGAAGACCAGCAAGGCCGCGGCCGCGGTCGGAGATGAGCAGAGGAGCATGGAGTGCGAGGACTCCGTGGAGGACGCGGTCACCGCGTACCCGCCCTACGCCACGGCGGGCATGGCCGGCGCAGGTGCGCATGGCAGCAACTACGTTCAACTGCTCCATCATCACGACAGCCACGAGGACAACTTCCAGCTAGACGGCCTGCTCACAGAACACGACGTCGGCCTCTCGGCGGGCGCCGCCTCGCTGGGCCACCTTGCCGCGGCGGCGAGGGCCACCAAACAGTTCCTCGCCCCGTCGTCCTCAACCCCGTTCAACTGGCTCGAGGCGTCAACCGG

>HS23_*NAM-1*

GCTTTTTATTATACTGTGCACAAGTATTTTTATATTCTTCCAGTAAGTACAGCGCATGTATGTGATCCTGTCGTCGTGCTTGTTCATGCGCTCGGGCGGGATCATCATCCATCAGAGAAGGCGACCTTCGGGGAGCACGAGTGGTACTTCTTCAGCCCGCGCGACCGCAAGTACCCCAACGGCGCGCGGCCGAACCGGGCGGCGACGTCGGGCTACTGGAAGGCCACCGGCACGGACAAGCCTATCCTGGCCTCGGCCACCGGGTGCGGCCGGGAGAAGGTCGGCGTCAAGAAGGCGCTCGTCTTCTACCGCGGGAAGCCGCCCAGGGGCCTCAAGACCAACTGGATCATGCATGAGTACCGCCTCACCGGAGCCTCTGCTGGCTCCACCACCACCAGCCGGCCGCCGCCGGTGACCGGCGGGAGCAGGGCCCCGGCCTCTCTCAGGGTACGTACTTACACGTGTCCATCGCACGGTCTATCAGTATTTATTTATTAACTACTCTCGAGCTTAATTATGGTATTGTTGATAGTTGATGAAGTTAATTATTGTACGCCGTCTCATCGATCAGTTGGACGACTGGGTGCTGTGCCGCATCTACAAGAAGACCAGCAAGGCCGCGGCCGCGGTCGGAGATGAGCAGAGGAGCATGGAGTGCGAGGACTCCGTGGAGGACGCGGTCACCGCGTACCCGCCCTACGCCACGGCGGGCATGGCCGGCGCAGGTGCGCATGGCAGCAACTACGTTCAACTGCTCCATCATCACGACAGCCACGAGGACAACTTCCAGCTAGACGGCCTGCTCACAGAACACGACGTCGGCCTCTCGGCGGGCGCCGCCTCGCTGGGCCACCTTGCCGCGGCGGCGAGGGCCACCAAACAGTTCCTCGCCCCGTCGTCCTCAACCCCGTTCAACTGGCTCGAGGCGTCAACCGG

>HS24_*NAM-1*

GCTTTTTATTATACTGTGCACAAGTATTTTTATATTCTTCCAGTAAGTACAGCGCATGTATGTGATCCTGTCGTCGTGCTTGTTCATGCGCTCGGGCGGGATCATCATCCATCAGAGAAGGCGACCTTCGGGGAGCACGAGTGGTACTTCTTCAGCCCGCGCGACCGCAAGTACCCCAACGGCGCGCGGCCGAACCGGGCGGCGACGTCGGGCTACTGGAAGGCCACCGGCACGGACAAGCCTATCCTGGCCTCGGCCACCGGGTGCGGCCGGGAGAAGGTCGGCGTCAAGAAGGCGCTCGTCTTCTACCGCGGGAAGCCGCCCAGGGGCCTCAAGACCAACTGGATCATGCATGAGTACCGCCTCACCGGAGCCTCTGCTGGCTCCACCACCACCAGCCGGCCGCCGCCGGTGACCGGCGGGAGCAGGGCCCCGGCCTCTCTCAGGGTACGTACTTACACGTGTCCATCGCACGGTCTATCAGTATTTATTTATTAACTACTCTCGAGCTTAATTATGGTATTGTTGATAGTTGATGAAGTTAATTATTGTACGCCGTCTCATCGATCAGTTGGACGACTGGGTGCTGTGCCGCATCTACAAGAAGACCAGCAAGGCCGCGGCCGCGGTCGGAGATGAGCAGAGGAGCATGGAGTGCGAGGACTCCGTGGAGGACGCGGTCACCGCGTACCCGCCCTACGCCACGGCGGGCATGGCCGGCGCAGGTGCGCATGGCAGCAACTACGTTCAACTGCTCCATCATCACGACAGCCACGAGGACAACTTCCAGCTAGACGGCCTGCTCACAGAACACGACGTCAGCCTCTCGGCGGGCGCCGCCTCGCTGGGCCACCTTGCCGCGGCGGCGAGGGCCACCAAACAGTTCCTCGCCCCGTCGTCCTCAACCCCGTTCAACTGGCTCGAGGCGTCAACCGG

>HS25_*NAM-1*

GCTTTTTATTATACTGTGCACAAGTATTTTTATATTCTTCCAGTAAGTACAGCGCATGTATGTGATCCTGTCGTCGTGCTTGTTCATGCGCTCGGGCGGGATCATCATCCATCAGAGAAGGCGACCTTCGGGGAGCACGAGTGGTACTTCTTCAGCCCGCGCGACCGCAAGTACCCCAACGGCGCGCGGCCGAACCGGGCGGCGACGTCGGGCTACTGGAAGGCCACCGGCACGGACAAGCCTATCCTGGCCTCGGCCACCGGGTGCGGCCGGGAGAAGGTCGGCGTCAAGAAGGCGCTCGTCTTCTACCGCGGGAAGCCGCCCAGGGGCCTCAAGACCAACTGGATCATGCATGAGTACCGCCTCACCGGAGCCTCTGCTGGCTCCACCACCACCAGCCGGCCGCCGCCGGTGACCGGCGGGAGCAGGGCCCCGGCCTCTCTCAGGGTACGTACTTACACGTGTCCATCGCACGGTCTATCAGTATTTATTTATTAACTACTCTCGAGCTTAATTATGGTATTGTTGATAGTTGATGAAGTTAATTATTGTACGCCGTCTCATCGATCAGTTGGACGACTGGGTGCTGTGCCGCATCTACAAGAAGACCAGCAAGGCCGCGGCCGCGGTCGGAGATGAGCAGAGGAGCATGGAGTGCGAGGACTCCGTGGAGGACGCGGTCACCGCGTACCCGCCCTACGCCACGGCGGGCATGGCCGGCGCAGGTGCGCATGGCAGCAACTACGTTCAACTGCTCCATCATCACGACAGCCACGAGGACAACTTCCAGCTAGACGGCCTGCTCACAGAACACGACGTCAGCCTCTCGGCGGGCGCCGCCTCGCTGGGCCACCTTGCCGCGGCGGCGAGGGCCACCAAACAGTTCCTCGCCCCGTCGTCCTCAACCCCGTTCAACTGGCTCGAGGCGTCAACCGG

>HS26_*NAM-1*

GCTTTTTATTATACTGTGCACAAGTATTTTTATATTCTTCCAGTAAGTACAGCGCATGTATGTGATCCTGTCGTCGTGCTTGTTCATGCGCTCGGGCGGGATCATCATCCATCAGAGAAGGCGACCTTCGGGGAGCACGAGTGGTACTTCTTCAGCCCGCGCGACCGCAAGTACCCCAACGGCGCGCGGCCGAACCGGGCGGCGACGTCGGGCTACTGGAAGGCCACCGGCACGGACAAGCCTATCCTGGCCTCGGCCACCGGGTGCGGCCGGGAGAAGGTCGGCGTCAAGAAGGCGCTCGTCTTCTACCGCGGGAAGCCGCCCAGGGGCCTCAAGACCAACTGGATCATGCATGAGTACCGCCTCACCGGAGCCTCTGCTGGCTCCACCACCACCAGCCGGCCGCCGCCGGTGACCGGCGGGAGCAGGGCCCCGGCCTCTCTCAGGGTACGTACTTACACGTGTCCATCGCACGGTCTATCAGTATTTATTTATTAACTACTCTCGAGCTTAATTATGGTATTGTTGATAGTTGATGAAGTTAATTATTGTACGCCGTCTCATCGATCAGTTGGACGACTGGGTGCTGTGCCGCATCTACAAGAAGACCAGCAAGGCCGCGGCCGCGGTCGGAGATGAGCAGAGGAGCATGGAGTGCGAGGACTCCGTGGAGGACGCGGTCACCGCGTACCCGCCCTACGCCACGGCGGGCATGGCCGGCGCAGGTGCGCATGGCAGCAACTACGTTCAACTGCTCCATCATCACGACAGCCACGAGGACAACTTCCAGCTAGACGGCCTGCTCACAGAACACGACGTCAGCCTCTCGGCGGGCGCCGCCTCGCTGGGCCACCTTGCCGCGGCGGCGAGGGCCACCAAACAGTTCCTCGCCCCGTCGTCCTCAACCCCGTTCAACTGGCTCGAGGCGTCAACCGG

>HS27_*NAM-1*

GCTTTTTATTATACTGTGCACAAGTATTTTTATATTCTTCCAGTAAGTACAGCGCATGTATGTGATCCTGTCGTCGTGCTTGTTCATGCGCTCGGGCGGGATCATCATCCATCAGAGAAGGCGACCTTCGGGGAGCATGAGTGGTACTTCTTCAGCCCGCGCGACCGCAAGTACCCCAACGGCGCGCGGCCGAACCGGGCGGCGACGTCGGGCTACTGGAAGGCCACCGGCACGGACAAGCCTATCCTGGCCTCGGCCACCGGGTGCGGCCGGGAGAAGGTCGGCGTCAAGAAGGCGCTCGTCTTCTACCGCGGGAAGCCGCCCAGGGGCCTCAAGACCAACTGGATCATGCATGAGTACCGCCTCACCGGAGCCTCTGCTGGCTCCACCACCACCAGCCGGCCGCCGCCGGTGACCGGCGGGAGCAGGGCCCCGGCCTCTCTCAGGGTACGTACTTACACGTGTCCATCGCACGGTCTATCAGTATTTATTTATTAACTACTCTCGAGCTTAATTATGGTATTGTTGATAGTTGATGAAGTTAATTATTGTACGCCGTCTCATCGATCAGTTGGACGACTGGGTGCTGTGCCGCATCTACAAGAAGACCAGCAAGGCCGCGGCCGCGGTCGGAGATGAGCAGAGGAGCATGGAGTGCGAGGACTCCGTGGAGGACGCGGTCACCGCGTACCCGCCCTACGCCACGGCGGGCATGGCCGGCGCAGGTGCGCATGGCAGCAACTACGTTCAACTGCTCCATCATCACGACAGCCACGAGGACAACTTCCAGCTAGACGGCCTGCTCACAGAACACGACGTCGGCCTCTCGGCGGGCGCCGCCTCGCTGGGCCACCTTGCCGCGGCGGCGAGGGCCACCAAACAGTTCCTCGCCCCGTCGTCCTCAACCCCGTTCAACTGGCTCGAGGCGTCAACCGG

>HS28_*NAM-1*

GCTTTTTATTATACTGTGCACAAGTATTTTTATATTCTTCCAGTAAGTACAGCGCATGTATGTGATCCTGTCGTCGTGCTTGTTCATGCGCTCGGGCGGGATCGTCATCCATCAGAGAAGGCGACCTTCGGGGAGCACGAGTGGTACTTCTTCAGCCCGCGCGACCGCAAGTACCCCAACGGCGCGCGGCCGAACCGGGCGGCGACGTCGGGCTACTGGAAGGCCACCGGCACGGACAAGCCTATCCTGGCCTCGGCCACCGGGTGCGGCCGGGAGAAGGTCGGCGTCAAGAAGGCGCTCGTCTTCTACCGCGGGAAGCCGCCCAGGGGCCTCAAGACCAACTGGATCATGCATGAGTACCGCCTCACCGGAGCCTCTGCTGGCTCCACCACCACCAGCCGGCCGCCGCCGGTGACCGGCGGGAGCAGGGCCCCGGCCTCTCTCAGGGTACGTACTTACACGTGTCCATCGCACGGTCTATCAGTATTTATTTATTAACTACTCTCGAGCTTAATTATGGTATTGTTGATAGTTGATGAAGTTAATTATTGTACGCCGTCTCATCGATCAGTTGGACGACTGGGTGCTGTGCCGCATCTACAAGAAGACCAGCAAGGCCGCGGCCGCGGTCGGAGATGAGCAGAGGAGCATGGAGTGCGAGGACTCCGTGGAGGACGCGGTCACCGCGTACCCGCCCTACGCCACGGCGGGCATGGCCGGCGCAGGTGCGCATGGCAGCAACTACGTTCAACTGCTCCATCATCACGACAGCCACGAGGACAACTTCCAGCTAGACGGCCTGCTCACAGAACACGACGTCGGCCTCTCGGCGGGCGCCGCCTCGCTGGGCCACCTTGCCGCGGCGGCGAGGGCCACCAAACAGTTCCTCGCCCCGTCGTCCTCAACCCCGTTCAACTGGCTCGAGGCGTCAACCGG

>HS29_*NAM-1*

GCTTTTTATTATACTGTGCACAAGTATTTTTATATTCTTCCAGTAAGTACAGCGCATGTATGTGATCCTGTCGTCGTGCTTGTTCATGCGCTCGGGCGGGATCATCATCCATCAGAGAAGGCGACCTTCGGGGAGCACGAGTGGTACTTCTTCAGCCCGCGCGACCGCAAGTACCCCAACGGCGCGCGGCCGAACCGGGCGGCGACGTCGGGCTACTGGAAGGCCACCGGCACGGACAAGCCTATCCTGGCCTCGGCCACCGGGTGCGGCCGGGAGAAGGTCGGCGTCAAGAAGGCGCTCGTCTTCTACCGCGGGAAGCCGCCCAGGGGCCTCAAGACCAACTGGATCATGCATGAGTACCGCCTCACCGGAGCCTCTGCTGGCTCCACCACCACCAGCCGGCCGCCGCCGGTGACCGGCGGGAGCAGGGCCCCGGCCTCTCTCAGGGTACGTACTTACACGTGTCCATCGCACGGTCTATCAGTATTTATTTATTAACTACTCTCGAGCTTAATTATGGTATTGTTGATAGTTGATGAAGTTAATTATTGTACGCCGTCTCATCGATCAGTTGGACGACTGGGTGCTGTGCCGCATCTACAAGAAGACCAGCAAGGCCGCGGCCGCGGTCGGAGATGAGCAGAGGAGCATGGAGTGCGAGGACTCCGTGGAGGACGCGGTCACCGCGTACCCGCCCTACGCCACGGCGGGCATGGCCGGCGCAGGTGCGCATGGCAGCAACTACGTTCAACTGCTCCATCATCACGACAGCCACGAGGACAACTTCCAGCTAGACGGCCTGCTCACAGAACACGACGTCGGCCTCTCGGCGGGCGCCGCCTCGCTGGGCCACCTTGCCGCGGCGGCGAGGGCCACCAAACAGTTCCTCGCCCCGTCGTCCTCAACCCCGTTCAACTGGCTCGAGGCGTCAACCGG

>HS30_*NAM-1*

GCTTTTTATTATACTGTGCACAAGTATTTTTATATTCTTCCAGTAAGTACAGCGCATGTATGTGATCCTGTCGTCGTGCTTGTTCATGCGCTCGGGCGGGATCATCATCCATCAGAGAAGGCGACCTTCGGGGAGCACGAGTGGTACTTCTTCAGCCCGCGCGACCGCAAGTACCCCAACGGCGCGCGGCCGAACCGGGCGGCGACGTCGGGCTACTGGAAGGCCACCGGCACGGACAAGCCTATCCTGGCCTCGGCCACCGGGTGCGGCCGGGAGAAGGTCGGCGTCAAGAAGGCGCTCGTCTTCTACCGCGGGAAGCCGCCCAGGGGCCTCAAGACCAACTGGATCATGCATGAGTACCGCCTCACCGGAGCCTCTGCTGGCTCCACCACCACCAGCCGGCCGCCGCCGGTGACCGGCGGGAGCAGGGCCCCGGCCTCTCTCAGGGTACGTACTTACACGTGTCCATCGCACGGTCTATCAGTATTTATTTATTAACTACTCTCGAGCTTAATTATGGTATTGTTGATAGTTGATGAAGTTAATTATTGTACGCCGTCTCATCGATCAGTTGGACGACTGGGTGCTGTGCCGCATCTACAAGAAGACCAGCAAGGCCGCGGCCGCGGTCGGAGATGAGCAGAGGAGCATGGAGTGCGAGGACTCCGTGGAGGACGCGGTCACCGCGTACCCGCCCTACGCCACGGCGGGCATGGCCGGCGCAGGTGCGCATGGCAGCAACTACGTTCAACTGCTCCATCATCACGACAGCCACGAGGACAACTTCCAGCTAGACGGCCTGCTCACAGAACACGACGTCGGCCTCTCGGCGGGCGCCGCCTCGCTGGGCCACCTTGCCGCGGCGGCGAGGGCCACCAAACAGTTCCTCGCCCCGTCGTCCTCAACCCCGTTCAACTGGCTCGAGGCGTCAACCGG

>HS31_*NAM-1*

GCTTTTTATTATACTGTGCACAAGTATTTTTATATTCTTCCAGTAAGTACAGCGCATGTATGTGATCCTGTCGTCGTGCTTGTTCATGCGCTCGGGCGGGATCATCATCCATCAGAGAAGGCGACCTTCGGGGAGCATGAGTGGTACTTCTTCAGCCCGCGCGACCGCAAGTACCCCAACGGCGCGCGGCCGAACCGGGCGGCGACGTCGGGCTACTGGAAGGCCACCGGCACGGACAAGCCTATCCTGGCCTCGGCCACCGGGTGCGGCCGGGAGAAGGTCGGCGTCAAGAAGGCGCTCGTCTTCTACCGCGGGAAGCCGCCCAGGGGCCTCAAGACCAACTGGATCATGCATGAGTACCGCCTCACCGGAGCCTCTGCTGGCTCCACCACCACCAGCCGGCCGCCGCCGGTGACCGGCGGGAGCAGGGCCCCGGCCTCTCTCAGGGTACGTACTTACACGTGTCCATCGCACGGTCTATCAGTATTTATTTATTAACTACTCTCGAGCTTAATTATGGTATTGTTGATAGTTGATGAAGTTAATTATTGTACGCCGTCTCATCGATCAGTTGGACGACTGGGTGCTGTGCCGCATCTACAAGAAGACCAGCAAGGCCGCGGCCGCGGTCGGAGATGAGCAGAGGAGCATGGAGTGCGAGGACTCCGTGGAGGACGCGGTCACCGCGTACCCGCCCTACGCCACGGCGGGCATGGCCGGCGCAGGTGCGCATGGCAGCAACTACGTTCAACTGCTCCATCATCACGACAGCCACGAGGACAACTTCCAGCTAGACGGCCTGCTCACAGAACACGACGTCGGCCTCTCGGCGGGCGCCGCCTCGCTGGGCCACCTTGCCGCGGCGGCGAGGGCCACCAAACAGTTCCTCGCCCCGTCGTCCTCAACCCCGTTCAACTGGCTCGAGGCGTCAACCGG

>HS32_*NAM-1*

GCTTTTTATTATACTGTGCACAAGTATTTTTATATTCTTCCAGTAAGTACAGCGCATGTATGTGATCCTGTCGTCGTGCTTGTTCATGCGCTCGGGCGGGATCATCATCCATCAGAGAAGGCGACCTTCGGGGAGCACGAGTGGTACTTCTTCAGCCCGCGCGACCGCAAGTACCCCAACGGCGCGCGGCCGAACCGGGCGGCGACGTCGGGCTACTGGAAGGCCACCGGCACGGACAAGCCTATCCTGGCCTCGGCCACCGGGTGCGGCCGGGAGAAGGTCGGCGTCAAGAAGGCGCTCGTCTTCTACCGCGGGAAGCCGCCCAGGGGCCTCAAGACCAACTGGATCATGCATGAGTACCGCCTCACCGGAGCCTCTGCTGGCTCCACCACCACCAGCCGGCCGCCGCCGGTGACCGGCGGGAGCAGGGCCCCGGCCTCTCTCAGGGTACGTACTTACACGTGTCCATCGCACGGTCTATCAGTATTTATTTATTAACTACTCTCGAGCTTAATTATGGTATTGTTGATAGTTGATGAAGTTAATTATTGTACGCCGTCTCATCGATCAGTTGGACGACTGGGTGCTGTGCCGCATCTACAAGAAGACCAGCAAGGCCGCGGCCGCGGTCGGAGATGAGCAGAGGAGCATGGAGTGCGAGGACTCCGTGGAGGACGCGGTCACCGCGTACCCGCCCTACGCCACGGCGGGCATGGCCGGCGCAGGTGCGCATGGCAGCAACTACGTTCAACTGCTCCATCATCACGACAGCCACGAGGACAACTTCCAGCTAGACGGCCTGCTCACAGAACACGACGTCGGCCTCTCGGCGGGCGCCGCCTCGCTGGGCCACCTTGCCGCGGCGGCGAGGGCCACCAAACAGTTCCTCGCCCCGTCGTCCTCAACCCCGTTCAACTGGCTCGAGGCGTCAACCGG

>HS33_*NAM-1*

GCTTTTTATTATACTGTGCACAAGTATTTTTATATTCTTCCAGTAAGTACAGCGCATGTATGTGATCCTGTCGTCGTGCTTGTTCATGCGCTCGGGCGGGATCATCATCCATCAGAGAAGGCGACCTTCGGGGAGCATGAGTGGTACTTCTTCAGCCCGCGCGACCGCAAGTACCCCAACGGCGCGCGGCCGAACCGGGCGGCGACGTCGGGCTACTGGAAGGCCACCGGCACGGACAAGCCTATCCTGGCCTCGGCCACCGGGTGCGGCCGGGAGAAGGTCGGCGTCAAGAAGGCGCTCGTCTTCTACCGCGGGAAGCCGCCCAGGGGCCTCAAGACCAACTGGATCATGCATGAGTACCGCCTCACCGGAGCCTCTGCTGGCTCCACCACCACCAGCCGGCCGCCGCCGGTGACCGGCGGGAGCAGGGCCCCGGCCTCTCTCAGGGTACGTACTTACACGTGTCCATCGCACGGTCTATCAGTATTTATTTATTAACTACTCTCGAGCTTAATTATGGTATTGTTGATAGTTGATGAAGTTAATTATTGTACGCCGTCTCATCGATCAGTTGGACGACTGGGTGCTGTGCCGCATCTACAAGAAGACCAGCAAGGCCGCGGCCGCGGTCGGAGATGAGCAGAGGAGCATGGAGTGCGAGGACTCCGTGGAGGACGCGGTCACCGCGTACCCGCCCTACGCCACGGCGGGCATGGCCGGCGCAGGTGCGCATGGCAGCAACTACGTTCAACTGCTCCATCATCACGACAGCCACGAGGACAACTTCCAGCTAGACGGCCTGCTCACAGAACACGACGTCGGCCTCTCGGCGGGCGCCGCCTCGCTGGGCCACCTTGCCGCGGCGGCGAGGGCCACCAAACAGTTCCTCGCCCCGTCGTCCTCAACCCCGTTCAACTGGCTCGAGGCGTCAACCGG

>HS34_*NAM-1*

GCTTTTTATTATACTGTGCACAAGTATTTTTATATTCTTCCAGTAAGTACAGCGCATGTATGTGATCCTGTCGTCGTGCTTGTTCATGCGCTCGGGCGGGATCGTCATCCATCAGAGAAGGCGACCTTCGGGGAGCACGAGTGGTACTTCTTCAGCCCGCGCGACCGCAAGTACCCCAACGGCGCGCGGCCGAACCGGGCGGCGACGTCGGGCTACTGGAAGGCCACCGGCACGGACAAGCCTATCCTGGCCTCGGCCACCGGGTGCGGCCGGGAGAAGGTCGGCGTCAAGAAGGCGCTCGTCTTCTACCGCGGGAAGCCGCCCAGGGGCCTCAAGACCAACTGGATCATGCATGAGTACCGCCTCACCGGAGCCTCTGCTGGCTCCACCACCACCAGCCGGCCGCCGCCGGTGACCGGCGGGAGCAGGGCCCCGGCCTCTCTCAGGGTACGTACTTACACGTGTCCATCGCACGGTCTATCAGTATTTATTTATTAACTACTCTCGAGCTTAATTATGGTATTGTTGATAGTTGATGAAGTTAATTATTGTACGCCGTCTCATCGATCAGTTGGACGACTGGGTGCTGTGCCGCATCTACAAGAAGACCAGCAAGGCCGCGGCCGCGGTCGGAGATGAGCAGAGGAGCATGGAGTGCGAGGACTCCGTGGAGGACGCGGTCACCGCGTACCCGCCCTACGCCACGGCGGGCATGGCCGGCGCAGGTGCGCATGGCAGCAACTACGTTCAACTGCTCCATCATCACGACAGCCACGAGGACAACTTCCAGCTAGACGGCCTGCTCACAGAACACGACGTCGGCCTCTCGGCGGGCGCCGCCTCGCTGGGCCACCTTGCCGCGGCGGCGAGGGCCACCAAACAGTTCCTCGCCCCGTCGTCCTCAACCCCGTTCAACTGGCTCGAGGCGTCAACCGG

>HS35_*NAM-1*

GCTTTTTATTATACTGTGCACAAGTATTTTTATATTCTTCCAGTAAGTACAGCGCATGTATGTGATCCTGTCGTCGTGCTTGTTCATGCGCTCGGGCGGGATCGTCATCCATCAGAGAAGGCGACCTTCGGGGAGCACGAGTGGTACTTCTTCAGCCCGCGCGACCGCAAGTACCCCAACGGCGCGCGGCCGAACCGGGCGGCGACGTCGGGCTACTGGAAGGCCACCGGCACGGACAAGCCTATCCTGGCCTCGGCCACCGGGTGCGGCCGGGAGAAGGTCGGCGTCAAGAAGGCGCTCGTCTTCTACCGCGGGAAGCCGCCCAGGGGCCTCAAGACCAACTGGATCATGCATGAGTACCGCCTCACCGGAGCCTCTGCTGGCTCCACCACCACCAGCCGGCCGCCGCCGGTGACCGGCGGGAGCAGGGCCCCGGCCTCTCTCAGGGTACGTACTTACACGTGTCCATCGCACGGTCTATCAGTATTTATTTATTAACTACTCTCGAGCTTAATTATGGTATTGTTGATAGTTGATGAAGTTAATTATTGTACGCCGTCTCATCGATCAGTTGGACGACTGGGTGCTGTGCCGCATCTACAAGAAGACCAGCAAGGCCGCGGCCGCGGTCGGAGATGAGCAGAGGAGCATGGAGTGCGAGGACTCCGTGGAGGACGCGGTCACCGCGTACCCGCCCTACGCCACGGCGGGCATGGCCGGCGCAGGTGCGCATGGCAGCAACTACGTTCAACTGCTCCATCATCACGACAGCCACGAGGACAACTTCCAGCTAGACGGCCTGCTCACAGAACACGACGTCGGCCTCTCGGCGGGCGCCGCCTCGCTGGGCCACCTTGCCGCGGCGGCGAGGGCCACCAAACAGTTCCTCGCCCCGTCGTCCTCAACCCCGTTCAACTGGCTCGAGGCGTCAACCGG

>HS36_*NAM-1*

GCTTTTTATTATACTGTGCACAAGTATTTTTATATTCTTCCAGTAAGTACAGCGCATGTATGTGATCCTGTCGTCGTGCTTGTTCATGCGCTCGGGCGGGATCGTCATCCATCAGAGAAGGCGACCTTCGGGGAGCACGAGTGGTACTTCTTCAGCCCGCGCGACCGCAAGTACCCCAACGGCGCGCGGCCGAACCGGGCGGCGACGTCGGGCTACTGGAAGGCCACCGGCACGGACAAGCCTATCCTGGCCTCGGCCACCGGGTGCGGCCGGGAGAAGGTCGGCGTCAAGAAGGCGCTCGTCTTCTACCGCGGGAAGCCGCCCAGGGGCCTCAAGACCAACTGGATCATGCATGAGTACCGCCTCACCGGAGCCTCTGCTGGCTCCACCACCACCAGCCGGCCGCCGCCGGTGACCGGCGGGAGCAGGGCCCCGGCCTCTCTCAGGGTACGTACTTACACGTGTCCATCGCACGGTCTATCAGTATTTATTTATTAACTACTCTCGAGCTTAATTATGGTATTGTTGATAGTTGATGAAGTTAATTATTGTACGCCGTCTCATCGATCAGTTGGACGACTGGGTGCTGTGCCGCATCTACAAGAAGACCAGCAAGGCCGCGGCCGCGGTCGGAGATGAGCAGAGGAGCATGGAGTGCGAGGACTCCGTGGAGGACGCGGTCACCGCGTACCCGCCCTACGCCACGGCGGGCATGGCCGGCGCAGGTGCGCATGGCAGCAACTACGTTCAACTGCTCCATCATCACGACAGCCACGAGGACAACTTCCAGCTAGACGGCCTGCTCACAGAACACGACGTCGGCCTCTCGGCGGGCGCCGCCTCGCTGGGCCACCTTGCCGCGGCGGCGAGGGCCACCAAACAGTTCCTCGCCCCGTCGTCCTCAACCCCGTTCAACTGGCTCGAGGCGTCAACCGG

>HS37_*NAM-1*

GCTTTTTATTATACTGTGCACAAGTATTTTTATATTCTTCCAGTAAGTACAGCGCATGTATGTGATCCTGTCGTCGTGCTTGTTCATGCGCTCGGGCGGGATCGTCATCCATCAGAGAAGGCGACCTTCGGGGAGCACGAGTGGTACTTCTTCAGCCCGCGCGACCGCAAGTACCCCAACGGCGCGCGGCCGAACCGGGCGGCGACGTCGGGCTACTGGAAGGCCACCGGCACGGACAAGCCTATCCTGGCCTCGGCCACCGGGTGCGGCCGGGAGAAGGTCGGCGTCAAGAAGGCGCTCGTCTTCTACCGCGGGAAGCCGCCCAGGGGCCTCAAGACCAACTGGATCATGCATGAGTACCGCCTCACCGGAGCCTCTGCTGGCTCCACCACCACCAGCCGGCCGCCGCCGGTGACCGGCGGGAGCAGGGCCCCGGCCTCTCTCAGGGTACGTACTTACACGTGTCCATCGCACGGTCTATCAGTATTTATTTATTAACTACTCTCGAGCTTAATTATGGTATTGTTGATAGTTGATGAAGTTAATTATTGTACGCCGTCTCATCGATCAGTTGGACGACTGGGTGCTGTGCCGCATCTACAAGAAGACCAGCAAGGCCGCGGCCGCGGTCGGAGATGAGCAGAGGAGCATGGAGTGCGAGGACTCCGTGGAGGACGCGGTCACCGCGTACCCGCCCTACGCCACGGCGGGCATGGCCGGCGCAGGTGCGCATGGCAGCAACTACGTTCAACTGCTCCATCATCACGACAGCCACGAGGACAACTTCCAGCTAGACGGCCTGCTCACAGAACACGACGTCGGCCTCTCGGCGGGCGCCGCCTCGCTGGGCCACCTTGCCGCGGCGGCGAGGGCCACCAAACAGTTCCTCGCCCCGTCGTCCTCAACCCCGTTCAACTGGCTCGAGGCGTCAACCGG

>HS38_*NAM-1*

GCTTTTTATTATACTGTGCACAAGTATTTTTATATTCTTCCAGTAAGTACAGCGCATGTATGTGATCCTGTCGTCGTGCTTGTTCATGCGCTCGGGCGGGATCGTCATCCATCAGAGAAGGCGACCTTCGGGGAGCACGAGTGGTACTTCTTCAGCCCGCGCGACCGCAAGTACCCCAACGGCGCGCGGCCGAACCGGGCGGCGACGTCGGGCTACTGGAAGGCCACCGGCACGGACAAGCCTATCCTGGCCTCGGCCACCGGGTGCGGCCGGGAGAAGGTCGGCGTCAAGAAGGCGCTCGTCTTCTACCGCGGGAAGCCGCCCAGGGGCCTCAAGACCAACTGGATCATGCATGAGTACCGCCTCACCGGAGCCTCTGCTGGCTCCACCACCACCAGCCGGCCGCCGCCGGTGACCGGCGGGAGCAGGGCCCCGGCCTCTCTCAGGGTACGTACTTACACGTGTCCATCGCACGGTCTATCAGTATTTATTTATTAACTACTCTCGAGCTTAATTATGGTATTGTTGATAGTTGATGAAGTTAATTATTGTACGCCGTCTCATCGATCAGTTGGACGACTGGGTGCTGTGCCGCATCTACAAGAAGACCAGCAAGGCCGCGGCCGCGGTCGGAGATGAGCAGAGGAGCATGGAGTGCGAGGACTCCGTGGAGGACGCGGTCACCGCGTACCCGCCCTACGCCACGGCGGGCATGGCCGGCGCAGGTGCGCATGGCAGCAACTACGTTCAACTGCTCCATCATCACGACAGCCACGAGGACAACTTCCAGCTAGACGGCCTGCTCACAGAACACGACGTCGGCCTCTCGGCGGGCGCCGCCTCGCTGGGCCACCTTGCCGCGGCGGCGAGGGCCACCAAACAGTTCCTCGCCCCGTCGTCCTCAACCCCGTTCAACTGGCTCGAGGCGTCAACCGG

>HS39_*NAM-1*

GCTTTTTATTATACTGTGCACAAGTATTTTTATATTCTTCCAGTAAGTACAGCGCATGTATGTGATCCTGTCGTCGTGCTTGTTCATGCGCTCGGGCGGGATCGTCATCCATCAGAGAAGGCGACCTTCGGGGAGCACGAGTGGTACTTCTTCAGCCCGCGCGACCGCAAGTACCCCAACGGCGCGCGGCCGAACCGGGCGGCGACGTCGGGCTACTGGAAGGCCACCGGCACGGACAAGCCTATCCTGGCCTCGGCCACCGGGTGCGGCCGGGAGAAGGTCGGCGTCAAGAAGGCGCTCGTCTTCTACCGCGGGAAGCCGCCCAGGGGCCTCAAGACCAACTGGATCATGCATGAGTACCGCCTCACCGGAGCCTCTGCTGGCTCCACCACCACCAGCCGGCCGCCGCCGGTGACCGGCGGGAGCAGGGCCCCGGCCTCTCTCAGGGTACGTACTTACACGTGTCCATCGCACGGTCTATCAGTATTTATTTATTAACTACTCTCGAGCTTAATTATGGTATTGTTGATAGTTGATGAAGTTAATTATTGTACGCCGTCTCATCGATCAGTTGGACGACTGGGTGCTGTGCCGCATCTACAAGAAGACCAGCAAGGCCGCGGCCGCGGTCGGAGATGAGCAGAGGAGCATGGAGTGCGAGGACTCCGTGGAGGACGCGGTCACCGCGTACCCGCCCTACGCCACGGCGGGCATGGCCGGCGCAGGTGCGCATGGCAGCAACTACGTTCAACTGCTCCATCATCACGACAGCCACGAGGACAACTTCCAGCTAGACGGCCTGCTCACAGAACACGACGTCGGCCTCTCGGCGGGCGCCGCCTCGCTGGGCCACCTTGCCGCGGCGGCGAGGGCCACCAAACAGTTCCTCGCCCCGTCGTCCTCAACCCCGTTCAACTGGCTCGAGGCGTCAACCGG

>HS40_*NAM-1*

GCTTTTTATTATACTGTGCACAAGTATTTTTATATTCTTCCAGTAAGTACAGCGCATGTATGTGATCCTGTCGTCGTGCTTGTTCATGCGCTCGGGCGGGATCATCATCCATCAGAGAAGGCGACCTTCGGGGAGCACGAGTGGTACTTCTTCAGCCCGCGCGACCGCAAGTACCCCAACGGCGCGCGGCCGAACCGGGCGGCGACGTCGGGCTACTGGAAGGCCACCGGCACGGACAAGCCTATCCTGGCCTCGGCCACCGGGTGCGGCCGGGAGAAGGTCGGCGTCAAGAAGGCGCTCGTCTTCTACCGCGGGAAGCCGCCCAGGGGCCTCAAGACCAACTGGATCATGCATGAGTACCGCCTCACCGGAGCCTCTGCTGGCTCCACCACCACCAGCCGGCCGCCGCCGGTGACCGGCGGGAGCAGGGCCCCGGCCTCTCTCAGGGTACGTACTTACACGTGTCCATCGCACGGTCTATCAGTATTTATTTATTAACTACTCTCGAGCTTAATTATGGTATTGTTGATAGTTGATGAAGTTAATTATTGTACGCCGTCTCATCGATCAGTTGGACGACTGGGTGCTGTGCCGCATCTACAAGAAGACCAGCAAGGCCGCGGCCGCGGTCGGAGATGAGCAGAGGAGCATGGAGTGCGAGGACTCCGTGGAGGACGCGGTCACCGCGTACCCGCCCTACGCCACGGCGGGCATGGCCGGCGCAGGTGCGCATGGCAGCAACTACGTTCAACTGCTCCATCATCACGACAGCCACGAGGACAACTTCCAGCTAGACGGCCTGCTCACAGAACACGACGTCGGCCTCTCGGCGGGCGCCGCCTCGCTGGGCCACCTTGCCGCGGCGGCGAGGGCCACCAAACAGTTCCTCGCCCCGTCGTCCTCAACCCCGTTCAACTGGCTCGAGGCGTCAACCGG

>HS41_*NAM-1*

GCTTTTTATTATACTGTGCACAAGTATTTTTATATTCTTCCAGTAAGTACAGCGCATGTATGTGATCCTGTCGTCGTGCTTGTTCATGCGCTCGGGCGGGATCGTCATCCATCAGAGAAGGCGACCTTCGGGGAGCACGAGTGGTACTTCTTCAGCCCGCGCGACCGCAAGTACCCCAACGGCGCGCGGCCGAACCGGGCGGCGACGTCGGGCTACTGGAAGGCCACCGGCACGGACAAGCCTATCCTGGCCTCGGCCACCGGGTGCGGCCGGGAGAAGGTCGGCGTCAAGAAGGCGCTCGTCTTCTACCGCGGGAAGCCGCCCAGGGGCCTCAAGACCAACTGGATCATGCATGAGTACCGCCTCACCGGAGCCTCTGCTGGCTCCACCACCACCAGCCGGCCGCCGCCGGTGACCGGCGGGAGCAGGGCCCCGGCCTCTCTCAGGGTACGTACTTACACGTGTCCATCGCACGGTCTATCAGTATTTATTTATTAACTACTCTCGAGCTTAATTATGGTATTGTTGATAGTTGATGAAGTTAATTATTGTACGCCGTCTCATCGATCAGTTGGACGACTGGGTGCTGTGCCGCATCTACAAGAAGACCAGCAAGGCCGCGGCCGCGGTCGGAGATGAGCAGAGGAGCATGGAGTGCGAGGACTCCGTGGAGGACGCGGTCACCGCGTACCCGCCCTACGCCACGGCGGGCATGGCCGGCGCAGGTGCGCATGGCAGCAACTACGTTCAACTGCTCCATCATCACGACAGCCACGAGGACAACTTCCAGCTAGACGGCCTGCTCACAGAACACGACGTCGGCCTCTCGGCGGGCGCCGCCTCGCTGGGCCACCTTGCCGCGGCGGCGAGGGCCACCAAACAGTTCCTCGCCCCGTCGTCCTCAACCCCGTTCAACTGGCTCGAGGCGTCAACCGG

>HS42_*NAM-1*

GCTTTTTATTATACTGTGCACAAGTATTTTTATATTCTTCCAGTAAGTACAGCGCATGTATGTGATCCTGTCGTCGTGCTTGTTCATGCGCTCGGGCGGGATCATCATCCATCAGAGAAGGCGACCTTCGGGGAGCACGAGTGGTACTTCTTCAGCCCGCGCGACCGCAAGTACCCCAACGGCGCGCGGCCGAACCGGGCGGCGACGTCGGGCTACTGGAAGGCCACCGGCACGGACAAGCCTATCCTGGCCTCGGCCACCGGGTGCGGCCGGGAGAAGGTCGGCGTCAAGAAGGCGCTCGTCTTCTACCGCGGGAAGCCGCCCAGGGGCCTCAAGACCAACTGGATCATGCATGAGTACCGCCTCACCGGAGCCTCTGCTGGCTCCACCACCACCAGCCGGCCGCCGCCGGTGACCGGCGGGAGCAGGGCCCCGGCCTCTCTCAGGGTACGTACTTACACGTGTCCATCGCACGGTCTATCAGTATTTATTTATTAACTACTCTCGAGCTTAATTATGGTATTGTTGATAGTTGATGAAGTTAATTATTGTACGCCGTCTCATCGATCAGTTGGACGACTGGGTGCTGTGCCGCATCTACAAGAAGACCAGCAAGGCCGCGGCCGCGGTCGGAGATGAGCAGAGGAGCATGGAGTGCGAGGACTCCGTGGAGGACGCGGTCACCGCGTACCCGCCCTACGCCACGGCGGGCATGGCCGGCGCAGGTGCGCATGGCAGCAACTACGTTCAACTGCTCCATCATCACGACAGCCACGAGGACAACTTCCAGCTAGACGGCCTGCTCACAGAACACGACGTCGGCCTCTCGGCGGGCGCCGCCTCGCTGGGCCACCTTGCCGCGGCGGCGAGGGCCACCAAACAGTTCCTCGCCCCGTCGTCCTCAACCCCGTTCAACTGGCTCGAGGCGTCAACCGG

>HS43_*NAM-1*

GCTTTTTATTATACTGTGCACAAGTATTTTTATATTCTTCCAGTAAGTACAGCGCATGTATGTGATCCTGTCGTCGTGCTTGTTCATGCGCTCGGGCGGGATCGTCATCCATCAGAGAAGGCGACCTTCGGGGAGCACGAGTGGTACTTCTTCAGCCCGCGCGACCGCAAGTACCCCAACGGCGCGCGGCCGAACCGGGCGGCGACGTCGGGCTACTGGAAGGCCACCGGCACGGACAAGCCTATCCTGGCCTCGGCCACCGGGTGCGGCCGGGAGAAGGTCGGCGTCAAGAAGGCGCTCGTCTTCTACCGCGGGAAGCCGCCCAGGGGCCTCAAGACCAACTGGATCATGCATGAGTACCGCCTCACCGGAGCCTCTGCTGGCTCCACCACCACCAGCCGGCCGCCGCCGGTGACCGGCGGGAGCAGGGCCCCGGCCTCTCTCAGGGTACGTACTTACACGTGTCCATCGCACGGTCTATCAGTATTTATTTATTAACTACTCTCGAGCTTAATTATGGTATTGTTGATAGTTGATGAAGTTAATTATTGTACGCCGTCTCATCGATCAGTTGGACGACTGGGTGCTGTGCCGCATCTACAAGAAGACCAGCAAGGCCGCGGCCGCGGTCGGAGATGAGCAGAGGAGCATGGAGTGCGAGGACTCCGTGGAGGACGCGGTCACCGCGTACCCGCCCTACGCCACGGCGGGCATGGCCGGCGCAGGTGCGCATGGCAGCAACTACGTTCAACTGCTCCATCATCACGACAGCCACGAGGACAACTTCCAGCTAGACGGCCTGCTCACAGAACACGACGTCGGCCTCTCGGCGGGCGCCGCCTCGCTGGGCCACCTTGCCGCGGCGGCGAGGGCCACCAAACAGTTCCTCGCCCCGTCGTCCTCAACCCCGTTCAACTGGCTCGAGGCGTCAACCGG

>HS44_*NAM-1*

GCTTTTTATTATACTGTGCACAAGTATTTTTATATTCTTCCAGTAAGTACAGCGCATGTATGTGATCCTGTCGTCGTGCTTGTTCATGCGCTCGGGCGGGATCATCATCCATCAGAGAAGGCGACCTTCGGGGAGCACGAGTGGTACTTCTTCAGCCCGCGCGACCGCAAGTACCCCAACGGCGCGCGGCCGAACCGGGCGGCGACGTCGGGCTACTGGAAGGCCACCGGCACGGACAAGCCTATCCTGGCCTCGGCCACCGGGTGCGGCCGGGAGAAGGTCGGCGTCAAGAAGGCGCTCGTCTTCTACCGCGGGAAGCCGCCCAGGGGCCTCAAGACCAACTGGATCATGCATGAGTACCGCCTCACCGGAGCCTCTGCTGGCTCCACCACCACCAGCCGGCCGCCGCCGGTGACCGGCGGGAGCAGGGCCCCGGCCTCTCTCAGGGTACGTACTTACACGTGTCCATCGCACGGTCTATCAGTATTTATTTATTAACTACTCTCGAGCTTAATTATGGTATTGTTGATAGTTGATGAAGTTAATTATTGTACGCCGTCTCATCGATCAGTTGGACGACTGGGTGCTGTGCCGCATCTACAAGAAGACCAGCAAGGCCGCGGCCGCGGTCGGAGATGAGCAGAGGAGCATGGAGTGCGAGGACTCCGTGGAGGACGCGGTCACCGCGTACCCGCCCTACGCCACGGCGGGCATGGCCGGCGCAGGTGCGCATGGCAGCAACTACGTTCAACTGCTCCATCATCACGACAGCCACGAGGACAACTTCCAGCTAGACGGCCTGCTCACAGAACACGACGTCGGCCTCTCGGCGGGCGCCGCCTCGCTGGGCCACCTTGCCGCGGCGGCGAGGGCCACCAAACAGTTCCTCGCCCCGTCGTCCTCAACCCCGTTCAACTGGCTCGAGGCGTCAACCGG

>HS45_*NAM-1*

GCTTTTTATTATACTGTGCACAAGTATTTTTATATTCTTCCAGTAAGTACAGCGCATGTATGTGATCCTGTCGTCGTGCTTGTTCATGCGCTCGGGCGGGATCATCATCCATCAGAGAAGGCGACCTTCGGGGAGCACGAGTGGTACTTCTTCAGCCCGCGCGACCGCAAGTACCCCAACGGCGCGCGGCCGAACCGGGCGGCGACGTCGGGCTACTGGAAGGCCACCGGCACGGACAAGCCTATCCTGGCCTCGGCCACCGGGTGCGGCCGGGAGAAGGTCGGCGTCAAGAAGGCGCTCGTCTTCTACCGCGGGAAGCCGCCCAGGGGCCTCAAGACCAACTGGATCATGCATGAGTACCGCCTCACCGGAGCCTCTGCTGGCTCCACCACCACCAGCCGGCCGCCGCCGGTGACCGGCGGGAGCAGGGCCCCGGCCTCTCTCAGGGTACGTACTTACACGTGTCCATCGCACGGTCTATCAGTATTTATTTATTAACTACTCTCGAGCTTAATTATGGTATTGTTGATAGTTGATGAAGTTAATTATTGTACGCCGTCTCATCGATCAGTTGGACGACTGGGTGCTGTGCCGCATCTACAAGAAGACCAGCAAGGCCGCGGCCGCGGTCGGAGATGAGCAGAGGAGCATGGAGTGCGAGGACTCCGTGGAGGACGCGGTCACCGCGTACCCGCCCTACGCCACGGCGGGCATGGCCGGCGCAGGTGCGCATGGCAGCAACTACGTTCAACTGCTCCATCATCACGACAGCCACGAGGACAACTTCCAGCTAGACGGCCTGCTCACAGAACACGACGTCGGCCTCTCGGCGGGCGCCGCCTCGCTGGGCCACCTTGCCGCGGCGGCGAGGGCCACCAAACAGTTCCTCGCCCCGTCGTCCTCAACCCCGTTCAACTGGCTCGAGGCGTCAACCGG

>HS46_*NAM-1*

GCTTTTTATTATACTGTGCACAAGTATTTTTATATTCTTCCAGTAAGTACAGCGCATGTATGTGATCCTGTCGTCGTGCTTGTTCATGCGCTCGGGCGGGATCGTCATCCATCAGAGAAGGCGACCTTCGGGGAGCACGAGTGGTACTTCTTCAGCCCGCGCGACCGCAAGTACCCCAACGGCGCGCGGCCGAACCGGGCGGCGACGTCGGGCTACTGGAAGGCCACCGGCACGGACAAGCCTATCCTGGCCTCGGCCACCGGGTGCGGCCGGGAGAAGGTCGGCGTCAAGAAGGCGCTCGTCTTCTACCGCGGGAAGCCGCCCAGGGGCCTCAAGACCAACTGGATCATGCATGAGTACCGCCTCACCGGAGCCTCTGCTGGCTCCACCACCACCAGCCGGCCGCCGCCGGTGACCGGCGGGAGCAGGGCCCCGGCCTCTCTCAGGGTACGTACTTACACGTGTCCATCGCACGGTCTATCAGTATTTATTTATTAACTACTCTCGAGCTTAATTATGGTATTGTTGATAGTTGATGAAGTTAATTATTGTACGCCGTCTCATCGATCAGTTGGACGACTGGGTGCTGTGCCGCATCTACAAGAAGACCAGCAAGGCCGCGGCCGCGGTCGGAGATGAGCAGAGGAGCATGGAGTGCGAGGACTCCGTGGAGGACGCGGTCACCGCGTACCCGCCCTACGCCACGGCGGGCATGGCCGGCGCAGGTGCGCATGGCAGCAACTACGTTCAACTGCTCCATCATCACGACAGCCACGAGGACAACTTCCAGCTAGACGGCCTGCTCACAGAACACGACGTCGGCCTCTCGGCGGGCGCCGCCTCGCTGGGCCACCTTGCCGCGGCGGCGAGGGCCACCAAACAGTTCCTCGCCCCGTCGTCCTCAACCCCGTTCAACTGGCTCGAGGCGTCAACCGG

>HS47_*NAM-1*

GCTTTTTATTATACTGTGCACAAGTATTTTTATATTCTTCCAGTAAGTACAGCGCATGTATGTGATCCTGTCGTCGTGCTTGTTCATGCGCTCGGGCGGGATCATCATCCATCAGAGAAGGCGACCTTCGGGGAGCACGAGTGGTACTTCTTCAGCCCGCGCGACCGCAAGTACCCCAACGGCGCGCGGCCGAACCGGGCGGCGACGTCGGGCTACTGGAAGGCCACCGGCACGGACAAGCCTATCCTGGCCTCGGCCACCGGGTGCGGCCGGGAGAAGGTCGGCGTCAAGAAGGCGCTCGTCTTCTACCGCGGGAAGCCGCCCAGGGGCCTCAAGACCAACTGGATCATGCATGAGTACCGCCTCACCGGAGCCTCTGCTGGCTCCACCACCACCAGCCGGCCGCCGCCGGTGACCGGCGGGAGCAGGGCCCCGGCCTCTCTCAGGGTACGTACTTACACGTGTCCATCGCACGGTCTATCAGTATTTATTTATTAACTACTCTCGAGCTTAATTATGGTATTGTTGATAGTTGATGAAGTTAATTATTGTACGCCGTCTCATCGATCAGTTGGACGACTGGGTGCTGTGCCGCATCTACAAGAAGACCAGCAAGGCCGCGGCCGCGGTCGGAGATGAGCAGAGGAGCATGGAGTGCGAGGACTCCGTGGAGGACGCGGTCACCGCGTACCCGCCCTACGCCACGGCGGGCATGGCCGGCGCAGGTGCGCATGGCAGCAACTACGTTCAACTGCTCCATCATCACGACAGCCACGAGGACAACTTCCAGCTAGACGGCCTGCTCACAGAACACGACGTCGGCCTCTCGGCGGGCGCCGCCTCGCTGGGCCACCTTGCCGCGGCGGCGAGGGCCACCAAACAGTTCCTCGCCCCGTCGTCCTCAACCCCGTTCAACTGGCTCGAGGCGTCAACCGG

>HS48_*NAM-1*

GCTTTTTATTATACTGTGCACAAGTATTTTTATATTCTTCCAGTAAGTACAGCGCATGTATGTGATCCTGTCGTCGTGCTTGTTCATGCGCTCGGGCGGGATCATCATCCATCAGAGAAGGCGACCTTCGGGGAGCACGAGTGGTACTTCTTCAGCCCGCGCGACCGCAAGTACCCCAACGGCGCGCGGCCGAACCGGGCGGCGACGTCGGGCTACTGGAAGGCCACCGGCACGGACAAGCCTATCCTGGCCTCGGCCACCGGGTGCGGCCGGGAGAAGGTCGGCGTCAAGAAGGCGCTCGTCTTCTACCGCGGGAAGCCGCCCAGGGGCCTCAAGACCAACTGGATCATGCATGAGTACCGCCTCACCGGAGCCTCTGCTGGCTCCACCACCACCAGCCGGCCGCCGCCGGTGACCGGCGGGAGCAGGGCCCCGGCCTCTCTCAGGGTACGTACTTACACGTGTCCATCGCACGGTCTATCAGTATTTATTTATTAACTACTCTCGAGCTTAATTATGGTATTGTTGATAGTTGATGAAGTTAATTATTGTACGCCGTCTCATCGATCAGTTGGACGACTGGGTGCTGTGCCGCATCTACAAGAAGACCAGCAAGGCCGCGGCCGCGGTCGGAGATGAGCAGAGGAGCATGGAGTGCGAGGACTCCGTGGAGGACGCGGTCACCGCGTACCCGCCCTACGCCACGGCGGGCATGGCCGGCGCAGGTGCGCATGGCAGCAACTACGTTCAACTGCTCCATCATCACGACAGCCACGAGGACAACTTCCAGCTAGACGGCCTGCTCACAGAACACGACGTCGGCCTCTCGGCGGGCGCCGCCTCGCTGGGCCACCTTGCCGCGGCGGCGAGGGCCACCAAACAGTTCCTCGCCCCGTCGTCCTCAACCCCGTTCAACTGGCTCGAGGCGTCAACCGG

>HS49_*NAM-1*

GCTTTTTATTATACTGTGCACAAGTATTTTTATATTCTTCCAGTAAGTACAGCGCATGTATGTGATCCTGTCGTCGTGCTTGTTCATGCGCTCGGGCGGGATCATCATCCATCAGAGAAGGCGACCTTCGGGGAGCACGAGTGGTACTTCTTCAGCCCGCGCGACCGCAAGTACCCCAACGGCGCGCGGCCGAACCGGGCGGCGACGTCGGGCTACTGGAAGGCCACCGGCACGGACAAGCCTATCCTGGCCTCGGCCACCGGGTGCGGCCGGGAGAAGGTCGGCGTCAAGAAGGCGCTCGTCTTCTACCGCGGGAAGCCGCCCAGGGGCCTCAAGACCAACTGGATCATGCATGAGTACCGCCTCACCGGAGCCTCTGCTGGCTCCACCACCACCAGCCGGCCGCCGCCGGTGACCGGCGGGAGCAGGGCCCCGGCCTCTCTCAGGGTACGTCCTTACACGTGTCCATCGCACGGTCTATCAGTATTTATTTATTAACTACTCTCGAGCTTAATTATGGTATTGTTGATAGTTGATGAAGTTAATTATTGTACGCCGTCTCATCGATCAGTTGGACGACTGGGTGCTGTGCCGCATCTACAAGAAGACCAGCAAGGCCGCGGCCGCGGTCGGAGATGAGCAGAGGAGCATGGAGTGCGAGGACTCCGTGGAGGACGCGGTCACCGCGTACCCGCCCTACGCCACGGCGGGCATGGCCGGCGCAGGTGCGCATGGCAGCAACTACGTTCAACTGCTCCATCATCACGACAGCCACGAGGACAACTTCCAGCTAGACGGCCTGCTCACAGAACACGACGTCGGCCTCTCGGCGGGCGCCGCCTCGCTGGGCCACCTTGCCGCGGCGGCGAGGGCCACCAAACAGTTCCTCGCCCCGTCGTCCTCAACCCCGTTCAACTGGCTCGAGGCGTCAACCGG

>HS50_*NAM-1*

GCTTTTTATTATACTGTGCACAAGTATTTTTATATTCTTCCAGTAAGTACAGCGCATGTATGTGATCCTGTCGTCGTGCTTGTTCATGCGCTCGGGCGGGATCATCATCCATCAGAGAAGGCGACCTTCGGGGAGCACGAGTGGTACTTCTTCAGCCCGCGCGACCGCAAGTACCCCAACGGCGCGCGGCCGAACCGGGCGGCGACGTCGGGCTACTGGAAGGCCACCGGCACGGACAAGCCTATCCTGGCCTCGGCCACCGGGTGCGGCCGGGAGAAGGTCGGCGTCAAGAAGGCGCTCGTCTTCTACCGCGGGAAGCCGCCCAGGGGCCTCAAGACCAACTGGATCATGCATGAGTACCGCCTCACCGGAGCCTCTGCTGGCTCCACCACCACCAGCCGGCCGCCGCCGGTGACCGGCGGGAGCAGGGCCCCGGCCTCTCTCAGGGTACGTACTTACACGTGTCCATCGCACGGTCTATCAGTATTTATTTATTAACTACTCTCGAGCTTAATTATGGTATTGTTGATAGTTGATGAAGTTAATTATTGTACGCCGTCTCATCGATCAGTTGGACGACTGGGTGCTGTGCCGCATCTACAAGAAGACCAGCAAGGCCGCGGCCGCGGTCGGAGATGAGCAGAGGAGCATGGAGTGCGAGGACTCCGTGGAGGACGCGGTCACCGCGTACCCGCCCTACGCCACGGCGGGCATGGCCGGCGCAGGTGCGCATGGCAGCAACTACGTTCAACTGCTCCATCATCACGACAGCCACGAGGACAACTTCCAGCTAGACGGCCTGCTCACAGAACACGACGTCGGCCTCTCGGCGGGCGCCGCCTCGCTGGGCCACCTTGCCGCGGCGGCGAGGGCCACCAAACAGTTCCTCGCCCCGTCGTCCTCAACCCCGTTCAACTGGCTCGAGGCGTCAACCGG

>HS51_*NAM-1*

GCTTTTTATTATACTGTGCACAAGTATTTTTATATTCTTCCAGTAAGTACAGCGCATGTATGTGATCCTGTCGTCGTGCTTGTTCATGCGCTCGGGCGGGATCATCATCCATCAGAGAAGGCGACCTTCGGGGAGCACGAGTGGTACTTCTTCAGCCCGCGCGACCGCAAGTACCCCAACGGCGCGCGGCCGAACCGGGCGGCGACGTCGGGCTACTGGAAGGCCACCGGCACGGACAAGCCTATCCTGGCCTCGGCCACCGGGTGCGGCCGGGAGAAGGTCGGCGTCAAGAAGGCGCTCGTCTTCTACCGCGGGAAGCCGCCCAGGGGCCTCAAGACCAACTGGATCATGCATGAGTACCGCCTCACCGGAGCCTCTGCTGGCTCCACCACCACCAGCCGGCCGCCGCCGGTGACCGGCGGGAGCAGGGCCCCGGCCTCTCTCAGGGTACGTACTTACACGTGTCCATCGCACGGTCTATCAGTATTTATTTATTAACTACTCTCGAGCTTAATTATGGTATTGTTGATAGTTGATGAAGTTAATTATTGTACGCCGTCTCATCGATCAGTTGGACGACTGGGTGCTGTGCCGCATCTACAAGAAGACCAGCAAGGCCGCGGCCGCGGTCGGAGATGAGCAGAGGAGCATGGAGTGCGAGGACTCCGTGGAGGACGCGGTCACCGCGTACCCGCCCTACGCCACGGCGGGCATGGCCGGCGCAGGTGCGCATGGCAGCAACTACGTTCAACTGCTCCATCATCACGACAGCCACGAGGACAACTTCCAGCTAGACGGCCTGCTCACAGAACACGACGTCGGCCTCTCGGCGGGCGCCGCCTCGCTGGGCCACCTTGCCGCGGCGGCGAGGGCCACCAAACAGTTCCTCGCCCCGTCGTCCTCAACCCCGTTCAACTGGCTCGAGGCGTCAACCGG

>HS52_*NAM-1*

GCTTTTTATTATACTGTGCACAAGTATTTTTATATTCTTCCAGTAAGTACAGCGCATGTATGTGATCCTGTCGTCGTGCTTGTTCATGCGCTCGGGCGGGATCATCATCCATCAGAGAAGGCGACCTTCGGGGAGCACGAGTGGTACTTCTTCAGCCCGCGCGACCGCAAGTACCCCAACGGCGCGCGGCCGAACCGGGCGGCGACGTCGGGCTACTGGAAGGCCACCGGCACGGACAAGCCTATCCTGGCCTCGGCCACCGGGTGCGGCCGGGAGAAGGTCGGCGTCAAGAAGGCGCTCGTCTTCTACCGCGGGAAGCCGCCCAGGGGCCTCAAGACCAACTGGATCATGCATGAGTACCGCCTCACCGGAGCCTCTGCTGGCTCCACCACCACCAGCCGGCCGCCGCCGGTGACCGGCGGGAGCAGGGCCCCGGCCTCTCTCAGGGTACGTACTTACACGTGTCCATCGCACGGTCTATCAGTATTTATTTATTAACTACTCTCGAGCTTAATTATGGTATTGTTGATAGTTGATGAAGTTAATTATTGTACGCCGTCTCATCGATCAGTTGGACGACTGGGTGCTGTGCCGCATCTACAAGAAGACCAGCAAGGCCGCGGCCGCGGTCGGAGATGAGCAGAGGAGCATGGAGTGCGAGGACTCCGTGGAGGACGCGGTCACCGCGTACCCGCCCTACGCCACGGCGGGCATGGCCGGCGCAGGTGCGCATGGCAGCAACTACGTTCAACTGCTCCATCATCACGACAGCCACGAGGACAACTTCCAGCTAGACGGCCTGCTCACAGAACACGACGTCGGCCTCTCGGCGGGCGCCGCCTCGCTGGGCCACCTTGCCGCGGCGGCGAGGGCCACCAAACAGTTCCTCGCCCCGTCGTCCTCAACCCCGTTCAACTGGCTCGAGGCGTCAACCGG

>HS53_*NAM-1*

GCTTTTTATTATACTGTGCACAAGTATTTTTATATTCTTCCAGTAAGTACAGCGCATGTATGTGATCCTGTCGTCGTGCTTGTTCATGCGCTCGGGCGGGATCATCATCCATCAGAGAAGGCGACCTTCGGGGAGCACGAGTGGTACTTCTTCAGCCCGCGCGACCGCAAGTACCCCAACGGCGCGCGGCCGAACCGGGCGGCGACGTCGGGCTACTGGAAGGCCACCGGCACGGACAAGCCTATCCTGGCCTCGGCCACCGGGTGCGGCCGGGAGAAGGTCGGCGTCAAGAAGGCGCTCGTCTTCTACCGCGGGAAGCCGCCCAGGGGCCTCAAGACCAACTGGATCATGCATGAGTACCGCCTCACCGGAGCCTCTGCTGGCTCCACCACCACCAGCCGGCCGCCGCCGGTGACCGGCGGGAGCAGGGCCCCGGCCTCTCTCAGGGTACGTACTTACACGTGTCCATCGCACGGTCTATCAGTATTTATTTATTAACTACTCTCGAGCTTAATTATGGTATTGTTGATAGTTGATGAAGTTAATTATTGTACGCCGTCTCATCGATCAGTTGGACGACTGGGTGCTGTGCCGCATCTACAAGAAGACCAGCAAGGCCGCGGCCGCGGTCGGAGATGAGCAGAGGAGCATGGAGTGCGAGGACTCCGTGGAGGACGCGGTCACCGCGTACCCGCCCTACGCCACGGCGGGCATGGCCGGCGCAGGTGCGCATGGCAGCAACTACGTTCAACTGCTCCATCATCACGACAGCCACGAGGACAACTTCCAGCTAGACGGCCTGCTCACAGAACACGACGTCGGCCTCTCGGCGGGCGCCGCCTCGCTGGGCCACCTTGCCGCGGCGGCGAGGGCCACCAAACAGTTCCTCGCCCCGTCGTCCTCAACCCCGTTCAACTGGCTCGAGGCGTCAACCGG

>HS54_*NAM-1*

GCTTTTTATTATACTGTGCACAAGTATTTTTATATTCTTCCAGTAAGTACAGCGCATGTATGTGATCCTGTCGTCGTGCTTGTTCATGCGCTCGGGCGGGATCATCATCCATCAGAGAAGGCGACCTTCGGGGAGCACGAGTGGTACTTCTTCAGCCCGCGCGACCGCAAGTACCCCAACGGCGCGCGGCCGAACCGGGCGGCGACGTCGGGCTACTGGAAGGCCACCGGCACGGACAAGCCTATCCTGGCCTCGGCCACCGGGTGCGGCCGGGAGAAGGTCGGCGTCAAGAAGGCGCTCGTCTTCTACCGCGGGAAGCCGCCCAGGGGCCTCAAGACCAACTGGATCATGCATGAGTACCGCCTCACCGGAGCCTCTGCTGGCTCCACCACCACCAGCCGGCCGCCGCCGGTGACCGGCGGGAGCAGGGCCCCGGCCTCTCTCAGGGTACGTACTTACACGTGTCCATCGCACGGTCTATCAGTATTTATTTATTAACTACTCTCGAGCTTAATTATGGTATTGTTGATAGTTGATGAAGTTAATTATTGTACGCCGTCTCATCGATCAGTTGGACGACTGGGTGCTGTGCCGCATCTACAAGAAGACCAGCAAGGCCGCGGCCGCGGTCGGAGATGAGCAGAGGAGCATGGAGTGCGAGGACTCCGTGGAGGACGCGGTCACCGCGTACCCGCCCTACGCCACGGCGGGCATGGCCGGCGCAGGTGCGCATGGCAGCAACTACGTTCAACTGCTCCATCATCACGACAGCCACGAGGACAACTTCCAGCTAGACGGCCTGCTCACAGAACACGACGTCGGCCTCTCGGCGGGCGCCGCCTCGCTGGGCCACCTTGCCGCGGCGGCGAGGGCCACCAAACAGTTCCTCGCCCCGTCGTCCTCAACCCCGTTCAACTGGCTCGAGGCGTCAACCGG

>HS55_*NAM-1*

GCTTTTTATTATACTGTGCACAAGTATTTTTATATTCTTCCAGTAAGTACAGCGCATGTATGTGATCCTGTCGTCGTGCTTGTTCATGCGCTCGGGCGGGATCATCATCCATCAGAGAAGGCGACCTTCGGGGAGCACGAGTGGTACTTCTTCAGCCCGCGCGACCGCAAGTACCCCAACGGCGCGCGGCCGAACCGGGCGGCGACGTCGGGCTACTGGAAGGCCACCGGCACGGACAAGCCTATCCTGGCCTCGGCCACCGGGTGCGGCCGGGAGAAGGTCGGCGTCAAGAAGGCGCTCGTCTTCTACCGCGGGAAGCCGCCCAGGGGCCTCAAGACCAACTGGATCATGCATGAGTACCGCCTCACCGGAGCCTCTGCTGGCTCCACCACCACCAGCCGGCCGCCGCCGGTGACCGGCGGGAGCAGGGCCCCGGCCTCTCTCAGGGTACGTACTTACACGTGTCCATCGCACGGTCTATCAGTATTTATTTATTAACTACTCTCGAGCTTAATTATGGTATTGTTGATAGTTGATGAAGTTAATTATTGTACGCCGTCTCATCGATCAGTTGGACGACTGGGTGCTGTGCCGCATCTACAAGAAGACCAGCAAGGCCGCGGCCGCGGTCGGAGATGAGCAGAGGAGCATGGAGTGCGAGGACTCCGTGGAGGACGCGGTCACCGCGTACCCGCCCTACGCCACGGCGGGCATGGCCGGCGCAGGTGCGCATGGCAGCAACTACGTTCAACTGCTCCATCATCACGACAGCCACGAGGACAACTTCCAGCTAGACGGCCTGCTCACAGAACACGACGTCGGCCTCTCGGCGGGCGCCGCCTCGCTGGGCCACCTTGCCGCGGCGGCGAGGGCCACCAAACAGTTCCTCGCCCCGTCGTCCTCAACCCCGTTCAACTGGCTCGAGGCGTCAACCGG

>HS56_*NAM-1*

GCTTTTTATTATACTGTGCACAAGTATTTTTATATTCTTCCAGTAAGTACAGCGCATGTATGTGATCCTGTCGTCGTGCTTGTTCATGCGCTCGGGCGGGATCATCATCCATCAGAGAAGGCGACCTTCGGGGAGCACGAGTGGTACTTCTTCAGCCCGCGCGACCGCAAGTACCCCAACGGCGCGCGGCCGAACCGGGCGGCGACGTCGGGCTACTGGAAGGCCACCGGCACGGACAAGCCTATCCTGGCCTCGGCCACCGGGTGCGGCCGGGAGAAGGTCGGCGTCAAGAAGGCGCTCGTCTTCTACCGCGGGAAGCCGCCCAGGGGCCTCAAGACCAACTGGATCATGCATGAGTACCGCCTCACCGGAGCCTCTGCTGGCTCCACCACCACCAGCCGGCCGCCGCCGGTGACCGGCGGGAGCAGGGCCCCGGCCTCTCTCAGGGTACGTACTTACACGTGTCCATCGCACGGTCTATCAGTATTTATTTATTAACTACTCTCGAGCTTAATTATGGTATTGTTGATAGTTGATGAAGTTAATTATTGTACGCCGTCTCATCGATCAGTTGGACGACTGGGTGCTGTGCCGCATCTACAAGAAGACCAGCAAGGCCGCGGCCGCGGTCGGAGATGAGCAGAGGAGCATGGAGTGCGAGGACTCCGTGGAGGACGCGGTCACCGCGTACCCGCCCTACGCCACGGCGGGCATGGCCGGCGCAGGTGCGCATGGCAGCAACTACGTTCAACTGCTCCATCATCACGACAGCCACGAGGACAACTTCCAGCTAGACGGCCTGCTCACAGAACACGACGTCGGCCTCTCGGCGGGCGCCGCCTCGCTGGGCCACCTTGCCGCGGCGGCGAGGGCCACCAAACAGTTCCTCGCCCCGTCGTCCTCAACCCCGTTCAACTGGCTCGAGGCGTCAACCGG

>HS57_*NAM-1*

GCTTTTTATTATACTGTGCACAAGTATTTTTATATTCTTCCAGTAAGTACAGCGCATGTATGTGATCCTGTCGTCGTGCTTGTTCATGCGCTCGGGCGGGATCATCATCCATCAGAGAAGGCGACCTTCGGGGAGCACGAGTGGTACTTCTTCAGCCCGCGCGACCGCAAGTACCCCAACGGCGCGCGGCCGAACCGGGCGGCGACGTCGGGCTACTGGAAGGCCACCGGCACGGACAAGCCTATCCTGGCCTCGGCCACCGGGTGCGGCCGGGAGAAGGTCGGCGTCAAGAAGGCGCTCGTCTTCTACCGCGGGAAGCCGCCCAGGGGCCTCAAGACCAACTGGATCATGCATGAGTACCGCCTCACCGGAGCCTCTGCTGGCTCCACCACCACCAGCCGGCCGCCGCCGGTGACCGGCGGGAGCAGGGCCCCGGCCTCTCTCAGGGTACGTACTTACACGTGTCCATCGCACGGTCTATCAGTATTTATTTATTAACTACTCTCGAGCTTAATTATGGTATTGTTGATAGTTGATGAAGTTAATTATTGTACGCCGTCTCATCGATCAGTTGGACGACTGGGTGCTGTGCCGCATCTACAAGAAGACCAGCAAGGCCGCGGCCGCGGTCGGAGATGAGCAGAGGAGCATGGAGTGCGAGGACTCCGTGGAGGACGCGGTCACCGCGTACCCGCCCTACGCCACGGCGGGCATGGCCGGCGCAGGTGCGCATGGCAGCAACTACGTTCAACTGCTCCATCATCACGACAGCCACGAGGACAACTTCCAGCTAGACGGCCTGCTCACAGAACACGACGTCGGCCTCTCGGCGGGCGCCGCCTCGCTGGGCCACCTTGCCGCGGCGGCGAGGGCCACCAAACAGTTCCTCGCCCCGTCGTCCTCAACCCCGTTCAACTGGCTCGAGGCGTCAACCGG

>HS58_*NAM-1*

GCTTTTTATTATACTGTGCACAAGTATTTTTATATTCTTCCAGTAAGTACAGCGCATGTATGTGATCCTGTCGTCGTGCTTGTTCATGCGCTCGGGCGGGATCATCATCCATCAGAGAAGGCGACCTTCGGGGAGCACGAGTGGTACTTCTTCAGCCCGCGCGACCGCAAGTACCCCAACGGCGCGCGGCCGAACCGGGCGGCGACGTCGGGCTACTGGAAGGCCACCGGCACGGACAAGCCTATCCTGGCCTCGGCCACCGGGTGCGGCCGGGAGAAGGTCGGCGTCAAGAAGGCGCTCGTCTTCTACCGCGGGAAGCCGCCCAGGGGCCTCAAGACCAACTGGATCATGCATGAGTACCGCCTCACCGGAGCCTCTGCTGGCTCCACCACCACCAGCCGGCCGCCGCCGGTGACCGGCGGGAGCAGGGCCCCGGCCTCTCTCAGGGTACGTACTTACACGTGTCCATCGCACGGTCTATCAGTATTTATTTATTAACTACTCTCGAGCTTAATTATGGTATTGTTGATAGTTGATGAAGTTAATTATTGTACGCCGTCTCATCGATCAGTTGGACGACTGGGTGCTGTGCCGCATCTACAAGAAGACCAGCAAGGCCGCGGCCGCGGTCGGAGATGAGCAGAGGAGCATGGAGTGCGAGGACTCCGTGGAGGACGCGGTCACCGCGTACCCGCCCTACGCCACGGCGGGCATGGCCGGCGCAGGTGCGCATGGCAGCAACTACGTTCAACTGCTCCATCATCACGACAGCCACGAGGACAACTTCCAGCTAGACGGCCTGCTCACAGAACACGACGTCGGCCTCTCGGCGGGCGCCGCCTCGCTGGGCCACCTTGCCGCGGCGGCGAGGGCCACCAAACAGTTCCTCGCCCCGTCGTCCTCAACCCCGTTCAACTGGCTCGAGGCGTCAACCGG

>HS59_*NAM-1*

GCTTTTTATTATACTGTGCACAAGTATTTTTATATTCTTCCAGTAAGTACAGCGCATGTATGTGATCCTGTCGTCGTGCTTGTTCATGCGCTCGGGCGGGATCATCATCCATCAGAGAAGGCGACCTTCGGGGAGCACGAGTGGTACTTCTTCAGCCCGCGCGACCGCAAGTACCCCAACGGCGCGCGGCCGAACCGGGCGGCGACGTCGGGCTACTGGAAGGCCACCGGCACGGACAAGCCTATCCTGGCCTCGGCCACCGGGTGCGGCCGGGAGAAGGTCGGCGTCAAGAAGGCGCTCGTCTTCTACCGCGGGAAGCCGCCCAGGGGCCTCAAGACCAACTGGATCATGCATGAGTACCGCCTCACCGGAGCCTCTGCTGGCTCCACCACCACCAGCCGGCCGCCGCCGGTGACCGGCGGGAGCAGGGCCCCGGCCTCTCTCAGGGTACGTACTTACACGTGTCCATCGCACGGTCTATCAGTATTTATTTATTAACTACTCTCGAGCTTAATTATGGTATTGTTGATAGTTGATGAAGTTAATTATTGTACGCCGTCTCATCGATCAGTTGGACGACTGGGTGCTGTGCCGCATCTACAAGAAGACCAGCAAGGCCGCGGCCGCGGTCGGAGATGAGCAGAGGAGCATGGAGTGCGAGGACTCCGTGGAGGACGCGGTCACCGCGTACCCGCCCTACGCCACGGCGGGCATGGCCGGCGCAGGTGCGCATGGCAGCAACTACGTTCAACTGCTCCATCATCACGACAGCCACGAGGACAACTTCCAGCTAGACGGCCTGCTCACAGAACACGACGTCGGCCTCTCGGCGGGCGCCGCCTCGCTGGGCCACCTTGCCGCGGCGGCGAGGGCCACCAAACAGTTCCTCGCCCCGTCGTCCTCAACCCCGTTCAACTGGCTCGAGGCGTCAACCGG

>HS60_*NAM-1*

GCTTTTTATTATACTGTGCACAAGTATTTTTATATTCTTCCAGTAAGTACAGCGCATGTATGTGATCCTGTCGTCGTGCTTGTTCATGCGCTCGGGCGGGATCATCATCCATCAGAGAAGGCGACCTTCGGGGAGCACGAGTGGTACTTCTTCAGCCCGCGCGACCGCAAGTACCCCAACGGCGCGCGGCCGAACCGGGCGGCGACGTCGGGCTACTGGAAGGCCACCGGCACGGACAAGCCTATCCTGGCCTCGGCCACCGGGTGCGGCCGGGAGAAGGTCGGCGTCAAGAAGGCGCTCGTCTTCTACCGCGGGAAGCCGCCCAGGGGCCTCAAGACCAACTGGATCATGCATGAGTACCGCCTCACCGGAGCCTCTGCTGGCTCCACCACCACCAGCCGGCCGCCGCCGGTGACCGGCGGGAGCAGGGCCCCGGCCTCTCTCAGGGTACGTACTTACACGTGTCCATCGCACGGTCTATCAGTATTTATTTATTAACTACTCTCGAGCTTAATTATGGTATTGTTGATAGTTGATGAAGTTAATTATTGTACGCCGTCTCATCGATCAGTTGGACGACTGGGTGCTGTGCCGCATCTACAAGAAGACCAGCAAGGCCGCGGCCGCGGTCGGAGATGAGCAGAGGAGCATGGAGTGCGAGGACTCCGTGGAGGACGCGGTCACCGCGTACCCGCCCTACGCCACGGCGGGCATGGCCGGCGCAGGTGCGCATGGCAGCAACTACGTTCAACTGCTCCATCATCACGACAGCCACGAGGACAACTTCCAGCTAGACGGCCTGCTCACAGAACACGACGTCGGCCTCTCGGCGGGCGCCGCCTCGCTGGGCCACCTTGCCGCGGCGGCGAGGGCCACCAAACAGTTCCTCGCCCCGTCGTCCTCAACCCCGTTCAACTGGCTCGAGGCGTCAACCGG

>HS63_*NAM-1*

GCTTTTTATTATACTGTGCACAAGTATTTTTATATTCTTCCAGTAAGTACAGCGCATGTATGTGATCCTGTCGTCGTGCTTGTTCATGCGCTCGGGCGGGATCATCATCCATCAGAGAAGGCGACCTTCGGGGAGCACGAGTGGTACTTCTTCAGCCCGCGCGACCGCAAGTACCCCAACGGCGCGCGGCCGAACCGGGCGGCGACGTCGGGCTACTGGAAGGCCACCGGCACGGACAAGCCTATCATGGCCTCGGCCACCGGGTGCGGCCGGGAGAAGGTCGGCGTCAAGAAGGCGCTCGTCTTCTACCGCGGGAAGCCGCCCAGGGGCCTCAAGACCAACTGGATCATGCATGAGTACCGCCTCACCGGAGCCTCTGCTGGCTCCACCACCACCAGCCGGCCGCCGCCGGTGACCGGCGGGAGCAGGGCCCCGGCCTCTCTCAGGGTACGTACTTACACGTGTCCATCGCACGGTCTATCAGTATTTATTTATTAACTACTCTCGAGCTTAATTATGGTATTGTTGATAGTTGATGAAGTTAATTATTGTACGCCGTCTCATCGATCAGTTGGACGACTGGGTGCTGTGCCGCATCTACAAGAAGACCAGCAAGGCCGCGGCCGCGGTCGGAGATGAGCAGAGGAGCATGGAGTGCGAGGACTCCGTGGAGGACGCGGTCACCGCGTACCCGCCCTACGCCACGGCGGGCATGGCCGGCGCAGGTGCGCATGGCAGCAACTACGTTCAACTGCTCCATCATCACGACAGCCACGAGGACAACTTCCAGCTAGACGGCCTGCTCACAGAACACGACGTCGGCCTCTCGGCGGGCGCCGCCTCGCTGGGCCACCTTGCCGCGGCGGCGAGGGCCACCAAACAGTTCCTCGCCCCGTCGTCCTCAACCCCGTTCAACTGGCTCGAGGCGTCAACCGG

>HS64_*NAM-1*

GCTTTTTATTATACTGTGCACAAGTATTTTTATATTCTTCCAGTAAGTACAGCGCATGTATGTGATCCTGTCGTCGTGCTTGTTCATGCGCTCGGGCGGGATCATCATCCATCAGAGAAGGCGACCTTCGGGGAGCACGAGTGGTACTTCTTCAGCCCGCGCGACCGCAAGTACCCCAACGGCGCGCGGCCGAACCGGGCGGCGACGTCGGGCTACTGGAAGGCCACCGGCACGGACAAGCCTATCCTGGCCTCGGCCACCGGGTGCGGCCGGGAGAAGGTCGGCGTCAAGAAGGCGCTCGTCTTCTACCGCGGGAAGCCGCCCAGGGGCCTCAAGACCAACTGGATCATGCATGAGTACCGCCTCACCGGAGCCTCTGCTGGCTCCACCACCACCAGCCGGCCGCCGCCGGTGACCGGCGGGAGCAGGGCCCCGGCCTCTCTCAGGGTACGTACTTACACGTGTCCATCGCACGGTCTATCAGTATTTATTTATTAACTACTCTCGAGCTTAATTATGGTATTGTTGATAGTTGATGAAGTTAATTATTGTACGCCGTCTCATCGATCAGTTGGACGACTGGGTGCTGTGCCGCATCTACAAGAAGACCAGCAAGGCCGCGGCCGCGGTCGGAGATGAGCAGAGGAGCATGGAGTGCGAGGACTCCGTGGAGGACGCGGTCACCGCGTACCCGCCCTACGCCACGGCGGGCATGGCCGGCGCAGGTGCGCATGGCAGCAACTACGTTCAACTGCTCCATCATCACGACAGCCACGAGGACAACTTCCAGCTAGACGGCCTGCTCACAGAACACGACGTCGGCCTCTCGGCGGGCGCCGCCTCGCTGGGCCACCTTGCCGCGGCGGCGAGGGCCACCAAACAGTTCCTCGCCCCGTCGTCCTCAACCCCGTTCAACTGGCTCGAGGCGTCAACCGG

>HS65_*NAM-1*

GCTTTTTATTATACTGTGCACAAGTATTTTTATATTCTTCCAGTAAGTACAGCGCATGTATGTGATCCTGTCGTCGTGCTTGTTCATGCGCTCGGGCGGGATCATCATCCATCAGAGAAGGCGACCTTCGGGGAGCACGAGTGGTACTTCTTCAGCCCGCGCGACCGCAAGTACCCCAACGGCGCGCGGCCGAACCGGGCGGCGACGTCGGGCTACTGGAAGGCCACCGGCACGGACAAGCCTATCCTGGCCTCGGCCACCGGGTGCGGCCGGGAGAAGGTCGGCGTCAAGAAGGCGCTCGTCTTCTACCGCGGGAAGCCGCCCAGGGGCCTCAAGACCAACTGGATCATGCATGAGTACCGCCTCACCGGAGCCTCTGCTGGCTCCACCACCACCAGCCGGCCGCCGCCGGTGACCGGCGGGAGCAGGGCCCCGGCCTCTCTCAGGGTACGTACTTACACGTGTCCATCGCACGGTCTATCAGTATTTATTTATTAACTACTCTCGAGCTTAATTATGGTATTGTTGATAGTTGATGAAGTTAATTATTGTACGCCGTCTCATCGATCAGTTGGACGACTGGGTGCTGTGCCGCATCTACAAGAAGACCAGCAAGGCCGCGGCCGCGGTCGGAGATGAGCAGAGGAGCATGGAGTGCGAGGACTCCGTGGAGGACGCGGTCACCGCGTACCCGCCCTACGCCACGGCGGGCATGGCCGGCGCAGGTGCGCATGGCAGCAACTACGTTCAACTGCTCCATCATCACGACAGCCACGAGGACAACTTCCAGCTAGACGGCCTGCTCACAGAACACGACGTCGGCCTCTCGGCGGGCGCCGCCTCGCTGGGCCACCTTGCCGCGGCGGCGAGGGCCACCAAACAGTTCCTCGCCCCGTCGTCCTCAACCCCGTTCAACTGGCTCGAGGCGTCAACCGG

>HS66_*NAM-1*

GCTTTTTATTATACTGTGCACAAGTATTTTTATATTCTTCCAGTAAGTACAGCGCATGTATGTGATCCTGTCGTCGTGCTTGTTCATGCGCTCGGGCGGGATCATCATCCATCAGAGAAGGCGACCTTCGGGGAGCACGAGTGGTACTTCTTCAGCCCGCGCGACCGCAAGTACCCCAACGGCGCGCGGCCGAACCGGGCGGCGACGTCGGGCTACTGGAAGGCCACCGGCACGGACAAGCCTATCATGGCCTCGGCCACCGGGTGCGGCCGGGAGAAGGTCGGCGTCAAGAAGGCGCTCGTCTTCTACCGCGGGAAGCCGCCCAGGGGCCTCAAGACCAACTGGATCATGCATGAGTACCGCCTCACCGGAGCCTCTGCTGGCTCCACCACCACCAGCCGGCCGCCGCCGGTGACCGGCGGGAGCAGGGCCCCGGCCTCTCTCAGGGTACGTACTTACACGTGTCCATCGCACGGTCTATCAGTATTTATTTATTAACTACTCTCGAGCTTAATTATGGTATTGTTGATAGTTGATGAAGTTAATTATTGTACGCCGTCTCATCGATCAGTTGGACGACTGGGTGCTGTGCCGCATCTACAAGAAGACCAGCAAGGCCGCGGCCGCGGTCGGAGATGAGCAGAGGAGCATGGAGTGCGAGGACTCCGTGGAGGACGCGGTCACCGCGTACCCGCCCTACGCCACGGCGGGCATGGCCGGCGCAGGTGCGCATGGCAGCAACTACGTTCAACTGCTCCATCATCACGACAGCCACGAGGACAACTTCCAGCTAGACGGCCTGCTCACAGAACACGACGTCGGCCTCTCGGCGGGCGCCGCCTCGCTGGGCCACCTTGCCGCGGCGGCGAGGGCCACCAAACAGTTCCTCGCCCCGTCGTCCTCAACCCCGTTCAACTGGCTCGAGGCGTCAACCGG

>HS67_*NAM-1*

GCTTTTTATTATACTGTGCACAAGTATTTTTATATTCTTCCAGTAAGTACAGCGCATGTATGTGATCCTGTCGTCGTGCTTGTTCATGCGCTCGGGCGGGATCATCATCCATCAGAGAAGGCGACCTTCGGGGAGCACGAGTGGTACTTCTTCAGCCCGCGCGACCGCAAGTACCCCAACGGCGCGCGGCCGAACCGGGCGGCGACGTCGGGCTACTGGAAGGCCACCGGCACGGACAAGCCTATCCTGGCCTCGGCCACCGGGTGCGGCCGGGAGAAGGTCGGCGTCAAGAAGGCGCTCGTCTTCTACCGCGGGAAGCCGCCCAGGGGCCTCAAGACCAACTGGATCATGCATGAGTACCGCCTCACCGGAGCCTCTGCTGGCTCCACCACCACCAGCCGGCCGCCGCCGGTGACCGGCGGGAGCAGGGCCCCGGCCTCTCTCAGGGTACGTACTTACACGTGTCCATCGCACGGTCTATCAGTATTTATTTATTAACTACTCTCGAGCTTAATTATGGTATTGTTGATAGTTGATGAAGTTAATTATTGTACGCCGTCTCATCGATCAGTTGGACGACTGGGTGCTGTGCCGCATCTACAAGAAGACCAGCAAGGCCGCGGCCGCGGTCGGAGATGAGCAGAGGAGCATGGAGTGCGAGGACTCCGTGGAGGACGCGGTCACCGCGTACCCGCCCTACGCCACGGCGGGCATGGCCGGCGCAGGTGCGCATGGCAGCAACTACGTTCAACTGCTCCATCATCACGACAGCCACGAGGACAACTTCCAGCTAGACGGCCTGCTCACAGAACACGACGTCGGCCTCTCGGCGGGCGCCGCCTCGCTGGGCCACCTTGCCGCGGCGGCGAGGGCCACCAAACAGTTCCTCGCCCCGTCGTCCTCAACCCCGTTCAACTGGCTCGAGGCGTCAACCGG

>HS68_*NAM-1*

GCTTTTTATTATACTGTGCACAAGTATTTTTATATTCTTCCAGTAAGTACAGCGCATGTATGTGATCCTGTCGTCGTGCTTGTTCATGCGCTCGGGCGGGATCATCATCCATCAGAGAAGGCGACCTTCGGGGAGCACGAGTGGTACTTCTTCAGCCCGCGCGACCGCAAGTACCCCAACGGCGCGCGGCCGAACCGGGCGGCGACGTCGGGCTACTGGAAGGCCACCGGCACGGACAAGCCTATCCTGGCCTCGGCCACCGGGTGCGGCCGGGAGAAGGTCGGCGTCAAGAAGGCGCTCGTCTTCTACCGCGGGAAGCCGCCCAGGGGCCTCAAGACCAACTGGATCATGCATGAGTACCGCCTCACCGGAGCCTCTGCTGGCTCCACCACCACCAGCCGGCCGCCGCCGGTGACCGGCGGGAGCAGGGCCCCGGCCTCTCTCAGGGTACGTACTTACACGTGTCCATCGCACGGTCTATCAGTATTTATTTATTAACTACTCTCGAGCTTAATTATGGTATTGTTGATAGTTGATGAAGTTAATTATTGTACGCCGTCTCATCGATCAGTTGGACGACTGGGTGCTGTGCCGCATCTACAAGAAGACCAGCAAGGCCGCGGCCGCGGTCGGAGATGAGCAGAGGAGCATGGAGTGCGAGGACTCCGTGGAGGACGCGGTCACCGCGTACCCGCCCTACGCCACGGCGGGCATGGCCGGCGCAGGTGCGCATGGCAGCAACTACGTTCAACTGCTCCATCATCACGACAGCCACGAGGACAACTTCCAGCTAGACGGCCTGCTCACAGAACACGACGTCGGCCTCTCGGCGGGCGCCGCCTCGCTGGGCCACCTTGCCGCGGCGGCGAGGGCCACCAAACAGTTCCTCGCCCCGTCGTCCTCAACCCCGTTCAACTGGCTCGAGGCGTCAACCGG

>HS69_*NAM-1*

GCTTTTTATTATACTGTGCACAAGTATTTTTATATTCTTCCAGTAAGTACAGCGCATGTATGTGATCCTGTCGTCGTGCTTGTTCATGCGCTCGGGCGGGATCATCATCCATCAGAGAAGGCGACCTTCGGGGAGCACGAGTGGTACTTCTTCAGCCCGCGCGACCGCAAGTACCCCAACGGCGCGCGGCCGAACCGGGCGGCGACGTCGGGCTACTGGAAGGCCACCGGCACGGACAAGCCTATCCTGGCCTCGGCCACCGGGTGCGGCCGGGAGAAGGTCGGCGTCAAGAAGGCGCTCGTCTTCTACCGCGGGAAGCCGCCCAGGGGCCTCAAGACCAACTGGATCATGCATGAGTACCGCCTCACCGGAGCCTCTGCTGGCTCCACCACCACCAGCCGGCCGCCGCCGGTGACCGGCGGGAGCAGGGCCCCGGCCTCTCTCAGGGTACGTACTTACACGTGTCCATCGCACGGTCTATCAGTATTTATTTATTAACTACTCTCGAGCTTAATTATGGTATTGTTGATAGTTGATGAAGTTAATTATTGTACGCCGTCTCATCGATCAGTTGGACGACTGGGTGCTGTGCCGCATCTACAAGAAGACCAGCAAGGCCGCGGCCGCGGTCGGAGATGAGCAGAGGAGCATGGAGTGCGAGGACTCCGTGGAGGACGCGGTCACCGCGTACCCGCCCTACGCCACGGCGGGCATGGCCGGCGCAGGTGCGCATGGCAGCAACTACGTTCAACTGCTCCATCATCACGACAGCCACGAGGACAACTTCCAGCTAGACGGCCTGCTCACAGAACACGACGTCGGCCTCTCGGCGGGCGCCGCCTCGCTGGGCCACCTTGCCGCGGCGGCGAGGGCCACCAAACAGTTCCTCGCCCCGTCGTCCTCAACCCCGTTCAACTGGCTCGAGGCGTCAACCGG

>HS70_*NAM-1*

GCTTTTTATTATACTGTGCACAAGTATTTTTATATTCTTCCAGTAAGTACAGCGCATGTATGTGATCCTGTCGTCGTGCTTGTTCATGCGCTCGGGCGGGATCATCATCCATCAGAGAAGGCGACCTTCGGGGAGCACGAGTGGTACTTCTTCAGCCCGCGCGACCGCAAGTACCCCAACGGCGCGCGGCCGAACCGGGCGGCGACGTCGGGCTACTGGAAGGCCACCGGCACGGACAAGCCTATCCTGGCCTCGGCCACCGGGTGCGGCCGGGAGAAGGTCGGCGTCAAGAAGGCGCTCGTCTTCTACCGCGGGAAGCCGCCCAGGGGCCTCAAGACCAACTGGATCATGCATGAGTACCGCCTCACCGGAGCCTCTGCTGGCTCCACCACCACCAGCCGGCCGCCGCCGGTGACCGGCGGGAGCAGGGCCCCGGCCTCTCTCAGGGTACGTACTTACACGTGTCCATCGCACGGTCTATCAGTATTTATTTATTAACTACTCTCGAGCTTAATTATGGTATTGTTGATAGTTGATGAAGTTAATTATTGTACGCCGTCTCATCGATCAGTTGGACGACTGGGTGCTGTGCCGCATCTACAAGAAGACCAGCAAGGCCGCGGCCGCGGTCGGAGATGAGCAGAGGAGCATGGAGTGCGAGGACTCCGTGGAGGACGCGGTCACCGCGTACCCGCCCTACGCCACGGCGGGCATGGCCGGCGCAGGTGCGCATGGCAGCAACTACGTTCAACTGCTCCATCATCACGACAGCCACGAGGACAACTTCCAGCTAGACGGCCTGCTCACAGAACACGACGTCGGCCTCTCGGCGGGCGCCGCCTCGCTGGGCCACCTTGCCGCGGCGGCGAGGGCCACCAAACAGTTCCTCGCCCCGTCGTCCTCAACCCCGTTCAACTGGCTCGAGGCGTCAACCGG

>HS71_*NAM-1*

GCTTTTTATTATACTGTGCACAAGTATTTTTATATTCTTCCAGTAAGTACAGCGCATGTATGTGATCCTGTCGTCGTGCTTGTTCATGCGCTCGGGCGGGATCATCATCCATCAGAGAAGGCGACCTTCGGGGAGCACGAGTGGTACTTCTTCAGCCCGCGCGACCGCAAGTACCCCAACGGCGCGCGGCCGAACCGGGCGGCGACGTCGGGCTACTGGAAGGCCACCGGCACGGACAAGCCTATCCTGGCCTCGGCCACCGGGTGCGGCCGGGAGAAGGTCGGCGTCAAGAAGGCGCTCGTCTTCTACCGCGGGAAGCCGCCCAGGGGCCTCAAGACCAACTGGATCATGCATGAGTACCGCCTCACCGGAGCCTCTGCTGGCTCCACCACCACCAGCCGGCCGCCGCCGGTGACCGGCGGGAGCAGGGCCCCGGCCTCTCTCAGGGTACGTACTTACACGTGTCCATCGCACGGTCTATCAGTATTTATTTATTAACTACTCTCGAGCTTAATTATGGTATTGTTGATAGTTGATGAAGTTAATTATTGTACGCCGTCTCATCGATCAGTTGGACGACTGGGTGCTGTGCCGCATCTACAAGAAGACCAGCAAGGCCGCGGCCGCGGTCGGAGATGAGCAGAGGAGCATGGAGTGCGAGGACTCCGTGGAGGACGCGGTCACCGCGTACCCGCCCTACGCCACGGCGGGCATGGCCGGCGCAGGTGCGCATGGCAGCAACTACGTTCAACTGCTCCATCATCACGACAGCCACGAGGACAACTTCCAGCTAGACGGCCTGCTCACAGAACACGACGTCGGCCTCTCGGCGGGCGCCGCCTCGCTGGGCCACCTTGCCGCGGCGGCGAGGGCCACCAAACAGTTCCTCGCCCCGTCGTCCTCAACCCCGTTCAACTGGCTCGAGGCGTCAACCGG

>HS72_*NAM-1*

GCTTTTTATTATACTGTGCACAAGTATTTTTATATTCTTCCAGTAAGTACAGCGCATGTATGTGATCCTGTCGTCGTGCTTGTTCATGCGCTCGGGCGGGATCATCATCCATCAGAGAAGGCGACCTTCGGGGAGCACGAGTGGTACTTCTTCAGCCCGCGCGACCGCAAGTACCCCAACGGCGCGCGGCCGAACCGGGCGGCGACGTCGGGCTACTGGAAGGCCACCGGCACGGACAAGCCTATCCTGGCCTCGGCCACCGGGTGCGGCCGGGAGAAGGTCGGCGTCAAGAAGGCGCTCGTCTTCTACCGCGGGAAGCCGCCCAGGGGCCTCAAGACCAACTGGATCATGCATGAGTACCGCCTCACCGGAGCCTCTGCTGGCTCCACCACCACCAGCCGGCCGCCGCCGGTGACCGGCGGGAGCAGGGCCCCGGCCTCTCTCAGGGTACGTACTTACACGTGTCCATCGCACGGTCTATCAGTATTTATTTATTAACTACTCTCGAGCTTAATTATGGTATTGTTGATAGTTGATGAAGTTAATTATTGTACGCCGTCTCATCGATCAGTTGGACGACTGGGTGCTGTGCCGCATCTACAAGAAGACCAGCAAGGCCGCGGCCGCGGTCGGAGATGAGCAGAGGAGCATGGAGTGCGAGGACTCCGTGGAGGACGCGGTCACCGCGTACCCGCCCTACGCCACGGCGGGCATGGCCGGCGCAGGTGCGCATGGCAGCAACTACGTTCAACTGCTCCATCATCACGACAGCCACGAGGACAACTTCCAGCTAGACGGCCTGCTCACAGAACACGACGTCGGCCTCTCGGCGGGCGCCGCCTCGCTGGGCCACCTTGCCGCGGCGGCGAGGGCCACCAAACAGTTCCTCGCCCCGTCGTCCTCAACCCCGTTCAACTGGCTCGAGGCGTCAACCGG

>HS73_*NAM-1*

GCTTTTTATTATACTGTGCACAAGTATTTTTATATTCTTCCAGTAAGTACAGCGCATGTATGTGATCCTGTCGTCGTGCTTGTTCATGCGCTCGGGCGGGATCGTCATCCATCAGAGAAGGCGACCTTCGGGGAGCACGAGTGGTACTTCTTCAGCCCGCGCGACCGCAAGTACCCCAACGGCGCGCGGCCGAACCGGGCGGCGACGTCGGGCTACTGGAAGGCCACCGGCACGGACAAGCCTATCCTGGCCTCGGCCACCGGGTGCGGCCGGGAGAAGGTCGGCGTCAAGAAGGCGCTCGTCTTCTACCGCGGGAAGCCGCCCAGGGGCCTCAAGACCAACTGGATCATGCATGAGTACCGCCTCACCGGAGCCTCTGCTGGCTCCACCACCACCAGCCGGCCGCCGCCGGTGACCGGCGGGAGCAGGGCCCCGGCCTCTCTCAGGGTACGTACTTACACGTGTCCATCGCACGGTCTATCAGTATTTATTTATTAACTACTCTCGAGCTTAATTATGGTATTGTTGATAGTTGATGAAGTTAATTATTGTACGCCGTCTCATCGATCAGTTGGACGACTGGGTGCTGTGCCGCATCTACAAGAAGACCAGCAAGGCCGCGGCCGCGGTCGGAGATGAGCAGAGGAGCATGGAGTGCGAGGACTCCGTGGAGGACGCGGTCACCGCGTACCCGCCCTACGCCACGGCGGGCATGGCCGGCGCAGGTGCGCATGGCAGCAACTACGTTCAACTGCTCCATCATCACGACAGCCACGAGGACAACTTCCAGCTAGACGGCCTGCTCACAGAACACGACGTCGGCCTCTCGGCGGGCGCCGCCTCGCTGGGCCACCTTGCCGCGGCGGCGAGGGCCACCAAACAGTTCCTCGCCCCGTCGTCCTCAACCCCGTTCAACTGGCTCGAGGCGTCAACCGG

>HS74_*NAM-1*

GCTTTTTATTATACTGTGCACAAGTATTTTTATATTCTTCCAGTAAGTACAGCGCATGTATGTGATCCTGTCGTCGTGCTTGTTCATGCGCTCGGGCGGGATCATCATCCATCAGAGAAGGCGACCTTCGGGGAGCACGAGTGGTACTTCTTCAGCCCGCGCGACCGCAAGTACCCCAACGGCGCGCGGCCGAACCGGGCGGCGACGTCGGGCTACTGGAAGGCCACCGGCACGGACAAGCCTATCCTGGCCTCGGCCACCGGGTGCGGCCGGGAGAAGGTCGGCGTCAAGAAGGCGCTCGTCTTCTACCGCGGGAAGCCGCCCAGGGGCCTCAAGACCAACTGGATCATGCATGAGTACCGCCTCACCGGAGCCTCTGCTGGCTCCACCACCACCAGCCGGCCGCCGCCGGTGACCGGCGGGAGCAGGGCCCCGGCCTCTCTCAGGGTACGTACTTACACGTGTCCATCGCACGGTCTATCAGTATTTATTTATTAACTACTCTCGAGCTTAATTATGGTATTGTTGATAGTTGATGAAGTTAATTATTGTACGCCGTCTCATCGATCAGTTGGACGACTGGGTGCTGTGCCGCATCTACAAGAAGACCAGCAAGGCCGCGGCCGCGGTCGGAGATGAGCAGAGGAGCATGGAGTGCGAGGACTCCGTGGAGGACGCGGTCACCGCGTACCCGCCCTACGCCACGGCGGGCATGGCCGGCGCAGGTGCGCATGGCAGCAACTACGTTCAACTGCTCCATCATCACGACAGCCACGAGGACAACTTCCAGCTAGACGGCCTGCTCACAGAACACGACGTCGGCCTCTCGGCGGGCGCCGCCTCGCTGGGCCACCTTGCCGCGGCGGCGAGGGCCACCAAACAGTTCCTCGCCCCGTCGTCCTCAACCCCGTTCAACTGGCTCGAGGCGTCAACCGG

>HS75_*NAM-1*

GCTTTTTATTATACTGTGCACAAGTATTTTTATATTCTTCCAGTAAGTACAGCGCATGTATGTGATCCTGTCGTCGTGCTTGTTCATGCGCTCGGGCGGGATCGTCATCCATCAGAGAAGGCGACCTTCGGGGAGCACGAGTGGTACTTCTTCAGCCCGCGCGACCGCAAGTACCCCAACGGCGCGCGGCCGAACCGGGCGGCGACGTCGGGCTACTGGAAGGCCACCGGCACGGACAAGCCTATCCTGGCCTCGGCCACCGGGTGCGGCCGGGAGAAGGTCGGCGTCAAGAAGGCGCTCGTCTTCTACCGCGGGAAGCCGCCCAGGGGCCTCAAGACCAACTGGATCATGCATGAGTACCGCCTCACCGGAGCCTCTGCTGGCTCCACCACCACCAGCCGGCCGCCGCCGGTGACCGGCGGGAGCAGGGCCCCGGCCTCTCTCAGGGTACGTACTTACACGTGTCCATCGCACGGTCTATCAGTATTTATTTATTAACTACTCTCGAGCTTAATTATGGTATTGTTGATAGTTGATGAAGTTAATTATTGTACGCCGTCTCATCGATCAGTTGGACGACTGGGTGCTGTGCCGCATCTACAAGAAGACCAGCAAGGCCGCGGCCGCGGTCGGAGATGAGCAGAGGAGCATGGAGTGCGAGGACTCCGTGGAGGACGCGGTCACCGCGTACCCGCCCTACGCCACGGCGGGCATGGCCGGCGCAGGTGCGCATGGCAGCAACTACGTTCAACTGCTCCATCATCACGACAGCCACGAGGACAACTTCCAGCTAGACGGCCTGCTCACAGAACACGACGTCGGCCTCTCGGCGGGCGCCGCCTCGCTGGGCCACCTTGCCGCGGCGGCGAGGGCCACCAAACAGTTCCTCGCCCCGTCGTCCTCAACCCCGTTCAACTGGCTCGAGGCGTCAACCGG

>HS76_*NAM-1*

GCTTTTTATTATACTGTGCACAAGTATTTTTATATTCTTCCAGTAAGTACAGCGCATGTATGTGATCCTGTCGTCGTGCTTGTTCATGCGCTCGGGCGGGATCGTCATCCATCAGAGAAGGCGACCTTCGGGGAGCACGAGTGGTACTTCTTCAGCCCGCGCGACCGCAAGTACCCCAACGGCGCGCGGCCGAACCGGGCGGCGACGTCGGGCTACTGGAAGGCCACCGGCACGGACAAGCCTATCCTGGCCTCGGCCACCGGGTGCGGCCGGGAGAAGGTCGGCGTCAAGAAGGCGCTCGTCTTCTACCGCGGGAAGCCGCCCAGGGGCCTCAAGACCAACTGGATCATGCATGAGTACCGCCTCACCGGAGCCTCTGCTGGCTCCACCACCACCAGCCGGCCGCCGCCGGTGACCGGCGGGAGCAGGGCCCCGGCCTCTCTCAGGGTACGTACTTACACGTGTCCATCGCACGGTCTATCAGTATTTATTTATTAACTACTCTCGAGCTTAATTATGGTATTGTTGATAGTTGATGAAGTTAATTATTGTACGCCGTCTCATCGATCAGTTGGACGACTGGGTGCTGTGCCGCATCTACAAGAAGACCAGCAAGGCCGCGGCCGCGGTCGGAGATGAGCAGAGGAGCATGGAGTGCGAGGACTCCGTGGAGGACGCGGTCACCGCGTACCCGCCCTACGCCACGGCGGGCATGGCCGGCGCAGGTGCGCATGGCAGCAACTACGTTCAACTGCTCCATCATCACGACAGCCACGAGGACAACTTCCAGCTAGACGGCCTGCTCACAGAACACGACGTCGGCCTCTCGGCGGGCGCCGCCTCGCTGGGCCACCTTGCCGCGGCGGCGAGGGCCACCAAACAGTTCCTCGCCCCGTCGTCCTCAACCCCGTTCAACTGGCTCGAGGCGTCAACCGG

>HS77_*NAM-1*

GCTTTTTATTATACTGTGCACAAGTATTTTTATATTCTTCCAGTAAGTACAGCGCATGTATGTGATCCTGTCGTCGTGCTTGTTCATGCGCTCGGGCGGGATCATCATCCATCAGAGAAGGCGACCTTCGGGGAGCACGAGTGGTACTTCTTCAGCCCGCGCGACCGCAAGTACCCCAACGGCGCGCGGCCGAACCGGGCGGCGACGTCGGGCTACTGGAAGGCCACCGGCACGGACAAGCCTATCATGGCCTCGGCCACCGGGTGCGGCCGGGAGAAGGTCGGCGTCAAGAAGGCGCTCGTCTTCTACCGCGGGAAGCCGCCCAGGGGCCTCAAGACCAACTGGATCATGCATGAGTACCGCCTCACCGGAGCCTCTGCTGGCTCCACCACCACCAGCCGGCCGCCGCCGGTGACCGGCGGGAGCAGGGCCCCGGCCTCTCTCAGGGTACGTACTTACACGTGTCCATCGCACGGTCTATCAGTATTTATTTATTAACTACTCTCGAGCTTAATTATGGTATTGTTGATAGTTGATGAAGTTAATTATTGTACGCCGTCTCATCGATCAGTTGGACGACTGGGTGCTGTGCCGCATCTACAAGAAGACCAGCAAGGCCGCGGCCGCGGTCGGAGATGAGCAGAGGAGCATGGAGTGCGAGGACTCCGTGGAGGACGCGGTCACCGCGTACCCGCCCTACGCCACGGCGGGCATGGCCGGCGCAGGTGCGCATGGCAGCAACTACGTTCAACTGCTCCATCATCACGACAGCCACGAGGACAACTTCCAGCTAGACGGCCTGCTCACAGAACACGACGTCGGCCTCTCGGCGGGCGCCGCCTCGCTGGGCCACCTTGCCGCGGCGGCGAGGGCCACCAAACAGTTCCTCGCCCCGTCGTCCTCAACCCCGTTCAACTGGCTCGAGGCGTCAACCGG

>HS78_*NAM-1*

GCTTTTTATTATACTGTGCACAAGTATTTTTATATTCTTCCAGTAAGTACAGCGCATGTATGTGATCCTGTCGTCGTGCTTGTTCATGCGCTCGGGCGGGATCATCATCCATCAGAGAAGGCGACCTTCGGGGAGCACGAGTGGTACTTCTTCAGCCCGCGCGACCGCAAGTACCCCAACGGCGCGCGGCCGAACCGGGCGGCGACGTCGGGCTACTGGAAGGCCACCGGCACGGACAAGCCTATCCTGGCCTCGGCCACCGGGTGCGGCCGGGAGAAGGTCGGCGTCAAGAAGGCGCTCGTCTTCTACCGCGGGAAGCCGCCCAGGGGCCTCAAGACCAACTGGATCATGCATGAGTACCGCCTCACCGGAGCCTCTGCTGGCTCCACCACCACCAGCCGGCCGCCGCCGGTGACCGGCGGGAGCAGGGCCCCGGCCTCTCTCAGGGTACGTACTTACACGTGTCCATCGCACGGTCTATCAGTATTTATTTATTAACTACTCTCGAGCTTAATTATGGTATTGTTGATAGTTGATGAAGTTAATTATTGTACGCCGTCTCATCGATCAGTTGGACGACTGGGTGCTGTGCCGCATCTACAAGAAGACCAGCAAGGCCGCGGCCGCGGTCGGAGATGAGCAGAGGAGCATGGAGTGCGAGGACTCCGTGGAGGACGCGGTCACCGCGTACCCGCCCTACGCCACGGCGGGCATGGCCGGCGCAGGTGCGCATGGCAGCAACTACGTTCAACTGCTCCATCATCACGACAGCCACGAGGACAACTTCCAGCTAGACGGCCTGCTCACAGAACACGACGTCGGCCTCTCGGCGGGCGCCGCCTCGCTGGGCCACCTTGCCGCGGCGGCGAGGGCCACCAAACAGTTCCTCGCCCCGTCGTCCTCAACCCCGTTCAACTGGCTCGAGGCGTCAACCGG

>HS79_*NAM-1*

GCTTTTTATTATACTGTGCACAAGTATTTTTATATTCTTCCAGTAAGTACAGCGCATGTATGTGATCCTGTCGTCGTGCTTGTTCATGCGCTCGGGCGGGATCGTCATCCATCAGAGAAGGCGACCTTCGGGGAGCACGAGTGGTACTTCTTCAGCCCGCGCGACCGCAAGTACCCCAACGGCGCGCGGCCGAACCGGGCGGCGACGTCGGGCTACTGGAAGGCCACCGGCACGGACAAGCCTATCCTGGCCTCGGCCACCGGGTGCGGCCGGGAGAAGGTCGGCGTCAAGAAGGCGCTCGTCTTCTACCGCGGGAAGCCGCCCAGGGGCCTCAAGACCAACTGGATCATGCATGAGTACCGCCTCACCGGAGCCTCTGCTGGCTCCACCACCACCAGCCGGCCGCCGCCGGTGACCGGCGGGAGCAGGGCCCCGGCCTCTCTCAGGGTACGTACTTACACGTGTCCATCGCACGGTCTATCAGTATTTATTTATTAACTACTCTCGAGCTTAATTATGGTATTGTTGATAGTTGATGAAGTTAATTATTGTACGCCGTCTCATCGATCAGTTGGACGACTGGGTGCTGTGCCGCATCTACAAGAAGACCAGCAAGGCCGCGGCCGCGGTCGGAGATGAGCAGAGGAGCATGGAGTGCGAGGACTCCGTGGAGGACGCGGTCACCGCGTACCCGCCCTACGCCACGGCGGGCATGGCCGGCGCAGGTGCGCATGGCAGCAACTACGTTCAACTGCTCCATCATCACGACAGCCACGAGGACAACTTCCAGCTAGACGGCCTGCTCACAGAACACGACGTCGGCCTCTCGGCGGGCGCCGCCTCGCTGGGCCACCTTGCCGCGGCGGCGAGGGCCACCAAACAGTTCCTCGCCCCGTCGTCCTCAACCCCGTTCAACTGGCTCGAGGCGTCAACCGG

>HS80_*NAM-1*

GCTTTTTATTATACTGTGCACAAGTATTTTTATATTCTTCCAGTAAGTACAGCGCATGTATGTGATCCTGTCGTCGTGCTTGTTCATGCGCTCGGGCGGGATCATCATCCATCAGAGAAGGCGACCTTCGGGGAGCACGAGTGGTACTTCTTCAGCCCGCGCGACCGCAAGTACGCCAACGGCGCGCGGCCGAACCGGGCGGCGACGTCGGGCTACTGGAAGGCCACCGGCACGGACAAGCCTATCCTGGCCTCGGCCACCGGGTGCGGCCGGGAGAAGGTCGGCGTCAAGAAGGCGCTCGTCTTCTACCGCGGGAAGCCGCCCAGGGGCCTCAAGACCAACTGGATCATGCATGAGTACCGCCTCACCGGAGCCTCTGCTGGCTCCACCACCACCAGCCGGCCGCCGCCGGTGACCGGCGGGAGCAGGGCCCCGGCCTCTCTCAGGGTACGTACTTACACGTGTCCATCGCACGGTCTATCAGTATTTATTTATTAACTACTCTCGAGCTTAATTATGGTATTGTTGATAGTTGATGAAGTTAATTATTGTACGCCGTCTCATCGATCAGTTGGACGACTGGGTGCTGTGCCGCATCTACAAGAAGACCAGCAAGGCCGCGGCCGCGGTCGGAGATGAGCAGAGGAGCATGGAGTGCGAGGACTCCGTGGAGGACGCGGTCACCGCGTACCCGCCCTACGCCACGGCGGGCATGGCCGGCGCAGGTGCGCATGGCAGCAACTACGTTCAACTGCTCCATCATCACGACAGCCACGAGGACAACTTCCAGCTAGACGGCCTGCTCACAGAACACGACGTCGGCCTCTCGGCGGGCGCCGCCTCGCTGGGCCACCTTGCCGCGGCGGCGAGGGCCACCAAACAGTTCCTCGCCCCGTCGTCCTCAACCCCGTTCAACTGGCTCGAGGCGTCAACCGG

>HS81_*NAM-1*

GCTTTTTATTATACTGTGCACAAGTATTTTTATATTCTTCCAGTAAGTACAGCGCATGTATGTGATCCTGTCGTCGTGCTTGTTCATGCGCTCGGGCGGGATCATCATCCATCAGAGAAGGCGACCTTCGGGGAGCACGAGTGGTACTTCTTCAGCCCGCGCGACCGCAAGTACCCCAACGGCGCGCGGCCGAACCGGGCGGCGACGTCGGGCTACTGGAAGGCCACCGGCACGGACAAGCCTATCCTGGCCTCGGCCACCGGGTGCGGCCGGGAGAAGGTCGGCGTCAAGAAGGCGCTCGTCTTCTACCGCGGGAAGCCGCCCAGGGGCCTCAAGACCAACTGGATCATGCATGAGTACCGCCTCACCGGAGCCTCTGCTGGCTCCACCACCACCAGCCGGCCGCCGCCGGTGACCGGCGGGAGCAGGGCCCCGGCCTCTCTCAGGGTACGTACTTACACGTGTCCATCGCACGGTCTATCAGTATTTATTTATTAACTACTCTCGAGCTTAATTATGGTATTGTTGATAGTTGATGAAGTTAATTATTGTACGCCGTCTCATCGATCAGTTGGACGACTGGGTGCTGTGCCGCATCTACAAGAAGACCAGCAAGGCCGCGGCCGCGGTCGGAGATGAGCAGAGGAGCATGGAGTGCGAGGACTCCGTGGAGGACGCGGTCACCGCGTACCCGCCCTACGCCACGGCGGGCATGGCCGGCGCAGGTGCGCATGGCAGCAACTACGTTCAACTGCTCCATCATCACGACAGCCACGAGGACAACTTCCAGCTAGACGGCCTGCTCACAGAACACGACGTCGGCCTCTCGGCGGGCGCCGCCTCGCTGGGCCACCTTGCCGCGGCGGCGAGGGCCACCAAACAGTTCCTCGCCCCGTCGTCCTCAACCCCGTTCAACTGGCTCGAGGCGTCAACCGG

>HS82_*NAM-1*

GCTTTTTATTATACTGTGCACAAGTATTTTTATATTCTTCCAGTAAGTACAGCGCATGTATGTGATCCTGTCGTCGTGCTTGTTCATGCGCTCGGGCGGGATCATCATCCATCAGAGAAGGCGACCTTCGGGGAGCACGAGTGGTACTTCTTCAGCCCGCGCGACCGCAAGTACCCCAACGGCGCGCGGCCGAACCGGGCGGCGACGTCGGGCTACTGGAAGGCCACCGGCACGGACAAGCCTATCCTGGCCTCGGCCACCGGGTGCGGCCGGGAGAAGGTCGGCGTCAAGAAGGCGCTCGTCTTCTACCGCGGGAAGCCGCCCAGGGGCCTCAAGACCAACTGGATCATGCATGAGTACCGCCTCACCGGAGCCTCTGCTGGCTCCACCACCACCAGCCGGCCGCCGCCGGTGACCGGCGGGAGCAGGGCCCCGGCCTCTCTCAGGGTACGTACTTACACGTGTCCATCGCACGGTCTATCAGTATTTATTTATTAACTACTCTCGAGCTTAATTATGGTATTGTTGATAGTTGATGAAGTTAATTATTGTACGCCGTCTCATCGATCAGTTGGACGACTGGGTGCTGTGCCGCATCTACAAGAAGACCAGCAAGGCCGCGGCCGCGGTCGGAGATGAGCAGAGGAGCATGGAGTGCGAGGACTCCGTGGAGGACGCGGTCACCGCGTACCCGCCCTACGCCACGGCGGGCATGGCCGGCGCAGGTGCGCATGGCAGCAACTACGTTCAACTGCTCCATCATCACGACAGCCACGAGGACAACTTCCAGCTAGACGGCCTGCTCACAGAACACGACGTCGGCCTCTCGGCGGGCGCCGCCTCGCTGGGCCACCTTGCCGCGGCGGCGAGGGCCACCAAACAGTTCCTCGCCCCGTCGTCCTCAACCCCGTTCAACTGGCTCGAGGCGTCAACCGG

>HS83_*NAM-1*

GCTTTTTATTATACTGTGCACAAGTATTTTTATATTCTTCCAGTAAGTACAGCGCATGTATGTGATCCTGTCGTCGTGCTTGTTCATGCGCTCGGGCGGGATCATCATCCATCAGAGAAGGCGACCTTCGGGGAGCACGAGTGGTACTTCTTCAGCCCGCGCGACCGCAAGTACCCCAACGGCGCGCGGCCGAACCGGGCGGCGACGTCGGGCTACTGGAAGGCCACCGGCACGGACAAGCCTATCCTGGCCTCGGCCACCGGGTGCGGCCGGGAGAAGGTCGGCGTCAAGAAGGCGCTCGTCTTCTACCGCGGGAAGCCGCCCAGGGGCCTCAAGACCAACTGGATCATGCATGAGTACCGCCTCACCGGAGCCTCTGCTGGCTCCACCACCACCAGCCGGCCGCCGCCGGTGACCGGCGGGAGCAGGGCCCCGGCCTCTCTCAGGGTACGTACTTACACGTGTCCATCGCACGGTCTATCAGTATTTATTTATTAACTACTCTCGAGCTTAATTATGGTATTGTTGATAGTTGATGAAGTTAATTATTGTACGCCGTCTCATCGATCAGTTGGACGACTGGGTGCTGTGCCGCATCTACAAGAAGACCAGCAAGGCCGCGGCCGCGGTCGGAGATGAGCAGAGGAGCATGGAGTGCGAGGACTCCGTGGAGGACGCGGTCACCGCGTACCCGCCCTACGCCACGGCGGGCATGGCCGGCGCAGGTGCGCATGGCAGCAACTACGTTCAACTGCTCCATCATCACGACAGCCACGAGGACAACTTCCAGCTAGACGGCCTGCTCACAGAACACGACGTCGGCCTCTCGGCGGGCGCCGCCTCGCTGGGCCACCTTGCCGCGGCGGCGAGGGCCACCAAACAGTTCCTCGCCCCGTCGTCCTCAACCCCGTTCAACTGGCTCGAGGCGTCAACCGG

>HS84_*NAM-1*

GCTTTTTATTATACTGTGCACAAGTATTTTTATATTCTTCCAGTAAGTACAGCGCATGTATGTGATCCTGTCGTCGTGCTTGTTCATGCGCTCGGGCGGGATCATCATCCATCAGAGAAGGCGACCTTCGGGGAGCACGAGTGGTACTTCTTCAGCCCGCGCGACCGCAAGTACCCCAACGGCGCGCGGCCGAACCGGGCGGCGACGTCGGGCTACTGGAAGGCCACCGGCACGGACAAGCCTATCCTGGCCTCGGCCACCGGGTGCGGCCGGGAGAAGGTCGGCGTCAAGAAGGCGCTCGTCTTCTACCGCGGGAAGCCGCCCAGGGGCCTCAAGACCAACTGGATCATGCATGAGTACCGCCTCACCGGAGCCTCTGCTGGCTCCACCACCACCAGCCGGCCGCCGCCGGTGACCGGCGGGAGCAGGGCCCCGGCCTCTCTCAGGGTACGTACTTACACGTGTCCATCGCACGGTCTATCAGTATTTATTTATTAACTACTCTCGAGCTTAATTATGGTATTGTTGATAGTTGATGAAGTTAATTATTGTACGCCGTCTCATCGATCAGTTGGACGACTGGGTGCTGTGCCGCATCTACAAGAAGACCAGCAAGGCCGCGGCCGCGGTCGGAGATGAGCAGAGGAGCATGGAGTGCGAGGACTCCGTGGAGGACGCGGTCACCGCGTACCCGCCCTACGCCACGGCGGGCATGGCCGGCGCAGGTGCGCATGGCAGCAACTACGTTCAACTGCTCCATCATCACGACAGCCACGAGGACAACTTCCAGCTAGACGGCCTGCTCACAGAACACGACGTCGGCCTCTCGGCGGGCGCCGCCTCGCTGGGCCACCTTGCCGCGGCGGCGAGGGCCACCAAACAGTTCCTCGCCCCGTCGTCCTCAACCCCGTTCAACTGGCTCGAGGCGTCAACCGG

>HS85_*NAM-1*

GCTTTTTATTATACTGTGCACAAGTATTTTTATATTCTTCCAGTAAGTACAGCGCATGTATGTGATCCTGTCGTCGTGCTTGTTCATGCGCTCGGGCGGGATCATCATCCATCAGAGAAGGCGACCTTCGGGGAGCACGAGTGGTACTTCTTCAGCCCGCGCGACCGCAAGTACCCCAACGGCGCGCGGCCGAACCGGGCGGCGACGTCGGGCTACTGGAAGGCCACCGGCACGGACAAGCCTATCCTGGCCTCGGCCACCGGGTGCGGCCGGGAGAAGGTCGGCGTCAAGAAGGCGCTCGTCTTCTACCGCGGGAAGCCGCCCAGGGGCCTCAAGACCAACTGGATCATGCATGAGTACCGCCTCACCGGAGCCTCTGCTGGCTCCACCACCACCAGCCGGCCGCCGCCGGTGACCGGCGGGAGCAGGGCCCCGGCCTCTCTCAGGGTACGTACTTACACGTGTCCATCGCACGGTCTATCAGTATTTATTTATTAACTACTCTCGAGCTTAATTATGGTATTGTTGATAGTTGATGAAGTTAATTATTGTACGCCGTCTCATCGATCAGTTGGACGACTGGGTGCTGTGCCGCATCTACAAGAAGACCAGCAAGGCCGCGGCCGCGGTCGGAGATGAGCAGAGGAGCATGGAGTGCGAGGACTCCGTGGAGGACGCGGTCACCGCGTACCCGCCCTACGCCACGGCGGGCATGGCCGGCGCAGGTGCGCATGGCAGCAACTACGTTCAACTGCTCCATCATCACGACAGCCACGAGGACAACTTCCAGCTAGACGGCCTGCTCACAGAACACGACGTCGGCCTCTCGGCGGGCGCCGCCTCGCTGGGCCACCTTGCCGCGGCGGCGAGGGCCACCAAACAGTTCCTCGCCCCGTCGTCCTCAACCCCGTTCAACTGGCTCGAGGCGTCAACCGG

>HS86_*NAM-1*

GCTTTTTATTATACTGTGCACAAGTATTTTTATATTCTTCCAGTAAGTACAGCGCATGTATGTGATCCTGTCGTCGTGCTTGTTCATGCGCTCGGGCGGGATCATCATCCATCAGAGAAGGCGACCTTCGGGGAGCACGAGTGGTACTTCTTCAGCCCGCGCGACCGCAAGTACGCCAACGGCGCGCGGCCGAACCGGGCGGCGACGTCGGGCTACTGGAAGGCCACCGGCACGGACAAGCCTATCCTGGCCTCGGCCACCGGGTGCGGCCGGGAGAAGGTCGGCGTCAAGAAGGCGCTCGTCTTCTACCGCGGGAAGCCGCCCAGGGGCCTCAAGACCAACTGGATCATGCATGAGTACCGCCTCACCGGAGCCTCTGCTGGCTCCACCACCACCAGCCGGCCGCCGCCGGTGACCGGCGGGAGCAGGGCCCCGGCCTCTCTCAGGGTACGTACTTACACGTGTCCATCGCACGGTCTATCAGTATTTATTTATTAACTACTCTCGAGCTTAATTATGGTATTGTTGATAGTTGATGAAGTTAATTATTGTACGCCGTCTCATCGATCAGTTGGACGACTGGGTGCTGTGCCGCATCTACAAGAAGACCAGCAAGGCCGCGGCCGCGGTCGGAGATGAGCAGAGGAGCATGGAGTGCGAGGACTCCGTGGAGGACGCGGTCACCGCGTACCCGCCCTACGCCACGGCGGGCATGGCCGGCGCAGGTGCGCATGGCAGCAACTACGTTCAACTGCTCCATCATCACGACAGCCACGAGGACAACTTCCAGCTAGACGGCCTGCTCACAGAACACGACGTCGGCCTCTCGGCGGGCGCCGCCTCGCTGGGCCACCTTGCCGCGGCGGCGAGGGCCACCAAACAGTTCCTCGCCCCGTCGTCCTCAACCCCGTTCAACTGGCTCGAGGCGTCAACCGG

>HS87_*NAM-1*

GCTTTTTATTATACTGTGCACAAGTATTTTTATATTCTTCCAGTAAGTACAGCGCATGTATGTGATCCTGTCGTCGTGCTTGTTCATGCGCTCGGGCGGGATCATCATCCATCAGAGAAGGCGACCTTCGGGGAGCACGAGTGGTACTTCTTCAGCCCGCGCGACCGCAAGTACCCCAACGGCGCGCGGCCGAACCGGGCGGCGACGTCGGGCTACTGGAAGGCCACCGGCACGGACAAGCCTATCCTGGCCTCGGCCACCGGGTGCGGCCGGGAGAAGGTCGGCGTCAAGAAGGCGCTCGTCTTCTACCGCGGGAAGCCGCCCAGGGGCCTCAAGACCAACTGGATCATGCATGAGTACCGCCTCACCGGAGCCTCTGCTGGCTCCACCACCACCAGCCGGCCGCCGCCGGTGACCGGCGGGAGCAGGGCCCCGGCCTCTCTCAGGGTACGTACTTACACGTGTCCATCGCACGGTCTATCAGTATTTATTTATTAACTACTCTCGAGCTTAATTATGGTATTGTTGATAGTTGATGAAGTTAATTATTGTACGCCGTCTCATCGATCAGTTGGACGACTGGGTGCTGTGCCGCATCTACAAGAAGACCAGCAAGGCCGCGGCCGCGGTCGGAGATGAGCAGAGGAGCATGGAGTGCGAGGACTCCGTGGAGGACGCGGTCACCGCGTACCCGCCCTACGCCACGGCGGGCATGGCCGGCGCAGGTGCGCATGGCAGCAACTACGTTCAACTGCTCCATCATCACGACAGCCACGAGGACAACTTCCAGCTAGACGGCCTGCTCACAGAACACGACGTCGGCCTCTCGGCGGGCGCCGCCTCGCTGGGCCACCTTGCCGCGGCGGCGAGGGCCACCAAACAGTTCCTCGCCCCGTCGTCCTCAACCCCGTTCAACTGGCTCGAGGCGTCAACCGG

>HS88_*NAM-1*

GCTTTTTATTATACTGTGCACAAGTATTTTTATATTCTTCCAGTAAGTACAGCGCATGTATGTGATCCTGTCGTCGTGCTTGTTCATGCGCTCGGGCGGGATCATCATCCATCAGAGAAGGCGACCTTCGGGGAGCACGAGTGGTACTTCTTCAGCCCGCGCGACCGCAAGTACCCCAACGGCGCGCGGCCGAACCGGGCGGCGACGTCGGGCTACTGGAAGGCCACCGGCACGGACAAGCCTATCCTGGCCTCGGCCACCGGGTGCGGCCGGGAGAAGGTCGGCGTCAAGAAGGCGCTCGTCTTCTACCGCGGGAAGCCGCCCAGGGGCCTCAAGACCAACTGGATCATGCATGAGTACCGCCTCACCGGAGCCTCTGCTGGCTCCACCACCACCAGCCGGCCGCCGCCGGTGACCGGCGGGAGCAGGGCCCCGGCCTCTCTCAGGGTACGTACTTACACGTGTCCATCGCACGGTCTATCAGTATTTATTTATTAACTACTCTCGAGCTTAATTATGGTATTGTTGATAGTTGATGAAGTTAATTATTGTACGCCGTCTCATCGATCAGTTGGACGACTGGGTGCTGTGCCGCATCTACAAGAAGACCAGCAAGGCCGCGGCCGCGGTCGGAGATGAGCAGAGGAGCATGGAGTGCGAGGACTCCGTGGAGGACGCGGTCACCGCGTACCCGCCCTACGCCACGGCGGGCATGGCCGGCGCAGGTGCGCATGGCAGCAACTACGTTCAACTGCTCCATCATCACGACAGCCACGAGGACAACTTCCAGCTAGACGGCCTGCTCACAGAACACGACGTCGGCCTCTCGGCGGGCGCCGCCTCGCTGGGCCACCTTGCCGCGGCGGCGAGGGCCACCAAACAGTTCCTCGCCCCGTCGTCCTCAACCCCGTTCAACTGGCTCGAGGCGTCAACCGG

>HS89_*NAM-1*

GCTTTTTATTATACTGTGCACAAGTATTTTTATATTCTTCCAGTAAGTACAGCGCATGTATGTGATCCTGTCGTCGTGCTTGTTCATGCGCTCGGGCGGGATCATCATCCATCAGAGAAGGCGACCTTCGGGGAGCACGAGTGGTACTTCTTCAGCCCGCGCGACCGCAAGTACCCCAACGGCGCGCGGCCGAACCGGGCGGCGACGTCGGGCTACTGGAAGGCCACCGGCACGGACAAGCCTATCCTGGCCTCGGCCACCGGGTGCGGCCGGGAGAAGGTCGGCGTCAAGAAGGCGCTCGTCTTCTACCGCGGGAAGCCGCCCAGGGGCCTCAAGACCAACTGGATCATGCATGAGTACCGCCTCACCGGAGCCTCTGCTGGCTCCACCACCACCAGCCGGCCGCCGCCGGTGACCGGCGGGAGCAGGGCCCCGGCCTCTCTCAGGGTACGTACTTACACGTGTCCATCGCACGGTCTATCAGTATTTATTTATTAACTACTCTCGAGCTTAATTATGGTATTGTTGATAGTTGATGAAGTTAATTATTGTACGCCGTCTCATCGATCAGTTGGACGACTGGGTGCTGTGCCGCATCTACAAGAAGACCAGCAAGGCCGCGGCCGCGGTCGGAGATGAGCAGAGGAGCATGGAGTGCGAGGACTCCGTGGAGGACGCGGTCACCGCGTACCCGCCCTACGCCACGGCGGGCATGGCCGGCGCAGGTGCGCATGGCAGCAACTACGTTCAACTGCTCCATCATCACGACAGCCACGAGGACAACTTCCAGCTAGACGGCCTGCTCACAGAACACGACGTCGGCCTCTCGGCGGGCGCCGCCTCGCTGGGCCACCTTGCCGCGGCGGCGAGGGCCACCAAACAGTTCCTCGCCCCGTCGTCCTCAACCCCGTTCAACTGGCTCGAGGCGTCAACCGG

>HS90_*NAM-1*

GCTTTTTATTATACTGTGCACAAGTATTTTTATATTCTTCCAGTAAGTACAGCGCATGTATGTGATCCTGTCGTCGTGCTTGTTCATGCGCTCGGGCGGGATCATCATCCATCAGAGAAGGCGACCTTCGGGGAGCACGAGTGGTACTTCTTCAGCCCGCGCGACCGCAAGTACCCCAACGGCGCGCGGCCGAACCGGGCGGCGACGTCGGGCTACTGGAAGGCCACCGGCACGGACAAGCCTATCCTGGCCTCGGCCACCGGGTGCGGCCGGGAGAAGGTCGGCGTCAAGAAGGCGCTCGTCTTCTACCGCGGGAAGCCGCCCAGGGGCCTCAAGACCAACTGGATCATGCATGAGTACCGCCTCACCGGAGCCTCTGCTGGCTCCACCACCACCAGCCGGCCGCCGCCGGTGACCGGCGGGAGCAGGGCCCCGGCCTCTCTCAGGGTACGTACTTACACGTGTCCATCGCACGGTCTATCAGTATTTATTTATTAACTACTCTCGAGCTTAATTATGGTATTGTTGATAGTTGATGAAGTTAATTATTGTACGCCGTCTCATCGATCAGTTGGACGACTGGGTGCTGTGCCGCATCTACAAGAAGACCAGCAAGGCCGCGGCCGCGGTCGGAGATGAGCAGAGGAGCATGGAGTGCGAGGACTCCGTGGAGGACGCGGTCACCGCGTACCCGCCCTACGCCACGGCGGGCATGGCCGGCGCAGGTGCGCATGGCAGCAACTACGTTCAACTGCTCCATCATCACGACAGCCACGAGGACAACTTCCAGCTAGACGGCCTGCTCACAGAACACGACGTCGGCCTCTCGGCGGGCGCCGCCTCGCTGGGCCACCTTGCCGCGGCGGCGAGGGCCACCAAACAGTTCCTCGCCCCGTCGTCCTCAACCCCGTTCAACTGGCTCGAGGCGTCAACCGG

>HS91_*NAM-1*

GCTTTTTATTATACTGTGCACAAGTATTTTTATATTCTTCCAGTAAGTACAGCGCATGTATGTGATCCTGTCGTCGTGCTTGTTCATGCGCTCGGGCGGGATCATCATCCATCAGAGAAGGCGACCTTCGGGGAGCACGAGTGGTACTTCTTCAGCCCGCGCGACCGCAAGTACGCCAACGGCGCGCGGCCGAACCGGGCGGCGACGTCGGGCTACTGGAAGGCCACCGGCACGGACAAGCCTATCCTGGCCTCGGCCACCGGGTGCGGCCGGGAGAAGGTCGGCGTCAAGAAGGCGCTCGTCTTCTACCGCGGGAAGCCGCCCAGGGGCCTCAAGACCAACTGGATCATGCATGAGTACCGCCTCACCGGAGCCTCTGCTGGCTCCACCACCACCAGCCGGCCGCCGCCGGTGACCGGCGGGAGCAGGGCCCCGGCCTCTCTCAGGGTACGTACTTACACGTGTCCATCGCACGGTCTATCAGTATTTATTTATTAACTACTCTCGAGCTTAATTATGGTATTGTTGATAGTTGATGAAGTTAATTATTGTACGCCGTCTCATCGATCAGTTGGACGACTGGGTGCTGTGCCGCATCTACAAGAAGACCAGCAAGGCCGCGGCCGCGGTCGGAGATGAGCAGAGGAGCATGGAGTGCGAGGACTCCGTGGAGGACGCGGTCACCGCGTACCCGCCCTACGCCACGGCGGGCATGGCCGGCGCAGGTGCGCATGGCAGCAACTACGTTCAACTGCTCCATCATCACGACAGCCACGAGGACAACTTCCAGCTAGACGGCCTGCTCACAGAACACGACGTCGGCCTCTCGGCGGGCGCCGCCTCGCTGGGCCACCTTGCCGCGGCGGCGAGGGCCACCAAACAGTTCCTCGCCCCGTCGTCCTCAACCCCGTTCAACTGGCTCGAGGCGTCAACCGG

>HS92_*NAM-1*

GCTTTTTATTATACTGTGCACAAGTATTTTTATATTCTTCCAGTAAGTACAGCGCATGTATGTGATCCTGTCGTCGTGCTTGTTCATGCGCTCGGGCGGGATCATCATCCATCAGAGAAGGCGACCTTCGGGGAGCACGAGTGGTACTTCTTCAGCCCGCGCGACCGCAAGTACGCCAACGGCGCGCGGCCGAACCGGGCGGCGACGTCGGGCTACTGGAAGGCCACCGGCACGGACAAGCCTATCCTGGCCTCGGCCACCGGGTGCGGCCGGGAGAAGGTCGGCGTCAAGAAGGCGCTCGTCTTCTACCGCGGGAAGCCGCCCAGGGGCCTCAAGACCAACTGGATCATGCATGAGTACCGCCTCACCGGAGCCTCTGCTGGCTCCACCACCACCAGCCGGCCGCCGCCGGTGACCGGCGGGAGCAGGGCCCCGGCCTCTCTCAGGGTACGTACTTACACGTGTCCATCGCACGGTCTATCAGTATTTATTTATTAACTACTCTCGAGCTTAATTATGGTATTGTTGATAGTTGATGAAGTTAATTATTGTACGCCGTCTCATCGATCAGTTGGACGACTGGGTGCTGTGCCGCATCTACAAGAAGACCAGCAAGGCCGCGGCCGCGGTCGGAGATGAGCAGAGGAGCATGGAGTGCGAGGACTCCGTGGAGGACGCGGTCACCGCGTACCCGCCCTACGCCACGGCGGGCATGGCCGGCGCAGGTGCGCATGGCAGCAACTACGTTCAACTGCTCCATCATCACGACAGCCACGAGGACAACTTCCAGCTAGACGGCCTGCTCACAGAACACGACGTCGGCCTCTCGGCGGGCGCCGCCTCGCTGGGCCACCTTGCCGCGGCGGCGAGGGCCACCAAACAGTTCCTCGCCCCGTCGTCCTCAACCCCGTTCAACTGGCTCGAGGCGTCAACCGG

>HS93_*NAM-1*

GCTTTTTATTATACTGTGCACAAGTATTTTTATATTCTTCCAGTAAGTACAGCGCATGTATGTGATCCTGTCGTCGTGCTTGTTCATGCGCTCGGGCGGGATCATCATCCATCAGAGAAGGCGACCTTCGGGGAGCACGAGTGGTACTTCTTCAGCCCGCGCGACCGCAAGTACCCCAACGGCGCGCGGCCGAACCGGGCGGCGACGTCGGGCTACTGGAAGGCCACCGGCACGGACAAGCCTATCCTGGCCTCGGCCACCGGGTGCGGCCGGGAGAAGGTCGGCGTCAAGAAGGCGCTCGTCTTCTACCGCGGGAAGCCGCCCAGGGGCCTCAAGACCAACTGGATCATGCATGAGTACCGCCTCACCGGAGCCTCTGCTGGCTCCACCACCACCAGCCGGCCGCCGCCGGTGACCGGCGGGAGCAGGGCCCCGGCCTCTCTCAGGGTACGTACTTACACGTGTCCATCGCACGGTCTATCAGTATTTATTTATTAACTACTCTCGAGCTTAATTATGGTATTGTTGATAGTTGATGAAGTTAATTATTGTACGCCGTCTCATCGATCAGTTGGACGACTGGGTGCTGTGCCGCATCTACAAGAAGACCAGCAAGGCCGCGGCCGCGGTCGGAGATGAGCAGAGGAGCATGGAGTGCGAGGACTCCGTGGAGGACGCGGTCACCGCGTACCCGCCCTACGCCACGGCGGGCATGGCCGGCGCAGGTGCGCATGGCAGCAACTACGTTCAACTGCTCCATCATCACGACAGCCACGAGGACAACTTCCAGCTAGACGGCCTGCTCACAGAACACGACGTCGGCCTCTCGGCGGGCGCCGCCTCGCTGGGCCACCTTGCCGCGGCGGCGAGGGCCACCAAACAGTTCCTCGCCCCGTCGTCCTCAACCCCGTTCAACTGGCTCGAGGCGTCAACCGG

>HS94_*NAM-1*

GCTTTTTATTATACTGTGCACAAGTATTTTTATATTCTTCCAGTAAGTACAGCGCATGTATGTGATCCTGTCGTCGTGCTTGTTCATGCGCTCGGGCGGGATCATCATCCATCAGAGAAGGCGACCTTCGGGGAGCACGAGTGGTACTTCTTCAGCCCGCGCGACCGCAAGTACGCCAACGGCGCGCGGCCGAACCGGGCGGCGACGTCGGGCTACTGGAAGGCCACCGGCACGGACAAGCCTATCCTGGCCTCGGCCACCGGGTGCGGCCGGGAGAAGGTCGGCGTCAAGAAGGCGCTCGTCTTCTACCGCGGGAAGCCGCCCAGGGGCCTCAAGACCAACTGGATCATGCATGAGTACCGCCTCACCGGAGCCTCTGCTGGCTCCACCACCACCAGCCGGCCGCCGCCGGTGACCGGCGGGAGCAGGGCCCCGGCCTCTCTCAGGGTACGTACTTACACGTGTCCATCGCACGGTCTATCAGTATTTATTTATTAACTACTCTCGAGCTTAATTATGGTATTGTTGATAGTTGATGAAGTTAATTATTGTACGCCGTCTCATCGATCAGTTGGACGACTGGGTGCTGTGCCGCATCTACAAGAAGACCAGCAAGGCCGCGGCCGCGGTCGGAGATGAGCAGAGGAGCATGGAGTGCGAGGACTCCGTGGAGGACGCGGTCACCGCGTACCCGCCCTACGCCACGGCGGGCATGGCCGGCGCAGGTGCGCATGGCAGCAACTACGTTCAACTGCTCCATCATCACGACAGCCACGAGGACAACTTCCAGCTAGACGGCCTGCTCACAGAACACGACGTCGGCCTCTCGGCGGGCGCCGCCTCGCTGGGCCACCTTGCCGCGGCGGCGAGGGCCACCAAACAGTTCCTCGCCCCGTCGTCCTCAACCCCGTTCAACTGGCTCGAGGCGTCAACCGG

>HS95_*NAM-1*

GCTTTTTATTATACTGTGCACAAGTATTTTTATATTCTTCCAGTAAGTACAGCGCATGTATGTGATCCTGTCGTCGTGCTTGTTCATGCGCTCGGGCGGGATCATCATCCATCAGAGAAGGCGACCTTCGGGGAGCACGAGTGGTACTTCTTCAGCCCGCGCGACCGCAAGTACGCCAACGGCGCGCGGCCGAACCGGGCGGCGACGTCGGGCTACTGGAAGGCCACCGGCACGGACAAGCCTATCCTGGCCTCGGCCACCGGGTGCGGCCGGGAGAAGGTCGGCGTCAAGAAGGCGCTCGTCTTCTACCGCGGGAAGCCGCCCAGGGGCCTCAAGACCAACTGGATCATGCATGAGTACCGCCTCACCGGAGCCTCTGCTGGCTCCACCACCACCAGCCGGCCGCCGCCGGTGACCGGCGGGAGCAGGGCCCCGGCCTCTCTCAGGGTACGTACTTACACGTGTCCATCGCACGGTCTATCAGTATTTATTTATTAACTACTCTCGAGCTTAATTATGGTATTGTTGATAGTTGATGAAGTTAATTATTGTACGCCGTCTCATCGATCAGTTGGACGACTGGGTGCTGTGCCGCATCTACAAGAAGACCAGCAAGGCCGCGGCCGCGGTCGGAGATGAGCAGAGGAGCATGGAGTGCGAGGACTCCGTGGAGGACGCGGTCACCGCGTACCCGCCCTACGCCACGGCGGGCATGGCCGGCGCAGGTGCGCATGGCAGCAACTACGTTCAACTGCTCCATCATCACGACAGCCACGAGGACAACTTCCAGCTAGACGGCCTGCTCACAGAACACGACGTCGGCCTCTCGGCGGGCGCCGCCTCGCTGGGCCACCTTGCCGCGGCGGCGAGGGCCACCAAACAGTTCCTCGCCCCGTCGTCCTCAACCCCGTTCAACTGGCTCGAGGCGTCAACCGG

>HS96_*NAM-1*

GCTTTTTATTATACTGTGCACAAGTATTTTTATATTCTTCCAGTAAGTACAGCGCATGTATGTGATCCTGTCGTCGTGCTTGTTCATGCGCTCGGGCGGGATCATCATCCATCAGAGAAGGCGACCTTCGGGGAGCACGAGTGGTACTTCTTCAGCCCGCGCGACCGCAAGTACGCCAACGGCGCGCGGCCGAACCGGGCGGCGACGTCGGGCTACTGGAAGGCCACCGGCACGGACAAGCCTATCCTGGCCTCGGCCACCGGGTGCGGCCGGGAGAAGGTCGGCGTCAAGAAGGCGCTCGTCTTCTACCGCGGGAAGCCGCCCAGGGGCCTCAAGACCAACTGGATCATGCATGAGTACCGCCTCACCGGAGCCTCTGCTGGCTCCACCACCACCAGCCGGCCGCCGCCGGTGACCGGCGGGAGCAGGGCCCCGGCCTCTCTCAGGGTACGTACTTACACGTGTCCATCGCACGGTCTATCAGTATTTATTTATTAACTACTCTCGAGCTTAATTATGGTATTGTTGATAGTTGATGAAGTTAATTATTGTACGCCGTCTCATCGATCAGTTGGACGACTGGGTGCTGTGCCGCATCTACAAGAAGACCAGCAAGGCCGCGGCCGCGGTCGGAGATGAGCAGAGGAGCATGGAGTGCGAGGACTCCGTGGAGGACGCGGTCACCGCGTACCCGCCCTACGCCACGGCGGGCATGGCCGGCGCAGGTGCGCATGGCAGCAACTACGTTCAACTGCTCCATCATCACGACAGCCACGAGGACAACTTCCAGCTAGACGGCCTGCTCACAGAACACGACGTCGGCCTCTCGGCGGGCGCCGCCTCGCTGGGCCACCTTGCCGCGGCGGCGAGGGCCACCAAACAGTTCCTCGCCCCGTCGTCCTCAACCCCGTTCAACTGGCTCGAGGCGTCAACCGG

>HS97_*NAM-1*

GCTTTTTATTATACTGTGCACAAGTATTTTTATATTCTTCCAGTAAGTACAGCGCATGTATGTGATCCTGTCGTCGTGCTTGTTCATGCGCTCGGGCGGGATCATCATCCATCAGAGAAGGCGACCTTCGGGGAGCACGAGTGGTACTTCTTCAGCCCGCGCGACCGCAAGTACCCCAACGGCGCGCGGCCGAACCGGGCGGCGACGTCGGGCTACTGGAAGGCCACCGGCACGGACAAGCCTATCCTGGCCTCGGCCACCGGGTGCGGCCGGGAGAAGGTCGGCGTCAAGAAGGCGCTCGTCTTCTACCGCGGGAAGCCGCCCAGGGGCCTCAAGACCAACTGGATCATGCATGAGTACCGCCTCACCGGAGCCTCTGCTGGCTCCACCACCACCAGCCGGCCGCCGCCGGTGACCGGCGGGAGCAGGGCCCCGGCCTCTCTCAGGGTACGTACTTACACGTGTCCATCGCACGGTCTATCAGTATTTATTTATTAACTACTCTCGAGCTTAATTATGGTATTGTTGATAGTTGATGAAGTTAATTATTGTACGCCGTCTCATCGATCAGTTGGACGACTGGGTGCTGTGCCGCATCTACAAGAAGACCAGCAAGGCCGCGGCCGCGGTCGGAGATGAGCAGAGGAGCATGGAGTGCGAGGACTCCGTGGAGGACGCGGTCACCGCGTACCCGCCCTACGCCACGGCGGGCATGGCCGGCGCAGGTGCGCATGGCAGCAACTACGTTCAACTGCTCCATCATCACGACAGCCACGAGGACAACTTCCAGCTAGACGGCCTGCTCACAGAACACGACGTCGGCCTCTCGGCGGGCGCCGCCTCGCTGGGCCACCTTGCCGCGGCGGCGAGGGCCACCAAACAGTTCCTCGCCCCGTCGTCCTCAACCCCGTTCAACTGGCTCGAGGCGTCAACCGG

>HS98_*NAM-1*

GCTTTTTATTATACTGTGCACAAGTATTTTTATATTCTTCCAGTAAGTACAGCGCATGTATGTGATCCTGTCGTCGTGCTTGTTCATGCGCTCGGGCGGGATCATCATCCATCAGAGAAGGCGACCTTCGGGGAGCACGAGTGGTACTTCTTCAGCCCGCGCGACCGCAAGTACCCCAACGGCGCGCGGCCGAACCGGGCGGCGACGTCGGGCTACTGGAAGGCCACCGGCACGGACAAGCCTATCCTGGCCTCGGCCACCGGGTGCGGCCGGGAGAAGGTCGGCGTCAAGAAGGCGCTCGTCTTCTACCGCGGGAAGCCGCCCAGGGGCCTCAAGACCAACTGGATCATGCATGAGTACCGCCTCACCGGAGCCTCTGCTGGCTCCACCACCACCAGCCGGCCGCCGCCGGTGACCGGCGGGAGCAGGGCCCCGGCCTCTCTCAGGGTACGTACTTACACGTGTCCATCGCACGGTCTATCAGTATTTATTTATTAACTACTCTCGAGCTTAATTATGGTATTGTTGATAGTTGATGAAGTTAATTATTGTACGCCGTCTCATCGATCAGTTGGACGACTGGGTGCTGTGCCGCATCTACAAGAAGACCAGCAAGGCCGCGGCCGCGGTCGGAGATGAGCAGAGGAGCATGGAGTGCGAGGACTCCGTGGAGGACGCGGTCACCGCGTACCCGCCCTACGCCACGGCGGGCATGGCCGGCGCAGGTGCGCATGGCAGCAACTACGTTCAACTGCTCCATCATCACGACAGCCACGAGGACAACTTCCAGCTAGACGGCCTGCTCACAGAACACGACGTCGGCCTCTCGGCGGGCGCCGCCTCGCTGGGCCACCTTGCCGCGGCGGCGAGGGCCACCAAACAGTTCCTCGCCCCGTCGTCCTCAACCCCGTTCAACTGGCTCGAGGCGTCAACCGG

>HS99_*NAM-1*

GCTTTTTATTATACTGTGCACAAGTATTTTTATATTCTTCCAGTAAGTACAGCGCATGTATGTGATCCTGTCGTCGTGCTTGTTCATGCGCTCGGGCGGGATCATCATCCATCAGAGAAGGCGACCTTCGGGGAGCACGAGTGGTACTTCTTCAGCCCGCGCGACCGCAAGTACGCCAACGGCGCGCGGCCGAACCGGGCGGCGACGTCGGGCTACTGGAAGGCCACCGGCACGGACAAGCCTATCCTGGCCTCGGCCACCGGGTGCGGCCGGGAGAAGGTCGGCGTCAAGAAGGCGCTCGTCTTCTACCGCGGGAAGCCGCCCAGGGGCCTCAAGACCAACTGGATCATGCATGAGTACCGCCTCACCGGAGCCTCTGCTGGCTCCACCACCACCAGCCGGCCGCCGCCGGTGACCGGCGGGAGCAGGGCCCCGGCCTCTCTCAGGGTACGTACTTACACGTGTCCATCGCACGGTCTATCAGTATTTATTTATTAACTACTCTCGAGCTTAATTATGGTATTGTTGATAGTTGATGAAGTTAATTATTGTACGCCGTCTCATCGATCAGTTGGACGACTGGGTGCTGTGCCGCATCTACAAGAAGACCAGCAAGGCCGCGGCCGCGGTCGGAGATGAGCAGAGGAGCATGGAGTGCGAGGACTCCGTGGAGGACGCGGTCACCGCGTACCCGCCCTACGCCACGGCGGGCATGGCCGGCGCAGGTGCGCATGGCAGCAACTACGTTCAACTGCTCCATCATCACGACAGCCACGAGGACAACTTCCAGCTAGACGGCCTGCTCACAGAACACGACGTCGGCCTCTCGGCGGGCGCCGCCTCGCTGGGCCACCTTGCCGCGGCGGCGAGGGCCACCAAACAGTTCCTCGCCCCGTCGTCCTCAACCCCGTTCAACTGGCTCGAGGCGTCAACCGG

>HS100_*NAM-1*

GCTTTTTATTATACTGTGCACAAGTATTTTTATATTCTTCCAGTAAGTACAGCGCATGTATGTGATCCTGTCGTCGTGCTTGTTCATGCGCTCGGGCGGGATCATCATCCATCAGAGAAGGCGACCTTCGGGGAGCACGAGTGGTACTTCTTCAGCCCGCGCGACCGCAAGTACCCCAACGGCGCGCGGCCGAACCGGGCGGCGACGTCGGGCTACTGGAAGGCCACCGGCACGGACAAGCCTATCCTGGCCTCGGCCACCGGGTGCGGCCGGGAGAAGGTCGGCGTCAAGAAGGCGCTCGTCTTCTACCGCGGGAAGCCGCCCAGGGGCCTCAAGACCAACTGGATCATGCATGAGTACCGCCTCACCGGAGCCTCTGCTGGCTCCACCACCACCAGCCGGCCGCCGCCGGTGACCGGCGGGAGCAGGGCCCCGGCCTCTCTCAGGGTACGTACTTACACGTGTCCATCGCACGGTCTATCAGTATTTATTTATTAACTACTCTCGAGCTTAATTATGGTATTGTTGATAGTTGATGAAGTTAATTATTGTACGCCGTCTCATCGATCAGTTGGACGACTGGGTGCTGTGCCGCATCTACAAGAAGACCAGCAAGGCCGCGGCCGCGGTCGGAGATGAGCAGAGGAGCATGGAGTGCGAGGACTCCGTGGAGGACGCGGTCACCGCGTACCCGCCCTACGCCACGGCGGGCATGGCCGGCGCAGGTGCGCATGGCAGCAACTACGTTCAACTGCTCCATCATCACGACAGCCACGAGGACAACTTCCAGCTAGACGGCCTGCTCACAGAACACGACGTCGGCCTCTCGGCGGGCGCCGCCTCGCTGGGCCACCTTGCCGCGGCGGCGAGGGCCACCAAACAGTTCCTCGCCCCGTCGTCCTCAACCCCGTTCAACTGGCTCGAGGCGTCAACCGG

>HS101_*NAM-1*

GCTTTTTATTATACTGTGCACAAGTATTTTTATATTCTTCCAGTAAGTACAGCGCATGTATGTGATCCTGTCGTCGTGCTTGTTCATGCGCTCGGGCGGGATCATCATCCATCAGAGAAGGCGACCTTCGGGGAGCACGAGTGGTACTTCTTCAGCCCGCGCGACCGCAAGTACCCCAACGGCGCGCGGCCGAACCGGGCGGCGACGTCGGGCTACTGGAAGGCCACCGGCACGGACAAGCCTATCCTGGCCTCGGCCACCGGGTGCGGCCGGGAGAAGGTCGGCGTCAAGAAGGCGCTCGTCTTCTACCGCGGGAAGCCGCCCAGGGGCCTCAAGACCAACTGGATCATGCATGAGTACCGCCTCACCGGAGCCTCTGCTGGCTCCACCACCACCAGCCGGCCGCCGCCGGTGACCGGCGGGAGCAGGGCCCCGGCCTCTCTCAGGGTACGTACTTACACGTGTCCATCGCACGGTCTATCAGTATTTATTTATTAACTACTCTCGAGCTTAATTATGGTATTGTTGATAGTTGATGAAGTTAATTATTGTACGCCGTCTCATCGATCAGTTGGACGACTGGGTGCTGTGCCGCATCTACAAGAAGACCAGCAAGGCCGCGGCCGCGGTCGGAGATGAGCAGAGGAGCATGGAGTGCGAGGACTCCGTGGAGGACGCGGTCACCGCGTACCCGCCCTACGCCACGGCGGGCATGGCCGGCGCAGGTGCGCATGGCAGCAACTACGTTCAACTGCTCCATCATCACGACAGCCACGAGGACAACTTCCAGCTAGACGGCCTGCTCACAGAACACGACGTCGGCCTCTCGGCGGGCGCCGCCTCGCTGGGCCACCTTGCCGCGGCGGCGAGGGCCACCAAACAGTTCCTCGCCCCGTCGTCCTCAACCCCGTTCAACTGGCTCGAGGCGTCAACCGG

>HS102_*NAM-1*

GCTTTTTATTATACTGTGCACAAGTATTTTTATATTCTTCCAGTAAGTACAGCGCATGTATGTGATCCTGTCGTCGTGCTTGTTCATGCGCTCGGGCGGGATCATCATCCATCAGAGAAGGCGACCTTCGGGGAGCACGAGTGGTACTTCTTCAGCCCGCGCGACCGCAAGTACGCCAACGGCGCGCGGCCGAACCGGGCGGCGACGTCGGGCTACTGGAAGGCCACCGGCACGGACAAGCCTATCCTGGCCTCGGCCACCGGGTGCGGCCGGGAGAAGGTCGGCGTCAAGAAGGCGCTCGTCTTCTACCGCGGGAAGCCGCCCAGGGGCCTCAAGACCAACTGGATCATGCATGAGTACCGCCTCACCGGAGCCTCTGCTGGCTCCACCACCACCAGCCGGCCGCCGCCGGTGACCGGCGGGAGCAGGGCCCCGGCCTCTCTCAGGGTACGTACTTACACGTGTCCATCGCACGGTCTATCAGTATTTATTTATTAACTACTCTCGAGCTTAATTATGGTATTGTTGATAGTTGATGAAGTTAATTATTGTACGCCGTCTCATCGATCAGTTGGACGACTGGGTGCTGTGCCGCATCTACAAGAAGACCAGCAAGGCCGCGGCCGCGGTCGGAGATGAGCAGAGGAGCATGGAGTGCGAGGACTCCGTGGAGGACGCGGTCACCGCGTACCCGCCCTACGCCACGGCGGGCATGGCCGGCGCAGGTGCGCATGGCAGCAACTACGTTCAACTGCTCCATCATCACGACAGCCACGAGGACAACTTCCAGCTAGACGGCCTGCTCACAGAACACGACGTCGGCCTCTCGGCGGGCGCCGCCTCGCTGGGCCACCTTGCCGCGGCGGCGAGGGCCACCAAACAGTTCCTCGCCCCGTCGTCCTCAACCCCGTTCAACTGGCTCGAGGCGTCAACCGG

>HS103_*NAM-1*

GCTTTTTATTATACTGTGCACAAGTATTTTTATATTCTTCCAGTAAGTACAGCGCATGTATGTGATCCTGTCGTCGTGCTTGTTCATGCGCTCGGGCGGGATCATCATCCATCAGAGAAGGCGACCTTCGGGGAGCACGAGTGGTACTTCTTCAGCCCGCGCGACCGCAAGTACCCCAACGGCGCGCGGCCGAACCGGGCGGCGACGTCGGGCTACTGGAAGGCCACCGGCACGGACAAGCCTATCCTGGCCTCGGCCACCGGGTGCGGCCGGGAGAAGGTCGGCGTCAAGAAGGCGCTCGTCTTCTACCGCGGGAAGCCGCCCAGGGGCCTCAAGACCAACTGGATCATGCATGAGTACCGCCTCACCGGAGCCTCTGCTGGCTCCACCACCACCAGCCGGCCGCCGCCGGTGACCGGCGGGAGCAGGGCCCCGGCCTCTCTCAGGGTACGTACTTACACGTGTCCATCGCACGGTCTATCAGTATTTATTTATTAACTACTCTCGAGCTTAATTATGGTATTGTTGATAGTTGATGAAGTTAATTATTGTACGCCGTCTCATCGATCAGTTGGACGACTGGGTGCTGTGCCGCATCTACAAGAAGACCAGCAAGGCCGCGGCCGCGGTCGGAGATGAGCAGAGGAGCATGGAGTGCGAGGACTCCGTGGAGGACGCGGTCACCGCGTACCCGCCCTACGCCACGGCGGGCATGGCCGGCGCAGGTGCGCATGGCAGCAACTACGTTCAACTGCTCCATCATCACGACAGCCACGAGGACAACTTCCAGCTAGACGGCCTGCTCACAGAACACGACGTCGGCCTCTCGGCGGGCGCCGCCTCGCTGGGCCACCTTGCCGCGGCGGCGAGGGCCACCAAACAGTTCCTCGCCCCGTCGTCCTCAACCCCGTTCAACTGGCTCGAGGCGTCAACCGG

>HS104_*NAM-1*

GCTTTTTATTATACTGTGCACAAGTATTTTTATATTCTTCCAGTAAGTACAGCGCATGTATGTGATCCTGTCGTCGTGCTTGTTCATGCGCTCGGGCGGGATCATCATCCATCAGAGAAGGCGACCTTCGGGGAGCACGAGTGGTACTTCTTCAGCCCGCGCGACCGCAAGTACCCCAACGGCGCGCGGCCGAACCGGGCGGCGACGTCGGGCTACTGGAAGGCCACCGGCACGGACAAGCCTATCCTGGCCTCGGCCACCGGGTGCGGCCGGGAGAAGGTCGGCGTCAAGAAGGCGCTCGTCTTCTACCGCGGGAAGCCGCCCAGGGGCCTCAAGACCAACTGGATCATGCATGAGTACCGCCTCACCGGAGCCTCTGCTGGCTCCACCACCACCAGCCGGCCGCCGCCGGTGACCGGCGGGAGCAGGGCCCCGGCCTCTCTCAGGGTACGTACTTACACGTGTCCATCGCACGGTCTATCAGTATTTATTTATTAACTACTCTCGAGCTTAATTATGGTATTGTTGATAGTTGATGAAGTTAATTATTGTACGCCGTCTCATCGATCAGTTGGACGACTGGGTGCTGTGCCGCATCTACAAGAAGACCAGCAAGGCCGCGGCCGCGGTCGGAGATGAGCAGAGGAGCATGGAGTGCGAGGACTCCGTGGAGGACGCGGTCACCGCGTACCCGCCCTACGCCACGGCGGGCATGGCCGGCGCAGGTGCGCATGGCAGCAACTACGTTCAACTGCTCCATCATCACGACAGCCACGAGGACAACTTCCAGCTAGACGGCCTGCTCACAGAACACGACGTCGGCCTCTCGGCGGGCGCCGCCTCGCTGGGCCACCTTGCCGCGGCGGCGAGGGCCACCAAACAGTTCCTCGCCCCGTCGTCCTCAACCCCGTTCAACTGGCTCGAGGCGTCAACCGG

>HS105_*NAM-1*

GCTTTTTATTATACTGTGCACAAGTATTTTTATATTCTTCCAGTAAGTACAGCGCATGTATGTGATCCTGTCGTCGTGCTTGTTCATGCGCTCGGGCGGGATCATCATCCATCAGAGAAGGCGACCTTCGGGGAGCACGAGTGGTACTTCTTCAGCCCGCGCGACCGCAAGTACCCCAACGGCGCGCGGCCGAACCGGGCGGCGACGTCGGGCTACTGGAAGGCCACCGGCACGGACAAGCCTATCCTGGCCTCGGCCACCGGGTGCGGCCGGGAGAAGGTCGGCGTCAAGAAGGCGCTCGTCTTCTACCGCGGGAAGCCGCCCAGGGGCCTCAAGACCAACTGGATCATGCATGAGTACCGCCTCACCGGAGCCTCTGCTGGCTCCACCACCACCAGCCGGCCGCCGCCGGTGACCGGCGGGAGCAGGGCCCCGGCCTCTCTCAGGGTACGTACTTACACGTGTCCATCGCACGGTCTATCAGTATTTATTTATTAACTACTCTCGAGCTTAATTATGGTATTGTTGATAGTTGATGAAGTTAATTATTGTACGCCGTCTCATCGATCAGTTGGACGACTGGGTGCTGTGCCGCATCTACAAGAAGACCAGCAAGGCCGCGGCCGCGGTCGGAGATGAGCAGAGGAGCATGGAGTGCGAGGACTCCGTGGAGGACGCGGTCACCGCGTACCCGCCCTACGCCACGGCGGGCATGGCCGGCGCAGGTGCGCATGGCAGCAACTACGTTCAACTGCTCCATCATCACGACAGCCACGAGGACAACTTCCAGCTAGACGGCCTGCTCACAGAACACGACGTCAGCCTCTCGGCGGGCGCCGCCTCGCTGGGCCACCTTGCCGCGGCGGCGAGGGCCACCAAACAGTTCCTCGCCCCGTCGTCCTCAACCCCGTTCAACTGGCTCGAGGCGTCAACCGG

>HS106_*NAM-1*

GCTTTATATTATACTGTGCACAAGTATTTTTATATTCTTCCAGTAAGTACAGCGCATGTATGTGATCCTGTCGTCGTGCTTGTTCATGCGCTCGGGCGGGATCATCATCCATCAGAGAAGGCGACCTTCGGGGAGCACGAGTGGTACTTCTTCAGCCCGCGCGACCGCAAGTACGCCAACGGCGCGCGGCCGAACCGGGCGGCGACGTCGGGCTACTGGAAGGCCACCGGCACGGACAAGCCTATCCTGGCCTCGGCCACCGGGTGCGGCCGGGAGAAGGTCGGCGTCAAGAAGGCGCTCGTCTTCTACCGCGGGAAGCCGCCCAGGGGCCTCAAGACCAACTGGATCATGCATGAGTACCGCCTCACCGGAGCCTCTGCTGGCTCCACCACCACCAGCCGGCCGCCGCCGGTGACCGGCGGGAGCAGGGCCCCGGCCTCTCTCAGGGTACGTACTTACACGTGTCCATCGCACGGTCTATCAGTATTTATTTATTAACTACTCTCGAGCTTAATTATGGTATTGTTGATAGTTGATGAAGTTAATTATTGTACGCCGTCTCATCGATCAGTTGGACGACTGGGTGCTGTGCCGCATCTACAAGAAGACCAGCAAGGCCGCGGCCGCGGTCGGAGATGAGCAGAGGAGCATGGAGTGCGAGGACTCCGTGGAGGACGCGGTCACCGCGTACCCGCCCTACGCCACGGCGGGCATGGCCGGCGCAGGTGCGCATGGCAGCAACTACGTTCAACTGCTCCATCATCACGACAGCCACGAGGACAACTTCCAGCTAGACGGCCTGCTCACAGAACACGACGTCGGCCTCTCGGCGGGCGCCGCCTCGCTGGGCCACCTTGCCGCGGCGGCGAGGGCCACCAAACAGTTCCTCGCCCCGTCGTCCTCAACCCCGTTCAACTGGCTCGAGGCGTCAACCGG

>HS107_*NAM-1*

GCTTTATATTATACTGTGCACAAGTATTTTTATATTCTTCCAGTAAGTACAGCGCATGTATGTGATCCTGTCGTCGTGCTTGTTCATGCGCTCGGGCGGGATCATCATCCATCAGAGAAGGCGACCTTCGGGGAGCACGAGTGGTACTTCTTCAGCCCGCGCGACCGCAAGTACCCCAACGGCGCGCGGCCGAACCGGGCGGCGACGTCGGGCTACTGGAAGGCCACCGGCACGGACAAGCCTATCCTGGCCTCGGCCACCGGGTGCGGCCGGGAGAAGGTCGGCGTCAAGAAGGCGCTCGTCTTCTACCGCGGGAAGCCGCCCAGGGGCCTCAAGACCAACTGGATCATGCATGAGTACCGCCTCACCGGAGCCTCTGCTGGCTCCACCACCACCAGCCGGCCGCCGCCGGTGACCGGCGGGAGCAGGGCCCCGGCCTCTCTCAGGGTACGTACTTACACGTGTCCATCGCACGGTCTATCAGTATTTATTTATTAACTACTCTCGAGCTTAATTATGGTATTGTTGATAGTTGATGAAGTTAATTATTGTACGCCGTCTCATCGATCAGTTGGACGACTGGGTGCTGTGCCGCATCTACAAGAAGACCAGCAAGGCCGCGGCCGCGGTCGGAGATGAGCAGAGGAGCATGGAGTGCGAGGACTCCGTGGAGGACGCGGTCACCGCGTACCCGCCCTACGCCACGGCGGGCATGGCCGGCGCAGGTGCGCATGGCAGCAACTACGTTCAACTGCTCCATCATCACGACAGCCACGAGGACAACTTCCAGCTAGACGGCCTGCTCACAGAACACGACGTCAGCCTCTCGGCGGGCGCCGCCTCGCTGGGCCACCTTGCCGCGGCGGCGAGGGCCACCAAACAGTTCCTCGCCCCGTCGTCCTCAACCCCGTTCAACTGGCTCGAGGCGTCAACCGG

>HS108_*NAM-1*

GCTTTTTATTATACTGTGCACAAGTATTTTTATATTCTTCCAGTAAGTACAGCGCATGTATGTGATCCTGTCGTCGTGCTTGTTCATGCGCTCGGGCGGGATCATCATCCATCAGAGAAGGCGACCTTCGGGGAGCACGAGTGGTACTTCTTCAGCCCGCGCGACCGCAAGTACGCCAACGGCGCGCGGCCGAACCGGGCGGCGACGTCGGGCTACTGGAAGGCCACCGGCACGGACAAGCCTATCCTGGCCTCGGCCACCGGGTGCGGCCGGGAGAAGGTCGGCGTCAAGAAGGCGCTCGTCTTCTACCGCGGGAAGCCGCCCAGGGGCCTCAAGACCAACTGGATCATGCATGAGTACCGCCTCACCGGAGCCTCTGCTGGCTCCACCACCACCAGCCGGCCGCCGCCGGTGACCGGCGGGAGCAGGGCCCCGGCCTCTCTCAGGGTACGTACTTACACGTGTCCATCGCACGGTCTATCAGTATTTATTTATTAACTACTCTCGAGCTTAATTATGGTATTGTTGATAGTTGATGAAGTTAATTATTGTACGCCGTCTCATCGATCAGTTGGACGACTGGGTGCTGTGCCGCATCTACAAGAAGACCAGCAAGGCCGCGGCCGCGGTCGGAGATGAGCAGAGGAGCATGGAGTGCGAGGACTCCGTGGAGGACGCGGTCACCGCGTACCCGCCCTACGCCACGGCGGGCATGGCCGGCGCAGGTGCGCATGGCAGCAACTACGTTCAACTGCTCCATCATCACGACAGCCACGAGGACAACTTCCAGCTAGACGGCCTGCTCACAGAACACGACGTCGGCCTCTCGGCGGGCGCCGCCTCGCTGGGCCACCTTGCCGCGGCGGCGAGGGCCACCAAACAGTTCCTCGCCCCGTCGTCCTCAACCCCGTTCAACTGGCTCGAGGCGTCAACCGG

>HS109_*NAM-1*

GCTTTATATTATACTGTGCACAAGTATTTTTATATTCTTCCAGTAAGTACAGCGCATGTATGTGATCCTGTCGTCGTGCTTGTTCATGCGCTCGGGCGGGATCATCATCCATCAGAGAAGGCGACCTTCGGGGAGCACGAGTGGTACTTCTTCAGCCCGCGCGACCGCAAGTACGCCAACGGCGCGCGGCCGAACCGGGCGGCGACGTCGGGCTACTGGAAGGCCACCGGCACGGACAAGCCTATCCTGGCCTCGGCCACCGGGTGCGGCCGGGAGAAGGTCGGCGTCAAGAAGGCGCTCGTCTTCTACCGCGGGAAGCCGCCCAGGGGCCTCAAGACCAACTGGATCATGCATGAGTACCGCCTCACCGGAGCCTCTGCTGGCTCCACCACCACCAGCCGGCCGCCGCCGGTGACCGGCGGGAGCAGGGCCCCGGCCTCTCTCAGGGTACGTACTTACACGTGTCCATCGCACGGTCTATCAGTATTTATTTATTAACTACTCTCGAGCTTAATTATGGTATTGTTGATAGTTGATGAAGTTAATTATTGTACGCCGTCTCATCGATCAGTTGGACGACTGGGTGCTGTGCCGCATCTACAAGAAGACCAGCAAGGCCGCGGCCGCGGTCGGAGATGAGCAGAGGAGCATGGAGTGCGAGGACTCCGTGGAGGACGCGGTCACCGCGTACCCGCCCTACGCCACGGCGGGCATGGCCGGCGCAGGTGCGCATGGCAGCAACTACGTTCAACTGCTCCATCATCACGACAGCCACGAGGACAACTTCCAGCTAGACGGCCTGCTCACAGAACACGACGTCGGCCTCTCGGCGGGCGCCGCCTCGCTGGGCCACCTTGCCGCGGCGGCGAGGGCCACCAAACAGTTCCTCGCCCCGTCGTCCTCAACCCCGTTCAACTGGCTCGAGGCGTCAACCGG

>HS110_*NAM-1*

GCTTTTTATTATACTGTGCACAAGTATTTTTATATTCTTCCAGTAAGTACAGCGCATGTATGTGATCCTGTCGTCGTGCTTGTTCATGCGCTCGGGCGGGATCATCATCCATCAGAGAAGGCGACCTTCGGGGAGCACGAGTGGTACTTCTTCAGCCCGCGCGACCGCAAGTACGCCAACGGCGCGCGGCCGAACCGGGCGGCGACGTCGGGCTACTGGAAGGCCACCGGCACGGACAAGCCTATCCTGGCCTCGGCCACCGGGTGCGGCCGGGAGAAGGTCGGCGTCAAGAAGGCGCTCGTCTTCTACCGCGGGAAGCCGCCCAGGGGCCTCAAGACCAACTGGATCATGCATGAGTACCGCCTCACCGGAGCCTCTGCTGGCTCCACCACCACCAGCCGGCCGCCGCCGGTGACCGGCGGGAGCAGGGCCCCGGCCTCTCTCAGGGTACGTACTTACACGTGTCCATCGCACGGTCTATCAGTATTTATTTATTAACTACTCTCGAGCTTAATTATGGTATTGTTGATAGTTGATGAAGTTAATTATTGTACGCCGTCTCATCGATCAGTTGGACGACTGGGTGCTGTGCCGCATCTACAAGAAGACCAGCAAGGCCGCGGCCGCGGTCGGAGATGAGCAGAGGAGCATGGAGTGCGAGGACTCCGTGGAGGACGCGGTCACCGCGTACCCGCCCTACGCCACGGCGGGCATGGCCGGCGCAGGTGCGCATGGCAGCAACTACGTTCAACTGCTCCATCATCACGACAGCCACGAGGACAACTTCCAGCTAGACGGCCTGCTCACAGAACACGACGTCGGCCTCTCGGCGGGCGCCGCCTCGCTGGGCCACCTTGCCGCGGCGGCGAGGGCCACCAAACAGTTCCTCGCCCCGTCGTCCTCAACCCCGTTCAACTGGCTCGAGGCGTCAACCGG

>HS111_*NAM-1*

GCTTTTTATTATACTGTGCACAAGTATTTTTATATTCTTCCAGTAAGTACAGCGCATGTATGTGATCCTGTCGTCGTGCTTGTTCATGCGCTCGGGCGGGATCATCATCCATCAGAGAAGGCGACCTTCGGGGAGCACGAGTGGTACTTCTTCAGCCCGCGCGACCGCAAGTACCCCAACGGCGCGCGGCCGAACCGGGCGGCGACGTCGGGCTACTGGAAGGCCACCGGCACGGACAAGCCTATCCTGGCCTCGGCCACCGGGTGCGGCCGGGAGAAGGTCGGCGTCAAGAAGGCGCTCGTCTTCTACCGCGGGAAGCCGCCCAGGGGCCTCAAGACCAACTGGATCATGCATGAGTACCGCCTCACCGGAGCCTCTGCTGGCTCCACCACCACCAGCCGGCCGCCGCCGGTGACCGGCGGGAGCAGGGCCCCGGCCTCTCTCAGGGTACGTACTTACACGTGTCCATCGCACGGTCTATCAGTATTTATTTATTAACTACTCTCGAGCTTAATTATGGTATTGTTGATAGTTGATGAAGTTAATTATTGTACGCCGTCTCATCGATCAGTTGGACGACTGGGTGCTGTGCCGCATCTACAAGAAGACCAGCAAGGCCGCGGCCGCGGTCGGAGATGAGCAGAGGAGCATGGAGTGCGAGGACTCCGTGGAGGACGCGGTCACCGCGTACCCGCCCTACGCCACGGCGGGCATGGCCGGCGCAGGTGCGCATGGCAGCAACTACGTTCAACTGCTCCATCATCACGACAGCCACGAGGACAACTTCCAGCTAGACGGCCTGCTCACAGAACACGACGTCGGCCTCTCGGCGGGCGCCGCCTCGCTGGGCCACCTTGCCGCGGCGGCGAGGGCCACCAAACAGTTCCTCGCCCCGTCGTCCTCAACCCCGTTCAACTGGCTCGAGGCGTCAACCGG

>HS112_*NAM-1*

GCTTTTTATTATACTGTGCACAAGTATTTTTATATTCTTCCAGTAAGTACAGCGCATGTATGTGATCCTGTCGTCGTGCTTGTTCATGCGCTCGGGCGGGATCATCATCCATCAGAGAAGGCGACCTTCGGGGAGCACGAGTGGTACTTCTTCAGCCCGCGCGACCGCAAGTACCCCAACGGCGCGCGGCCGAACCGGGCGGCGACGTCGGGCTACTGGAAGGCCACCGGCACGGACAAGCCTATCCTGGCCTCGGCCACCGGGTGCGGCCGGGAGAAGGTCGGCGTCAAGAAGGCGCTCGTCTTCTACCGCGGGAAGCCGCCCAGGGGCCTCAAGACCAACTGGATCATGCATGAGTACCGCCTCACCGGAGCCTCTGCTGGCTCCACCACCACCAGCCGGCCGCCGCCGGTGACCGGCGGGAGCAGGGCCCCGGCCTCTCTCAGGGTACGTACTTACACGTGTCCATCGCACGGTCTATCAGTATTTATTTATTAACTACTCTCGAGCTTAATTATGGTATTGTTGATAGTTGATGAAGTTAATTATTGTACGCCGTCTCATCGATCAGTTGGACGACTGGGTGCTGTGCCGCATCTACAAGAAGACCAGCAAGGCCGCGGCCGCGGTCGGAGATGAGCAGAGGAGCATGGAGTGCGAGGACTCCGTGGAGGACGCGGTCACCGCGTACCCGCCCTACGCCACGGCGGGCATGGCCGGCGCAGGTGCGCATGGCAGCAACTACGTTCAACTGCTCCATCATCACGACAGCCACGAGGACAACTTCCAGCTAGACGGCCTGCTCACAGAACACGACGTCGGCCTCTCGGCGGGCGCCGCCTCGCTGGGCCACCTTGCCGCGGCGGCGAGGGCCACCAAACAGTTCCTCGCCCCGTCGTCCTCAACCCCGTTCAACTGGCTCGAGGCGTCAACCGG

>HS113_*NAM-1*

GCTTTTTATTATACTGTGCACAAGTATTTTTATATTCTTCCAGTAAGTACAGCGCATGTATGTGATCCTGTCGTCGTGCTTGTTCATGCGCTCGGGCGGGATCATCATCCATCAGAGAAGGCGACCTTCGGGGAGCACGAGTGGTACTTCTTCAGCCCGCGCGACCGCAAGTACCCCAACGGCGCGCGGCCGAACCGGGCGGCGACGTCGGGCTACTGGAAGGCCACCGGCACGGACAAGCCTATCCTGGCCTCGGCCACCGGGTGCGGCCGGGAGAAGGTCGGCGTCAAGAAGGCGCTCGTCTTCTACCGCGGGAAGCCGCCCAGGGGCCTCAAGACCAACTGGATCATGCATGAGTACCGCCTCACCGGAGCCTCTGCTGGCTCCACCACCACCAGCCGGCCGCCGCCGGTGACCGGCGGGAGCAGGGCCCCGGCCTCTCTCAGGGTACGTACTTACACGTGTCCATCGCACGGTCTATCAGTATTTATTTATTAACTACTCTCGAGCTTAATTATGGTATTGTTGATAGTTGATGAAGTTAATTATTGTACGCCGTCTCATCGATCAGTTGGACGACTGGGTGCTGTGCCGCATCTACAAGAAGACCAGCAAGGCCGCGGCCGCGGTCGGAGATGAGCAGAGGAGCATGGAGTGCGAGGACTCCGTGGAGGACGCGGTCACCGCGTACCCGCCCTACGCCACGGCGGGCATGGCCGGCGCAGGTGCGCATGGCAGCAACTACGTTCAACTGCTCCATCATCACGACAGCCACGAGGACAACTTCCAGCTAGACGGCCTGCTCACAGAACACGACGTCGGCCTCTCGGCGGGCGCCGCCTCGCTGGGCCACCTTGCCGCGGCGGCGAGGGCCACCAAACAGTTCCTCGCCCCGTCGTCCTCAACCCCGTTCAACTGGCTCGAGGCGTCAACCGG

>HS114_*NAM-1*

GCTTTTTATTATACTGTGCACAAGTATTTTTATATTCTTCCAGTAAGTACAGCGCATGTATGTGATCCTGTCGTCGTGCTTGTTCATGCGCTCGGGCGGGATCATCATCCATCAGAGAAGGCGACCTTCGGGGAGCACGAGTGGTACTTCTTCAGCCCGCGCGACCGCAAGTACGCCAACGGCGCGCGGCCGAACCGGGCGGCGACGTCGGGCTACTGGAAGGCCACCGGCACGGACAAGCCTATCCTGGCCTCGGCCACCGGGTGCGGCCGGGAGAAGGTCGGCGTCAAGAAGGCGCTCGTCTTCTACCGCGGGAAGCCGCCCAGGGGCCTCAAGACCAACTGGATCATGCATGAGTACCGCCTCACCGGAGCCTCTGCTGGCTCCACCACCACCAGCCGGCCGCCGCCGGTGACCGGCGGGAGCAGGGCCCCGGCCTCTCTCAGGGTACGTACTTACACGTGTCCATCGCACGGTCTATCAGTATTTATTTATTAACTACTCTCGAGCTTAATTATGGTATTGTTGATAGTTGATGAAGTTAATTATTGTACGCCGTCTCATCGATCAGTTGGACGACTGGGTGCTGTGCCGCATCTACAAGAAGACCAGCAAGGCCGCGGCCGCGGTCGGAGATGAGCAGAGGAGCATGGAGTGCGAGGACTCCGTGGAGGACGCGGTCACCGCGTACCCGCCCTACGCCACGGCGGGCATGGCCGGCGCAGGTGCGCATGGCAGCAACTACGTTCAACTGCTCCATCATCACGACAGCCACGAGGACAACTTCCAGCTAGACGGCCTGCTCACAGAACACGACGTCGGCCTCTCGGCGGGCGCCGCCTCGCTGGGCCACCTTGCCGCGGCGGCGAGGGCCACCAAACAGTTCCTCGCCCCGTCGTCCTCAACCCCGTTCAACTGGCTCGAGGCGTCAACCGG

>HS115_*NAM-1*

GCTTTTTATTATACTGTGCACAAGTATTTTTATATTCTTCCAGTAAGTACAGCGCATGTATGTGATCCTGTCGTCGTGCTTGTTCATGCGCTCGGGCGGGATCATCATCCATCAGAGAAGGCGACCTTCGGGGAGCACGAGTGGTACTTCTTCAGCCCGCGCGACCGCAAGTACGCCAACGGCGCGCGGCCGAACCGGGCGGCGACGTCGGGCTACTGGAAGGCCACCGGCACGGACAAGCCTATCCTGGCCTCGGCCACCGGGTGCGGCCGGGAGAAGGTCGGCGTCAAGAAGGCGCTCGTCTTCTACCGCGGGAAGCCGCCCAGGGGCCTCAAGACCAACTGGATCATGCATGAGTACCGCCTCACCGGAGCCTCTGCTGGCTCCACCACCACCAGCCGGCCGCCGCCGGTGACCGGCGGGAGCAGGGCCCCGGCCTCTCTCAGGGTACGTACTTACACGTGTCCATCGCACGGTCTATCAGTATTTATTTATTAACTACTCTCGAGCTTAATTATGGTATTGTTGATAGTTGATGAAGTTAATTATTGTACGCCGTCTCATCGATCAGTTGGACGACTGGGTGCTGTGCCGCATCTACAAGAAGACCAGCAAGGCCGCGGCCGCGGTCGGAGATGAGCAGAGGAGCATGGAGTGCGAGGACTCCGTGGAGGACGCGGTCACCGCGTACCCGCCCTACGCCACGGCGGGCATGGCCGGCGCAGGTGCGCATGGCAGCAACTACGTTCAACTGCTCCATCATCACGACAGCCACGAGGACAACTTCCAGCTAGACGGCCTGCTCACAGAACACGACGTCGGCCTCTCGGCGGGCGCCGCCTCGCTGGGCCACCTTGCCGCGGCGGCGAGGGCCACCAAACAGTTCCTCGCCCCGTCGTCCTCAACCCCGTTCAACTGGCTCGAGGCGTCAACCGG

>HS116_*NAM-1*

GCTTTTTATTATACTGTGCACAAGTATTTTTATATTCTTCCAGTAAGTACAGCGCATGTATGTGATCCTGTCGTCGTGCTTGTTCATGCGCTCGGGCGGGATCATCATCCATCAGAGAAGGCGACCTTCGGGGAGCACGAGTGGTACTTCTTCAGCCCGCGCGACCGCAAGTACCCCAACGGCGCGCGGCCGAACCGGGCGGCGACGTCGGGCTACTGGAAGGCCACCGGCACGGACAAGCCTATCCTGGCCTCGGCCACCGGGTGCGGCCGGGAGAAGGTCGGCGTCAAGAAGGCGCTCGTCTTCTACCGCGGGAAGCCGCCCAGGGGCCTCAAGACCAACTGGATCATGCATGAGTACCGCCTCACCGGAGCCTCTGCTGGCTCCACCACCACCAGCCGGCCGCCGCCGGTGACCGGCGGGAGCAGGGCCCCGGCCTCTCTCAGGGTACGTACTTACACGTGTCCATCGCACGGTCTATCAGTATTTATTTATTAACTACTCTCGAGCTTAATTATGGTATTGTTGATAGTTGATGAAGTTAATTATTGTACGCCGTCTCATCGATCAGTTGGACGACTGGGTGCTGTGCCGCATCTACAAGAAGACCAGCAAGGCCGCGGCCGCGGTCGGAGATGAGCAGAGGAGCATGGAGTGCGAGGACTCCGTGGAGGACGCGGTCACCGCGTACCCGCCCTACGCCACGGCGGGCATGGCCGGCGCAGGTGCGCATGGCAGCAACTACGTTCAACTGCTCCATCATCACGACAGCCACGAGGACAACTTCCAGCTAGACGGCCTGCTCACAGAACACGACGTCGGCCTCTCGGCGGGCGCCGCCTCGCTGGGCCACCTTGCCGCGGCGGCGAGGGCCACCAAACAGTTCCTCGCCCCGTCGTCCTCAACCCCGTTCAACTGGCTCGAGGCGTCAACCGG

>HS117_*NAM-1*

GCTTTTTATTATACTGTGCACAAGTATTTTTATATTCTTCCAGTAAGTACAGCGCATGTATGTGATCCTGTCGTCGTGCTTGTTCATGCGCTCGGGCGGGATCATCATCCATCAGAGAAGGCGACCTTCGGGGAGCACGAGTGGTACTTCTTCAGCCCGCGCGACCGCAAGTACCCCAACGGCGCGCGGCCGAACCGGGCGGCGACGTCGGGCTACTGGAAGGCCACCGGCACGGACAAGCCTATCCTGGCCTCGGCCACCGGGTGCGGCCGGGAGAAGGTCGGCGTCAAGAAGGCGCTCGTCTTCTACCGCGGGAAGCCGCCCAGGGGCCTCAAGACCAACTGGATCATGCATGAGTACCGCCTCACCGGAGCCTCTGCTGGCTCCACCACCACCAGCCGGCCGCCGCCGGTGACCGGCGGGAGCAGGGCCCCGGCCTCTCTCAGGGTACGTACTTACACGTGTCCATCGCACGGTCTATCAGTATTTATTTATTAACTACTCTCGAGCTTAATTATGGTATTGTTGATAGTTGATGAAGTTAATTATTGTACGCCGTCTCATCGATCAGTTGGACGACTGGGTGCTGTGCCGCATCTACAAGAAGACCAGCAAGGCCGCGGCCGCGGTCGGAGATGAGCAGAGGAGCATGGAGTGCGAGGACTCCGTGGAGGACGCGGTCACCGCGTACCCGCCCTACGCCACGGCGGGCATGGCCGGCGCAGGTGCGCATGGCAGCAACTACGTTCAACTGCTCCATCATCACGACAGCCACGAGGACAACTTCCAGCTAGACGGCCTGCTCACAGAACACGACGTCGGCCTCTCGGCGGGCGCCGCCTCGCTGGGCCACCTTGCCGCGGCGGCGAGGGCCACCAAACAGTTCCTCGCCCCGTCGTCCTCAACCCCGTTCAACTGGCTCGAGGCGTCAACCGG

>HS118_*NAM-1*

GCTTTTTATTATACTGTGCACAAGTATTTTTATATTCTTCCAGTAAGTACAGCGCATGTATGTGATCCTGTCGTCGTGCTTGTTCATGCGCTCGGGCGGGATCATCATCCATCAGAGAAGGCGACCTTCGGGGAGCACGAGTGGTACTTCTTCAGCCCGCGCGACCGCAAGTACCCCAACGGCGCGCGGCCGAACCGGGCGGCGACGTCGGGCTACTGGAAGGCCACCGGCACGGACAAGCCTATCCTGGCCTCGGCCACCGGGTGCGGCCGGGAGAAGGTCGGCGTCAAGAAGGCGCTCGTCTTCTACCGCGGGAAGCCGCCCAGGGGCCTCAAGACCAACTGGATCATGCATGAGTACCGCCTCACCGGAGCCTCTGCTGGCTCCACCACCACCAGCCGGCCGCCGCCGGTGACCGGCGGGAGCAGGGCCCCGGCCTCTCTCAGGGTACGTACTTACACGTGTCCATCGCACGGTCTATCAGTATTTATTTATTAACTACTCTCGAGCTTAATTATGGTATTGTTGATAGTTGATGAAGTTAATTATTGTACGCCGTCTCATCGATCAGTTGGACGACTGGGTGCTGTGCCGCATCTACAAGAAGACCAGCAAGGCCGCGGCCGCGGTCGGAGATGAGCAGAGGAGCATGGAGTGCGAGGACTCCGTGGAGGACGCGGTCACCGCGTACCCGCCCTACGCCACGGCGGGCATGGCCGGCGCAGGTGCGCATGGCAGCAACTACGTTCAACTGCTCCATCATCACGACAGCCACGAGGACAACTTCCAGCTAGACGGCCTGCTCACAGAACACGACGTCGGCCTCTCGGCGGGCGCCGCCTCGCTGGGCCACCTTGCCGCGGCGGCGAGGGCCACCAAACAGTTCCTCGCCCCGTCGTCCTCAACCCCGTTCAACTGGCTCGAGGCGTCAACCGG

>HS119_*NAM-1*

GCTTTTTATTATACTGTGCACAAGTATTTTTATATTCTTCCAGTAAGTACAGCGCATGTATGTGATCCTGTCGTCGTGCTTGTTCATGCGCTCGGGCGGGATCATCATCCATCAGAGAAGGCGACCTTCGGGGAGCACGAGTGGTACTTCTTCAGCCCGCGCGACCGCAAGTACGCCAACGGCGCGCGGCCGAACCGGGCGGCGACGTCGGGCTACTGGAAGGCCACCGGCACGGACAAGCCTATCCTGGCCTCGGCCACCGGGTGCGGCCGGGAGAAGGTCGGCGTCAAGAAGGCGCTCGTCTTCTACCGCGGGAAGCCGCCCAGGGGCCTCAAGACCAACTGGATCATGCATGAGTACCGCCTCACCGGAGCCTCTGCTGGCTCCACCACCACCAGCCGGCCGCCGCCGGTGACCGGCGGGAGCAGGGCCCCGGCCTCTCTCAGGGTACGTACTTACACGTGTCCATCGCACGGTCTATCAGTATTTATTTATTAACTACTCTCGAGCTTAATTATGGTATTGTTGATAGTTGATGAAGTTAATTATTGTACGCCGTCTCATCGATCAGTTGGACGACTGGGTGCTGTGCCGCATCTACAAGAAGACCAGCAAGGCCGCGGCCGCGGTCGGAGATGAGCAGAGGAGCATGGAGTGCGAGGACTCCGTGGAGGACGCGGTCACCGCGTACCCGCCCTACGCCACGGCGGGCATGGCCGGCGCAGGTGCGCATGGCAGCAACTACGTTCAACTGCTCCATCATCACGACAGCCACGAGGACAACTTCCAGCTAGACGGCCTGCTCACAGAACACGACGTCGGCCTCTCGGCGGGCGCCGCCTCGCTGGGCCACCTTGCCGCGGCGGCGAGGGCCACCAAACAGTTCCTCGCCCCGTCGTCCTCAACCCCGTTCAACTGGCTCGAGGCGTCAACCGG

>67_*NAM-1*

GCTTTTTATTATACTGTGCACAAGTATTTTTATATTCTTCCAGTAAGTACAGCGCATGTATGTGATCCTGTCGTCGTGCTTGTTCATGCGCTCGGGCGGGATCATCATCCATCAGAGAAGGCGACCTTCGGGGAGCACGAGTGGTACTTCTTCAGCCCGCGCGACCGCAAGTACGCCAACGGCGCGCGGCCGAACCGGGCGGCGACGTCGGGCTACTGGAAGGCCACCGGCACGGACAAGCCTATCCTGGCCTCGGCCACCGGGTGCGGCCGGGAGAAGGTCGGCGTCAAGAAGGCGCTCGTCTTCTACCGCGGGAAGCCGCCCAGGGGCCTCAAGACCAACTGGATCATGCATGAGTACCGCCTCACCGGAGCCTCTGCTGGCTCCACCACCACCAGCCGGCCGCCGCCGGTGACCGGCGGGAGCAGGGCCCCGGCCTCTCTCAGGGTACGTACTTACACGTGTCCATCGCACGGTCTATCAGTATTTATTTATTAACTACTCTCGAGCTTAATTATGGTATTGTTGATAGTTGATGAAGTTAATTATTGTACGCCGTCTCATCGATCAGTTGGACGACTGGGTGCTGTGCCGCATCTACAAGAAGACCAGCAAGGCCGCGGCCGCGGTCGGAGATGAGCAGAGGAGCATGGAGTGCGAGGACTCCGTGGAGGACGCGGTCACCGCGTACCCGCCCTACGCCACGGCGGGCATGGCCGGCGCAGGTGCGCATGGCAGCAACTACGTTCAACTGCTCCATCATCACGACAGCCACGAGGACAACTTCCAGCTAGACGGCCTGCTCACAGAACACGACGTCGGCCTCTCGGCGGGCGCCGCCTCGCTGGGCCACCTTGCCGCGGCGGCGAGGGCCACCAAACAGTTCCTCGCCCCGTCGTCCTCAACCCCGTTCAACTGGCTCGAGGCGTCAACCGG

>68_*NAM-1*

GCTTTTTATTATACTGTGCACAAGTATTTTTATATTCTTCCAGTAAGTACAGCGCATGTATGTGATCCTGTCGTCGTGCTTGTTCATGCGCTCGGGCGGGATCATCATCCATCAGAGAAGGCGACCTTCGGGGAGCACGAGTGGTACTTCTTCAGCCCGCGCGACCGCAAGTACGCCAACGGCGCGCGGCCGAACCGGGCGGCGACGTCGGGCTACTGGAAGGCCACCGGCACGGACAAGCCTATCCTGGCCTCGGCCACCGGGTGCGGCCGGGAGAAGGTCGGCGTCAAGAAGGCGCTCGTCTTCTACCGCGGGAAGCCGCCCAGGGGCCTCAAGACCAACTGGATCATGCATGAGTACCGCCTCACCGGAGCCTCTGCTGGCTCCACCACCACCAGCCGGCCGCCGCCGGTGACCGGCGGGAGCAGGGCCCCGGCCTCTCTCAGGGTACGTACTTACACGTGTCCATCGCACGGTCTATCAGTATTTATTTATTAACTACTCTCGAGCTTAATTATGGTATTGTTGATAGTTGATGAAGTTAATTATTGTACGCCGTCTCATCGATCAGTTGGACGACTGGGTGCTGTGCCGCATCTACAAGAAGACCAGCAAGGCCGCGGCCGCGGTCGGAGATGAGCAGAGGAGCATGGAGTGCGAGGACTCCGTGGAGGACGCGGTCACCGCGTACCCGCCCTACGCCACGGCGGGCATGGCCGGCGCAGGTGCGCATGGCAGCAACTACGTTCAACTGCTCCATCATCACGACAGCCACGAGGACAACTTCCAGCTAGACGGCCTGCTCACAGAACACGACGTCGGCCTCTCGGCGGGCGCCGCCTCGCTGGGCCACCTTGCCGCGGCGGCGAGGGCCACCAAACAGTTCCTCGCCCCGTCGTCCTCAACCCCGTTCAACTGGCTCGAGGCGTCAACCGG

>69_*NAM-1*

GCTTTTTATTATACTGTGCACAAGTATTTTTATATTCTTCCAGTAAGTACAGCGCATGTATGTGATCCTGTCGTCGTGCTTGTTCATGCGCTCGGGCGGGATCATCATCCATCAGAGAAGGCGACCTTCGGGGAGCACGAGTGGTACTTCTTCAGCCCGCGCGACCGCAAGTACGCCAACGGCGCGCGGCCGAACCGGGCGGCGACGTCGGGCTACTGGAAGGCCACCGGCACGGACAAGCCTATCCTGGCCTCGGCCACCGGGTGCGGCCGGGAGAAGGTCGGCGTCAAGAAGGCGCTCGTCTTCTACCGCGGGAAGCCGCCCAGGGGCCTCAAGACCAACTGGATCATGCATGAGTACCGCCTCACCGGAGCCTCTGCTGGCTCCACCACCACCAGCCGGCCGCCGCCGGTGACCGGCGGGAGCAGGGCCCCGGCCTCTCTCAGGGTACGTACTTACACGTGTCCATCGCACGGTCTATCAGTATTTATTTATTAACTACTCTCGAGCTTAATTATGGTATTGTTGATAGTTGATGAAGTTAATTATTGTACGCCGTCTCATCGATCAGTTGGACGACTGGGTGCTGTGCCGCATCTACAAGAAGACCAGCAAGGCCGCGGCCGCGGTCGGAGATGAGCAGAGGAGCATGGAGTGCGAGGACTCCGTGGAGGACGCGGTCACCGCGTACCCGCCCTACGCCACGGCGGGCATGGCCGGCGCAGGTGCGCATGGCAGCAACTACGTTCAACTGCTCCATCATCACGACAGCCACGAGGACAACTTCCAGCTAGACGGCCTGCTCACAGAACACGACGTCGGCCTCTCGGCGGGCGCCGCCTCGCTGGGCCACCTTGCCGCGGCGGCGAGGGCCACCAAACAGTTCCTCGCCCCGTCGTCCTCAACCCCGTTCAACTGGCTCGAGGCGTCAACCGG

>71_*NAM-1*

GCTTTTTATTATACTGTGCACAAGTATTTTTATATTCTTCCAGTAAGTACAGCGCATGTATGTGATCCTGTCGTCGTGCTTGTTCATGCGCTCGGGCGGGATCATCATCCATCAGAGAAGGCGACCTTCGGGGAGCACGAGTGGTACTTCTTCAGCCCGCGCGACCGCAAGTACCCCAACGGCGCGCGGCCGAACCGGGCGGCGACGTCGGGCTACTGGAAGGCCACCGGCACGGACAAGCCTATCCTGGCCTCGGCCACCGGGTGCGGCCGGGAGAAGGTCGGCGTCAAGAAGGCGCTCGTCTTCTACCGCGGGAAGCCGCCCAGGGGCCTCAAGACCAACTGGATCATGCATGAGTACCGCCTCACCGGAGCCTCTGCTGGCTCCACCACCACCAGCCGGCCGCCGCCGGTGACCGGCGGGAGCAGGGCCCCGGCCTCTCTCAGGGTACGTACTTACACGTGTCCATCGCACGGTCTATCAGTATTTATTTATTAACTACTCTCGAGCTTAATTATGGTATTGTTGATAGTTGATGAAGTTAATTATTGTACGCCGTCTCATCGATCAGTTGGACGACTGGGTGCTGTGCCGCATCTACAAGAAGACCAGCAAGGCCGCGGCCGCGGTCGGAGATGAGCAGAGGAGCATGGAGTGCGAGGACTCCGTGGAGGACGCGGTCACCGCGTACCCGCCCTACGCCACGGCGGGCATGGCCGGCGCAGGTGCGCATGGCAGCAACTACGTTCAACTGCTCCATCATCACGACAGCCACGAGGACAACTTCCAGCTAGACGGCCTGCTCACAGAACACGACGTCGGCCTCTCGGCGGGCGCCGCCTCGCTGGGCCACCTTGCCGCGGCGGCGAGGGCCACCAAACAGTTCCTCGCCCCGTCGTCCTCAACCCCGTTCAACTGGCTCGAGGCGTCAACCGG

>79_*NAM-1*

GCTTTTTATTATACTGTGCACAAGTATTTTTATATTCTTCCAGTAAGTACAGCGCATGTATGTGATCCTGTCGTCGTGCTTGTTCATGCGCTCGGGCGGGATCATCATCCATCAGAGAAGGCGACCTTCGGGGAGCACGAGTGGTACTTCTTCAGCCCGCGCGACCGCAAGTACGCCAACGGCGCGCGGCCGAACCGGGCGGCGACGTCGGGCTACTGGAAGGCCACCGGCACGGACAAGCCTATCCTGGCCTCGGCCACCGGGTGCGGCCGGGAGAAGGTCGGCGTCAAGAAGGCGCTCGTCTTCTACCGCGGGAAGCCGCCCAGGGGCCTCAAGACCAACTGGATCATGCATGAGTACCGCCTCACCGGAGCCTCTGCTGGCTCCACCACCACCAGCCGGCCGCCGCCGGTGACCGGCGGGAGCAGGGCCCCGGCCTCTCTCAGGGTACGTACTTACACGTGTCCATCGCACGGTCTATCAGTATTTATTTATTAACTACTCTCGAGCTTAATTATGGTATTGTTGATAGTTGATGAAGTTAATTATTGTACGCCGTCTCATCGATCAGTTGGACGACTGGGTGCTGTGCCGCATCTACAAGAAGACCAGCAAGGCCGCGGCCGCGGTCGGAGATGAGCAGAGGAGCATGGAGTGCGAGGACTCCGTGGAGGACGCGGTCACCGCGTACCCGCCCTACGCCACGGCGGGCATGGCCGGCGCAGGTGCGCATGGCAGCAACTACGTTCAACTGCTCCATCATCACGACAGCCACGAGGACAACTTCCAGCTAGACGGCCTGCTCACAGAACACGACGTCGGCCTCTCGGCGGGCGCCGCCTCGCTGGGCCACCTTGCCGCGGCGGCGAGGGCCACCAAACAGTTCCTCGCCCCGTCGTCCTCAACCCCGTTCAACTGGCTCGAGGCGTCAACCGG

>80_*NAM-1*

GCTTTTTATTATACTGTGCACAAGTATTTTTATATTCTTCCAGTAAGTACAGCGCATGTATGTGATCCTGTCGTCGTGCTTGTTCATGCGCTCGGGCGGGATCATCATCCATCAGAGAAGGCGACCTTCGGGGAGCACGAGTGGTACTTCTTCAGCCCGCGCGACCGCAAGTACGCCAACGGCGCGCGGCCGAACCGGGCGGCGACGTCGGGCTACTGGAAGGCCACCGGCACGGACAAGCCTATCCTGGCCTCGGCCACCGGGTGCGGCCGGGAGAAGGTCGGCGTCAAGAAGGCGCTCGTCTTCTACCGCGGGAAGCCGCCCAGGGGCCTCAAGACCAACTGGATCATGCATGAGTACCGCCTCACCGGAGCCTCTGCTGGCTCCACCACCACCAGCCGGCCGCCGCCGGTGACCGGCGGGAGCAGGGCCCCGGCCTCTCTCAGGGTACGTACTTACACGTGTCCATCGCACGGTCTATCAGTATTTATTTATTAACTACTCTCGAGCTTAATTATGGTATTGTTGATAGTTGATGAAGTTAATTATTGTACGCCGTCTCATCGATCAGTTGGACGACTGGGTGCTGTGCCGCATCTACAAGAAGACCAGCAAGGCCGCGGCCGCGGTCGGAGATGAGCAGAGGAGCATGGAGTGCGAGGACTCCGTGGAGGACGCGGTCACCGCGTACCCGCCCTACGCCACGGCGGGCATGGCCGGCGCAGGTGCGCATGGCAGCAACTACGTTCAACTGCTCCATCATCACGACAGCCACGAGGACAACTTCCAGCTAGACGGCCTGCTCACAGAACACGACGTCGGCCTCTCGGCGGGCGCCGCCTCGCTGGGCCACCTTGCCGCGGCGGCGAGGGCCACCAAACAGTTCCTCGCCCCGTCGTCCTCAACCCCGTTCAACTGGCTCGAGGCGTCAACCGG

>81_*NAM-1*

GCTTTTTATTATACTGTGCACAAGTATTTTTATATTCTTCCAGTAAGTACAGCGCATGTATGTGATCCTGTCGTCGTGCTTGTTCATGCGCTCGGGCGGGATCATCATCCATCAGAGAAGGCGACCTTCGGGGAGCACGAGTGGTACTTCTTCAGCCCGCGCGACCGCAAGTACGCCAACGGCGCGCGGCCGAACCGGGCGGCGACGTCGGGCTACTGGAAGGCCACCGGCACGGACAAGCCTATCCTGGCCTCGGCCACCGGGTGCGGCCGGGAGAAGGTCGGCGTCAAGAAGGCGCTCGTCTTCTACCGCGGGAAGCCGCCCAGGGGCCTCAAGACCAACTGGATCATGCATGAGTACCGCCTCACCGGAGCCTCTGCTGGCTCCACCACCACCAGCCGGCCGCCGCCGGTGACCGGCGGGAGCAGGGCCCCGGCCTCTCTCAGGGTACGTACTTACACGTGTCCATCGCACGGTCTATCAGTATTTATTTATTAACTACTCTCGAGCTTAATTATGGTATTGTTGATAGTTGATGAAGTTAATTATTGTACGCCGTCTCATCGATCAGTTGGACGACTGGGTGCTGTGCCGCATCTACAAGAAGACCAGCAAGGCCGCGGCCGCGGTCGGAGATGAGCAGAGGAGCATGGAGTGCGAGGACTCCGTGGAGGACGCGGTCACCGCGTACCCGCCCTACGCCACGGCGGGCATGGCCGGCGCAGGTGCGCATGGCAGCAACTACGTTCAACTGCTCCATCATCACGACAGCCACGAGGACAACTTCCAGCTAGACGGCCTGCTCACAGAACACGACGTCGGCCTCTCGGCGGGCGCCGCCTCGCTGGGCCACCTTGCCGCGGCGGCGAGGGCCACCAAACAGTTCCTCGCCCCGTCGTCCTCAACCCCGTTCAACTGGCTCGAGGCGTCAACCGG

>82_*NAM-1*

GCTTTTTATTATACTGTGCACAAGTATTTTTATATTCTTCCAGTAAGTACAGCGCATGTATGTGATCCTGTCGTCGTGCTTGTTCATGCGCTCGGGCGGGATCATCATCCATCAGAGAAGGCGACCTTCGGGGAGCACGAGTGGTACTTCTTCAGCCCGCGCGACCGCAAGTACGCCAACGGCGCGCGGCCGAACCGGGCGGCGACGTCGGGCTACTGGAAGGCCACCGGCACGGACAAGCCTATCCTGGCCTCGGCCACCGGGTGCGGCCGGGAGAAGGTCGGCGTCAAGAAGGCGCTCGTCTTCTACCGCGGGAAGCCGCCCAGGGGCCTCAAGACCAACTGGATCATGCATGAGTACCGCCTCACCGGAGCCTCTGCTGGCTCCACCACCACCAGCCGGCCGCCGCCGGTGACCGGCGGGAGCAGGGCCCCGGCCTCTCTCAGGGTACGTACTTACACGTGTCCATCGCACGGTCTATCAGTATTTATTTATTAACTACTCTCGAGCTTAATTATGGTATTGTTGATAGTTGATGAAGTTAATTATTGTACGCCGTCTCATCGATCAGTTGGACGACTGGGTGCTGTGCCGCATCTACAAGAAGACCAGCAAGGCCGCGGCCGCGGTCGGAGATGAGCAGAGGAGCATGGAGTGCGAGGACTCCGTGGAGGACGCGGTCACCGCGTACCCGCCCTACGCCACGGCGGGCATGGCCGGCGCAGGTGCGCATGGCAGCAACTACGTTCAACTGCTCCATCATCACGACAGCCACGAGGACAACTTCCAGCTAGACGGCCTGCTCACAGAACACGACGTCGGCCTCTCGGCGGGCGCCGCCTCGCTGGGCCACCTTGCCGCGGCGGCGAGGGCCACCAAACAGTTCCTCGCCCCGTCGTCCTCAACCCCGTTCAACTGGCTCGAGGCGTCAACCGG

>83_*NAM-1*

GCTTTTTATTATACTGTGCACAAGTATTTTTATATTCTTCCAGTAAGTACAGCGCATGTATGTGATCCTGTCGTCGTGCTTGTTCATGCGCTCGGGCGGGATCATCATCCATCAGAGAAGGCGACCTTCGGGGAGCACGAGTGGTACTTCTTCAGCCCGCGCGACCGCAAGTACCCCAACGGCGCGCGGCCGAACCGGGCGGCGACGTCGGGCTACTGGAAGGCCACCGGCACGGACAAGCCTATCCTGGCCTCGGCCACCGGGTGCGGCCGGGAGAAGGTCGGCGTCAAGAAGGCGCTCGTCTTCTACCGCGGGAAGCCGCCCAGGGGCCTCAAGACCAACTGGATCATGCATGAGTACCGCCTCACCGGAGCCTCTGCTGGCTCCACCACCACCAGCCGGCCGCCGCCGGTGACCGGCGGGAGCAGGGCCCCGGCCTCTCTCAGGGTACGTACTTACACGTGTCCATCGCACGGTCTATCAGTATTTATTTATTAACTACTCTCGAGCTTAATTATGGTATTGTTGATAGTTGATGAAGTTAATTATTGTACGCCGTCTCATCGATCAGTTGGACGACTGGGTGCTGTGCCGCATCTACAAGAAGACCAGCAAGGCCGCGGCCGCGGTCGGAGATGAGCAGAGGAGCATGGAGTGCGAGGACTCCGTGGAGGACGCGGTCACCGCGTACCCGCCCTACGCCACGGCGGGCATGGCCGGCGCAGGTGCGCATGGCAGCAACTACGTTCAACTGCTCCATCATCACGACAGCCACGAGGACAACTTCCAGCTAGACGGCCTGCTCACAGAACACGACGTCGGCCTCTCGGCGGGCGCCGCCTCGCTGGGCCACCTTGCCGCGGCGGCGAGGGCCACCAAACAGTTCCTCGCCCCGTCGTCCTCAACCCCGTTCAACTGGCTCGAGGCGTCAACCGG

>84_*NAM-1*

GCTTTTTATTATACTGTGCACAAGTATTTTTATATTCTTCCAGTAAGTACAGCGCATGTATGTGATCCTGTCGTCGTGCTTGTTCATGCGCTCGGGCGGGATCATCATCCATCAGAGAAGGCGACCTTCGGGGAGCACGAGTGGTACTTCTTCAGCCCGCGCGACCGCAAGTACGCCAACGGCGCGCGGCCGAACCGGGCGGCGACGTCGGGCTACTGGAAGGCCACCGGCACGGACAAGCCTATCCTGGCCTCGGCCACCGGGTGCGGCCGGGAGAAGGTCGGCGTCAAGAAGGCGCTCGTCTTCTACCGCGGGAAGCCGCCCAGGGGCCTCAAGACCAACTGGATCATGCATGAGTACCGCCTCACCGGAGCCTCTGCTGGCTCCACCACCACCAGCCGGCCGCCGCCGGTGACCGGCGGGAGCAGGGCCCCGGCCTCTCTCAGGGTACGTACTTACACGTGTCCATCGCACGGTCTATCAGTATTTATTTATTAACTACTCTCGAGCTTAATTATGGTATTGTTGATAGTTGATGAAGTTAATTATTGTACGCCGTCTCATCGATCAGTTGGACGACTGGGTGCTGTGCCGCATCTACAAGAAGACCAGCAAGGCCGCGGCCGCGGTCGGAGATGAGCAGAGGAGCATGGAGTGCGAGGACTCCGTGGAGGACGCGGTCACCGCGTACCCGCCCTACGCCACGGCGGGCATGGCCGGCGCAGGTGCGCATGGCAGCAACTACGTTCAACTGCTCCATCATCACGACAGCCACGAGGACAACTTCCAGCTAGACGGCCTGCTCACAGAACACGACGTCGGCCTCTCGGCGGGCGCCGCCTCGCTGGGCCACCTTGCCGCGGCGGCGAGGGCCACCAAACAGTTCCTCGCCCCGTCGTCCTCAACCCCGTTCAACTGGCTCGAGGCGTCAACCGG

>85_*NAM-1*

GCTTTTTATTATACTGTGCACAAGTATTTTTATATTCTTCCAGTAAGTACAGCGCATGTATGTGATCCTGTCGTCGTGCTTGTTCATGCGCTCGGGCGGGATCATCATCCATCAGAGAAGGCGACCTTCGGGGAGCACGAGTGGTACTTCTTCAGCCCGCGCGACCGCAAGTACCCCAACGGCGCGCGGCCGAACCGGGCGGCGACGTCGGGCTACTGGAAGGCCACCGGCACGGACAAGCCTATCCTGGCCTCGGCCACCGGGTGCGGCCGGGAGAAGGTCGGCGTCAAGAAGGCGCTCGTCTTCTACCGCGGGAAGCCGCCCAGGGGCCTCAAGACCAACTGGATCATGCATGAGTACCGCCTCACCGGAGCCTCTGCTGGCTCCACCACCACCAGCCGGCCGCCGCCGGTGACCGGCGGGAGCAGGGCCCCGGCCTCTCTCAGGGTACGTACTTACACGTGTCCATCGCACGGTCTATCAGTATTTATTTATTAACTACTCTCGAGCTTAATTATGGTATTGTTGATAGTTGATGAAGTTAATTATTGTACGCCGTCTCATCGATCAGTTGGACGACTGGGTGCTGTGCCGCATCTACAAGAAGACCAGCAAGGCCGCGGCCGCGGTCGGAGATGAGCAGAGGAGCATGGAGTGCGAGGACTCCGTGGAGGACGCGGTCACCGCGTACCCGCCCTACGCCACGGCGGGCATGGCCGGCGCAGGTGCGCATGGCAGCAACTACGTTCAACTGCTCCATCATCACGACAGCCACGAGGACAACTTCCAGCTAGACGGCCTGCTCACAGAACACGACGTCGGCCTCTCGGCGGGCGCCGCCTCGCTGGGCCACCTTGCCGCGGCGGCGAGGGCCACCAAACAGTTCCTCGCCCCGTCGTCCTCAACCCCGTTCAACTGGCTCGAGGCGTCAACCGG

>86_*NAM-1*

GCTTTTTATTATACTGTGCACAAGTATTTTTATATTCTTCCAGTAAGTACAGCGCATGTATGTGATCCTGTCGTCGTGCTTGTTCATGCGCTCGGGCGGGATCATCATCCATCAGAGAAGGCGACCTTCGGGGAGCACGAGTGGTACTTCTTCAGCCCGCGCGACCGCAAGTACCCCAACGGCGCGCGGCCGAACCGGGCGGCGACGTCGGGCTACTGGAAGGCCACCGGCACGGACAAGCCTATCCTGGCCTCGGCCACCGGGTGCGGCCGGGAGAAGGTCGGCGTCAAGAAGGCGCTCGTCTTCTACCGCGGGAAGCCGCCCAGGGGCCTCAAGACCAACTGGATCATGCATGAGTACCGCCTCACCGGAGCCTCTGCTGGCTCCACCACCACCAGCCGGCCGCCGCCGGTGACCGGCGGGAGCAGGGCCCCGGCCTCTCTCAGGGTACGTACTTACACGTGTCCATCGCACGGTCTATCAGTATTTATTTATTAACTACTCTCGAGCTTAATTATGGTATTGTTGATAGTTGATGAAGTTAATTATTGTACGCCGTCTCATCGATCAGTTGGACGACTGGGTGCTGTGCCGCATCTACAAGAAGACCAGCAAGGCCGCGGCCGCGGTCGGAGATGAGCAGAGGAGCATGGAGTGCGAGGACTCCGTGGAGGACGCGGTCACCGCGTACCCGCCCTACGCCACGGCGGGCATGGCCGGCGCAGGTGCGCATGGCAGCAACTACGTTCAACTGCTCCATCATCACGACAGCCACGAGGACAACTTCCAGCTAGACGGCCTGCTCACAGAACACGACGTCGGCCTCTCGGCGGGCGCCGCCTCGCTGGGCCACCTTGCCGCGGCGGCGAGGGCCACCAAACAGTTCCTCGCCCCGTCGTCCTCAACCCCGTTCAACTGGCTCGAGGCGTCAACCGG

>87_*NAM-1*

GCTTTTTATTATACTGTGCACAAGTATTTTTATATTCTTCCAGTAAGTACAGCGCATGTATGTGATCCTGTCGTCGTGCTTGTTCATGCGCTCGGGCGGGATCATCATCCATCAGAGAAGGCGACCTTCGGGGAGCACGAGTGGTACTTCTTCAGCCCGCGCGACCGCAAGTACCCCAACGGCGCGCGGCCGAACCGGGCGGCGACGTCGGGCTACTGGAAGGCCACCGGCACGGACAAGCCTATCCTGGCCTCGGCCACCGGGTGCGGCCGGGAGAAGGTCGGCGTCAAGAAGGCGCTCGTCTTCTACCGCGGGAAGCCGCCCAGGGGCCTCAAGACCAACTGGATCATGCATGAGTACCGCCTCACCGGAGCCTCTGCTGGCTCCACCACCACCAGCCGGCCGCCGCCGGTGACCGGCGGGAGCAGGGCCCCGGCCTCTCTCAGGGTACGTACTTACACGTGTCCATCGCACGGTCTATCAGTATTTATTTATTAACTACTCTCGAGCTTAATTATGGTATTGTTGATAGTTGATGAAGTTAATTATTGTACGCCGTCTCATCGATCAGTTGGACGACTGGGTGCTGTGCCGCATCTACAAGAAGACCAGCAAGGCCGCGGCCGCGGTCGGAGATGAGCAGAGGAGCATGGAGTGCGAGGACTCCGTGGAGGACGCGGTCACCGCGTACCCGCCCTACGCCACGGCGGGCATGGCCGGCGCAGGTGCGCATGGCAGCAACTACGTTCAACTGCTCCATCATCACGACAGCCACGAGGACAACTTCCAGCTAGACGGCCTGCTCACAGAACACGACGTCGGCCTCTCGGCGGGCGCCGCCTCGCTGGGCCACCTTGCCGCGGCGGCGAGGGCCACCAAACAGTTCCTCGCCCCGTCGTCCTCAACCCCGTTCAACTGGCTCGAGGCGTCAACCGG

>98_*NAM-1*

GCTTTTTATTATACTGTGCACAAGTATTTTTATATTCTTCCAGTAAGTACAGCGCATGTATGTGATCCTGTCGTCGTGCTTGTTCATGCGCTCGGGCGGGATCATCATCCATCAGAGAAGGCGACCTTCGGGGAGCACGAGTGGTACTTCTTCAGCCCGCGCGACCGCAAGTACGCCAACGGCGCGCGGCCGAACCGGGCGGCGACGTCGGGCTACTGGAAGGCCACCGGCACGGACAAGCCTATCCTGGCCTCGGCCACCGGGTGCGGCCGGGAGAAGGTCGGCGTCAAGAAGGCGCTCGTCTTCTACCGCGGGAAGCCGCCCAGGGGCCTCAAGACCAACTGGATCATGCATGAGTACCGCCTCACCGGAGCCTCTGCTGGCTCCACCACCACCAGCCGGCCGCCGCCGGTGACCGGCGGGAGCAGGGCCCCGGCCTCTCTCAGGGTACGTACTTACACGTGTCCATCGCACGGTCTATCAGTATTTATTTATTAACTACTCTCGAGCTTAATTATGGTATTGTTGATAGTTGATGAAGTTAATTATTGTACGCCGTCTCATCGATCAGTTGGACGACTGGGTGCTGTGCCGCATCTACAAGAAGACCAGCAAGGCCGCGGCCGCGGTCGGAGATGAGCAGAGGAGCATGGAGTGCGAGGACTCCGTGGAGGACGCGGTCACCGCGTACCCGCCCTACGCCACGGCGGGCATGGCCGGCGCAGGTGCGCATGGCAGCAACTACGTTCAACTGCTCCATCATCACGACAGCCACGAGGACAACTTCCAGCTAGACGGCCTGCTCACAGAACACGACGTCGGCCTCTCGGCGGGCGCCGCCTCGCTGGGCCACCTTGCCGCGGCGGCGAGGGCCACCAAACAGTTCCTCGCCCCGTCGTCCTCAACCCCGTTCAACTGGCTCGAGGCGTCAACCGG

>99_*NAM-1*

GCTTTTTATTATACTGTGCACAAGTATTTTTATATTCTTCCAGTAAGTACAGCGCATGTATGTGATCCTGTCGTCGTGCTTGTTCATGCGCTCGGGCGGGATCATCATCCATCAGAGAAGGCGACCTTCGGGGAGCACGAGTGGTACTTCTTCAGCCCGCGCGACCGCAAGTACGCCAACGGCGCGCGGCCGAACCGGGCGGCGACGTCGGGCTACTGGAAGGCCACCGGCACGGACAAGCCTATCCTGGCCTCGGCCACCGGGTGCGGCCGGGAGAAGGTCGGCGTCAAGAAGGCGCTCGTCTTCTACCGCGGGAAGCCGCCCAGGGGCCTCAAGACCAACTGGATCATGCATGAGTACCGCCTCACCGGAGCCTCTGCTGGCTCCACCACCACCAGCCGGCCGCCGCCGGTGACCGGCGGGAGCAGGGCCCCGGCCTCTCTCAGGGTACGTACTTACACGTGTCCATCGCACGGTCTATCAGTATTTATTTATTAACTACTCTCGAGCTTAATTATGGTATTGTTGATAGTTGATGAAGTTAATTATTGTACGCCGTCTCATCGATCAGTTGGACGACTGGGTGCTGTGCCGCATCTACAAGAAGACCAGCAAGGCCGCGGCCGCGGTCGGAGATGAGCAGAGGAGCATGGAGTGCGAGGACTCCGTGGAGGACGCGGTCACCGCGTACCCGCCCTACGCCACGGCGGGCATGGCCGGCGCAGGTGCGCATGGCAGCAACTACGTTCAACTGCTCCATCATCACGACAGCCACGAGGACAACTTCCAGCTAGACGGCCTGCTCACAGAACACGACGTCGGCCTCTCGGCGGGCGCCGCCTCGCTGGGCCACCTTGCCGCGGCGGCGAGGGCCACCAAACAGTTCCTCGCCCCGTCGTCCTCAACCCCGTTCAACTGGCTCGAGGCGTCAACCGG

>140_*NAM-1*

GCTTTTTATTATACTGTGCACAAGTATTTTTATATTCTTCCAGTAAGTACAGCGCATGTATGTGATCCTGTCGTCGTGCTTGTTCATGCGCTCGGGCGGGATCGTCATCCATCAGAGAAGGCGACCTTCGGGGAGCACGAGTGGTACTTCTTCAGCCCGCGCGACCGCAAGTACCCCAACGGCGCGCGGCCGAACCGGGCGGCGACGTCGGGCTACTGGAAGGCCACCGGCACGGACAAGCCTATCCTGGCCTCGGCCACCGGGTGCGGCCGGGAGAAGGTCGGCGTCAAGAAGGCGCTCGTCTTCTACCGCGGGAAGCCGCCCAGGGGCCTCAAGACCAACTGGATCATGCATGAGTACCGCCTCACCGGAGCCTCTGCTGGCTCCACCACCACCAGCCGGCCGCCGCCGGTGACCGGCGGGAGCAGGGCCCCGGCCTCTCTCAGGGTACGTACTTACACGTGTCCATCGCACGGTCTATCAGTATTTATTTATTAACTACTCTCGAGCTTAATTATGGTATTGTTGATAGTTGATGAAGTTAATTATTGTACGCCGTCTCATCGATCAGTTGGACGACTGGGTGCTGTGCCGCATCTACAAGAAGACCAGCAAGGCCGCGGCCGCGGTCGGAGATGAGCAGAGGAGCATGGAGTGCGAGGACTCCGTGGAGGACGCGGTCACCGCGTACCCGCCCTACGCCACGGCGGGCATGGCCGGCGCAGGTGCGCATGGCAGCAACTACGTTCAACTGCTCCATCATCACGACAGCCACGAGGACAACTTCCAGCTAGACGGCCTGCTCACAGAACACGACGTCGGCCTCTCGGCGGGCGCCGCCTCGCTGGGCCACCTTGCCGCGGCGGCGAGGGCCACCAAACAGTTCCTCGCCCCGTCGTCCTCAACCCCGTTCAACTGGCTCGAGGCGTCAACCGG

>141_*NAM-1*

GCTTTTTATTATACTGTGCACAAGTATTTTTATATTCTTCCAGTAAGTACAGCGCATGTATGTGATCCTGTCGTCGTGCTTGTTCATGCGCTCGGGCGGGATCATCATCCATCAGAGAAGGCGACCTTCGGGGAGCACGAGTGGTACTTCTTCAGCCCGCGCGACCGCAAGTACCCCAACGGCGCGCGGCCGAACCGGGCGGCGACGTCGGGCTACTGGAAGGCCACCGGCACGGACAAGCCTATCCTGGCCTCGGCCACCGGGTGCGGCCGGGAGAAGGTCGGCGTCAAGAAGGCGCTCGTCTTCTACCGCGGGAAGCCGCCCAGGGGCCTCAAGACCAACTGGATCATGCATGAGTACCGCCTCACCGGAGCCTCTGCTGGCTCCACCACCACCAGCCGGCCGCCGCCGGTGACCGGCGGGAGCAGGGCCCCGGCCTCTCTCAGGGTACGTACTTACACGTGTCCATCGCACGGTCTATCAGTATTTATTTATTAACTACTCTCGAGCTTAATTATGGTATTGTTGATAGTTGATGAAGTTAATTATTGTACGCCGTCTCATCGATCAGTTGGACGACTGGGTGCTGTGCCGCATCTACAAGAAGACCAGCAAGGCCGCGGCCGCGGTCGGAGATGAGCAGAGGAGCATGGAGTGCGAGGACTCCGTGGAGGACGCGGTCACCGCGTACCCGCCCTACGCCACGGCGGGCATGGCCGGCGCAGGTGCGCATGGCAGCAACTACGTTCAACTGCTCCATCATCACGACAGCCACGAGGACAACTTCCAGCTAGACGGCCTGCTCACAGAACACGACGTCGGCCTCTCGGCGGGCGCCGCCTCGCTGGGCCACCTTGCCGCGGCGGCGAGGGCCACCAAACAGTTCCTCGCCCCGTCGTCCTCAACCCCGTTCAACTGGCTCGAGGCGTCAACCGG

>142_*NAM-1*

GCTTTTTATTATACTGTGCACAAGTATTTTTATATTCTTCCAGTAAGTACAGCGCATGTATGTGATCCTGTCGTCGTGCTTGTTCATGCGCTCGGGCGGGATCATCATCCATCAGAGAAGGCGACCTTCGGGGAGCACGAGTGGTACTTCTTCAGCCCGCGCGACCGCAAGTACGCCAACGGCGCGCGGCCGAACCGGGCGGCGACGTCGGGCTACTGGAAGGCCACCGGCACGGACAAGCCTATCCTGGCCTCGGCCACCGGGTGCGGCCGGGAGAAGGTCGGCGTCAAGAAGGCGCTCGTCTTCTACCGCGGGAAGCCGCCCAGGGGCCTCAAGACCAACTGGATCATGCATGAGTACCGCCTCACCGGAGCCTCTGCTGGCTCCACCACCACCAGCCGGCCGCCGCCGGTGACCGGCGGGAGCAGGGCCCCGGCCTCTCTCAGGGTACGTACTTACACGTGTCCATCGCACGGTCTATCAGTATTTATTTATTAACTACTCTCGAGCTTAATTATGGTATTGTTGATAGTTGATGAAGTTAATTATTGTACGCCGTCTCATCGATCAGTTGGACGACTGGGTGCTGTGCCGCATCTACAAGAAGACCAGCAAGGCCGCGGCCGCGGTCGGAGATGAGCAGAGGAGCATGGAGTGCGAGGACTCCGTGGAGGACGCGGTCACCGCGTACCCGCCCTACGCCACGGCGGGCATGGCCGGCGCAGGTGCGCATGGCAGCAACTACGTTCAACTGCTCCATCATCACGACAGCCACGAGGACAACTTCCAGCTAGACGGCCTGCTCACAGAACACGACGTCGGCCTCTCGGCGGGCGCCGCCTCGCTGGGCCACCTTGCCGCGGCGGCGAGGGCCACCAAACAGTTCCTCGCCCCGTCGTCCTCAACCCCGTTCAACTGGCTCGAGGCGTCAACCGG

>143_*NAM-1*

GCTTTTTATTATACTGTGCACAAGTATTTTTATATTCTTCCAGTAAGTACAGCGCATGTATGTGATCCTGTCGTCGTGCTTGTTCATGCGCTCGGGCGGGATCATCATCCATCAGAGAAGGCGACCTTCGGGGAGCACGAGTGGTACTTCTTCAGCCCGCGCGACCGCAAGTACGCCAACGGCGCGCGGCCGAACCGGGCGGCGACGTCGGGCTACTGGAAGGCCACCGGCACGGACAAGCCTATCCTGGCCTCGGCCACCGGGTGCGGCCGGGAGAAGGTCGGCGTCAAGAAGGCGCTCGTCTTCTACCGCGGGAAGCCGCCCAGGGGCCTCAAGACCAACTGGATCATGCATGAGTACCGCCTCACCGGAGCCTCTGCTGGCTCCACCACCACCAGCCGGCCGCCGCCGGTGACCGGCGGGAGCAGGGCCCCGGCCTCTCTCAGGGTACGTACTTACACGTGTCCATCGCACGGTCTATCAGTATTTATTTATTAACTACTCTCGAGCTTAATTATGGTATTGTTGATAGTTGATGAAGTTAATTATTGTACGCCGTCTCATCGATCAGTTGGACGACTGGGTGCTGTGCCGCATCTACAAGAAGACCAGCAAGGCCGCGGCCGCGGTCGGAGATGAGCAGAGGAGCATGGAGTGCGAGGACTCCGTGGAGGACGCGGTCACCGCGTACCCGCCCTACGCCACGGCGGGCATGGCCGGCGCAGGTGCGCATGGCAGCAACTACGTTCAACTGCTCCATCATCACGACAGCCACGAGGACAACTTCCAGCTAGACGGCCTGCTCACAGAACACGACGTCGGCCTCTCGGCGGGCGCCGCCTCGCTGGGCCACCTTGCCGCGGCGGCGAGGGCCACCAAACAGTTCCTCGCCCCGTCGTCCTCAACCCCGTTCAACTGGCTCGAGGCGTCAACCGG

>144_*NAM-1*

GCTTTTTATTATACTGTGCACAAGTATTTTTATATTCTTCCAGTAAGTACAGCGCATGTATGTGATCCTGTCGTCGTGCTTGTTCATGCGCTCGGGCGGGATCATCATCCATCAGAGAAGGCGACCTTCGGGGAGCACGAGTGGTACTTCTTCAGCCCGCGCGACCGCAAGTACGCCAACGGCGCGCGGCCGAACCGGGCGGCGACGTCGGGCTACTGGAAGGCCACCGGCACGGACAAGCCTATCCTGGCCTCGGCCACCGGGTGCGGCCGGGAGAAGGTCGGCGTCAAGAAGGCGCTCGTCTTCTACCGCGGGAAGCCGCCCAGGGGCCTCAAGACCAACTGGATCATGCATGAGTACCGCCTCACCGGAGCCTCTGCTGGCTCCACCACCACCAGCCGGCCGCCGCCGGTGACCGGCGGGAGCAGGGCCCCGGCCTCTCTCAGGGTACGTACTTACACGTGTCCATCGCACGGTCTATCAGTATTTATTTATTAACTACTCTCGAGCTTAATTATGGTATTGTTGATAGTTGATGAAGTTAATTATTGTACGCCGTCTCATCGATCAGTTGGACGACTGGGTGCTGTGCCGCATCTACAAGAAGACCAGCAAGGCCGCGGCCGCGGTCGGAGATGAGCAGAGGAGCATGGAGTGCGAGGACTCCGTGGAGGACGCGGTCACCGCGTACCCGCCCTACGCCACGGCGGGCATGGCCGGCGCAGGTGCGCATGGCAGCAACTACGTTCAACTGCTCCATCATCACGACAGCCACGAGGACAACTTCCAGCTAGACGGCCTGCTCACAGAACACGACGTCGGCCTCTCGGCGGGCGCCGCCTCGCTGGGCCACCTTGCCGCGGCGGCGAGGGCCACCAAACAGTTCCTCGCCCCGTCGTCCTCAACCCCGTTCAACTGGCTCGAGGCGTCAACCGG

>146_*NAM-1*

GCTTTTTATTATACTGTGCACAAGTATTTTTATATTCTTCCAGTAAGTACAGCGCATGTATGTGATCCTGTCGTCGTGCTTGTTCATGCGCTCGGGCGGGATCATCATCCATCAGAGAAGGCGACCTTCGGGGAGCACGAGTGGTACTTCTTCAGCCCGCGCGACCGCAAGTACGCCAACGGCGCGCGGCCGAACCGGGCGGCGACGTCGGGCTACTGGAAGGCCACCGGCACGGACAAGCCTATCCTGGCCTCGGCCACCGGGTGCGGCCGGGAGAAGGTCGGCGTCAAGAAGGCGCTCGTCTTCTACCGCGGGAAGCCGCCCAGGGGCCTCAAGACCAACTGGATCATGCATGAGTACCGCCTCACCGGAGCCTCTGCTGGCTCCACCACCACCAGCCGGCCGCCGCCGGTGACCGGCGGGAGCAGGGCCCCGGCCTCTCTCAGGGTACGTACTTACACGTGTCCATCGCACGGTCTATCAGTATTTATTTATTAACTACTCTCGAGCTTAATTATGGTATTGTTGATAGTTGATGAAGTTAATTATTGTACGCCGTCTCATCGATCAGTTGGACGACTGGGTGCTGTGCCGCATCTACAAGAAGACCAGCAAGGCCGCGGCCGCGGTCGGAGATGAGCAGAGGAGCATGGAGTGCGAGGACTCCGTGGAGGACGCGGTCACCGCGTACCCGCCCTACGCCACGGCGGGCATGGCCGGCGCAGGTGCGCATGGCAGCAACTACGTTCAACTGCTCCATCATCACGACAGCCACGAGGACAACTTCCAGCTAGACGGCCTGCTCACAGAACACGACGTCGGCCTCTCGGCGGGCGCCGCCTCGCTGGGCCACCTTGCCGCGGCGGCGAGGGCCACCAAACAGTTCCTCGCCCCGTCGTCCTCAACCCCGTTCAACTGGCTCGAGGCGTCAACCGG

>147_*NAM-1*

GCTTTTTATTATACTGTGCACAAGTATTTTTATATTCTTCCAGTAAGTACAGCGCATGTATGTGATCCTGTCGTCGTGCTTGTTCATGCGCTCGGGCGGGATCATCATCCATCAGAGAAGGCGACCTTCGGGGAGCACGAGTGGTACTTCTTCAGCCCGCGCGACCGCAAGTACGCCAACGGCGCGCGGCCGAACCGGGCGGCGACGTCGGGCTACTGGAAGGCCACCGGCACGGACAAGCCTATCCTGGCCTCGGCCACCGGGTGCGGCCGGGAGAAGGTCGGCGTCAAGAAGGCGCTCGTCTTCTACCGCGGGAAGCCGCCCAGGGGCCTCAAGACCAACTGGATCATGCATGAGTACCGCCTCACCGGAGCCTCTGCTGGCTCCACCACCACCAGCCGGCCGCCGCCGGTGACCGGCGGGAGCAGGGCCCCGGCCTCTCTCAGGGTACGTACTTACACGTGTCCATCGCACGGTCTATCAGTATTTATTTATTAACTACTCTCGAGCTTAATTATGGTATTGTTGATAGTTGATGAAGTTAATTATTGTACGCCGTCTCATCGATCAGTTGGACGACTGGGTGCTGTGCCGCATCTACAAGAAGACCAGCAAGGCCGCGGCCGCGGTCGGAGATGAGCAGAGGAGCATGGAGTGCGAGGACTCCGTGGAGGACGCGGTCACCGCGTACCCGCCCTACGCCACGGCGGGCATGGCCGGCGCAGGTGCGCATGGCAGCAACTACGTTCAACTGCTCCATCATCACGACAGCCACGAGGACAACTTCCAGCTAGACGGCCTGCTCACAGAACACGACGTCGGCCTCTCGGCGGGCGCCGCCTCGCTGGGCCACCTTGCCGCGGCGGCGAGGGCCACCAAACAGTTCCTCGCCCCGTCGTCCTCAACCCCGTTCAACTGGCTCGAGGCGTCAACCGG

>148_*NAM-1*

GCTTTTTATTATACTGTGCACAAGTATTTTTATATTCTTCCAGTAAGTACAGCGCATGTATGTGATCCTGTCGTCGTGCTTGTTCATGCGCTCGGGCGGGATCATCATCCATCAGAGAAGGCGACCTTCGGGGAGCACGAGTGGTACTTCTTCAGCCCGCGCGACCGCAAGTACGCCAACGGCGCGCGGCCGAACCGGGCGGCGACGTCGGGCTACTGGAAGGCCACCGGCACGGACAAGCCTATCCTGGCCTCGGCCACCGGGTGCGGCCGGGAGAAGGTCGGCGTCAAGAAGGCGCTCGTCTTCTACCGCGGGAAGCCGCCCAGGGGCCTCAAGACCAACTGGATCATGCATGAGTACCGCCTCACCGGAGCCTCTGCTGGCTCCACCACCACCAGCCGGCCGCCGCCGGTGACCGGCGGGAGCAGGGCCCCGGCCTCTCTCAGGGTACGTACTTACACGTGTCCATCGCACGGTCTATCAGTATTTATTTATTAACTACTCTCGAGCTTAATTATGGTATTGTTGATAGTTGATGAAGTTAATTATTGTACGCCGTCTCATCGATCAGTTGGACGACTGGGTGCTGTGCCGCATCTACAAGAAGACCAGCAAGGCCGCGGCCGCGGTCGGAGATGAGCAGAGGAGCATGGAGTGCGAGGACTCCGTGGAGGACGCGGTCACCGCGTACCCGCCCTACGCCACGGCGGGCATGGCCGGCGCAGGTGCGCATGGCAGCAACTACGTTCAACTGCTCCATCATCACGACAGCCACGAGGACAACTTCCAGCTAGACGGCCTGCTCACAGAACACGACGTCGGCCTCTCGGCGGGCGCCGCCTCGCTGGGCCACCTTGCCGCGGCGGCGAGGGCCACCAAACAGTTCCTCGCCCCGTCGTCCTCAACCCCGTTCAACTGGCTCGAGGCGTCAACCGG

>149_*NAM-1*

GCTTTTTATTATACTGTGCACAAGTATTTTTATATTCTTCCAGTAAGTACAGCGCATGTATGTGATCCTGTCGTCGTGCTTGTTCATGCGCTCGGGCGGGATCATCATCCATCAGAGAAGGCGACCTTCGGGGAGCACGAGTGGTACTTCTTCAGCCCGCGCGACCGCAAGTACGCCAACGGCGCGCGGCCGAACCGGGCGGCGACGTCGGGCTACTGGAAGGCCACCGGCACGGACAAGCCTATCCTGGCCTCGGCCACCGGGTGCGGCCGGGAGAAGGTCGGCGTCAAGAAGGCGCTCGTCTTCTACCGCGGGAAGCCGCCCAGGGGCCTCAAGACCAACTGGATCATGCATGAGTACCGCCTCACCGGAGCCTCTGCTGGCTCCACCACCACCAGCCGGCCGCCGCCGGTGACCGGCGGGAGCAGGGCCCCGGCCTCTCTCAGGGTACGTACTTACACGTGTCCATCGCACGGTCTATCAGTATTTATTTATTAACTACTCTCGAGCTTAATTATGGTATTGTTGATAGTTGATGAAGTTAATTATTGTACGCCGTCTCATCGATCAGTTGGACGACTGGGTGCTGTGCCGCATCTACAAGAAGACCAGCAAGGCCGCGGCCGCGGTCGGAGATGAGCAGAGGAGCATGGAGTGCGAGGACTCCGTGGAGGACGCGGTCACCGCGTACCCGCCCTACGCCACGGCGGGCATGGCCGGCGCAGGTGCGCATGGCAGCAACTACGTTCAACTGCTCCATCATCACGACAGCCACGAGGACAACTTCCAGCTAGACGGCCTGCTCACAGAACACGACGTCGGCCTCTCGGCGGGCGCCGCCTCGCTGGGCCACCTTGCCGCGGCGGCGAGGGCCACCAAACAGTTCCTCGCCCCGTCGTCCTCAACCCCGTTCAACTGGCTCGAGGCGTCAACCGG

>175_*NAM-1*

GCTTTTTATTATACTGTGCACAAGTATTTTTATATTCTTCCAGTAAGTACAGCGCATGTATGTGATCCTGTCGTCGTGCTTGTTCATGCGCTCGGGCGGGATCATCATCCATCAGAGAAGGCGACCTTCGGGGAGCACGAGTGGTACTTCTTCAGCCCGCGCGACCGCAAGTACGCCAACGGCGCGCGGCCGAACCGGGCGGCGACGTCGGGCTACTGGAAGGCCACCGGCACGGACAAGCCTATCCTGGCCTCGGCCACCGGGTGCGGCCGGGAGAAGGTCGGCGTCAAGAAGGCGCTCGTCTTCTACCGCGGGAAGCCGCCCAGGGGCCTCAAGACCAACTGGATCATGCATGAGTACCGCCTCACCGGAGCCTCTGCTGGCTCCACCACCACCAGCCGGCCGCCGCCGGTGACCGGCGGGAGCAGGGCCCCGGCCTCTCTCAGGGTACGTACTTACACGTGTCCATCGCACGGTCTATCAGTATTTATTTATTAACTACTCTCGAGCTTAATTATGGTATTGTTGATAGTTGATGAAGTTAATTATTGTACGCCGTCTCATCGATCAGTTGGACGACTGGGTGCTGTGCCGCATCTACAAGAAGACCAGCAAGGCCGCGGCCGCGGTCGGAGATGAGCAGAGGAGCATGGAGTGCGAGGACTCCGTGGAGGACGCGGTCACCGCGTACCCGCCCTACGCCACGGCGGGCATGGCCGGCGCAGGTGCGCATGGCAGCAACTACGTTCAACTGCTCCATCATCACGACAGCCACGAGGACAACTTCCAGCTAGACGGCCTGCTCACAGAACACGACGTCGGCCTCTCGGCGGGCGCCGCCTCGCTGGGCCACCTTGCCGCGGCGGCGAGGGCCACCAAACAGTTCCTCGCCCCGTCGTCCTCAACCCCGTTCAACTGGCTCGAGGCGTCAACCGG

>189_*NAM-1*

GCTTTTTATTATACTGTGCACAAGTATTTTTATATTCTTCCAGTAAGTACAGCGCATGTATGTGATCCTGTCGTCGTGCTTGTTCATGCGCTCGGGCGGGATCATCATCCATCAGAGAAGGCGACCTTCGGGGAGCACGAGTGGTACTTCTTCAGCCCGCGCGACCGCAAGTACGCCAACGGCGCGCGGCCGAACCGGGCGGCGACGTCGGGCTACTGGAAGGCCACCGGCACGGACAAGCCTATCCTGGCCTCGGCCACCGGGTGCGGCCGGGAGAAGGTCGGCGTCAAGAAGGCGCTCGTCTTCTACCGCGGGAAGCCGCCCAGGGGCCTCAAGACCAACTGGATCATGCATGAGTACCGCCTCACCGGAGCCTCTGCTGGCTCCACCACCACCAGCCGGCCGCCGCCGGTGACCGGCGGGAGCAGGGCCCCGGCCTCTCTCAGGGTACGTACTTACACGTGTCCATCGCACGGTCTATCAGTATTTATTTATTAACTACTCTCGAGCTTAATTATGGTATTGTTGATAGTTGATGAAGTTAATTATTGTACGCCGTCTCATCGATCAGTTGGACGACTGGGTGCTGTGCCGCATCTACAAGAAGACCAGCAAGGCCGCGGCCGCGGTCGGAGATGAGCAGAGGAGCATGGAGTGCGAGGACTCCGTGGAGGACGCGGTCACCGCGTACCCGCCCTACGCCACGGCGGGCATGGCCGGCGCAGGTGCGCATGGCAGCAACTACGTTCAACTGCTCCATCATCACGACAGCCACGAGGACAACTTCCAGCTAGACGGCCTGCTCACAGAACACGACGTCGGCCTCTCGGCGGGCGCCGCCTCGCTGGGCCACCTTGCCGCGGCGGCGAGGGCCACCAAACAGTTCCTCGCCCCGTCGTCCTCAACCCCGTTCAACTGGCTCGAGGCGTCAACCGG

>190_*NAM-1*

GCTTTTTATTATACTGTGCACAAGTATTTTTATATTCTTCCAGTAAGTACAGCGCATGTATGTGATCCTGTCGTCGTGCTTGTTCATGCGCTCGGGCGGGATCATCATCCATCAGAGAAGGCGACCTTCGGGGAGCACGAGTGGTACTTCTTCAGCCCGCGCGACCGCAAGTACGCCAACGGCGCGCGGCCGAACCGGGCGGCGACGTCGGGCTACTGGAAGGCCACCGGCACGGACAAGCCTATCCTGGCCTCGGCCACCGGGTGCGGCCGGGAGAAGGTCGGCGTCAAGAAGGCGCTCGTCTTCTACCGCGGGAAGCCGCCCAGGGGCCTCAAGACCAACTGGATCATGCATGAGTACCGCCTCACCGGAGCCTCTGCTGGCTCCACCACCACCAGCCGGCCGCCGCCGGTGACCGGCGGGAGCAGGGCCCCGGCCTCTCTCAGGGTACGTACTTACACGTGTCCATCGCACGGTCTATCAGTATTTATTTATTAACTACTCTCGAGCTTAATTATGGTATTGTTGATAGTTGATGAAGTTAATTATTGTACGCCGTCTCATCGATCAGTTGGACGACTGGGTGCTGTGCCGCATCTACAAGAAGACCAGCAAGGCCGCGGCCGCGGTCGGAGATGAGCAGAGGAGCATGGAGTGCGAGGACTCCGTGGAGGACGCGGTCACCGCGTACCCGCCCTACGCCACGGCGGGCATGGCCGGCGCAGGTGCGCATGGCAGCAACTACGTTCAACTGCTCCATCATCACGACAGCCACGAGGACAACTTCCAGCTAGACGGCCTGCTCACAGAACACGACGTCGGCCTCTCGGCGGGCGCCGCCTCGCTGGGCCACCTTGCCGCGGCGGCGAGGGCCACCAAACAGTTCCTCGCCCCGTCGTCCTCAACCCCGTTCAACTGGCTCGAGGCGTCAACCGG

>191_*NAM-1*

GCTTTTTATTATACTGTGCACAAGTATTTTTATATTCTTCCAGTAAGTACAGCGCATGTATGTGATCCTGTCGTCGTGCTTGTTCATGCGCTCGGGCGGGATCATCATCCATCAGAGAAGGCGACCTTCGGGGAGCACGAGTGGTACTTCTTCAGCCCGCGCGACCGCAAGTACGCCAACGGCGCGCGGCCGAACCGGGCGGCGACGTCGGGCTACTGGAAGGCCACCGGCACGGACAAGCCTATCCTGGCCTCGGCCACCGGGTGCGGCCGGGAGAAGGTCGGCGTCAAGAAGGCGCTCGTCTTCTACCGCGGGAAGCCGCCCAGGGGCCTCAAGACCAACTGGATCATGCATGAGTACCGCCTCACCGGAGCCTCTGCTGGCTCCACCACCACCAGCCGGCCGCCGCCGGTGACCGGCGGGAGCAGGGCCCCGGCCTCTCTCAGGGTACGTACTTACACGTGTCCATCGCACGGTCTATCAGTATTTATTTATTAACTACTCTCGAGCTTAATTATGGTATTGTTGATAGTTGATGAAGTTAATTATTGTACGCCGTCTCATCGATCAGTTGGACGACTGGGTGCTGTGCCGCATCTACAAGAAGACCAGCAAGGCCGCGGCCGCGGTCGGAGATGAGCAGAGGAGCATGGAGTGCGAGGACTCCGTGGAGGACGCGGTCACCGCGTACCCGCCCTACGCCACGGCGGGCATGGCCGGCGCAGGTGCGCATGGCAGCAACTACGTTCAACTGCTCCATCATCACGACAGCCACGAGGACAACTTCCAGCTAGACGGCCTGCTCACAGAACACGACGTCGGCCTCTCGGCGGGCGCCGCCTCGCTGGGCCACCTTGCCGCGGCGGCGAGGGCCACCAAACAGTTCCTCGCCCCGTCGTCCTCAACCCCGTTCAACTGGCTCGAGGCGTCAACCGG

>192_*NAM-1*

GCTTTTTATTATACTGTGCACAAGTATTTTTATATTCTTCCAGTAAGTACAGCGCATGTATGTGATCCTGTCGTCGTGCTTGTTCATGCGCTCGGGCGGGATCATCATCCATCAGAGAAGGCGACCTTCGGGGAGCACGAGTGGTACTTCTTCAGCCCGCGCGACCGCAAGTACCCCAACGGCGCGCGGCCGAACCGGGCGGCGACGTCGGGCTACTGGAAGGCCACCGGCACGGACAAGCCTATCCTGGCCTCGGCCACCGGGTGCGGCCGGGAGAAGGTCGGCGTCAAGAAGGCGCTCGTCTTCTACCGCGGGAAGCCGCCCAGGGGCCTCAAGACCAACTGGATCATGCATGAGTACCGCCTCACCGGAGCCTCTGCTGGCTCCACCACCACCAGCCGGCCGCCGCCGGTGACCGGCGGGAGCAGGGCCCCGGCCTCTCTCAGGGTACGTACTTACACGTGTCCATCGCACGGTCTATCAGTATTTATTTATTAACTACTCTCGAGCTTAATTATGGTATTGTTGATAGTTGATGAAGTTAATTATTGTACGCCGTCTCATCGATCAGTTGGACGACTGGGTGCTGTGCCGCATCTACAAGAAGACCAGCAAGGCCGCGGCCGCGGTCGGAGATGAGCAGAGGAGCATGGAGTGCGAGGACTCCGTGGAGGACGCGGTCACCGCGTACCCGCCCTACGCCACGGCGGGCATGGCCGGCGCAGGTGCGCATGGCAGCAACTACGTTCAACTGCTCCATCATCACGACAGCCACGAGGACAACTTCCAGCTAGACGGCCTGCTCACAGAACACGACGTCGGCCTCTCGGCGGGCGCCGCCTCGCTGGGCCACCTTGCCGCGGCGGCGAGGGCCACCAAACAGTTCCTCGCCCCGTCGTCCTCAACCCCGTTCAACTGGCTCGAGGCGTCAACCGG

>193_*NAM-1*

GCTTTTTATTATACTGTGCACAAGTATTTTTATATTCTTCCAGTAAGTACAGCGCATGTATGTGATCCTGTCGTCGTGCTTGTTCATGCGCTCGGGCGGGATCATCATCCATCAGAGAAGGCGACCTTCGGGGAGCACGAGTGGTACTTCTTCAGCCCGCGCGACCGCAAGTACGCCAACGGCGCGCGGCCGAACCGGGCGGCGACGTCGGGCTACTGGAAGGCCACCGGCACGGACAAGCCTATCCTGGCCTCGGCCACCGGGTGCGGCCGGGAGAAGGTCGGCGTCAAGAAGGCGCTCGTCTTCTACCGCGGGAAGCCGCCCAGGGGCCTCAAGACCAACTGGATCATGCATGAGTACCGCCTCACCGGAGCCTCTGCTGGCTCCACCACCACCAGCCGGCCGCCGCCGGTGACCGGCGGGAGCAGGGCCCCGGCCTCTCTCAGGGTACGTACTTACACGTGTCCATCGCACGGTCTATCAGTATTTATTTATTAACTACTCTCGAGCTTAATTATGGTATTGTTGATAGTTGATGAAGTTAATTATTGTACGCCGTCTCATCGATCAGTTGGACGACTGGGTGCTGTGCCGCATCTACAAGAAGACCAGCAAGGCCGCGGCCGCGGTCGGAGATGAGCAGAGGAGCATGGAGTGCGAGGACTCCGTGGAGGACGCGGTCACCGCGTACCCGCCCTACGCCACGGCGGGCATGGCCGGCGCAGGTGCGCATGGCAGCAACTACGTTCAACTGCTCCATCATCACGACAGCCACGAGGACAACTTCCAGCTAGACGGCCTGCTCACAGAACACGACGTCGGCCTCTCGGCGGGCGCCGCCTCGCTGGGCCACCTTGCCGCGGCGGCGAGGGCCACCAAACAGTTCCTCGCCCCGTCGTCCTCAACCCCGTTCAACTGGCTCGAGGCGTCAACCGG

>194_*NAM-1*

GCTTTTTATTATACTGTGCACAAGTATTTTTATATTCTTCCAGTAAGTACAGCGCATGTATGTGATCCTGTCGTCGTGCTTGTTCATGCGCTCGGGCGGGATCATCATCCATCAGAGAAGGCGACCTTCGGGGAGCACGAGTGGTACTTCTTCAGCCCGCGCGACCGCAAGTACGCCAACGGCGCGCGGCCGAACCGGGCGGCGACGTCGGGCTACTGGAAGGCCACCGGCACGGACAAGCCTATCCTGGCCTCGGCCACCGGGTGCGGCCGGGAGAAGGTCGGCGTCAAGAAGGCGCTCGTCTTCTACCGCGGGAAGCCGCCCAGGGGCCTCAAGACCAACTGGATCATGCATGAGTACCGCCTCACCGGAGCCTCTGCTGGCTCCACCACCACCAGCCGGCCGCCGCCGGTGACCGGCGGGAGCAGGGCCCCGGCCTCTCTCAGGGTACGTACTTACACGTGTCCATCGCACGGTCTATCAGTATTTATTTATTAACTACTCTCGAGCTTAATTATGGTATTGTTGATAGTTGATGAAGTTAATTATTGTACGCCGTCTCATCGATCAGTTGGACGACTGGGTGCTGTGCCGCATCTACAAGAAGACCAGCAAGGCCGCGGCCGCGGTCGGAGATGAGCAGAGGAGCATGGAGTGCGAGGACTCCGTGGAGGACGCGGTCACCGCGTACCCGCCCTACGCCACGGCGGGCATGGCCGGCGCAGGTGCGCATGGCAGCAACTACGTTCAACTGCTCCATCATCACGACAGCCACGAGGACAACTTCCAGCTAGACGGCCTGCTCACAGAACACGACGTCGGCCTCTCGGCGGGCGCCGCCTCGCTGGGCCACCTTGCCGCGGCGGCGAGGGCCACCAAACAGTTCCTCGCCCCGTCGTCCTCAACCCCGTTCAACTGGCTCGAGGCGTCAACCGG

>195_*NAM-1*

GCTTTTTATTATACTGTGCACAAGTATTTTTATATTCTTCCAGTAAGTACAGCGCATGTATGTGATCCTGTCGTCGTGCTTGTTCATGCGCTCGGGCGGGATCATCATCCATCAGAGAAGGCGACCTTCGGGGAGCACGAGTGGTACTTCTTCAGCCCGCGCGACCGCAAGTACCCCAACGGCGCGCGGCCGAACCGGGCGGCGACGTCGGGCTACTGGAAGGCCACCGGCACGGACAAGCCTATCCTGGCCTCGGCCACCGGGTGCGGCCGGGAGAAGGTCGGCGTCAAGAAGGCGCTCGTCTTCTACCGCGGGAAGCCGCCCAGGGGCCTCAAGACCAACTGGATCATGCATGAGTACCGCCTCACCGGAGCCTCTGCTGGCTCCACCACCACCAGCCGGCCGCCGCCGGTGACCGGCGGGAGCAGGGCCCCGGCCTCTCTCAGGGTACGTACTTACACGTGTCCATCGCACGGTCTATCAGTATTTATTTATTAACTACTCTCGAGCTTAATTATGGTATTGTTGATAGTTGATGAAGTTAATTATTGTACGCCGTCTCATCGATCAGTTGGACGACTGGGTGCTGTGCCGCATCTACAAGAAGACCAGCAAGGCCGCGGCCGCGGTCGGAGATGAGCAGAGGAGCATGGAGTGCGAGGACTCCGTGGAGGACGCGGTCACCGCGTACCCGCCCTACGCCACGGCGGGCATGGCCGGCGCAGGTGCGCATGGCAGCAACTACGTTCAACTGCTCCATCATCACGACAGCCACGAGGACAACTTCCAGCTAGACGGCCTGCTCACAGAACACGACGTCGGCCTCTCGGCGGGCGCCGCCTCGCTGGGCCACCTTGCCGCGGCGGCGAGGGCCACCAAACAGTTCCTCGCCCCGTCGTCCTCAACCCCGTTCAACTGGCTCGAGGCGTCAACCGG

>200_*NAM-1*

GCTTTTTATTATACTGTGCACAAGTATTTTTATATTCTTCCAGTAAGTACAGCGCATGTATGTGATCCTGTCGTCGTGCTTGTTCATGCGCTCGGGCGGGATCATCATCCATCAGAGAAGGCGACCTTCGGGGAGCACGAGTGGTACTTCTTCAGCCCGCGCGACCGCAAGTACGCCAACGGCGCGCGGCCGAACCGGGCGGCGACGTCGGGCTACTGGAAGGCCACCGGCACGGACAAGCCTATCCTGGCCTCGGCCACCGGGTGCGGCCGGGAGAAGGTCGGCGTCAAGAAGGCGCTCGTCTTCTACCGCGGGAAGCCGCCCAGGGGCCTCAAGACCAACTGGATCATGCATGAGTACCGCCTCACCGGAGCCTCTGCTGGCTCCACCACCACCAGCCGGCCGCCGCCGGTGACCGGCGGGAGCAGGGCCCCGGCCTCTCTCAGGGTACGTACTTACACGTGTCCATCGCACGGTCTATCAGTATTTATTTATTAACTACTCTCGAGCTTAATTATGGTATTGTTGATAGTTGATGAAGTTAATTATTGTACGCCGTCTCATCGATCAGTTGGACGACTGGGTGCTGTGCCGCATCTACAAGAAGACCAGCAAGGCCGCGGCCGCGGTCGGAGATGAGCAGAGGAGCATGGAGTGCGAGGACTCCGTGGAGGACGCGGTCACCGCGTACCCGCCCTACGCCACGGCGGGCATGGCCGGCGCAGGTGCGCATGGCAGCAACTACGTTCAACTGCTCCATCATCACGACAGCCACGAGGACAACTTCCAGCTAGACGGCCTGCTCACAGAACACGACGTCGGCCTCTCGGCGGGCGCCGCCTCGCTGGGCCACCTTGCCGCGGCGGCGAGGGCCACCAAACAGTTCCTCGCCCCGTCGTCCTCAACCCCGTTCAACTGGCTCGAGGCGTCAACCGG

>201_*NAM-1*

GCTTTTTATTATACTGTGCACAAGTATTTTTATATTCTTCCAGTAAGTACAGCGCATGTATGTGATCCTGTCGTCGTGCTTGTTCATGCGCTCGGGCGGGATCATCATCCATCAGAGAAGGCGACCTTCGGGGAGCACGAGTGGTACTTCTTCAGCCCGCGCGACCGCAAGTACGCCAACGGCGCGCGGCCGAACCGGGCGGCGACGTCGGGCTACTGGAAGGCCACCGGCACGGACAAGCCTATCCTGGCCTCGGCCACCGGGTGCGGCCGGGAGAAGGTCGGCGTCAAGAAGGCGCTCGTCTTCTACCGCGGGAAGCCGCCCAGGGGCCTCAAGACCAACTGGATCATGCATGAGTACCGCCTCACCGGAGCCTCTGCTGGCTCCACCACCACCAGCCGGCCGCCGCCGGTGACCGGCGGGAGCAGGGCCCCGGCCTCTCTCAGGGTACGTACTTACACGTGTCCATCGCACGGTCTATCAGTATTTATTTATTAACTACTCTCGAGCTTAATTATGGTATTGTTGATAGTTGATGAAGTTAATTATTGTACGCCGTCTCATCGATCAGTTGGACGACTGGGTGCTGTGCCGCATCTACAAGAAGACCAGCAAGGCCGCGGCCGCGGTCGGAGATGAGCAGAGGAGCATGGAGTGCGAGGACTCCGTGGAGGACGCGGTCACCGCGTACCCGCCCTACGCCACGGCGGGCATGGCCGGCGCAGGTGCGCATGGCAGCAACTACGTTCAACTGCTCCATCATCACGACAGCCACGAGGACAACTTCCAGCTAGACGGCCTGCTCACAGAACACGACGTCGGCCTCTCGGCGGGCGCCGCCTCGCTGGGCCACCTTGCCGCGGCGGCGAGGGCCACCAAACAGTTCCTCGCCCCGTCGTCCTCAACCCCGTTCAACTGGCTCGAGGCGTCAACCGG

>202_*NAM-1*

GCTTTTTATTATACTGTGCACAAGTATTTTTATATTCTTCCAGTAAGTACAGCGCATGTATGTGATCCTGTCGTCGTGCTTGTTCATGCGCTCGGGCGGGATCATCATCCATCAGAGAAGGCGACCTTCGGGGAGCACGAGTGGTACTTCTTCAGCCCGCGCGACCGCAAGTACCCCAACGGCGCGCGGCCGAACCGGGCGGCGACGTCGGGCTACTGGAAGGCCACCGGCACGGACAAGCCTATCCTGGCCTCGGCCACCGGGTGCGGCCGGGAGAAGGTCGGCGTCAAGAAGGCGCTCGTCTTCTACCGCGGGAAGCCGCCCAGGGGCCTCAAGACCAACTGGATCATGCATGAGTACCGCCTCACCGGAGCCTCTGCTGGCTCCACCACCACCAGCCGGCCGCCGCCGGTGACCGGCGGGAGCAGGGCCCCGGCCTCTCTCAGGGTACGTACTTACACGTGTCCATCGCACGGTCTATCAGTATTTATTTATTAACTACTCTCGAGCTTAATTATGGTATTGTTGATAGTTGATGAAGTTAATTATTGTACGCCGTCTCATCGATCAGTTGGACGACTGGGTGCTGTGCCGCATCTACAAGAAGACCAGCAAGGCCGCGGCCGCGGTCGGAGATGAGCAGAGGAGCATGGAGTGCGAGGACTCCGTGGAGGACGCGGTCACCGCGTACCCGCCCTACGCCACGGCGGGCATGGCCGGCGCAGGTGCGCATGGCAGCAACTACGTTCAACTGCTCCATCATCACGACAGCCACGAGGACAACTTCCAGCTAGACGGCCTGCTCACAGAACACGACGTCGGCCTCTCGGCGGGCGCCGCCTCGCTGGGCCACCTTGCCGCGGCGGCGAGGGCCACCAAACAGTTCCTCGCCCCGTCGTCCTCAACCCCGTTCAACTGGCTCGAGGCGTCAACCGG

>206_*NAM-1*

GCTTTTTATTATACTGTGCACAAGTATTTTTATATTCTTCCAGTAAGTACAGCGCATGTATGTGATCCTGTCGTCGTGCTTGTTCATGCGCTCGGGCGGGATCATCATCCATCAGAGAAGGCGACCTTCGGGGAGCACGAGTGGTACTTCTTCAGCCCGCGCGACCGCAAGTACGCCAACGGCGCGCGGCCGAACCGGGCGGCGACGTCGGGCTACTGGAAGGCCACCGGCACGGACAAGCCTATCCTGGCCTCGGCCACCGGGTGCGGCCGGGAGAAGGTCGGCGTCAAGAAGGCGCTCGTCTTCTACCGCGGGAAGCCGCCCAGGGGCCTCAAGACCAACTGGATCATGCATGAGTACCGCCTCACCGGAGCCTCTGCTGGCTCCACCACCACCAGCCGGCCGCCGCCGGTGACCGGCGGGAGCAGGGCCCCGGCCTCTCTCAGGGTACGTACTTACACGTGTCCATCGCACGGTCTATCAGTATTTATTTATTAACTACTCTCGAGCTTAATTATGGTATTGTTGATAGTTGATGAAGTTAATTATTGTACGCCGTCTCATCGATCAGTTGGACGACTGGGTGCTGTGCCGCATCTACAAGAAGACCAGCAAGGCCGCGGCCGCGGTCGGAGATGAGCAGAGGAGCATGGAGTGCGAGGACTCCGTGGAGGACGCGGTCACCGCGTACCCGCCCTACGCCACGGCGGGCATGGCCGGCGCAGGTGCGCATGGCAGCAACTACGTTCAACTGCTCCATCATCACGACAGCCACGAGGACAACTTCCAGCTAGACGGCCTGCTCACAGAACACGACGTCGGCCTCTCGGCGGGCGCCGCCTCGCTGGGCCACCTTGCCGCGGCGGCGAGGGCCACCAAACAGTTCCTCGCCCCGTCGTCCTCAACCCCGTTCAACTGGCTCGAGGCGTCAACCGG

>208_*NAM-1*

GCTTTTTATTATACTGTGCACAAGTATTTTTATATTCTTCCAGTAAGTACAGCGCATGTATGTGATCCTGTCGTCGTGCTTGTTCATGCGCTCGGGCGGGATCATCATCCATCAGAGAAGGCGACCTTCGGGGAGCACGAGTGGTACTTCTTCAGCCCGCGCGACCGCAAGTACCCCAACGGCGCGCGGCCGAACCGGGCGGCGACGTCGGGCTACTGGAAGGCCACCGGCACGGACAAGCCTATCCTGGCCTCGGCCACCGGGTGCGGCCGGGAGAAGGTCGGCGTCAAGAAGGCGCTCGTCTTCTACCGCGGGAAGCCGCCCAGGGGCCTCAAGACCAACTGGATCATGCATGAGTACCGCCTCACCGGAGCCTCTGCTGGCTCCACCACCACCAGCCGGCCGCCGCCGGTGACCGGCGGGAGCAGGGCCCCGGCCTCTCTCAGGGTACGTACTTACACGTGTCCATCGCACGGTCTATCAGTATTTATTTATTAACTACTCTCGAGCTTAATTATGGTATTGTTGATAGTTGATGAAGTTAATTATTGTACGCCGTCTCATCGATCAGTTGGACGACTGGGTGCTGTGCCGCATCTACAAGAAGACCAGCAAGGCCGCGGCCGCGGTCGGAGATGAGCAGAGGAGCATGGAGTGCGAGGACTCCGTGGAGGACGCGGTCACCGCGTACCCGCCCTACGCCACGGCGGGCATGGCCGGCGCAGGTGCGCATGGCAGCAACTACGTTCAACTGCTCCATCATCACGACAGCCACGAGGACAACTTCCAGCTAGACGGCCTGCTCACAGAACACGACGTCGGCCTCTCGGCGGGCGCCGCCTCGCTGGGCCACCTTGCCGCGGCGGCGAGGGCCACCAAACAGTTCCTCGCCCCGTCGTCCTCAACCCCGTTCAACTGGCTCGAGGCGTCAACCGG

>209_*NAM-1*

GCTTTTTATTATACTGTGCACAAGTATTTTTATATTCTTCCAGTAAGTACAGCGCATGTATGTGATCCTGTCGTCGTGCTTGTTCATGCGCTCGGGCGGGATCATCATCCATCAGAGAAGGCGACCTTCGGGGAGCACGAGTGGTACTTCTTCAGCCCGCGCGACCGCAAGTACCCCAACGGCGCGCGGCCGAACCGGGCGGCGACGTCGGGCTACTGGAAGGCCACCGGCACGGACAAGCCTATCCTGGCCTCGGCCACCGGGTGCGGCCGGGAGAAGGTCGGCGTCAAGAAGGCGCTCGTCTTCTACCGCGGGAAGCCGCCCAGGGGCCTCAAGACCAACTGGATCATGCATGAGTACCGCCTCACCGGAGCCTCTGCTGGCTCCACCACCACCAGCCGGCCGCCGCCGGTGACCGGCGGGAGCAGGGCCCCGGCCTCTCTCAGGGTACGTACTTACACGTGTCCATCGCACGGTCTATCAGTATTTATTTATTAACTACTCTCGAGCTTAATTATGGTATTGTTGATAGTTGATGAAGTTAATTATTGTACGCCGTCTCATCGATCAGTTGGACGACTGGGTGCTGTGCCGCATCTACAAGAAGACCAGCAAGGCCGCGGCCGCGGTCGGAGATGAGCAGAGGAGCATGGAGTGCGAGGACTCCGTGGAGGACGCGGTCACCGCGTACCCGCCCTACGCCACGGCGGGCATGGCCGGCGCAGGTGCGCATGGCAGCAACTACGTTCAACTGCTCCATCATCACGACAGCCACGAGGACAACTTCCAGCTAGACGGCCTGCTCACAGAACACGACGTCGGCCTCTCGGCGGGCGCCGCCTCGCTGGGCCACCTTGCCGCGGCGGCGAGGGCCACCAAACAGTTCCTCGCCCCGTCGTCCTCAACCCCGTTCAACTGGCTCGAGGCGTCAACCGG

>213_*NAM-1*

GCTTTTTATTATACTGTGCACAAGTATTTTTATATTCTTCCAGTAAGTACAGCGCATGTATGTGATCCTGTCGTCGTGCTTGTTCATGCGCTCGGGCGGGATCATCATCCATCAGAGAAGGCGACCTTCGGGGAGCACGAGTGGTACTTCTTCAGCCCGCGCGACCGCAAGTACGCCAACGGCGCGCGGCCGAACCGGGCGGCGACGTCGGGCTACTGGAAGGCCACCGGCACGGACAAGCCTATCCTGGCCTCGGCCACCGGGTGCGGCCGGGAGAAGGTCGGCGTCAAGAAGGCGCTCGTCTTCTACCGCGGGAAGCCGCCCAGGGGCCTCAAGACCAACTGGATCATGCATGAGTACCGCCTCACCGGAGCCTCTGCTGGCTCCACCACCACCAGCCGGCCGCCGCCGGTGACCGGCGGGAGCAGGGCCCCGGCCTCTCTCAGGGTACGTACTTACACGTGTCCATCGCACGGTCTATCAGTATTTATTTATTAACTACTCTCGAGCTTAATTATGGTATTGTTGATAGTTGATGAAGTTAATTATTGTACGCCGTCTCATCGATCAGTTGGACGACTGGGTGCTGTGCCGCATCTACAAGAAGACCAGCAAGGCCGCGGCCGCGGTCGGAGATGAGCAGAGGAGCATGGAGTGCGAGGACTCCGTGGAGGACGCGGTCACCGCGTACCCGCCCTACGCCACGGCGGGCATGGCCGGCGCAGGTGCGCATGGCAGCAACTACGTTCAACTGCTCCATCATCACGACAGCCACGAGGACAACTTCCAGCTAGACGGCCTGCTCACAGAACACGACGTCGGCCTCTCGGCGGGCGCCGCCTCGCTGGGCCACCTTGCCGCGGCGGCGAGGGCCACCAAACAGTTCCTCGCCCCGTCGTCCTCAACCCCGTTCAACTGGCTCGAGGCGTCAACCGG

>214_*NAM-1*

GCTTTTTATTATACTGTGCACAAGTATTTTTATATTCTTCCAGTAAGTACAGCGCATGTATGTGATCCTGTCGTCGTGCTTGTTCATGCGCTCGGGCGGGATCATCATCCATCAGAGAAGGCGACCTTCGGGGAGCACGAGTGGTACTTCTTCAGCCCGCGCGACCGCAAGTACGCCAACGGCGCGCGGCCGAACCGGGCGGCGACGTCGGGCTACTGGAAGGCCACCGGCACGGACAAGCCTATCCTGGCCTCGGCCACCGGGTGCGGCCGGGAGAAGGTCGGCGTCAAGAAGGCGCTCGTCTTCTACCGCGGGAAGCCGCCCAGGGGCCTCAAGACCAACTGGATCATGCATGAGTACCGCCTCACCGGAGCCTCTGCTGGCTCCACCACCACCAGCCGGCCGCCGCCGGTGACCGGCGGGAGCAGGGCCCCGGCCTCTCTCAGGGTACGTACTTACACGTGTCCATCGCACGGTCTATCAGTATTTATTTATTAACTACTCTCGAGCTTAATTATGGTATTGTTGATAGTTGATGAAGTTAATTATTGTACGCCGTCTCATCGATCAGTTGGACGACTGGGTGCTGTGCCGCATCTACAAGAAGACCAGCAAGGCCGCGGCCGCGGTCGGAGATGAGCAGAGGAGCATGGAGTGCGAGGACTCCGTGGAGGACGCGGTCACCGCGTACCCGCCCTACGCCACGGCGGGCATGGCCGGCGCAGGTGCGCATGGCAGCAACTACGTTCAACTGCTCCATCATCACGACAGCCACGAGGACAACTTCCAGCTAGACGGCCTGCTCACAGAACACGACGTCGGCCTCTCGGCGGGCGCCGCCTCGCTGGGCCACCTTGCCGCGGCGGCGAGGGCCACCAAACAGTTCCTCGCCCCGTCGTCCTCAACCCCGTTCAACTGGCTCGAGGCGTCAACCGG

>215_*NAM-1*

GCTTTTTATTATACTGTGCACAAGTATTTTTATATTCTTCCAGTAAGTACAGCGCATGTATGTGATCCTGTCGTCGTGCTTGTTCATGCGCTCGGGCGGGATCGTCATCCATCAGAGAAGGCGACCTTCGGGGAGCACGAGTGGTACTTCTTCAGCCCGCGCGACCGCAAGTACCCCAACGGCGCGCGGCCGAACCGGGCGGCGACGTCGGGCTACTGGAAGGCCACCGGCACGGACAAGCCTATCCTGGCCTCGGCCACCGGGTGCGGCCGGGAGAAGGTCGGCGTCAAGAAGGCGCTCGTCTTCTACCGCGGGAAGCCGCCCAGGGGCCTCAAGACCAACTGGATCATGCATGAGTACCGCCTCACCGGAGCCTCTGCTGGCTCCACCACCACCAGCCGGCCGCCGCCGGTGACCGGCGGGAGCAGGGCCCCGGCCTCTCTCAGGGTACGTACTTACACGTGTCCATCGCACGGTCTATCAGTATTTATTTATTAACTACTCTCGAGCTTAATTATGGTATTGTTGATAGTTGATGAAGTTAATTATTGTACGCCGTCTCATCGATCAGTTGGACGACTGGGTGCTGTGCCGCATCTACAAGAAGACCAGCAAGGCCGCGGCCGCGGTCGGAGATGAGCAGAGGAGCATGGAGTGCGAGGACTCCGTGGAGGACGCGGTCACCGCGTACCCGCCCTACGCCACGGCGGGCATGGCCGGCGCAGGTGCGCATGGCAGCAACTACGTTCAACTGCTCCATCATCACGACAGCCACGAGGACAACTTCCAGCTAGACGGCCTGCTCACAGAACACGACGTCGGCCTCTCGGCGGGCGCCGCCTCGCTGGGCCACCTTGCCGCGGCGGCGAGGGCCACCAAACAGTTCCTCGCCCCGTCGTCCTCAACCCCGTTCAACTGGCTCGAGGCGTCAACCGG

>216_*NAM-1*

GCTTTTTATTATACTGTGCACAAGTATTTTTATATTCTTCCAGTAAGTACAGCGCATGTATGTGATCCTGTCGTCGTGCTTGTTCATGCGCTCGGGCGGGATCATCATCCATCAGAGAAGGCGACCTTCGGGGAGCACGAGTGGTACTTCTTCAGCCCGCGCGACCGCAAGTACCCCAACGGCGCGCGGCCGAACCGGGCGGCGACGTCGGGCTACTGGAAGGCCACCGGCACGGACAAGCCTATCCTGGCCTCGGCCACCGGGTGCGGCCGGGAGAAGGTCGGCGTCAAGAAGGCGCTCGTCTTCTACCGCGGGAAGCCGCCCAGGGGCCTCAAGACCAACTGGATCATGCATGAGTACCGCCTCACCGGAGCCTCTGCTGGCTCCACCACCACCAGCCGGCCGCCGCCGGTGACCGGCGGGAGCAGGGCCCCGGCCTCTCTCAGGGTACGTACTTACACGTGTCCATCGCACGGTCTATCAGTATTTATTTATTAACTACTCTCGAGCTTAATTATGGTATTGTTGATAGTTGATGAAGTTAATTATTGTACGCCGTCTCATCGATCAGTTGGACGACTGGGTGCTGTGCCGCATCTACAAGAAGACCAGCAAGGCCGCGGCCGCGGTCGGAGATGAGCAGAGGAGCATGGAGTGCGAGGACTCCGTGGAGGACGCGGTCACCGCGTACCCGCCCTACGCCACGGCGGGCATGGCCGGCGCAGGTGCGCATGGCAGCAACTACGTTCAACTGCTCCATCATCACGACAGCCACGAGGACAACTTCCAGCTAGACGGCCTGCTCACAGAACACGACGTCGGCCTCTCGGCGGGCGCCGCCTCGCTGGGCCACCTTGCCGCGGCGGCGAGGGCCACCAAACAGTTCCTCGCCCCGTCGTCCTCAACCCCGTTCAACTGGCTCGAGGCGTCAACCGG

>217_*NAM-1*

GCTTTTTATTATACTGTGCACAAGTATTTTTATATTCTTCCAGTAAGTACAGCGCATGTATGTGATCCTGTCGTCGTGCTTGTTCATGCGCTCGGGCGGGATCATCATCCATCAGAGAAGGCGACCTTCGGGGAGCACGAGTGGTACTTCTTCAGCCCGCGCGACCGCAAGTACCCCAACGGCGCGCGGCCGAACCGGGCGGCGACGTCGGGCTACTGGAAGGCCACCGGCACGGACAAGCCTATCCTGGCCTCGGCCACCGGGTGCGGCCGGGAGAAGGTCGGCGTCAAGAAGGCGCTCGTCTTCTACCGCGGGAAGCCGCCCAGGGGCCTCAAGACCAACTGGATCATGCATGAGTACCGCCTCACCGGAGCCTCTGCTGGCTCCACCACCACCAGCCGGCCGCCGCCGGTGACCGGCGGGAGCAGGGCCCCGGCCTCTCTCAGGGTACGTACTTACACGTGTCCATCGCACGGTCTATCAGTATTTATTTATTAACTACTCTCGAGCTTAATTATGGTATTGTTGATAGTTGATGAAGTTAATTATTGTACGCCGTCTCATCGATCAGTTGGACGACTGGGTGCTGTGCCGCATCTACAAGAAGACCAGCAAGGCCGCGGCCGCGGTCGGAGATGAGCAGAGGAGCATGGAGTGCGAGGACTCCGTGGAGGACGCGGTCACCGCGTACCCGCCCTACGCCACGGCGGGCATGGCCGGCGCAGGTGCGCATGGCAGCAACTACGTTCAACTGCTCCATCATCACGACAGCCACGAGGACAACTTCCAGCTAGACGGCCTGCTCACAGAACACGACGTCGGCCTCTCGGCGGGCGCCGCCTCGCTGGGCCACCTTGCCGCGGCGGCGAGGGCCACCAAACAGTTCCTCGCCCCGTCGTCCTCAACCCCGTTCAACTGGCTCGAGGCGTCAACCGG

>242_*NAM-1*

GCTTTTTATTATACTGTGCACAAGTATTTTTATATTCTTCCAGTAAGTACAGCGCATGTATGTGATCCTGTCGTCGTGCTTGTTCATGCGCTCGGGCGGGATCATCATCCATCAGAGAAGGCGACCTTCGGGGAGCACGAGTGGTACTTCTTCAGCCCGCGCGACCGCAAGTACGCCAACGGCGCGCGGCCGAACCGGGCGGCGACGTCGGGCTACTGGAAGGCCACCGGCACGGACAAGCCTATCCTGGCCTCGGCCACCGGGTGCGGCCGGGAGAAGGTCGGCGTCAAGAAGGCGCTCGTCTTCTACCGCGGGAAGCCGCCCAGGGGCCTCAAGACCAACTGGATCATGCATGAGTACCGCCTCACCGGAGCCTCTGCTGGCTCCACCACCACCAGCCGGCCGCCGCCGGTGACCGGCGGGAGCAGGGCCCCGGCCTCTCTCAGGGTACGTACTTACACGTGTCCATCGCACGGTCTATCAGTATTTATTTATTAACTACTCTCGAGCTTAATTATGGTATTGTTGATAGTTGATGAAGTTAATTATTGTACGCCGTCTCATCGATCAGTTGGACGACTGGGTGCTGTGCCGCATCTACAAGAAGACCAGCAAGGCCGCGGCCGCGGTCGGAGATGAGCAGAGGAGCATGGAGTGCGAGGACTCCGTGGAGGACGCGGTCACCGCGTACCCGCCCTACGCCACGGCGGGCATGGCCGGCGCAGGTGCGCATGGCAGCAACTACGTTCAACTGCTCCATCATCACGACAGCCACGAGGACAACTTCCAGCTAGACGGCCTGCTCACAGAACACGACGTCGGCCTCTCGGCGGGCGCCGCCTCGCTGGGCCACCTTGCCGCGGCGGCGAGGGCCACCAAACAGTTCCTCGCCCCGTCGTCCTCAACCCCGTTCAACTGGCTCGAGGCGTCAACCGG

>247_*NAM-1*

GCTTTTTATTATACTGTGCACAAGTATTTTTATATTCTTCCAGTAAGTACAGCGCATGTATGTGATCCTGTCGTCGTGCTTGTTCATGCGCTCGGGCGGGATCATCATCCATCAGAGAAGGCGACCTTCGGGGAGCACGAGTGGTACTTCTTCAGCCCGCGCGACCGCAAGTACGCCAACGGCGCGCGGCCGAACCGGGCGGCGACGTCGGGCTACTGGAAGGCCACCGGCACGGACAAGCCTATCCTGGCCTCGGCCACCGGGTGCGGCCGGGAGAAGGTCGGCGTCAAGAAGGCGCTCGTCTTCTACCGCGGGAAGCCGCCCAGGGGCCTCAAGACCAACTGGATCATGCATGAGTACCGCCTCACCGGAGCCTCTGCTGGCTCCACCACCACCAGCCGGCCGCCGCCGGTGACCGGCGGGAGCAGGGCCCCGGCCTCTCTCAGGGTACGTACTTACACGTGTCCATCGCACGGTCTATCAGTATTTATTTATTAACTACTCTCGAGCTTAATTATGGTATTGTTGATAGTTGATGAAGTTAATTATTGTACGCCGTCTCATCGATCAGTTGGACGACTGGGTGCTGTGCCGCATCTACAAGAAGACCAGCAAGGCCGCGGCCGCGGTCGGAGATGAGCAGAGGAGCATGGAGTGCGAGGACTCCGTGGAGGACGCGGTCACCGCGTACCCGCCCTACGCCACGGCGGGCATGGCCGGCGCAGGTGCGCATGGCAGCAACTACGTTCAACTGCTCCATCATCACGACAGCCACGAGGACAACTTCCAGCTAGACGGCCTGCTCACAGAACACGACGTCGGCCTCTCGGCGGGCGCCGCCTCGCTGGGCCACCTTGCCGCGGCGGCGAGGGCCACCAAACAGTTCCTCGCCCCGTCGTCCTCAACCCCGTTCAACTGGCTCGAGGCGTCAACCGG

>263_*NAM-1*

GCTTTTTATTATACTGTGCACAAGTATTTTTATATTCTTCCAGTAAGTACAGCGCATGTATGTGATCCTGTCGTCGTGCTTGTTCATGCGCTCGGGCGGGATCATCATCCATCAGAGAAGGCGACCTTCGGGGAGCACGAGTGGTACTTCTTCAGCCCGCGCGACCGCAAGTACCCCAACGGCGCGCGGCCGAACCGGGCGGCGACGTCGGGCTACTGGAAGGCCACCGGCACGGACAAGCCTATCCTGGCCTCGGCCACCGGGTGCGGCCGGGAGAAGGTCGGCGTCAAGAAGGCGCTCGTCTTCTACCGCGGGAAGCCGCCCAGGGGCCTCAAGACCAACTGGATCATGCATGAGTACCGCCTCACCGGAGCCTCTGCTGGCTCCACCACCACCAGCCGGCCGCCGCCGGTGACCGGCGGGAGCAGGGCCCCGGCCTCTCTCAGGGTACGTACTTACACGTGTCCATCGCACGGTCTATCAGTATTTATTTATTAACTACTCTCGAGCTTAATTATGGTATTGTTGATAGTTGATGAAGTTAATTATTGTACGCCGTCTCATCGATCAGTTGGACGACTGGGTGCTGTGCCGCATCTACAAGAAGACCAGCAAGGCCGCGGCCGCGGTCGGAGATGAGCAGAGGAGCATGGAGTGCGAGGACTCCGTGGAGGACGCGGTCACCGCGTACCCGCCCTACGCCACGGCGGGCATGGCCGGCGCAGGTGCGCATGGCAGCAACTACGTTCAACTGCTCCATCATCACGACAGCCACGAGGACAACTTCCAGCTAGACGGCCTGCTCACAGAACACGACGTCGGCCTCTCGGCGGGCGCCGCCTCGCTGGGCCACCTTGCCGCGGCGGCGAGGGCCACCAAACAGTTCCTCGCCCCGTCGTCCTCAACCCCGTTCAACTGGCTCGAGGCGTCAACCGG

>265_*NAM-1*

GCTTTTTATTATACTGTGCACAAGTATTTTTATATTCTTCCAGTAAGTACAGCGCATGTATGTGATCCTGTCGTCGTGCTTGTTCATGCGCTCGGGCGGGATCATCATCCATCAGAGAAGGCGACCTTCGGGGAGCACGAGTGGTACTTCTTCAGCCCGCGCGACCGCAAGTACCCCAACGGCGCGCGGCCGAACCGGGCGGCGACGTCGGGCTACTGGAAGGCCACCGGCACGGACAAGCCTATCCTGGCCTCGGCCACCGGGTGCGGCCGGGAGAAGGTCGGCGTCAAGAAGGCGCTCGTCTTCTACCGCGGGAAGCCGCCCAGGGGCCTCAAGACCAACTGGATCATGCATGAGTACCGCCTCACCGGAGCCTCTGCTGGCTCCACCACCACCAGCCGGCCGCCGCCGGTGACCGGCGGGAGCAGGGCCCCGGCCTCTCTCAGGGTACGTACTTACACGTGTCCATCGCACGGTCTATCAGTATTTATTTATTAACTACTCTCGAGCTTAATTATGGTATTGTTGATAGTTGATGAAGTTAATTATTGTACGCCGTCTCATCGATCAGTTGGACGACTGGGTGCTGTGCCGCATCTACAAGAAGACCAGCAAGGCCGCGGCCGCGGTCGGAGATGAGCAGAGGAGCATGGAGTGCGAGGACTCCGTGGAGGACGCGGTCACCGCGTACCCGCCCTACGCCACGGCGGGCATGGCCGGCGCAGGTGCGCATGGCAGCAACTACGTTCAACTGCTCCATCATCACGACAGCCACGAGGACAACTTCCAGCTAGACGGCCTGCTCACAGAACACGACGTCGGCCTCTCGGCGGGCGCCGCCTCGCTGGGCCACCTTGCCGCGGCGGCGAGGGCCACCAAACAGTTCCTCGCCCCGTCGTCCTCAACCCCGTTCAACTGGCTCGAGGCGTCAACCGG

>288_*NAM-1*

GCTTTTTATTATACTGTGCACAAGTATTTTTATATTCTTCCAGTAAGTACAGCGCATGTATGTGATCCTGTCGTCGTGCTTGTTCATGCGCTCGGGCGGGATCATCATCCATCAGAGAAGGCGACCTTCGGGGAGCACGAGTGGTACTTCTTCAGCCCGCGCGACCGCAAGTACGCCAACGGCGCGCGGCCGAACCGGGCGGCGACGTCGGGCTACTGGAAGGCCACCGGCACGGACAAGCCTATCCTGGCCTCGGCCACCGGGTGCGGCCGGGAGAAGGTCGGCGTCAAGAAGGCGCTCGTCTTCTACCGCGGGAAGCCGCCCAGGGGCCTCAAGACCAACTGGATCATGCATGAGTACCGCCTCACCGGAGCCTCTGCTGGCTCCACCACCACCAGCCGGCCGCCGCCGGTGACCGGCGGGAGCAGGGCCCCGGCCTCTCTCAGGGTACGTACTTACACGTGTCCATCGCACGGTCTATCAGTATTTATTTATTAACTACTCTCGAGCTTAATTATGGTATTGTTGATAGTTGATGAAGTTAATTATTGTACGCCGTCTCATCGATCAGTTGGACGACTGGGTGCTGTGCCGCATCTACAAGAAGACCAGCAAGGCCGCGGCCGCGGTCGGAGATGAGCAGAGGAGCATGGAGTGCGAGGACTCCGTGGAGGACGCGGTCACCGCGTACCCGCCCTACGCCACGGCGGGCATGGCCGGCGCAGGTGCGCATGGCAGCAACTACGTTCAACTGCTCCATCATCACGACAGCCACGAGGACAACTTCCAGCTAGACGGCCTGCTCACAGAACACGACGTCGGCCTCTCGGCGGGCGCCGCCTCGCTGGGCCACCTTGCCGCGGCGGCGAGGGCCACCAAACAGTTCCTCGCCCCGTCGTCCTCAACCCCGTTCAACTGGCTCGAGGCGTCAACCGG

>290_*NAM-1*

GCTTTTTATTATACTGTGCACAAGTATTTTTATATTCTTCCAGTAAGTACAGCGCATGTATGTGATCCTGTCGTCGTGCTTGTTCATGCGCTCGGGCGGGATCATCATCCATCAGAGAAGGCGACCTTCGGGGAGCACGAGTGGTACTTCTTCAGCCCGCGCGACCGCAAGTACCCCAACGGCGCGCGGCCGAACCGGGCGGCGACGTCGGGCTACTGGAAGGCCACCGGCACGGACAAGCCTATCCTGGCCTCGGCCACCGGGTGCGGCCGGGAGAAGGTCGGCGTCAAGAAGGCGCTCGTCTTCTACCGCGGGAAGCCGCCCAGGGGCCTCAAGACCAACTGGATCATGCATGAGTACCGCCTCACCGGAGCCTCTGCTGGCTCCACCACCACCAGCCGGCCGCCGCCGGTGACCGGCGGGAGCAGGGCCCCGGCCTCTCTCAGGGTACGTACTTACACGTGTCCATCGCACGGTCTATCAGTATTTATTTATTAACTACTCTCGAGCTTAATTATGGTATTGTTGATAGTTGATGAAGTTAATTATTGTACGCCGTCTCATCGATCAGTTGGACGACTGGGTGCTGTGCCGCATCTACAAGAAGACCAGCAAGGCCGCGGCCGCGGTCGGAGATGAGCAGAGGAGCATGGAGTGCGAGGACTCCGTGGAGGACGCGGTCACCGCGTACCCGCCCTACGCCACGGCGGGCATGGCCGGCGCAGGTGCGCATGGCAGCAACTACGTTCAACTGCTCCATCATCACGACAGCCACGAGGACAACTTCCAGCTAGACGGCCTGCTCACAGAACACGACGTCGGCCTCTCGGCGGGCGCCGCCTCGCTGGGCCACCTTGCCGCGGCGGCGAGGGCCACCAAACAGTTCCTCGCCCCGTCGTCCTCAACCCCGTTCAACTGGCTCGAGGCGTCAACCGG

>294_*NAM-1*

GCTTTTTATTATACTGTGCACAAGTATTTTTATATTCTTCCAGTAAGTACAGCGCATGTATGTGATCCTGTCGTCGTGCTTGTTCATGCGCTCGGGCGGGATCATCATCCATCAGAGAAGGCGACCTTCGGGGAGCACGAGTGGTACTTCTTCAGCCCGCGCGACCGCAAGTACCCCAACGGCGCGCGGCCGAACCGGGCGGCGACGTCGGGCTACTGGAAGGCCACCGGCACGGACAAGCCTATCCTGGCCTCGGCCACCGGGTGCGGCCGGGAGAAGGTCGGCGTCAAGAAGGCGCTCGTCTTCTACCGCGGGAAGCCGCCCAGGGGCCTCAAGACCAACTGGATCATGCATGAGTACCGCCTCACCGGAGCCTCTGCTGGCTCCACCACCACCAGCCGGCCGCCGCCGGTGACCGGCGGGAGCAGGGCCCCGGCCTCTCTCAGGGTACGTACTTACACGTGTCCATCGCACGGTCTATCAGTATTTATTTATTAACTACTCTCGAGCTTAATTATGGTATTGTTGATAGTTGATGAAGTTAATTATTGTACGCCGTCTCATCGATCAGTTGGACGACTGGGTGCTGTGCCGCATCTACAAGAAGACCAGCAAGGCCGCGGCCGCGGTCGGAGATGAGCAGAGGAGCATGGAGTGCGAGGACTCCGTGGAGGACGCGGTCACCGCGTACCCGCCCTACGCCACGGCGGGCATGGCCGGCGCAGGTGCGCATGGCAGCAACTACGTTCAACTGCTCCATCATCACGACAGCCACGAGGACAACTTCCAGCTAGACGGCCTGCTCACAGAACACGACGTCGGCCTCTCGGCGGGCGCCGCCTCGCTGGGCCACCTTGCCGCGGCGGCGAGGGCCACCAAACAGTTCCTCGCCCCGTCGTCCTCAACCCCGTTCAACTGGCTCGAGGCGTCAACCGG

>295_*NAM-1*

GCTTTTTATTATACTGTGCACAAGTATTTTTATATTCTTCCAGTAAGTACAGCGCATGTATGTGATCCTGTCGTCGTGCTTGTTCATGCGCTCGGGCGGGATCATCATCCATCAGAGAAGGCGACCTTCGGGGAGCACGAGTGGTACTTCTTCAGCCCGCGCGACCGCAAGTACCCCAACGGCGCGCGGCCGAACCGGGCGGCGACGTCGGGCTACTGGAAGGCCACCGGCACGGACAAGCCTATCCTGGCCTCGGCCACCGGGTGCGGCCGGGAGAAGGTCGGCGTCAAGAAGGCGCTCGTCTTCTACCGCGGGAAGCCGCCCAGGGGCCTCAAGACCAACTGGATCATGCATGAGTACCGCCTCACCGGAGCCTCTGCTGGCTCCACCACCACCAGCCGGCCGCCGCCGGTGACCGGCGGGAGCAGGGCCCCGGCCTCTCTCAGGGTACGTACTTACACGTGTCCATCGCACGGTCTATCAGTATTTATTTATTAACTACTCTCGAGCTTAATTATGGTATTGTTGATAGTTGATGAAGTTAATTATTGTACGCCGTCTCATCGATCAGTTGGACGACTGGGTGCTGTGCCGCATCTACAAGAAGACCAGCAAGGCCGCGGCCGCGGTCGGAGATGAGCAGAGGAGCATGGAGTGCGAGGACTCCGTGGAGGACGCGGTCACCGCGTACCCGCCCTACGCCACGGCGGGCATGGCCGGCGCAGGTGCGCATGGCAGCAACTACGTTCAACTGCTCCATCATCACGACAGCCACGAGGACAACTTCCAGCTAGACGGCCTGCTCACAGAACACGACGTCGGCCTCTCGGCGGGCGCCGCCTCGCTGGGCCACCTTGCCGCGGCGGCGAGGGCCACCAAACAGTTCCTCGCCCCGTCGTCCTCAACCCCGTTCAACTGGCTCGAGGCGTCAACCGG

>296_*NAM-1*

GCTTTTTATTATACTGTGCACAAGTATTTTTATATTCTTCCAGTAAGTACAGCGCATGTATGTGATCCTGTCGTCGTGCTTGTTCATGCGCTCGGGCGGGATCATCATCCATCAGAGAAGGCGACCTTCGGGGAGCACGAGTGGTACTTCTTCAGCCCGCGCGACCGCAAGTACGCCAACGGCGCGCGGCCGAACCGGGCGGCGACGTCGGGCTACTGGAAGGCCACCGGCACGGACAAGCCTATCCTGGCCTCGGCCACCGGGTGCGGCCGGGAGAAGGTCGGCGTCAAGAAGGCGCTCGTCTTCTACCGCGGGAAGCCGCCCAGGGGCCTCAAGACCAACTGGATCATGCATGAGTACCGCCTCACCGGAGCCTCTGCTGGCTCCACCACCACCAGCCGGCCGCCGCCGGTGACCGGCGGGAGCAGGGCCCCGGCCTCTCTCAGGGTACGTACTTACACGTGTCCATCGCACGGTCTATCAGTATTTATTTATTAACTACTCTCGAGCTTAATTATGGTATTGTTGATAGTTGATGAAGTTAATTATTGTACGCCGTCTCATCGATCAGTTGGACGACTGGGTGCTGTGCCGCATCTACAAGAAGACCAGCAAGGCCGCGGCCGCGGTCGGAGATGAGCAGAGGAGCATGGAGTGCGAGGACTCCGTGGAGGACGCGGTCACCGCGTACCCGCCCTACGCCACGGCGGGCATGGCCGGCGCAGGTGCGCATGGCAGCAACTACGTTCAACTGCTCCATCATCACGACAGCCACGAGGACAACTTCCAGCTAGACGGCCTGCTCACAGAACACGACGTCGGCCTCTCGGCGGGCGCCGCCTCGCTGGGCCACCTTGCCGCGGCGGCGAGGGCCACCAAACAGTTCCTCGCCCCGTCGTCCTCAACCCCGTTCAACTGGCTCGAGGCGTCAACCGG

>297_*NAM-1*

GCTTTTTATTATACTGTGCACAAGTATTTTTATATTCTTCCAGTAAGTACAGCGCATGTATGTGATCCTGTCGTCGTGCTTGTTCATGCGCTCGGGCGGGATCATCATCCATCAGAGAAGGCGACCTTCGGGGAGCACGAGTGGTACTTCTTCAGCCCGCGCGACCGCAAGTACGCCAACGGCGCGCGGCCGAACCGGGCGGCGACGTCGGGCTACTGGAAGGCCACCGGCACGGACAAGCCTATCCTGGCCTCGGCCACCGGGTGCGGCCGGGAGAAGGTCGGCGTCAAGAAGGCGCTCGTCTTCTACCGCGGGAAGCCGCCCAGGGGCCTCAAGACCAACTGGATCATGCATGAGTACCGCCTCACCGGAGCCTCTGCTGGCTCCACCACCACCAGCCGGCCGCCGCCGGTGACCGGCGGGAGCAGGGCCCCGGCCTCTCTCAGGGTACGTACTTACACGTGTCCATCGCACGGTCTATCAGTATTTATTTATTAACTACTCTCGAGCTTAATTATGGTATTGTTGATAGTTGATGAAGTTAATTATTGTACGCCGTCTCATCGATCAGTTGGACGACTGGGTGCTGTGCCGCATCTACAAGAAGACCAGCAAGGCCGCGGCCGCGGTCGGAGATGAGCAGAGGAGCATGGAGTGCGAGGACTCCGTGGAGGACGCGGTCACCGCGTACCCGCCCTACGCCACGGCGGGCATGGCCGGCGCAGGTGCGCATGGCAGCAACTACGTTCAACTGCTCCATCATCACGACAGCCACGAGGACAACTTCCAGCTAGACGGCCTGCTCACAGAACACGACGTCGGCCTCTCGGCGGGCGCCGCCTCGCTGGGCCACCTTGCCGCGGCGGCGAGGGCCACCAAACAGTTCCTCGCCCCGTCGTCCTCAACCCCGTTCAACTGGCTCGAGGCGTCAACCGG

>298_*NAM-1*

GCTTTTTATTATACTGTGCACAAGTATTTTTATATTCTTCCAGTAAGTACAGCGCATGTATGTGATCCTGTCGTCGTGCTTGTTCATGCGCTCGGGCGGGATCATCATCCATCAGAGAAGGCGACCTTCGGGGAGCACGAGTGGTACTTCTTCAGCCCGCGCGACCGCAAGTACCCCAACGGCGCGCGGCCGAACCGGGCGGCGACGTCGGGCTACTGGAAGGCCACCGGCACGGACAAGCCTATCCTGGCCTCGGCCACCGGGTGCGGCCGGGAGAAGGTCGGCGTCAAGAAGGCGCTCGTCTTCTACCGCGGGAAGCCGCCCAGGGGCCTCAAGACCAACTGGATCATGCATGAGTACCGCCTCACCGGAGCCTCTGCTGGCTCCACCACCACCAGCCGGCCGCCGCCGGTGACCGGCGGGAGCAGGGCCCCGGCCTCTCTCAGGGTACGTACTTACACGTGTCCATCGCACGGTCTATCAGTATTTATTTATTAACTACTCTCGAGCTTAATTATGGTATTGTTGATAGTTGATGAAGTTAATTATTGTACGCCGTCTCATCGATCAGTTGGACGACTGGGTGCTGTGCCGCATCTACAAGAAGACCAGCAAGGCCGCGGCCGCGGTCGGAGATGAGCAGAGGAGCATGGAGTGCGAGGACTCCGTGGAGGACGCGGTCACCGCGTACCCGCCCTACGCCACGGCGGGCATGGCCGGCGCAGGTGCGCATGGCAGCAACTACGTTCAACTGCTCCATCATCACGACAGCCACGAGGACAACTTCCAGCTAGACGGCCTGCTCACAGAACACGACGTCGGCCTCTCGGCGGGCGCCGCCTCGCTGGGCCACCTTGCCGCGGCGGCGAGGGCCACCAAACAGTTCCTCGCCCCGTCGTCCTCAACCCCGTTCAACTGGCTCGAGGCGTCAACCGG

>299_*NAM-1*

GCTTTTTATTATACTGTGCACAAGTATTTTTATATTCTTCCAGTAAGTACAGCGCATGTATGTGATCCTGTCGTCGTGCTTGTTCATGCGCTCGGGCGGGATCATCATCCATCAGAGAAGGCGACCTTCGGGGAGCACGAGTGGTACTTCTTCAGCCCGCGCGACCGCAAGTACGCCAACGGCGCGCGGCCGAACCGGGCGGCGACGTCGGGCTACTGGAAGGCCACCGGCACGGACAAGCCTATCCTGGCCTCGGCCACCGGGTGCGGCCGGGAGAAGGTCGGCGTCAAGAAGGCGCTCGTCTTCTACCGCGGGAAGCCGCCCAGGGGCCTCAAGACCAACTGGATCATGCATGAGTACCGCCTCACCGGAGCCTCTGCTGGCTCCACCACCACCAGCCGGCCGCCGCCGGTGACCGGCGGGAGCAGGGCCCCGGCCTCTCTCAGGGTACGTACTTACACGTGTCCATCGCACGGTCTATCAGTATTTATTTATTAACTACTCTCGAGCTTAATTATGGTATTGTTGATAGTTGATGAAGTTAATTATTGTACGCCGTCTCATCGATCAGTTGGACGACTGGGTGCTGTGCCGCATCTACAAGAAGACCAGCAAGGCCGCGGCCGCGGTCGGAGATGAGCAGAGGAGCATGGAGTGCGAGGACTCCGTGGAGGACGCGGTCACCGCGTACCCGCCCTACGCCACGGCGGGCATGGCCGGCGCAGGTGCGCATGGCAGCAACTACGTTCAACTGCTCCATCATCACGACAGCCACGAGGACAACTTCCAGCTAGACGGCCTGCTCACAGAACACGACGTCGGCCTCTCGGCGGGCGCCGCCTCGCTGGGCCACCTTGCCGCGGCGGCGAGGGCCACCAAACAGTTCCTCGCCCCGTCGTCCTCAACCCCGTTCAACTGGCTCGAGGCGTCAACCGG

>310_*NAM-1*

GCTTTTTATTATACTGTGCACAAGTATTTTTATATTCTTCCAGTAAGTACAGCGCATGTATGTGATCCTGTCGTCGTGCTTGTTCATGCGCTCGGGCGGGATCATCATCCATCAGAGAAGGCGACCTTCGGGGAGCACGAGTGGTACTTCTTCAGCCCGCGCGACCGCAAGTACCCCAACGGCGCGCGGCCGAACCGGGCGGCGACGTCGGGCTACTGGAAGGCCACCGGCACGGACAAGCCTATCCTGGCCTCGGCCACCGGGTGCGGCCGGGAGAAGGTCGGCGTCAAGAAGGCGCTCGTCTTCTACCGCGGGAAGCCGCCCAGGGGCCTCAAGACCAACTGGATCATGCATGAGTACCGCCTCACCGGAGCCTCTGCTGGCTCCACCACCACCAGCCGGCCGCCGCCGGTGACCGGCGGGAGCAGGGCCCCGGCCTCTCTCAGGGTACGTACTTACACGTGTCCATCGCACGGTCTATCAGTATTTATTTATTAACTACTCTCGAGCTTAATTATGGTATTGTTGATAGTTGATGAAGTTAATTATTGTACGCCGTCTCATCGATCAGTTGGACGACTGGGTGCTGTGCCGCATCTACAAGAAGACCAGCAAGGCCGCGGCCGCGGTCGGAGATGAGCAGAGGAGCATGGAGTGCGAGGACTCCGTGGAGGACGCGGTCACCGCGTACCCGCCCTACGCCACGGCGGGCATGGCCGGCGCAGGTGCGCATGGCAGCAACTACGTTCAACTGCTCCATCATCACGACAGCCACGAGGACAACTTCCAGCTAGACGGCCTGCTCACAGAACACGACGTCGGCCTCTCGGCGGGCGCCGCCTCGCTGGGCCACCTTGCCGCGGCGGCGAGGGCCACCAAACAGTTCCTCGCCCCGTCGTCCTCAACCCCGTTCAACTGGCTCGAGGCGTCAACCGG

>311_*NAM-1*

GCTTTTTATTATACTGTGCACAAGTATTTTTATATTCTTCCAGTAAGTACAGCGCATGTATGTGATCCTGTCGTCGTGCTTGTTCATGCGCTCGGGCGGGATCATCATCCATCAGAGAAGGCGACCTTCGGGGAGCACGAGTGGTACTTCTTCAGCCCGCGCGACCGCAAGTACGCCAACGGCGCGCGGCCGAACCGGGCGGCGACGTCGGGCTACTGGAAGGCCACCGGCACGGACAAGCCTATCCTGGCCTCGGCCACCGGGTGCGGCCGGGAGAAGGTCGGCGTCAAGAAGGCGCTCGTCTTCTACCGCGGGAAGCCGCCCAGGGGCCTCAAGACCAACTGGATCATGCATGAGTACCGCCTCACCGGAGCCTCTGCTGGCTCCACCACCACCAGCCGGCCGCCGCCGGTGACCGGCGGGAGCAGGGCCCCGGCCTCTCTCAGGGTACGTACTTACACGTGTCCATCGCACGGTCTATCAGTATTTATTTATTAACTACTCTCGAGCTTAATTATGGTATTGTTGATAGTTGATGAAGTTAATTATTGTACGCCGTCTCATCGATCAGTTGGACGACTGGGTGCTGTGCCGCATCTACAAGAAGACCAGCAAGGCCGCGGCCGCGGTCGGAGATGAGCAGAGGAGCATGGAGTGCGAGGACTCCGTGGAGGACGCGGTCACCGCGTACCCGCCCTACGCCACGGCGGGCATGGCCGGCGCAGGTGCGCATGGCAGCAACTACGTTCAACTGCTCCATCATCACGACAGCCACGAGGACAACTTCCAGCTAGACGGCCTGCTCACAGAACACGACGTCGGCCTCTCGGCGGGCGCCGCCTCGCTGGGCCACCTTGCCGCGGCGGCGAGGGCCACCAAACAGTTCCTCGCCCCGTCGTCCTCAACCCCGTTCAACTGGCTCGAGGCGTCAACCGG

>312_*NAM-1*

GCTTTTTATTATACTGTGCACAAGTATTTTTATATTCTTCCAGTAAGTACAGCGCATGTATGTGATCCTGTCGTCGTGCTTGTTCATGCGCTCGGGCGGGATCATCATCCATCAGAGAAGGCGACCTTCGGGGAGCACGAGTGGTACTTCTTCAGCCCGCGCGACCGCAAGTACGCCAACGGCGCGCGGCCGAACCGGGCGGCGACGTCGGGCTACTGGAAGGCCACCGGCACGGACAAGCCTATCCTGGCCTCGGCCACCGGGTGCGGCCGGGAGAAGGTCGGCGTCAAGAAGGCGCTCGTCTTCTACCGCGGGAAGCCGCCCAGGGGCCTCAAGACCAACTGGATCATGCATGAGTACCGCCTCACCGGAGCCTCTGCTGGCTCCACCACCACCAGCCGGCCGCCGCCGGTGACCGGCGGGAGCAGGGCCCCGGCCTCTCTCAGGGTACGTACTTACACGTGTCCATCGCACGGTCTATCAGTATTTATTTATTAACTACTCTCGAGCTTAATTATGGTATTGTTGATAGTTGATGAAGTTAATTATTGTACGCCGTCTCATCGATCAGTTGGACGACTGGGTGCTGTGCCGCATCTACAAGAAGACCAGCAAGGCCGCGGCCGCGGTCGGAGATGAGCAGAGGAGCATGGAGTGCGAGGACTCCGTGGAGGACGCGGTCACCGCGTACCCGCCCTACGCCACGGCGGGCATGGCCGGCGCAGGTGCGCATGGCAGCAACTACGTTCAACTGCTCCATCATCACGACAGCCACGAGGACAACTTCCAGCTAGACGGCCTGCTCACAGAACACGACGTCGGCCTCTCGGCGGGCGCCGCCTCGCTGGGCCACCTTGCCGCGGCGGCGAGGGCCACCAAACAGTTCCTCGCCCCGTCGTCCTCAACCCCGTTCAACTGGCTCGAGGCGTCAACCGG

>313_*NAM-1*

GCTTTTTATTATACTGTGCACAAGTATTTTTATATTCTTCCAGTAAGTACAGCGCATGTATGTGATCCTGTCGTCGTGCTTGTTCATGCGCTCGGGCGGGATCATCATCCATCAGAGAAGGCGACCTTCGGGGAGCACGAGTGGTACTTCTTCAGCCCGCGCGACCGCAAGTACGCCAACGGCGCGCGGCCGAACCGGGCGGCGACGTCGGGCTACTGGAAGGCCACCGGCACGGACAAGCCTATCCTGGCCTCGGCCACCGGGTGCGGCCGGGAGAAGGTCGGCGTCAAGAAGGCGCTCGTCTTCTACCGCGGGAAGCCGCCCAGGGGCCTCAAGACCAACTGGATCATGCATGAGTACCGCCTCACCGGAGCCTCTGCTGGCTCCACCACCACCAGCCGGCCGCCGCCGGTGACCGGCGGGAGCAGGGCCCCGGCCTCTCTCAGGGTACGTACTTACACGTGTCCATCGCACGGTCTATCAGTATTTATTTATTAACTACTCTCGAGCTTAATTATGGTATTGTTGATAGTTGATGAAGTTAATTATTGTACGCCGTCTCATCGATCAGTTGGACGACTGGGTGCTGTGCCGCATCTACAAGAAGACCAGCAAGGCCGCGGCCGCGGTCGGAGATGAGCAGAGGAGCATGGAGTGCGAGGACTCCGTGGAGGACGCGGTCACCGCGTACCCGCCCTACGCCACGGCGGGCATGGCCGGCGCAGGTGCGCATGGCAGCAACTACGTTCAACTGCTCCATCATCACGACAGCCACGAGGACAACTTCCAGCTAGACGGCCTGCTCACAGAACACGACGTCGGCCTCTCGGCGGGCGCCGCCTCGCTGGGCCACCTTGCCGCGGCGGCGAGGGCCACCAAACAGTTCCTCGCCCCGTCGTCCTCAACCCCGTTCAACTGGCTCGAGGCGTCAACCGG

>314_*NAM-1*

GCTTTTTATTATACTGTGCACAAGTATTTTTATATTCTTCCAGTAAGTACAGCGCATGTATGTGATCCTGTCGTCGTGCTTGTTCATGCGCTCGGGCGGGATCATCATCCATCAGAGAAGGCGACCTTCGGGGAGCACGAGTGGTACTTCTTCAGCCCGCGCGACCGCAAGTACGCCAACGGCGCGCGGCCGAACCGGGCGGCGACGTCGGGCTACTGGAAGGCCACCGGCACGGACAAGCCTATCCTGGCCTCGGCCACCGGGTGCGGCCGGGAGAAGGTCGGCGTCAAGAAGGCGCTCGTCTTCTACCGCGGGAAGCCGCCCAGGGGCCTCAAGACCAACTGGATCATGCATGAGTACCGCCTCACCGGAGCCTCTGCTGGCTCCACCACCACCAGCCGGCCGCCGCCGGTGACCGGCGGGAGCAGGGCCCCGGCCTCTCTCAGGGTACGTACTTACACGTGTCCATCGCACGGTCTATCAGTATTTATTTATTAACTACTCTCGAGCTTAATTATGGTATTGTTGATAGTTGATGAAGTTAATTATTGTACGCCGTCTCATCGATCAGTTGGACGACTGGGTGCTGTGCCGCATCTACAAGAAGACCAGCAAGGCCGCGGCCGCGGTCGGAGATGAGCAGAGGAGCATGGAGTGCGAGGACTCCGTGGAGGACGCGGTCACCGCGTACCCGCCCTACGCCACGGCGGGCATGGCCGGCGCAGGTGCGCATGGCAGCAACTACGTTCAACTGCTCCATCATCACGACAGCCACGAGGACAACTTCCAGCTAGACGGCCTGCTCACAGAACACGACGTCGGCCTCTCGGCGGGCGCCGCCTCGCTGGGCCACCTTGCCGCGGCGGCGAGGGCCACCAAACAGTTCCTCGCCCCGTCGTCCTCAACCCCGTTCAACTGGCTCGAGGCGTCAACCGG

>328_*NAM-1*

GCTTTTTATTATACTGTGCACAAGTATTTTTATATTCTTCCAGTAAGTACAGCGCATGTATGTGATCCTGTCGTCGTGCTTGTTCATGCGCTCGGGCGGGATCATCATCCATCAGAGAAGGCGACCTTCGGGGAGCACGAGTGGTACTTCTTCAGCCCGCGCGACCGCAAGTACCCCAACGGCGCGCGGCCGAACCGGGCGGCGACGTCGGGCTACTGGAAGGCCACCGGCACGGACAAGCCTATCCTGGCCTCGGCCACCGGGTGCGGCCGGGAGAAGGTCGGCGTCAAGAAGGCGCTCGTCTTCTACCGCGGGAAGCCGCCCAGGGGCCTCAAGACCAACTGGATCATGCATGAGTACCGCCTCACCGGAGCCTCTGCTGGCTCCACCACCACCAGCCGGCCGCCGCCGGTGACCGGCGGGAGCAGGGCCCCGGCCTCTCTCAGGGTACGTACTTACACGTGTCCATCGCACGGTCTATCAGTATTTATTTATTAACTACTCTCGAGCTTAATTATGGTATTGTTGATAGTTGATGAAGTTAATTATTGTACGCCGTCTCATCGATCAGTTGGACGACTGGGTGCTGTGCCGCATCTACAAGAAGACCAGCAAGGCCGCGGCCGCGGTCGGAGATGAGCAGAGGAGCATGGAGTGCGAGGACTCCGTGGAGGACGCGGTCACCGCGTACCCGCCCTACGCCACGGCGGGCATGGCCGGCGCAGGTGCGCATGGCAGCAACTACGTTCAACTGCTCCATCATCACGACAGCCACGAGGACAACTTCCAGCTAGACGGCCTGCTCACAGAACACGACGTCGGCCTCTCGGCGGGCGCCGCCTCGCTGGGCCACCTTGCCGCGGCGGCGAGGGCCACCAAACAGTTCCTCGCCCCGTCGTCCTCAACCCCGTTCAACTGGCTCGAGGCGTCAACCGG

>330_*NAM-1*

GCTTTTTATTATACTGTGCACAAGTATTTTTATATTCTTCCAGTAAGTACAGCGCATGTATGTGATCCTGTCGTCGTGCTTGTTCATGCGCTCGGGCGGGATCATCATCCATCAGAGAAGGCGACCTTCGGGGAGCACGAGTGGTACTTCTTCAGCCCGCGCGACCGCAAGTACGCCAACGGCGCGCGGCCGAACCGGGCGGCGACGTCGGGCTACTGGAAGGCCACCGGCACGGACAAGCCTATCCTGGCCTCGGCCACCGGGTGCGGCCGGGAGAAGGTCGGCGTCAAGAAGGCGCTCGTCTTCTACCGCGGGAAGCCGCCCAGGGGCCTCAAGACCAACTGGATCATGCATGAGTACCGCCTCACCGGAGCCTCTGCTGGCTCCACCACCACCAGCCGGCCGCCGCCGGTGACCGGCGGGAGCAGGGCCCCGGCCTCTCTCAGGGTACGTACTTACACGTGTCCATCGCACGGTCTATCAGTATTTATTTATTAACTACTCTCGAGCTTAATTATGGTATTGTTGATAGTTGATGAAGTTAATTATTGTACGCCGTCTCATCGATCAGTTGGACGACTGGGTGCTGTGCCGCATCTACAAGAAGACCAGCAAGGCCGCGGCCGCGGTCGGAGATGAGCAGAGGAGCATGGAGTGCGAGGACTCCGTGGAGGACGCGGTCACCGCGTACCCGCCCTACGCCACGGCGGGCATGGCCGGCGCAGGTGCGCATGGCAGCAACTACGTTCAACTGCTCCATCATCACGACAGCCACGAGGACAACTTCCAGCTAGACGGCCTGCTCACAGAACACGACGTCGGCCTCTCGGCGGGCGCCGCCTCGCTGGGCCACCTTGCCGCGGCGGCGAGGGCCACCAAACAGTTCCTCGCCCCGTCGTCCTCAACCCCGTTCAACTGGCTCGAGGCGTCAACCGG

>333_*NAM-1*

GCTTTTTATTATACTGTGCACAAGTATTTTTATATTCTTCCAGTAAGTACAGCGCATGTATGTGATCCTGTCGTCGTGCTTGTTCATGCGCTCGGGCGGGATCATCATCCATCAGAGAAGGCGACCTTCGGGGAGCACGAGTGGTACTTCTTCAGCCCGCGCGACCGCAAGTACGCCAACGGCGCGCGGCCGAACCGGGCGGCGACGTCGGGCTACTGGAAGGCCACCGGCACGGACAAGCCTATCCTGGCCTCGGCCACCGGGTGCGGCCGGGAGAAGGTCGGCGTCAAGAAGGCGCTCGTCTTCTACCGCGGGAAGCCGCCCAGGGGCCTCAAGACCAACTGGATCATGCATGAGTACCGCCTCACCGGAGCCTCTGCTGGCTCCACCACCACCAGCCGGCCGCCGCCGGTGACCGGCGGGAGCAGGGCCCCGGCCTCTCTCAGGGTACGTACTTACACGTGTCCATCGCACGGTCTATCAGTATTTATTTATTAACTACTCTCGAGCTTAATTATGGTATTGTTGATAGTTGATGAAGTTAATTATTGTACGCCGTCTCATCGATCAGTTGGACGACTGGGTGCTGTGCCGCATCTACAAGAAGACCAGCAAGGCCGCGGCCGCGGTCGGAGATGAGCAGAGGAGCATGGAGTGCGAGGACTCCGTGGAGGACGCGGTCACCGCGTACCCGCCCTACGCCACGGCGGGCATGGCCGGCGCAGGTGCGCATGGCAGCAACTACGTTCAACTGCTCCATCATCACGACAGCCACGAGGACAACTTCCAGCTAGACGGCCTGCTCACAGAACACGACGTCGGCCTCTCGGCGGGCGCCGCCTCGCTGGGCCACCTTGCCGCGGCGGCGAGGGCCACCAAACAGTTCCTCGCCCCGTCGTCCTCAACCCCGTTCAACTGGCTCGAGGCGTCAACCGG

>334_*NAM-1*

GCTTTTTATTATACTGTGCACAAGTATTTTTATATTCTTCCAGTAAGTACAGCGCATGTATGTGATCCTGTCGTCGTGCTTGTTCATGCGCTCGGGCGGGATCATCATCCATCAGAGAAGGCGACCTTCGGGGAGCACGAGTGGTACTTCTTCAGCCCGCGCGACCGCAAGTACGCCAACGGCGCGCGGCCGAACCGGGCGGCGACGTCGGGCTACTGGAAGGCCACCGGCACGGACAAGCCTATCCTGGCCTCGGCCACCGGGTGCGGCCGGGAGAAGGTCGGCGTCAAGAAGGCGCTCGTCTTCTACCGCGGGAAGCCGCCCAGGGGCCTCAAGACCAACTGGATCATGCATGAGTACCGCCTCACCGGAGCCTCTGCTGGCTCCACCACCACCAGCCGGCCGCCGCCGGTGACCGGCGGGAGCAGGGCCCCGGCCTCTCTCAGGGTACGTACTTACACGTGTCCATCGCACGGTCTATCAGTATTTATTTATTAACTACTCTCGAGCTTAATTATGGTATTGTTGATAGTTGATGAAGTTAATTATTGTACGCCGTCTCATCGATCAGTTGGACGACTGGGTGCTGTGCCGCATCTACAAGAAGACCAGCAAGGCCGCGGCCGCGGTCGGAGATGAGCAGAGGAGCATGGAGTGCGAGGACTCCGTGGAGGACGCGGTCACCGCGTACCCGCCCTACGCCACGGCGGGCATGGCCGGCGCAGGTGCGCATGGCAGCAACTACGTTCAACTGCTCCATCATCACGACAGCCACGAGGACAACTTCCAGCTAGACGGCCTGCTCACAGAACACGACGTCGGCCTCTCGGCGGGCGCCGCCTCGCTGGGCCACCTTGCCGCGGCGGCGAGGGCCACCAAACAGTTCCTCGCCCCGTCGTCCTCAACCCCGTTCAACTGGCTCGAGGCGTCAACCGG

>335_*NAM-1*

GCTTTTTATTATACTGTGCACAAGTATTTTTATATTCTTCCAGTAAGTACAGCGCATGTATGTGATCCTGTCGTCGTGCTTGTTCATGCGCTCGGGCGGGATCATCATCCATCAGAGAAGGCGACCTTCGGGGAGCACGAGTGGTACTTCTTCAGCCCGCGCGACCGCAAGTACGCCAACGGCGCGCGGCCGAACCGGGCGGCGACGTCGGGCTACTGGAAGGCCACCGGCACGGACAAGCCTATCCTGGCCTCGGCCACCGGGTGCGGCCGGGAGAAGGTCGGCGTCAAGAAGGCGCTCGTCTTCTACCGCGGGAAGCCGCCCAGGGGCCTCAAGACCAACTGGATCATGCATGAGTACCGCCTCACCGGAGCCTCTGCTGGCTCCACCACCACCAGCCGGCCGCCGCCGGTGACCGGCGGGAGCAGGGCCCCGGCCTCTCTCAGGGTACGTACTTACACGTGTCCATCGCACGGTCTATCAGTATTTATTTATTAACTACTCTCGAGCTTAATTATGGTATTGTTGATAGTTGATGAAGTTAATTATTGTACGCCGTCTCATCGATCAGTTGGACGACTGGGTGCTGTGCCGCATCTACAAGAAGACCAGCAAGGCCGCGGCCGCGGTCGGAGATGAGCAGAGGAGCATGGAGTGCGAGGACTCCGTGGAGGACGCGGTCACCGCGTACCCGCCCTACGCCACGGCGGGCATGGCCGGCGCAGGTGCGCATGGCAGCAACTACGTTCAACTGCTCCATCATCACGACAGCCACGAGGACAACTTCCAGCTAGACGGCCTGCTCACAGAACACGACGTCGGCCTCTCGGCGGGCGCCGCCTCGCTGGGCCACCTTGCCGCGGCGGCGAGGGCCACCAAACAGTTCCTCGCCCCGTCGTCCTCAACCCCGTTCAACTGGCTCGAGGCGTCAACCGG

>336_*NAM-1*

GCTTTTTATTATACTGTGCACAAGTATTTTTATATTCTTCCAGTAAGTACAGCGCATGTATGTGATCCTGTCGTCGTGCTTGTTCATGCGCTCGGGCGGGATCATCATCCATCAGAGAAGGCGACCTTCGGGGAGCACGAGTGGTACTTCTTCAGCCCGCGCGACCGCAAGTACGCCAACGGCGCGCGGCCGAACCGGGCGGCGACGTCGGGCTACTGGAAGGCCACCGGCACGGACAAGCCTATCCTGGCCTCGGCCACCGGGTGCGGCCGGGAGAAGGTCGGCGTCAAGAAGGCGCTCGTCTTCTACCGCGGGAAGCCGCCCAGGGGCCTCAAGACCAACTGGATCATGCATGAGTACCGCCTCACCGGAGCCTCTGCTGGCTCCACCACCACCAGCCGGCCGCCGCCGGTGACCGGCGGGAGCAGGGCCCCGGCCTCTCTCAGGGTACGTACTTACACGTGTCCATCGCACGGTCTATCAGTATTTATTTATTAACTACTCTCGAGCTTAATTATGGTATTGTTGATAGTTGATGAAGTTAATTATTGTACGCCGTCTCATCGATCAGTTGGACGACTGGGTGCTGTGCCGCATCTACAAGAAGACCAGCAAGGCCGCGGCCGCGGTCGGAGATGAGCAGAGGAGCATGGAGTGCGAGGACTCCGTGGAGGACGCGGTCACCGCGTACCCGCCCTACGCCACGGCGGGCATGGCCGGCGCAGGTGCGCATGGCAGCAACTACGTTCAACTGCTCCATCATCACGACAGCCACGAGGACAACTTCCAGCTAGACGGCCTGCTCACAGAACACGACGTCGGCCTCTCGGCGGGCGCCGCCTCGCTGGGCCACCTTGCCGCGGCGGCGAGGGCCACCAAACAGTTCCTCGCCCCGTCGTCCTCAACCCCGTTCAACTGGCTCGAGGCGTCAACCGG

>378_*NAM-1*

GCTTTTTATTATACTGTGCACAAGTATTTTTATATTCTTCCAGTAAGTACAGCGCATGTATGTGATCCTGTCGTCGTGCTTGTTCATGCGCTCGGGCGGGATCATCATCCATCAGAGAAGGCGACCTTCGGGGAGCACGAGTGGTACTTCTTCAGCCCGCGCGACCGCAAGTACGCCAACGGCGCGCGGCCGAACCGGGCGGCGACGTCGGGCTACTGGAAGGCCACCGGCACGGACAAGCCTATCCTGGCCTCGGCCACCGGGTGCGGCCGGGAGAAGGTCGGCGTCAAGAAGGCGCTCGTCTTCTACCGCGGGAAGCCGCCCAGGGGCCTCAAGACCAACTGGATCATGCATGAGTACCGCCTCACCGGAGCCTCTGCTGGCTCCACCACCACCAGCCGGCCGCCGCCGGTGACCGGCGGGAGCAGGGCCCCGGCCTCTCTCAGGGTACGTACTTACACGTGTCCATCGCACGGTCTATCAGTATTTATTTATTAACTACTCTCGAGCTTAATTATGGTATTGTTGATAGTTGATGAAGTTAATTATTGTACGCCGTCTCATCGATCAGTTGGACGACTGGGTGCTGTGCCGCATCTACAAGAAGACCAGCAAGGCCGCGGCCGCGGTCGGAGATGAGCAGAGGAGCATGGAGTGCGAGGACTCCGTGGAGGACGCGGTCACCGCGTACCCGCCCTACGCCACGGCGGGCATGGCCGGCGCAGGTGCGCATGGCAGCAACTACGTTCAACTGCTCCATCATCACGACAGCCACGAGGACAACTTCCAGCTAGACGGCCTGCTCACAGAACACGACGTCGGCCTCTCGGCGGGCGCCGCCTCGCTGGGCCACCTTGCCGCGGCGGCGAGGGCCACCAAACAGTTCCTCGCCCCGTCGTCCTCAACCCCGTTCAACTGGCTCGAGGCGTCAACCGG

>379_*NAM-1*

GCTTTTTATTATACTGTGCACAAGTATTTTTATATTCTTCCAGTAAGTACAGCGCATGTATGTGATCCTGTCGTCGTGCTTGTTCATGCGCTCGGGCGGGATCATCATCCATCAGAGAAGGCGACCTTCGGGGAGCACGAGTGGTACTTCTTCAGCCCGCGCGACCGCAAGTACGCCAACGGCGCGCGGCCGAACCGGGCGGCGACGTCGGGCTACTGGAAGGCCACCGGCACGGACAAGCCTATCCTGGCCTCGGCCACCGGGTGCGGCCGGGAGAAGGTCGGCGTCAAGAAGGCGCTCGTCTTCTACCGCGGGAAGCCGCCCAGGGGCCTCAAGACCAACTGGATCATGCATGAGTACCGCCTCACCGGAGCCTCTGCTGGCTCCACCACCACCAGCCGGCCGCCGCCGGTGACCGGCGGGAGCAGGGCCCCGGCCTCTCTCAGGGTACGTACTTACACGTGTCCATCGCACGGTCTATCAGTATTTATTTATTAACTACTCTCGAGCTTAATTATGGTATTGTTGATAGTTGATGAAGTTAATTATTGTACGCCGTCTCATCGATCAGTTGGACGACTGGGTGCTGTGCCGCATCTACAAGAAGACCAGCAAGGCCGCGGCCGCGGTCGGAGATGAGCAGAGGAGCATGGAGTGCGAGGACTCCGTGGAGGACGCGGTCACCGCGTACCCGCCCTACGCCACGGCGGGCATGGCCGGCGCAGGTGCGCATGGCAGCAACTACGTTCAACTGCTCCATCATCACGACAGCCACGAGGACAACTTCCAGCTAGACGGCCTGCTCACAGAACACGACGTCGGCCTCTCGGCGGGCGCCGCCTCGCTGGGCCACCTTGCCGCGGCGGCGAGGGCCACCAAACAGTTCCTCGCCCCGTCGTCCTCAACCCCGTTCAACTGGCTCGAGGCGTCAACCGG

>380_*NAM-1*

GCTTTTTATTATACTGTGCACAAGTATTTTTATATTCTTCCAGTAAGTACAGCGCATGTATGTGATCCTGTCGTCGTGCTTGTTCATGCGCTCGGGCGGGATCATCATCCATCAGAGAAGGCGACCTTCGGGGAGCACGAGTGGTACTTCTTCAGCCCGCGCGACCGCAAGTACGCCAACGGCGCGCGGCCGAACCGGGCGGCGACGTCGGGCTACTGGAAGGCCACCGGCACGGACAAGCCTATCCTGGCCTCGGCCACCGGGTGCGGCCGGGAGAAGGTCGGCGTCAAGAAGGCGCTCGTCTTCTACCGCGGGAAGCCGCCCAGGGGCCTCAAGACCAACTGGATCATGCATGAGTACCGCCTCACCGGAGCCTCTGCTGGCTCCACCACCACCAGCCGGCCGCCGCCGGTGACCGGCGGGAGCAGGGCCCCGGCCTCTCTCAGGGTACGTACTTACACGTGTCCATCGCACGGTCTATCAGTATTTATTTATTAACTACTCTCGAGCTTAATTATGGTATTGTTGATAGTTGATGAAGTTAATTATTGTACGCCGTCTCATCGATCAGTTGGACGACTGGGTGCTGTGCCGCATCTACAAGAAGACCAGCAAGGCCGCGGCCGCGGTCGGAGATGAGCAGAGGAGCATGGAGTGCGAGGACTCCGTGGAGGACGCGGTCACCGCGTACCCGCCCTACGCCACGGCGGGCATGGCCGGCGCAGGTGCGCATGGCAGCAACTACGTTCAACTGCTCCATCATCACGACAGCCACGAGGACAACTTCCAGCTAGACGGCCTGCTCACAGAACACGACGTCGGCCTCTCGGCGGGCGCCGCCTCGCTGGGCCACCTTGCCGCGGCGGCGAGGGCCACCAAACAGTTCCTCGCCCCGTCGTCCTCAACCCCGTTCAACTGGCTCGAGGCGTCAACCGG

>381_*NAM-1*

GCTTTTTATTATACTGTGCACAAGTATTTTTATATTCTTCCAGTAAGTACAGCGCATGTATGTGATCCTGTCGTCGTGCTTGTTCATGCGCTCGGGCGGGATCATCATCCATCAGAGAAGGCGACCTTCGGGGAGCACGAGTGGTACTTCTTCAGCCCGCGCGACCGCAAGTACGCCAACGGCGCGCGGCCGAACCGGGCGGCGACGTCGGGCTACTGGAAGGCCACCGGCACGGACAAGCCTATCCTGGCCTCGGCCACCGGGTGCGGCCGGGAGAAGGTCGGCGTCAAGAAGGCGCTCGTCTTCTACCGCGGGAAGCCGCCCAGGGGCCTCAAGACCAACTGGATCATGCATGAGTACCGCCTCACCGGAGCCTCTGCTGGCTCCACCACCACCAGCCGGCCGCCGCCGGTGACCGGCGGGAGCAGGGCCCCGGCCTCTCTCAGGGTACGTACTTACACGTGTCCATCGCACGGTCTATCAGTATTTATTTATTAACTACTCTCGAGCTTAATTATGGTATTGTTGATAGTTGATGAAGTTAATTATTGTACGCCGTCTCATCGATCAGTTGGACGACTGGGTGCTGTGCCGCATCTACAAGAAGACCAGCAAGGCCGCGGCCGCGGTCGGAGATGAGCAGAGGAGCATGGAGTGCGAGGACTCCGTGGAGGACGCGGTCACCGCGTACCCGCCCTACGCCACGGCGGGCATGGCCGGCGCAGGTGCGCATGGCAGCAACTACGTTCAACTGCTCCATCATCACGACAGCCACGAGGACAACTTCCAGCTAGACGGCCTGCTCACAGAACACGACGTCGGCCTCTCGGCGGGCGCCGCCTCGCTGGGCCACCTTGCCGCGGCGGCGAGGGCCACCAAACAGTTCCTCGCCCCGTCGTCCTCAACCCCGTTCAACTGGCTCGAGGCGTCAACCGG

>382_*NAM-1*

GCTTTTTATTATACTGTGCACAAGTATTTTTATATTCTTCCAGTAAGTACAGCGCATGTATGTGATCCTGTCGTCGTGCTTGTTCATGCGCTCGGGCGGGATCATCATCCATCAGAGAAGGCGACCTTCGGGGAGCACGAGTGGTACTTCTTCAGCCCGCGCGACCGCAAGTACCCCAACGGCGCGCGGCCGAACCGGGCGGCGACGTCGGGCTACTGGAAGGCCACCGGCACGGACAAGCCTATCCTGGCCTCGGCCACCGGGTGCGGCCGGGAGAAGGTCGGCGTCAAGAAGGCGCTCGTCTTCTACCGCGGGAAGCCGCCCAGGGGCCTCAAGACCAACTGGATCATGCATGAGTACCGCCTCACCGGAGCCTCTGCTGGCTCCACCACCACCAGCCGGCCGCCGCCGGTGACCGGCGGGAGCAGGGCCCCGGCCTCTCTCAGGGTACGTACTTACACGTGTCCATCGCACGGTCTATCAGTATTTATTTATTAACTACTCTCGAGCTTAATTATGGTATTGTTGATAGTTGATGAAGTTAATTATTGTACGCCGTCTCATCGATCAGTTGGACGACTGGGTGCTGTGCCGCATCTACAAGAAGACCAGCAAGGCCGCGGCCGCGGTCGGAGATGAGCAGAGGAGCATGGAGTGCGAGGACTCCGTGGAGGACGCGGTCACCGCGTACCCGCCCTACGCCACGGCGGGCATGGCCGGCGCAGGTGCGCATGGCAGCAACTACGTTCAACTGCTCCATCATCACGACAGCCACGAGGACAACTTCCAGCTAGACGGCCTGCTCACAGAACACGACGTCGGCCTCTCGGCGGGCGCCGCCTCGCTGGGCCACCTTGCCGCGGCGGCGAGGGCCACCAAACAGTTCCTCGCCCCGTCGTCCTCAACCCCGTTCAACTGGCTCGAGGCGTCAACCGG

>406_*NAM-1*

GCTTTTTATTATACTGTGCACAAGTATTTTTATATTCTTCCAGTAAGTACAGCGCATGTATGTGATCCTGTCGTCGTGCTTGTTCATGCGCTCGGGCGGGATCATCATCCATCAGAGAAGGCGACCTTCGGGGAGCACGAGTGGTACTTCTTCAGCCCGCGCGACCGCAAGTACCCCAACGGCGCGCGGCCGAACCGGGCGGCGACGTCGGGCTACTGGAAGGCCACCGGCACGGACAAGCCTATCCTGGCCTCGGCCACCGGGTGCGGCCGGGAGAAGGTCGGCGTCAAGAAGGCGCTCGTCTTCTACCGCGGGAAGCCGCCCAGGGGCCTCAAGACCAACTGGATCATGCATGAGTACCGCCTCACCGGAGCCTCTGCTGGCTCCACCACCACCAGCCGGCCGCCGCCGGTGACCGGCGGGAGCAGGGCCCCGGCCTCTCTCAGGGTACGTACTTACACGTGTCCATCGCACGGTCTATCAGTATTTATTTATTAACTACTCTCGAGCTTAATTATGGTATTGTTGATAGTTGATGAAGTTAATTATTGTACGCCGTCTCATCGATCAGTTGGACGACTGGGTGCTGTGCCGCATCTACAAGAAGACCAGCAAGGCCGCGGCCGCGGTCGGAGATGAGCAGAGGAGCATGGAGTGCGAGGACTCCGTGGAGGACGCGGTCACCGCGTACCCGCCCTACGCCACGGCGGGCATGGCCGGCGCAGGTGCGCATGGCAGCAACTACGTTCAACTGCTCCATCATCACGACAGCCACGAGGACAACTTCCAGCTAGACGGCCTGCTCACAGAACACGACGTCGGCCTCTCGGCGGGCGCCGCCTCGCTGGGCCACCTTGCCGCGGCGGCGAGGGCCACCAAACAGTTCCTCGCCCCGTCGTCCTCAACCCCGTTCAACTGGCTCGAGGCGTCAACCGG

>407_*NAM-1*

GCTTTTTATTATACTGTGCACAAGTATTTTTATATTCTTCCAGTAAGTACAGCGCATGTATGTGATCCTGTCGTCGTGCTTGTTCATGCGCTCGGGCGGGATCATCATCCATCAGAGAAGGCGACCTTCGGGGAGCACGAGTGGTACTTCTTCAGCCCGCGCGACCGCAAGTACCCCAACGGCGCGCGGCCGAACCGGGCGGCGACGTCGGGCTACTGGAAGGCCACCGGCACGGACAAGCCTATCCTGGCCTCGGCCACCGGGTGCGGCCGGGAGAAGGTCGGCGTCAAGAAGGCGCTCGTCTTCTACCGCGGGAAGCCGCCCAGGGGCCTCAAGACCAACTGGATCATGCATGAGTACCGCCTCACCGGAGCCTCTGCTGGCTCCACCACCACCAGCCGGCCGCCGCCGGTGACCGGCGGGAGCAGGGCCCCGGCCTCTCTCAGGGTACGTACTTACACGTGTCCATCGCACGGTCTATCAGTATTTATTTATTAACTACTCTCGAGCTTAATTATGGTATTGTTGATAGTTGATGAAGTTAATTATTGTACGCCGTCTCATCGATCAGTTGGACGACTGGGTGCTGTGCCGCATCTACAAGAAGACCAGCAAGGCCGCGGCCGCGGTCGGAGATGAGCAGAGGAGCATGGAGTGCGAGGACTCCGTGGAGGACGCGGTCACCGCGTACCCGCCCTACGCCACGGCGGGCATGGCCGGCGCAGGTGCGCATGGCAGCAACTACGTTCAACTGCTCCATCATCACGACAGCCACGAGGACAACTTCCAGCTAGACGGCCTGCTCACAGAACACGACGTCGGCCTCTCGGCGGGCGCCGCCTCGCTGGGCCACCTTGCCGCGGCGGCGAGGGCCACCAAACAGTTCCTCGCCCCGTCGTCCTCAACCCCGTTCAACTGGCTCGAGGCGTCAACCGG

>408_*NAM-1*

GCTTTTTATTATACTGTGCACAAGTATTTTTATATTCTTCCAGTAAGTACAGCGCATGTATGTGATCCTGTCGTCGTGCTTGTTCATGCGCTCGGGCGGGATCATCATCCATCAGAGAAGGCGACCTTCGGGGAGCACGAGTGGTACTTCTTCAGCCCGCGCGACCGCAAGTACCCCAACGGCGCGCGGCCGAACCGGGCGGCGACGTCGGGCTACTGGAAGGCCACCGGCACGGACAAGCCTATCCTGGCCTCGGCCACCGGGTGCGGCCGGGAGAAGGTCGGCGTCAAGAAGGCGCTCGTCTTCTACCGCGGGAAGCCGCCCAGGGGCCTCAAGACCAACTGGATCATGCATGAGTACCGCCTCACCGGAGCCTCTGCTGGCTCCACCACCACCAGCCGGCCGCCGCCGGTGACCGGCGGGAGCAGGGCCCCGGCCTCTCTCAGGGTACGTACTTACACGTGTCCATCGCACGGTCTATCAGTATTTATTTATTAACTACTCTCGAGCTTAATTATGGTATTGTTGATAGTTGATGAAGTTAATTATTGTACGCCGTCTCATCGATCAGTTGGACGACTGGGTGCTGTGCCGCATCTACAAGAAGACCAGCAAGGCCGCGGCCGCGGTCGGAGATGAGCAGAGGAGCATGGAGTGCGAGGACTCCGTGGAGGACGCGGTCACCGCGTACCCGCCCTACGCCACGGCGGGCATGGCCGGCGCAGGTGCGCATGGCAGCAACTACGTTCAACTGCTCCATCATCACGACAGCCACGAGGACAACTTCCAGCTAGACGGCCTGCTCACAGAACACGACGTCGGCCTCTCGGCGGGCGCCGCCTCGCTGGGCCACCTTGCCGCGGCGGCGAGGGCCACCAAACAGTTCCTCGCCCCGTCGTCCTCAACCCCGTTCAACTGGCTCGAGGCGTCAACCGG

>409_*NAM-1*

GCTTTTTATTATACTGTGCACAAGTATTTTTATATTCTTCCAGTAAGTACAGCGCATGTATGTGATCCTGTCGTCGTGCTTGTTCATGCGCTCGGGCGGGATCATCATCCATCAGAGAAGGCGACCTTCGGGGAGCACGAGTGGTACTTCTTCAGCCCGCGCGACCGCAAGTACCCCAACGGCGCGCGGCCGAACCGGGCGGCGACGTCGGGCTACTGGAAGGCCACCGGCACGGACAAGCCTATCCTGGCCTCGGCCACCGGGTGCGGCCGGGAGAAGGTCGGCGTCAAGAAGGCGCTCGTCTTCTACCGCGGGAAGCCGCCCAGGGGCCTCAAGACCAACTGGATCATGCATGAGTACCGCCTCACCGGAGCCTCTGCTGGCTCCACCACCACCAGCCGGCCGCCGCCGGTGACCGGCGGGAGCAGGGCCCCGGCCTCTCTCAGGGTACGTACTTACACGTGTCCATCGCACGGTCTATCAGTATTTATTTATTAACTACTCTCGAGCTTAATTATGGTATTGTTGATAGTTGATGAAGTTAATTATTGTACGCCGTCTCATCGATCAGTTGGACGACTGGGTGCTGTGCCGCATCTACAAGAAGACCAGCAAGGCCGCGGCCGCGGTCGGAGATGAGCAGAGGAGCATGGAGTGCGAGGACTCCGTGGAGGACGCGGTCACCGCGTACCCGCCCTACGCCACGGCGGGCATGGCCGGCGCAGGTGCGCATGGCAGCAACTACGTTCAACTGCTCCATCATCACGACAGCCACGAGGACAACTTCCAGCTAGACGGCCTGCTCACAGAACACGACGTCGGCCTCTCGGCGGGCGCCGCCTCGCTGGGCCACCTTGCCGCGGCGGCGAGGGCCACCAAACAGTTCCTCGCCCCGTCGTCCTCAACCCCGTTCAACTGGCTCGAGGCGTCAACCGG

>416_*NAM-1*

GCTTTTTATTATACTGTGCACAAGTATTTTTATATTCTTCCAGTAAGTACAGCGCATGTATGTGATCCTGTCGTCGTGCTTGTTCATGCGCTCGGGCGGGATCATCATCCATCAGAGAAGGCGACCTTCGGGGAGCACGAGTGGTACTTCTTCAGCCCGCGCGACCGCAAGTACGCCAACGGCGCGCGGCCGAACCGGGCGGCGACGTCGGGCTACTGGAAGGCCACCGGCACGGACAAGCCTATCCTGGCCTCGGCCACCGGGTGCGGCCGGGAGAAGGTCGGCGTCAAGAAGGCGCTCGTCTTCTACCGCGGGAAGCCGCCCAGGGGCCTCAAGACCAACTGGATCATGCATGAGTACCGCCTCACCGGAGCCTCTGCTGGCTCCACCACCACCAGCCGGCCGCCGCCGGTGACCGGCGGGAGCAGGGCCCCGGCCTCTCTCAGGGTACGTACTTACACGTGTCCATCGCACGGTCTATCAGTATTTATTTATTAACTACTCTCGAGCTTAATTATGGTATTGTTGATAGTTGATGAAGTTAATTATTGTACGCCGTCTCATCGATCAGTTGGACGACTGGGTGCTGTGCCGCATCTACAAGAAGACCAGCAAGGCCGCGGCCGCGGTCGGAGATGAGCAGAGGAGCATGGAGTGCGAGGACTCCGTGGAGGACGCGGTCACCGCGTACCCGCCCTACGCCACGGCGGGCATGGCCGGCGCAGGTGCGCATGGCAGCAACTACGTTCAACTGCTCCATCATCACGACAGCCACGAGGACAACTTCCAGCTAGACGGCCTGCTCACAGAACACGACGTCGGCCTCTCGGCGGGCGCCGCCTCGCTGGGCCACCTTGCCGCGGCGGCGAGGGCCACCAAACAGTTCCTCGCCCCGTCGTCCTCAACCCCGTTCAACTGGCTCGAGGCGTCAACCGG

>417_*NAM-1*

GCTTTTTATTATACTGTGCACAAGTATTTTTATATTCTTCCAGTAAGTACAGCGCATGTATGTGATCCTGTCGTCGTGCTTGTTCATGCGCTCGGGCGGGATCATCATCCATCAGAGAAGGCGACCTTCGGGGAGCACGAGTGGTACTTCTTCAGCCCGCGCGACCGCAAGTACGCCAACGGCGCGCGGCCGAACCGGGCGGCGACGTCGGGCTACTGGAAGGCCACCGGCACGGACAAGCCTATCCTGGCCTCGGCCACCGGGTGCGGCCGGGAGAAGGTCGGCGTCAAGAAGGCGCTCGTCTTCTACCGCGGGAAGCCGCCCAGGGGCCTCAAGACCAACTGGATCATGCATGAGTACCGCCTCACCGGAGCCTCTGCTGGCTCCACCACCACCAGCCGGCCGCCGCCGGTGACCGGCGGGAGCAGGGCCCCGGCCTCTCTCAGGGTACGTACTTACACGTGTCCATCGCACGGTCTATCAGTATTTATTTATTAACTACTCTCGAGCTTAATTATGGTATTGTTGATAGTTGATGAAGTTAATTATTGTACGCCGTCTCATCGATCAGTTGGACGACTGGGTGCTGTGCCGCATCTACAAGAAGACCAGCAAGGCCGCGGCCGCGGTCGGAGATGAGCAGAGGAGCATGGAGTGCGAGGACTCCGTGGAGGACGCGGTCACCGCGTACCCGCCCTACGCCACGGCGGGCATGGCCGGCGCAGGTGCGCATGGCAGCAACTACGTTCAACTGCTCCATCATCACGACAGCCACGAGGACAACTTCCAGCTAGACGGCCTGCTCACAGAACACGACGTCGGCCTCTCGGCGGGCGCCGCCTCGCTGGGCCACCTTGCCGCGGCGGCGAGGGCCACCAAACAGTTCCTCGCCCCGTCGTCCTCAACCCCGTTCAACTGGCTCGAGGCGTCAACCGG

>418_*NAM-1*

GCTTTTTATTATACTGTGCACAAGTATTTTTATATTCTTCCAGTAAGTACAGCGCATGTATGTGATCCTGTCGTCGTGCTTGTTCATGCGCTCGGGCGGGATCATCATCCATCAGAGAAGGCGACCTTCGGGGAGCACGAGTGGTACTTCTTCAGCCCGCGCGACCGCAAGTACGCCAACGGCGCGCGGCCGAACCGGGCGGCGACGTCGGGCTACTGGAAGGCCACCGGCACGGACAAGCCTATCCTGGCCTCGGCCACCGGGTGCGGCCGGGAGAAGGTCGGCGTCAAGAAGGCGCTCGTCTTCTACCGCGGGAAGCCGCCCAGGGGCCTCAAGACCAACTGGATCATGCATGAGTACCGCCTCACCGGAGCCTCTGCTGGCTCCACCACCACCAGCCGGCCGCCGCCGGTGACCGGCGGGAGCAGGGCCCCGGCCTCTCTCAGGGTACGTACTTACACGTGTCCATCGCACGGTCTATCAGTATTTATTTATTAACTACTCTCGAGCTTAATTATGGTATTGTTGATAGTTGATGAAGTTAATTATTGTACGCCGTCTCATCGATCAGTTGGACGACTGGGTGCTGTGCCGCATCTACAAGAAGACCAGCAAGGCCGCGGCCGCGGTCGGAGATGAGCAGAGGAGCATGGAGTGCGAGGACTCCGTGGAGGACGCGGTCACCGCGTACCCGCCCTACGCCACGGCGGGCATGGCCGGCGCAGGTGCGCATGGCAGCAACTACGTTCAACTGCTCCATCATCACGACAGCCACGAGGACAACTTCCAGCTAGACGGCCTGCTCACAGAACACGACGTCGGCCTCTCGGCGGGCGCCGCCTCGCTGGGCCACCTTGCCGCGGCGGCGAGGGCCACCAAACAGTTCCTCGCCCCGTCGTCCTCAACCCCGTTCAACTGGCTCGAGGCGTCAACCGG

>419_*NAM-1*

GCTTTTTATTATACTGTGCACAAGTATTTTTATATTCTTCCAGTAAGTACAGCGCATGTATGTGATCCTGTCGTCGTGCTTGTTCATGCGCTCGGGCGGGATCATCATCCATCAGAGAAGGCGACCTTCGGGGAGCACGAGTGGTACTTCTTCAGCCCGCGCGACCGCAAGTACGCCAACGGCGCGCGGCCGAACCGGGCGGCGACGTCGGGCTACTGGAAGGCCACCGGCACGGACAAGCCTATCCTGGCCTCGGCCACCGGGTGCGGCCGGGAGAAGGTCGGCGTCAAGAAGGCGCTCGTCTTCTACCGCGGGAAGCCGCCCAGGGGCCTCAAGACCAACTGGATCATGCATGAGTACCGCCTCACCGGAGCCTCTGCTGGCTCCACCACCACCAGCCGGCCGCCGCCGGTGACCGGCGGGAGCAGGGCCCCGGCCTCTCTCAGGGTACGTACTTACACGTGTCCATCGCACGGTCTATCAGTATTTATTTATTAACTACTCTCGAGCTTAATTATGGTATTGTTGATAGTTGATGAAGTTAATTATTGTACGCCGTCTCATCGATCAGTTGGACGACTGGGTGCTGTGCCGCATCTACAAGAAGACCAGCAAGGCCGCGGCCGCGGTCGGAGATGAGCAGAGGAGCATGGAGTGCGAGGACTCCGTGGAGGACGCGGTCACCGCGTACCCGCCCTACGCCACGGCGGGCATGGCCGGCGCAGGTGCGCATGGCAGCAACTACGTTCAACTGCTCCATCATCACGACAGCCACGAGGACAACTTCCAGCTAGACGGCCTGCTCACAGAACACGACGTCGGCCTCTCGGCGGGCGCCGCCTCGCTGGGCCACCTTGCCGCGGCGGCGAGGGCCACCAAACAGTTCCTCGCCCCGTCGTCCTCAACCCCGTTCAACTGGCTCGAGGCGTCAACCGG

>420_*NAM-1*

GCTTTTTATTATACTGTGCACAAGTATTTTTATATTCTTCCAGTAAGTACAGCGCATGTATGTGATCCTGTCGTCGTGCTTGTTCATGCGCTCGGGCGGGATCATCATCCATCAGAGAAGGCGACCTTCGGGGAGCACGAGTGGTACTTCTTCAGCCCGCGCGACCGCAAGTACCCCAACGGCGCGCGGCCGAACCGGGCGGCGACGTCGGGCTACTGGAAGGCCACCGGCACGGACAAGCCTATCCTGGCCTCGGCCACCGGGTGCGGCCGGGAGAAGGTCGGCGTCAAGAAGGCGCTCGTCTTCTACCGCGGGAAGCCGCCCAGGGGCCTCAAGACCAACTGGATCATGCATGAGTACCGCCTCACCGGAGCCTCTGCTGGCTCCACCACCACCAGCCGGCCGCCGCCGGTGACCGGCGGGAGCAGGGCCCCGGCCTCTCTCAGGGTACGTACTTACACGTGTCCATCGCACGGTCTATCAGTATTTATTTATTAACTACTCTCGAGCTTAATTATGGTATTGTTGATAGTTGATGAAGTTAATTATTGTACGCCGTCTCATCGATCAGTTGGACGACTGGGTGCTGTGCCGCATCTACAAGAAGACCAGCAAGGCCGCGGCCGCGGTCGGAGATGAGCAGAGGAGCATGGAGTGCGAGGACTCCGTGGAGGACGCGGTCACCGCGTACCCGCCCTACGCCACGGCGGGCATGGCCGGCGCAGGTGCGCATGGCAGCAACTACGTTCAACTGCTCCATCATCACGACAGCCACGAGGACAACTTCCAGCTAGACGGCCTGCTCACAGAACACGACGTCGGCCTCTCGGCGGGCGCCGCCTCGCTGGGCCACCTTGCCGCGGCGGCGAGGGCCACCAAACAGTTCCTCGCCCCGTCGTCCTCAACCCCGTTCAACTGGCTCGAGGCGTCAACCGG

>424_*NAM-1*

GCTTTTTATTATACTGTGCACAAGTATTTTTATATTCTTCCAGTAAGTACAGCGCATGTATGTGATCCTGTCGTCGTGCTTGTTCATGCGCTCGGGCGGGATCATCATCCATCAGAGAAGGCGACCTTCGGGGAGCACGAGTGGTACTTCTTCAGCCCGCGCGACCGCAAGTACGCCAACGGCGCGCGGCCGAACCGGGCGGCGACGTCGGGCTACTGGAAGGCCACCGGCACGGACAAGCCTATCCTGGCCTCGGCCACCGGGTGCGGCCGGGAGAAGGTCGGCGTCAAGAAGGCGCTCGTCTTCTACCGCGGGAAGCCGCCCAGGGGCCTCAAGACCAACTGGATCATGCATGAGTACCGCCTCACCGGAGCCTCTGCTGGCTCCACCACCACCAGCCGGCCGCCGCCGGTGACCGGCGGGAGCAGGGCCCCGGCCTCTCTCAGGGTACGTACTTACACGTGTCCATCGCACGGTCTATCAGTATTTATTTATTAACTACTCTCGAGCTTAATTATGGTATTGTTGATAGTTGATGAAGTTAATTATTGTACGCCGTCTCATCGATCAGTTGGACGACTGGGTGCTGTGCCGCATCTACAAGAAGACCAGCAAGGCCGCGGCCGCGGTCGGAGATGAGCAGAGGAGCATGGAGTGCGAGGACTCCGTGGAGGACGCGGTCACCGCGTACCCGCCCTACGCCACGGCGGGCATGGCCGGCGCAGGTGCGCATGGCAGCAACTACGTTCAACTGCTCCATCATCACGACAGCCACGAGGACAACTTCCAGCTAGACGGCCTGCTCACAGAACACGACGTCGGCCTCTCGGCGGGCGCCGCCTCGCTGGGCCACCTTGCCGCGGCGGCGAGGGCCACCAAACAGTTCCTCGCCCCGTCGTCCTCAACCCCGTTCAACTGGCTCGAGGCGTCAACCGG

>425_*NAM-1*

GCTTTTTATTATACTGTGCACAAGTATTTTTATATTCTTCCAGTAAGTACAGCGCATGTATGTGATCCTGTCGTCGTGCTTGTTCATGCGCTCGGGCGGGATCATCATCCATCAGAGAAGGCGACCTTCGGGGAGCACGAGTGGTACTTCTTCAGCCCGCGCGACCGCAAGTACGCCAACGGCGCGCGGCCGAACCGGGCGGCGACGTCGGGCTACTGGAAGGCCACCGGCACGGACAAGCCTATCCTGGCCTCGGCCACCGGGTGCGGCCGGGAGAAGGTCGGCGTCAAGAAGGCGCTCGTCTTCTACCGCGGGAAGCCGCCCAGGGGCCTCAAGACCAACTGGATCATGCATGAGTACCGCCTCACCGGAGCCTCTGCTGGCTCCACCACCACCAGCCGGCCGCCGCCGGTGACCGGCGGGAGCAGGGCCCCGGCCTCTCTCAGGGTACGTACTTACACGTGTCCATCGCACGGTCTATCAGTATTTATTTATTAACTACTCTCGAGCTTAATTATGGTATTGTTGATAGTTGATGAAGTTAATTATTGTACGCCGTCTCATCGATCAGTTGGACGACTGGGTGCTGTGCCGCATCTACAAGAAGACCAGCAAGGCCGCGGCCGCGGTCGGAGATGAGCAGAGGAGCATGGAGTGCGAGGACTCCGTGGAGGACGCGGTCACCGCGTACCCGCCCTACGCCACGGCGGGCATGGCCGGCGCAGGTGCGCATGGCAGCAACTACGTTCAACTGCTCCATCATCACGACAGCCACGAGGACAACTTCCAGCTAGACGGCCTGCTCACAGAACACGACGTCGGCCTCTCGGCGGGCGCCGCCTCGCTGGGCCACCTTGCCGCGGCGGCGAGGGCCACCAAACAGTTCCTCGCCCCGTCGTCCTCAACCCCGTTCAACTGGCTCGAGGCGTCAACCGG

>426_*NAM-1*

GCTTTTTATTATACTGTGCACAAGTATTTTTATATTCTTCCAGTAAGTACAGCGCATGTATGTGATCCTGTCGTCGTGCTTGTTCATGCGCTCGGGCGGGATCATCATCCATCAGAGAAGGCGACCTTCGGGGAGCACGAGTGGTACTTCTTCAGCCCGCGCGACCGCAAGTACGCCAACGGCGCGCGGCCGAACCGGGCGGCGACGTCGGGCTACTGGAAGGCCACCGGCACGGACAAGCCTATCCTGGCCTCGGCCACCGGGTGCGGCCGGGAGAAGGTCGGCGTCAAGAAGGCGCTCGTCTTCTACCGCGGGAAGCCGCCCAGGGGCCTCAAGACCAACTGGATCATGCATGAGTACCGCCTCACCGGAGCCTCTGCTGGCTCCACCACCACCAGCCGGCCGCCGCCGGTGACCGGCGGGAGCAGGGCCCCGGCCTCTCTCAGGGTACGTACTTACACGTGTCCATCGCACGGTCTATCAGTATTTATTTATTAACTACTCTCGAGCTTAATTATGGTATTGTTGATAGTTGATGAAGTTAATTATTGTACGCCGTCTCATCGATCAGTTGGACGACTGGGTGCTGTGCCGCATCTACAAGAAGACCAGCAAGGCCGCGGCCGCGGTCGGAGATGAGCAGAGGAGCATGGAGTGCGAGGACTCCGTGGAGGACGCGGTCACCGCGTACCCGCCCTACGCCACGGCGGGCATGGCCGGCGCAGGTGCGCATGGCAGCAACTACGTTCAACTGCTCCATCATCACGACAGCCACGAGGACAACTTCCAGCTAGACGGCCTGCTCACAGAACACGACGTCGGCCTCTCGGCGGGCGCCGCCTCGCTGGGCCACCTTGCCGCGGCGGCGAGGGCCACCAAACAGTTCCTCGCCCCGTCGTCCTCAACCCCGTTCAACTGGCTCGAGGCGTCAACCGG

>427_*NAM-1*

GCTTTTTATTATACTGTGCACAAGTATTTTTATATTCTTCCAGTAAGTACAGCGCATGTATGTGATCCTGTCGTCGTGCTTGTTCATGCGCTCGGGCGGGATCATCATCCATCAGAGAAGGCGACCTTCGGGGAGCACGAGTGGTACTTCTTCAGCCCGCGCGACCGCAAGTACGCCAACGGCGCGCGGCCGAACCGGGCGGCGACGTCGGGCTACTGGAAGGCCACCGGCACGGACAAGCCTATCCTGGCCTCGGCCACCGGGTGCGGCCGGGAGAAGGTCGGCGTCAAGAAGGCGCTCGTCTTCTACCGCGGGAAGCCGCCCAGGGGCCTCAAGACCAACTGGATCATGCATGAGTACCGCCTCACCGGAGCCTCTGCTGGCTCCACCACCACCAGCCGGCCGCCGCCGGTGACCGGCGGGAGCAGGGCCCCGGCCTCTCTCAGGGTACGTACTTACACGTGTCCATCGCACGGTCTATCAGTATTTATTTATTAACTACTCTCGAGCTTAATTATGGTATTGTTGATAGTTGATGAAGTTAATTATTGTACGCCGTCTCATCGATCAGTTGGACGACTGGGTGCTGTGCCGCATCTACAAGAAGACCAGCAAGGCCGCGGCCGCGGTCGGAGATGAGCAGAGGAGCATGGAGTGCGAGGACTCCGTGGAGGACGCGGTCACCGCGTACCCGCCCTACGCCACGGCGGGCATGGCCGGCGCAGGTGCGCATGGCAGCAACTACGTTCAACTGCTCCATCATCACGACAGCCACGAGGACAACTTCCAGCTAGACGGCCTGCTCACAGAACACGACGTCGGCCTCTCGGCGGGCGCCGCCTCGCTGGGCCACCTTGCCGCGGCGGCGAGGGCCACCAAACAGTTCCTCGCCCCGTCGTCCTCAACCCCGTTCAACTGGCTCGAGGCGTCAACCGG

>428_*NAM-1*

GCTTTTTATTATACTGTGCACAAGTATTTTTATATTCTTCCAGTAAGTACAGCGCATGTATGTGATCCTGTCGTCGTGCTTGTTCATGCGCTCGGGCGGGATCATCATCCATCAGAGAAGGCGACCTTCGGGGAGCACGAGTGGTACTTCTTCAGCCCGCGCGACCGCAAGTACGCCAACGGCGCGCGGCCGAACCGGGCGGCGACGTCGGGCTACTGGAAGGCCACCGGCACGGACAAGCCTATCCTGGCCTCGGCCACCGGGTGCGGCCGGGAGAAGGTCGGCGTCAAGAAGGCGCTCGTCTTCTACCGCGGGAAGCCGCCCAGGGGCCTCAAGACCAACTGGATCATGCATGAGTACCGCCTCACCGGAGCCTCTGCTGGCTCCACCACCACCAGCCGGCCGCCGCCGGTGACCGGCGGGAGCAGGGCCCCGGCCTCTCTCAGGGTACGTACTTACACGTGTCCATCGCACGGTCTATCAGTATTTATTTATTAACTACTCTCGAGCTTAATTATGGTATTGTTGATAGTTGATGAAGTTAATTATTGTACGCCGTCTCATCGATCAGTTGGACGACTGGGTGCTGTGCCGCATCTACAAGAAGACCAGCAAGGCCGCGGCCGCGGTCGGAGATGAGCAGAGGAGCATGGAGTGCGAGGACTCCGTGGAGGACGCGGTCACCGCGTACCCGCCCTACGCCACGGCGGGCATGGCCGGCGCAGGTGCGCATGGCAGCAACTACGTTCAACTGCTCCATCATCACGACAGCCACGAGGACAACTTCCAGCTAGACGGCCTGCTCACAGAACACGACGTCGGCCTCTCGGCGGGCGCCGCCTCGCTGGGCCACCTTGCCGCGGCGGCGAGGGCCACCAAACAGTTCCTCGCCCCGTCGTCCTCAACCCCGTTCAACTGGCTCGAGGCGTCAACCGG

>429_*NAM-1*

GCTTTTTATTATACTGTGCACAAGTATTTTTATATTCTTCCAGTAAGTACAGCGCATGTATGTGATCCTGTCGTCGTGCTTGTTCATGCGCTCGGGCGGGATCATCATCCATCAGAGAAGGCGACCTTCGGGGAGCACGAGTGGTACTTCTTCAGCCCGCGCGACCGCAAGTACGCCAACGGCGCGCGGCCGAACCGGGCGGCGACGTCGGGCTACTGGAAGGCCACCGGCACGGACAAGCCTATCCTGGCCTCGGCCACCGGGTGCGGCCGGGAGAAGGTCGGCGTCAAGAAGGCGCTCGTCTTCTACCGCGGGAAGCCGCCCAGGGGCCTCAAGACCAACTGGATCATGCATGAGTACCGCCTCACCGGAGCCTCTGCTGGCTCCACCACCACCAGCCGGCCGCCGCCGGTGACCGGCGGGAGCAGGGCCCCGGCCTCTCTCAGGGTACGTACTTACACGTGTCCATCGCACGGTCTATCAGTATTTATTTATTAACTACTCTCGAGCTTAATTATGGTATTGTTGATAGTTGATGAAGTTAATTATTGTACGCCGTCTCATCGATCAGTTGGACGACTGGGTGCTGTGCCGCATCTACAAGAAGACCAGCAAGGCCGCGGCCGCGGTCGGAGATGAGCAGAGGAGCATGGAGTGCGAGGACTCCGTGGAGGACGCGGTCACCGCGTACCCGCCCTACGCCACGGCGGGCATGGCCGGCGCAGGTGCGCATGGCAGCAACTACGTTCAACTGCTCCATCATCACGACAGCCACGAGGACAACTTCCAGCTAGACGGCCTGCTCACAGAACACGACGTCGGCCTCTCGGCGGGCGCCGCCTCGCTGGGCCACCTTGCCGCGGCGGCGAGGGCCACCAAACAGTTCCTCGCCCCGTCGTCCTCAACCCCGTTCAACTGGCTCGAGGCGTCAACCGG

>430_*NAM-1*

GCTTTTTATTATACTGTGCACAAGTATTTTTATATTCTTCCAGTAAGTACAGCGCATGTATGTGATCCTGTCGTCGTGCTTGTTCATGCGCTCGGGCGGGATCATCATCCATCAGAGAAGGCGACCTTCGGGGAGCACGAGTGGTACTTCTTCAGCCCGCGCGACCGCAAGTACGCCAACGGCGCGCGGCCGAACCGGGCGGCGACGTCGGGCTACTGGAAGGCCACCGGCACGGACAAGCCTATCCTGGCCTCGGCCACCGGGTGCGGCCGGGAGAAGGTCGGCGTCAAGAAGGCGCTCGTCTTCTACCGCGGGAAGCCGCCCAGGGGCCTCAAGACCAACTGGATCATGCATGAGTACCGCCTCACCGGAGCCTCTGCTGGCTCCACCACCACCAGCCGGCCGCCGCCGGTGACCGGCGGGAGCAGGGCCCCGGCCTCTCTCAGGGTACGTACTTACACGTGTCCATCGCACGGTCTATCAGTATTTATTTATTAACTACTCTCGAGCTTAATTATGGTATTGTTGATAGTTGATGAAGTTAATTATTGTACGCCGTCTCATCGATCAGTTGGACGACTGGGTGCTGTGCCGCATCTACAAGAAGACCAGCAAGGCCGCGGCCGCGGTCGGAGATGAGCAGAGGAGCATGGAGTGCGAGGACTCCGTGGAGGACGCGGTCACCGCGTACCCGCCCTACGCCACGGCGGGCATGGCCGGCGCAGGTGCGCATGGCAGCAACTACGTTCAACTGCTCCATCATCACGACAGCCACGAGGACAACTTCCAGCTAGACGGCCTGCTCACAGAACACGACGTCGGCCTCTCGGCGGGCGCCGCCTCGCTGGGCCACCTTGCCGCGGCGGCGAGGGCCACCAAACAGTTCCTCGCCCCGTCGTCCTCAACCCCGTTCAACTGGCTCGAGGCGTCAACCGG

>489_*NAM-1*

GCTTTTTATTATACTGTGCACAAGTATTTTTATATTCTTCCAGTAAGTACAGCGCATGTATGTGATCCTGTCGTCGTGCTTGTTCATGCGCTCGGGCGGGATCATCATCCATCAGAGAAGGCGACCTTCGGGGAGCACGAGTGGTACTTCTTCAGCCCGCGCGACCGCAAGTACGCCAACGGCGCGCGGCCGAACCGGGCGGCGACGTCGGGCTACTGGAAGGCCACCGGCACGGACAAGCCTATCCTGGCCTCGGCCACCGGGTGCGGCCGGGAGAAGGTCGGCGTCAAGAAGGCGCTCGTCTTCTACCGCGGGAAGCCGCCCAGGGGCCTCAAGACCAACTGGATCATGCATGAGTACCGCCTCACCGGAGCCTCTGCTGGCTCCACCACCACCAGCCGGCCGCCGCCGGTGACCGGCGGGAGCAGGGCCCCGGCCTCTCTCAGGGTACGTACTTACACGTGTCCATCGCACGGTCTATCAGTATTTATTTATTAACTACTCTCGAGCTTAATTATGGTATTGTTGATAGTTGATGAAGTTAATTATTGTACGCCGTCTCATCGATCAGTTGGACGACTGGGTGCTGTGCCGCATCTACAAGAAGACCAGCAAGGCCGCGGCCGCGGTCGGAGATGAGCAGAGGAGCATGGAGTGCGAGGACTCCGTGGAGGACGCGGTCACCGCGTACCCGCCCTACGCCACGGCGGGCATGGCCGGCGCAGGTGCGCATGGCAGCAACTACGTTCAACTGCTCCATCATCACGACAGCCACGAGGACAACTTCCAGCTAGACGGCCTGCTCACAGAACACGACGTCGGCCTCTCGGCGGGCGCCGCCTCGCTGGGCCACCTTGCCGCGGCGGCGAGGGCCACCAAACAGTTCCTCGCCCCGTCGTCCTCAACCCCGTTCAACTGGCTCGAGGCGTCAACCGG

>490_*NAM-1*

GCTTTTTATTATACTGTGCACAAGTATTTTTATATTCTTCCAGTAAGTACAGCGCATGTATGTGATCCTGTCGTCGTGCTTGTTCATGCGCTCGGGCGGGATCATCATCCATCAGAGAAGGCGACCTTCGGGGAGCACGAGTGGTACTTCTTCAGCCCGCGCGACCGCAAGTACCCCAACGGCGCGCGGCCGAACCGGGCGGCGACGTCGGGCTACTGGAAGGCCACCGGCACGGACAAGCCTATCCTGGCCTCGGCCACCGGGTGCGGCCGGGAGAAGGTCGGCGTCAAGAAGGCGCTCGTCTTCTACCGCGGGAAGCCGCCCAGGGGCCTCAAGACCAACTGGATCATGCATGAGTACCGCCTCACCGGAGCCTCTGCTGGCTCCACCACCACCAGCCGGCCGCCGCCGGTGACCGGCGGGAGCAGGGCCCCGGCCTCTCTCAGGGTACGTACTTACACGTGTCCATCGCACGGTCTATCAGTATTTATTTATTAACTACTCTCGAGCTTAATTATGGTATTGTTGATAGTTGATGAAGTTAATTATTGTACGCCGTCTCATCGATCAGTTGGACGACTGGGTGCTGTGCCGCATCTACAAGAAGACCAGCAAGGCCGCGGCCGCGGTCGGAGATGAGCAGAGGAGCATGGAGTGCGAGGACTCCGTGGAGGACGCGGTCACCGCGTACCCGCCCTACGCCACGGCGGGCATGGCCGGCGCAGGTGCGCATGGCAGCAACTACGTTCAACTGCTCCATCATCACGACAGCCACGAGGACAACTTCCAGCTAGACGGCCTGCTCACAGAACACGACGTCGGCCTCTCGGCGGGCGCCGCCTCGCTGGGCCACCTTGCCGCGGCGGCGAGGGCCACCAAACAGTTCCTCGCCCCGTCGTCCTCAACCCCGTTCAACTGGCTCGAGGCGTCAACCGG

>491_*NAM-1*

GCTTTTTATTATACTGTGCACAAGTATTTTTATATTCTTCCAGTAAGTACAGCGCATGTATGTGATCCTGTCGTCGTGCTTGTTCATGCGCTCGGGCGGGATCATCATCCATCAGAGAAGGCGACCTTCGGGGAGCACGAGTGGTACTTCTTCAGCCCGCGCGACCGCAAGTACGCCAACGGCGCGCGGCCGAACCGGGCGGCGACGTCGGGCTACTGGAAGGCCACCGGCACGGACAAGCCTATCCTGGCCTCGGCCACCGGGTGCGGCCGGGAGAAGGTCGGCGTCAAGAAGGCGCTCGTCTTCTACCGCGGGAAGCCGCCCAGGGGCCTCAAGACCAACTGGATCATGCATGAGTACCGCCTCACCGGAGCCTCTGCTGGCTCCACCACCACCAGCCGGCCGCCGCCGGTGACCGGCGGGAGCAGGGCCCCGGCCTCTCTCAGGGTACGTACTTACACGTGTCCATCGCACGGTCTATCAGTATTTATTTATTAACTACTCTCGAGCTTAATTATGGTATTGTTGATAGTTGATGAAGTTAATTATTGTACGCCGTCTCATCGATCAGTTGGACGACTGGGTGCTGTGCCGCATCTACAAGAAGACCAGCAAGGCCGCGGCCGCGGTCGGAGATGAGCAGAGGAGCATGGAGTGCGAGGACTCCGTGGAGGACGCGGTCACCGCGTACCCGCCCTACGCCACGGCGGGCATGGCCGGCGCAGGTGCGCATGGCAGCAACTACGTTCAACTGCTCCATCATCACGACAGCCACGAGGACAACTTCCAGCTAGACGGCCTGCTCACAGAACACGACGTCGGCCTCTCGGCGGGCGCCGCCTCGCTGGGCCACCTTGCCGCGGCGGCGAGGGCCACCAAACAGTTCCTCGCCCCGTCGTCCTCAACCCCGTTCAACTGGCTCGAGGCGTCAACCGG

>494_*NAM-1*

GCTTTTTATTATACTGTGCACAAGTATTTTTATATTCTTCCAGTAAGTACAGCGCATGTATGTGATCCTGTCGTCGTGCTTGTTCATGCGCTCGGGCGGGATCATCATCCATCAGAGAAGGCGACCTTCGGGGAGCACGAGTGGTACTTCTTCAGCCCGCGCGACCGCAAGTACCCCAACGGCGCGCGGCCGAACCGGGCGGCGACGTCGGGCTACTGGAAGGCCACCGGCACGGACAAGCCTATCCTGGCCTCGGCCACCGGGTGCGGCCGGGAGAAGGTCGGCGTCAAGAAGGCGCTCGTCTTCTACCGCGGGAAGCCGCCCAGGGGCCTCAAGACCAACTGGATCATGCATGAGTACCGCCTCACCGGAGCCTCTGCTGGCTCCACCACCACCAGCCGGCCGCCGCCGGTGACCGGCGGGAGCAGGGCCCCGGCCTCTCTCAGGGTACGTACTTACACGTGTCCATCGCACGGTCTATCAGTATTTATTTATTAACTACTCTCGAGCTTAATTATGGTATTGTTGATAGTTGATGAAGTTAATTATTGTACGCCGTCTCATCGATCAGTTGGACGACTGGGTGCTGTGCCGCATCTACAAGAAGACCAGCAAGGCCGCGGCCGCGGTCGGAGATGAGCAGAGGAGCATGGAGTGCGAGGACTCCGTGGAGGACGCGGTCACCGCGTACCCGCCCTACGCCACGGCGGGCATGGCCGGCGCAGGTGCGCATGGCAGCAACTACGTTCAACTGCTCCATCATCACGACAGCCACGAGGACAACTTCCAGCTAGACGGCCTGCTCACAGAACACGACGTCGGCCTCTCGGCGGGCGCCGCCTCGCTGGGCCACCTTGCCGCGGCGGCGAGGGCCACCAAACAGTTCCTCGCCCCGTCGTCCTCAACCCCGTTCAACTGGCTCGAGGCGTCAACCGG

>495_*NAM-1*

GCTTTTTATTATACTGTGCACAAGTATTTTTATATTCTTCCAGTAAGTACAGCGCATGTATGTGATCCTGTCGTCGTGCTTGTTCATGCGCTCGGGCGGGATCATCATCCATCAGAGAAGGCGACCTTCGGGGAGCACGAGTGGTACTTCTTCAGCCCGCGCGACCGCAAGTACCCCAACGGCGCGCGGCCGAACCGGGCGGCGACGTCGGGCTACTGGAAGGCCACCGGCACGGACAAGCCTATCCTGGCCTCGGCCACCGGGTGCGGCCGGGAGAAGGTCGGCGTCAAGAAGGCGCTCGTCTTCTACCGCGGGAAGCCGCCCAGGGGCCTCAAGACCAACTGGATCATGCATGAGTACCGCCTCACCGGAGCCTCTGCTGGCTCCACCACCACCAGCCGGCCGCCGCCGGTGACCGGCGGGAGCAGGGCCCCGGCCTCTCTCAGGGTACGTACTTACACGTGTCCATCGCACGGTCTATCAGTATTTATTTATTAACTACTCTCGAGCTTAATTATGGTATTGTTGATAGTTGATGAAGTTAATTATTGTACGCCGTCTCATCGATCAGTTGGACGACTGGGTGCTGTGCCGCATCTACAAGAAGACCAGCAAGGCCGCGGCCGCGGTCGGAGATGAGCAGAGGAGCATGGAGTGCGAGGACTCCGTGGAGGACGCGGTCACCGCGTACCCGCCCTACGCCACGGCGGGCATGGCCGGCGCAGGTGCGCATGGCAGCAACTACGTTCAACTGCTCCATCATCACGACAGCCACGAGGACAACTTCCAGCTAGACGGCCTGCTCACAGAACACGACGTCGGCCTCTCGGCGGGCGCCGCCTCGCTGGGCCACCTTGCCGCGGCGGCGAGGGCCACCAAACAGTTCCTCGCCCCGTCGTCCTCAACCCCGTTCAACTGGCTCGAGGCGTCAACCGG

>533_*NAM-1*

GCTTTTTATTATACTGTGCACAAGTATTTTTATATTCTTCCAGTAAGTACAGCGCATGTATGTGATCCTGTCGTCGTGCTTGTTCATGCGCTCGGGCGGGATCATCATCCATCAGAGAAGGCGACCTTCGGGGAGCACGAGTGGTACTTCTTCAGCCCGCGCGACCGCAAGTACGCCAACGGCGCGCGGCCGAACCGGGCGGCGACGTCGGGCTACTGGAAGGCCACCGGCACGGACAAGCCTATCCTGGCCTCGGCCACCGGGTGCGGCCGGGAGAAGGTCGGCGTCAAGAAGGCGCTCGTCTTCTACCGCGGGAAGCCGCCCAGGGGCCTCAAGACCAACTGGATCATGCATGAGTACCGCCTCACCGGAGCCTCTGCTGGCTCCACCACCACCAGCCGGCCGCCGCCGGTGACCGGCGGGAGCAGGGCCCCGGCCTCTCTCAGGGTACGTACTTACACGTGTCCATCGCACGGTCTATCAGTATTTATTTATTAACTACTCTCGAGCTTAATTATGGTATTGTTGATAGTTGATGAAGTTAATTATTGTACGCCGTCTCATCGATCAGTTGGACGACTGGGTGCTGTGCCGCATCTACAAGAAGACCAGCAAGGCCGCGGCCGCGGTCGGAGATGAGCAGAGGAGCATGGAGTGCGAGGACTCCGTGGAGGACGCGGTCACCGCGTACCCGCCCTACGCCACGGCGGGCATGGCCGGCGCAGGTGCGCATGGCAGCAACTACGTTCAACTGCTCCATCATCACGACAGCCACGAGGACAACTTCCAGCTAGACGGCCTGCTCACAGAACACGACGTCGGCCTCTCGGCGGGCGCCGCCTCGCTGGGCCACCTTGCCGCGGCGGCGAGGGCCACCAAACAGTTCCTCGCCCCGTCGTCCTCAACCCCGTTCAACTGGCTCGAGGCGTCAACCGG

>539_*NAM-1*

GCTTTTTATTATACTGTGCACAAGTATTTTTATATTCTTCCAGTAAGTACAGCGCATGTATGTGATCCTGTCGTCGTGCTTGTTCATGCGCTCGGGCGGGATCATCATCCATCAGAGAAGGCGACCTTCGGGGAGCACGAGTGGTACTTCTTCAGCCCGCGCGACCGCAAGTACGCCAACGGCGCGCGGCCGAACCGGGCGGCGACGTCGGGCTACTGGAAGGCCACCGGCACGGACAAGCCTATCCTGGCCTCGGCCACCGGGTGCGGCCGGGAGAAGGTCGGCGTCAAGAAGGCGCTCGTCTTCTACCGCGGGAAGCCGCCCAGGGGCCTCAAGACCAACTGGATCATGCATGAGTACCGCCTCACCGGAGCCTCTGCTGGCTCCACCACCACCAGCCGGCCGCCGCCGGTGACCGGCGGGAGCAGGGCCCCGGCCTCTCTCAGGGTACGTACTTACACGTGTCCATCGCACGGTCTATCAGTATTTATTTATTAACTACTCTCGAGCTTAATTATGGTATTGTTGATAGTTGATGAAGTTAATTATTGTACGCCGTCTCATCGATCAGTTGGACGACTGGGTGCTGTGCCGCATCTACAAGAAGACCAGCAAGGCCGCGGCCGCGGTCGGAGATGAGCAGAGGAGCATGGAGTGCGAGGACTCCGTGGAGGACGCGGTCACCGCGTACCCGCCCTACGCCACGGCGGGCATGGCCGGCGCAGGTGCGCATGGCAGCAACTACGTTCAACTGCTCCATCATCACGACAGCCACGAGGACAACTTCCAGCTAGACGGCCTGCTCACAGAACACGACGTCGGCCTCTCGGCGGGCGCCGCCTCGCTGGGCCACCTTGCCGCGGCGGCGAGGGCCACCAAACAGTTCCTCGCCCCGTCGTCCTCAACCCCGTTCAACTGGCTCGAGGCGTCAACCGG

>540_*NAM-1*

GCTTTTTATTATACTGTGCACAAGTATTTTTATATTCTTCCAGTAAGTACAGCGCATGTATGTGATCCTGTCGTCGTGCTTGTTCATGCGCTCGGGCGGGATCATCATCCATCAGAGAAGGCGACCTTCGGGGAGCACGAGTGGTACTTCTTCAGCCCGCGCGACCGCAAGTACGCCAACGGCGCGCGGCCGAACCGGGCGGCGACGTCGGGCTACTGGAAGGCCACCGGCACGGACAAGCCTATCCTGGCCTCGGCCACCGGGTGCGGCCGGGAGAAGGTCGGCGTCAAGAAGGCGCTCGTCTTCTACCGCGGGAAGCCGCCCAGGGGCCTCAAGACCAACTGGATCATGCATGAGTACCGCCTCACCGGAGCCTCTGCTGGCTCCACCACCACCAGCCGGCCGCCGCCGGTGACCGGCGGGAGCAGGGCCCCGGCCTCTCTCAGGGTACGTACTTACACGTGTCCATCGCACGGTCTATCAGTATTTATTTATTAACTACTCTCGAGCTTAATTATGGTATTGTTGATAGTTGATGAAGTTAATTATTGTACGCCGTCTCATCGATCAGTTGGACGACTGGGTGCTGTGCCGCATCTACAAGAAGACCAGCAAGGCCGCGGCCGCGGTCGGAGATGAGCAGAGGAGCATGGAGTGCGAGGACTCCGTGGAGGACGCGGTCACCGCGTACCCGCCCTACGCCACGGCGGGCATGGCCGGCGCAGGTGCGCATGGCAGCAACTACGTTCAACTGCTCCATCATCACGACAGCCACGAGGACAACTTCCAGCTAGACGGCCTGCTCACAGAACACGACGTCGGCCTCTCGGCGGGCGCCGCCTCGCTGGGCCACCTTGCCGCGGCGGCGAGGGCCACCAAACAGTTCCTCGCCCCGTCGTCCTCAACCCCGTTCAACTGGCTCGAGGCGTCAACCGG

>541_*NAM-1*

GCTTTTTATTATACTGTGCACAAGTATTTTTATATTCTTCCAGTAAGTACAGCGCATGTATGTGATCCTGTCGTCGTGCTTGTTCATGCGCTCGGGCGGGATCATCATCCATCAGAGAAGGCGACCTTCGGGGAGCACGAGTGGTACTTCTTCAGCCCGCGCGACCGCAAGTACGCCAACGGCGCGCGGCCGAACCGGGCGGCGACGTCGGGCTACTGGAAGGCCACCGGCACGGACAAGCCTATCCTGGCCTCGGCCACCGGGTGCGGCCGGGAGAAGGTCGGCGTCAAGAAGGCGCTCGTCTTCTACCGCGGGAAGCCGCCCAGGGGCCTCAAGACCAACTGGATCATGCATGAGTACCGCCTCACCGGAGCCTCTGCTGGCTCCACCACCACCAGCCGGCCGCCGCCGGTGACCGGCGGGAGCAGGGCCCCGGCCTCTCTCAGGGTACGTACTTACACGTGTCCATCGCACGGTCTATCAGTATTTATTTATTAACTACTCTCGAGCTTAATTATGGTATTGTTGATAGTTGATGAAGTTAATTATTGTACGCCGTCTCATCGATCAGTTGGACGACTGGGTGCTGTGCCGCATCTACAAGAAGACCAGCAAGGCCGCGGCCGCGGTCGGAGATGAGCAGAGGAGCATGGAGTGCGAGGACTCCGTGGAGGACGCGGTCACCGCGTACCCGCCCTACGCCACGGCGGGCATGGCCGGCGCAGGTGCGCATGGCAGCAACTACGTTCAACTGCTCCATCATCACGACAGCCACGAGGACAACTTCCAGCTAGACGGCCTGCTCACAGAACACGACGTCGGCCTCTCGGCGGGCGCCGCCTCGCTGGGCCACCTTGCCGCGGCGGCGAGGGCCACCAAACAGTTCCTCGCCCCGTCGTCCTCAACCCCGTTCAACTGGCTCGAGGCGTCAACCGG

>542_*NAM-1*

GCTTTTTATTATACTGTGCACAAGTATTTTTATATTCTTCCAGTAAGTACAGCGCATGTATGTGATCCTGTCGTCGTGCTTGTTCATGCGCTCGGGCGGGATCATCATCCATCAGAGAAGGCGACCTTCGGGGAGCACGAGTGGTACTTCTTCAGCCCGCGCGACCGCAAGTACGCCAACGGCGCGCGGCCGAACCGGGCGGCGACGTCGGGCTACTGGAAGGCCACCGGCACGGACAAGCCTATCCTGGCCTCGGCCACCGGGTGCGGCCGGGAGAAGGTCGGCGTCAAGAAGGCGCTCGTCTTCTACCGCGGGAAGCCGCCCAGGGGCCTCAAGACCAACTGGATCATGCATGAGTACCGCCTCACCGGAGCCTCTGCTGGCTCCACCACCACCAGCCGGCCGCCGCCGGTGACCGGCGGGAGCAGGGCCCCGGCCTCTCTCAGGGTACGTACTTACACGTGTCCATCGCACGGTCTATCAGTATTTATTTATTAACTACTCTCGAGCTTAATTATGGTATTGTTGATAGTTGATGAAGTTAATTATTGTACGCCGTCTCATCGATCAGTTGGACGACTGGGTGCTGTGCCGCATCTACAAGAAGACCAGCAAGGCCGCGGCCGCGGTCGGAGATGAGCAGAGGAGCATGGAGTGCGAGGACTCCGTGGAGGACGCGGTCACCGCGTACCCGCCCTACGCCACGGCGGGCATGGCCGGCGCAGGTGCGCATGGCAGCAACTACGTTCAACTGCTCCATCATCACGACAGCCACGAGGACAACTTCCAGCTAGACGGCCTGCTCACAGAACACGACGTCGGCCTCTCGGCGGGCGCCGCCTCGCTGGGCCACCTTGCCGCGGCGGCGAGGGCCACCAAACAGTTCCTCGCCCCGTCGTCCTCAACCCCGTTCAACTGGCTCGAGGCGTCAACCGG

>543_*NAM-1*

GCTTTTTATTATACTGTGCACAAGTATTTTTATATTCTTCCAGTAAGTACAGCGCATGTATGTGATCCTGTCGTCGTGCTTGTTCATGCGCTCGGGCGGGATCATCATCCATCAGAGAAGGCGACCTTCGGGGAGCACGAGTGGTACTTCTTCAGCCCGCGCGACCGCAAGTACGCCAACGGCGCGCGGCCGAACCGGGCGGCGACGTCGGGCTACTGGAAGGCCACCGGCACGGACAAGCCTATCCTGGCCTCGGCCACCGGGTGCGGCCGGGAGAAGGTCGGCGTCAAGAAGGCGCTCGTCTTCTACCGCGGGAAGCCGCCCAGGGGCCTCAAGACCAACTGGATCATGCATGAGTACCGCCTCACCGGAGCCTCTGCTGGCTCCACCACCACCAGCCGGCCGCCGCCGGTGACCGGCGGGAGCAGGGCCCCGGCCTCTCTCAGGGTACGTACTTACACGTGTCCATCGCACGGTCTATCAGTATTTATTTATTAACTACTCTCGAGCTTAATTATGGTATTGTTGATAGTTGATGAAGTTAATTATTGTACGCCGTCTCATCGATCAGTTGGACGACTGGGTGCTGTGCCGCATCTACAAGAAGACCAGCAAGGCCGCGGCCGCGGTCGGAGATGAGCAGAGGAGCATGGAGTGCGAGGACTCCGTGGAGGACGCGGTCACCGCGTACCCGCCCTACGCCACGGCGGGCATGGCCGGCGCAGGTGCGCATGGCAGCAACTACGTTCAACTGCTCCATCATCACGACAGCCACGAGGACAACTTCCAGCTAGACGGCCTGCTCACAGAACACGACGTCGGCCTCTCGGCGGGCGCCGCCTCGCTGGGCCACCTTGCCGCGGCGGCGAGGGCCACCAAACAGTTCCTCGCCCCGTCGTCCTCAACCCCGTTCAACTGGCTCGAGGCGTCAACCGG

>558_*NAM-1*

GCTTTTTATTATACTGTGCACAAGTATTTTTATATTCTTCCAGTAAGTACAGCGCATGTATGTGATCCTGTCGTCGTGCTTGTTCATGCGCTCGGGCGGGATCATCATCCATCAGAGAAGGCGACCTTCGGGGAGCACGAGTGGTACTTCTTCAGCCCGCGCGACCGCAAGTACCCCAACGGCGCGCGGCCGAACCGGGCGGCGACGTCGGGCTACTGGAAGGCCACCGGCACGGACAAGCCTATCCTGGCCTCGGCCACCGGGTGCGGCCGGGAGAAGGTCGGCGTCAAGAAGGCGCTCGTCTTCTACCGCGGGAAGCCGCCCAGGGGCCTCAAGACCAACTGGATCATGCATGAGTACCGCCTCACCGGAGCCTCTGCTGGCTCCACCACCACCAGCCGGCCGCCGCCGGTGACCGGCGGGAGCAGGGCCCCGGCCTCTCTCAGGGTACGTACTTACACGTGTCCATCGCACGGTCTATCAGTATTTATTTATTAACTACTCTCGAGCTTAATTATGGTATTGTTGATAGTTGATGAAGTTAATTATTGTACGCCGTCTCATCGATCAGTTGGACGACTGGGTGCTGTGCCGCATCTACAAGAAGACCAGCAAGGCCGCGGCCGCGGTCGGAGATGAGCAGAGGAGCATGGAGTGCGAGGACTCCGTGGAGGACGCGGTCACCGCGTACCCGCCCTACGCCACGGCGGGCATGGCCGGCGCAGGTGCGCATGGCAGCAACTACGTTCAACTGCTCCATCATCACGACAGCCACGAGGACAACTTCCAGCTAGACGGCCTGCTCACAGAACACGACGTCGGCCTCTCGGCGGGCGCCGCCTCGCTGGGCCACCTTGCCGCGGCGGCGAGGGCCACCAAACAGTTCCTCGCCCCGTCGTCCTCAACCCCGTTCAACTGGCTCGAGGCGTCAACCGG

>560_*NAM-1*

GCTTTTTATTATACTGTGCACAAGTATTTTTATATTCTTCCAGTAAGTACAGCGCATGTATGTGATCCTGTCGTCGTGCTTGTTCATGCGCTCGGGCGGGATCGTCATCCATCAGAGAAGGCGACCTTCGGGGAGCACGAGTGGTACTTCTTCAGCCCGCGCGACCGCAAGTACCCCAACGGCGCGCGGCCGAACCGGGCGGCGACGTCGGGCTACTGGAAGGCCACCGGCACGGACAAGCCTATCCTGGCCTCGGCCACCGGGTGCGGCCGGGAGAAGGTCGGCGTCAAGAAGGCGCTCGTCTTCTACCGCGGGAAGCCGCCCAGGGGCCTCAAGACCAACTGGATCATGCATGAGTACCGCCTCACCGGAGCCTCTGCTGGCTCCACCACCACCAGCCGGCCGCCGCCGGTGACCGGCGGGAGCAGGGCCCCGGCCTCTCTCAGGGTACGTACTTACACGTGTCCATCGCACGGTCTATCAGTATTTATTTATTAACTACTCTCGAGCTTAATTATGGTATTGTTGATAGTTGATGAAGTTAATTATTGTACGCCGTCTCATCGATCAGTTGGACGACTGGGTGCTGTGCCGCATCTACAAGAAGACCAGCAAGGCCGCGGCCGCGGTCGGAGATGAGCAGAGGAGCATGGAGTGCGAGGACTCCGTGGAGGACGCGGTCACCGCGTACCCGCCCTACGCCACGGCGGGCATGGCCGGCGCAGGTGCGCATGGCAGCAACTACGTTCAACTGCTCCATCATCACGACAGCCACGAGGACAACTTCCAGCTAGACGGCCTGCTCACAGAACACGACGTCGGCCTCTCGGCGGGCGCCGCCTCGCTGGGCCACCTTGCCGCGGCGGCGAGGGCCACCAAACAGTTCCTCGCCCCGTCGTCCTCAACCCCGTTCAACTGGCTCGAGGCGTCAACCGG
