# Supplementary material for: Origin of worldwide cultivated barley revealed by NAM-1 gene and grain protein content
Source: Front Plant Sci. 2015 Sep 30;6:803. doi: 10.3389/fpls.2015.00803 (PMC4588695; doi:10.3389/fpls.2015.00803)
Supplement: Supplementary file 3 [file Data_Sheet_2.DOCX]

***Supplementary Material***

**Origin of worldwide cultivated barley revealed by *NAM-1* gene and grain protein content**

**Yonggang Wang, Xifeng Ren, Dongfa Sun*, Genlou Sun***

*** Correspondence:**

Dongfa Sun: [sundongfa1@mail.hzau.edu.cn](mailto:sundongfa1@mail.hzau.edu.cn)

Genlou Sun: [genlou.sun@smu.ca](mailto:genlou.sun@smu.ca)

**Supplementary Data Sheet 2:** **Multiple sequence alignment of *NAM-1* gene for different haplotypes.** The symbols under the sequence alignment indicate identical residues (*), and weakly conserved (.). Nucleotides belong to exon are shaded in gray. The SNPs are marked in red.

DQ869678_NAM-1 ATCCATCATGCTTTTTATTATACTGTGCACAAGTATTTTTATATTCTTCCAGTAAGTACA

Hap1 ---------GCTTTTTATTATACTGTGCACAAGTATTTTTATATTCTTCCAGTAAGTACA

Hap2 ---------GCTTTTTATTATACTGTGCACAAGTATTTTTATATTCTTCCAGTAAGTACA

Hap3 ---------GCTTTTTATTATACTGTGCACAAGTATTTTTATATTCTTCCAGTAAGTACA

Hap4 ---------GCTTTTTATTATACTGTGCACAAGTATTTTTATATTCTTCCAGTAAGTACA

Hap5 ---------GCTTTTTATTATACTGTGCACAAGTATTTTTATATTCTTCCAGTAAGTACA

Hap6 ---------GCTTTTTATTATACTGTGCACAAGTATTTTTATATTCTTCCAGTAAGTACA

Hap7 ---------GCTTTTTATTATACTGTGCACAAGTATTTTTATATTCTTCCAGTAAGTACA

Hap8 ---------GCTTTATATTATACTGTGCACAAGTATTTTTATATTCTTCCAGTAAGTACA

Hap9 ---------GCTTTTTATTATACTGTGCACAAGTATTTTTATATTCTTCCAGTAAGTACA

Hap10 ---------GCTTTATATTATACTGTGCACAAGTATTTTTATATTCTTCCAGTAAGTACA

***** *********************************************

DQ869678_NAM-1 GCGCATGTATGTGATCCTGTCGTCGTGCTTGTTCATGCGCTCGGGCGGGATCATCATCCA

Hap1 GCGCATGTATGTGATCCTGTCGTCGTGCTTGTTCATGCGCTCGGGCGGGATCGTCATCCA

Hap2 GCGCATGTATGTGATCCTGTCGTCGTGCTTGTTCATGCGCTCGGGCGGGATCATCATCCA

Hap3 GCGCATGTATGTGATCCTGTCGTCGTGCTTGTTCATGCGCTCGGGCGGGATCATCATCCA

Hap4 GCGCATGTATGTGATCCTGTCGTCGTGCTTGTTCATGCGCTCGGGCGGGATCATCATCCA

Hap5 GCGCATGTATGTGATCCTGTCGTCGTGCTTGTTCATGCGCTCGGGCGGGATCATCATCCA

Hap6 GCGCATGTATGTGATCCTGTCGTCGTGCTTGTTCATGCGCTCGGGCGGGATCATCATCCA

Hap7 GCGCATGTATGTGATCCTGTCGTCGTGCTTGTTCATGCGCTCGGGCGGGATCATCATCCA

Hap8 GCGCATGTATGTGATCCTGTCGTCGTGCTTGTTCATGCGCTCGGGCGGGATCATCATCCA

Hap9 GCGCATGTATGTGATCCTGTCGTCGTGCTTGTTCATGCGCTCGGGCGGGATCATCATCCA

Hap10 GCGCATGTATGTGATCCTGTCGTCGTGCTTGTTCATGCGCTCGGGCGGGATCATCATCCA

**************************************************** *******

DQ869678_NAM-1 TCAGAGAAGGCGACCTTCGGGGAGCACGAGTGGTACTTCTTCAGCCCGCGCGACCGCAAG

Hap1 TCAGAGAAGGCGACCTTCGGGGAGCACGAGTGGTACTTCTTCAGCCCGCGCGACCGCAAG

Hap2 TCAGAGAAGGCGACCTTCGGGGAGCACGAGTGGTACTTCTTCAGCCCGCGCGACCGCAAG

Hap3 TCAGAGAAGGCGACCTTCGGGGAGCACGAGTGGTACTTCTTCAGCCCGCGCGACCGCAAG

Hap4 TCAGAGAAGGCGACCTTCGGGGAGCATGAGTGGTACTTCTTCAGCCCGCGCGACCGCAAG

Hap5 TCAGAGAAGGCGACCTTCGGGGAGCACGAGTGGTACTTCTTCAGCCCGCGCGACCGCAAG

Hap6 TCAGAGAAGGCGACCTTCGGGGAGCACGAGTGGTACTTCTTCAGCCCGCGCGACCGCAAG

Hap7 TCAGAGAAGGCGACCTTCGGGGAGCACGAGTGGTACTTCTTCAGCCCGCGCGACCGCAAG

Hap8 TCAGAGAAGGCGACCTTCGGGGAGCACGAGTGGTACTTCTTCAGCCCGCGCGACCGCAAG

Hap9 TCAGAGAAGGCGACCTTCGGGGAGCACGAGTGGTACTTCTTCAGCCCGCGCGACCGCAAG

Hap10 TCAGAGAAGGCGACCTTCGGGGAGCACGAGTGGTACTTCTTCAGCCCGCGCGACCGCAAG

************************** *********************************

DQ869678_NAM-1 TACGCCAACGGCGCGCGGCCGAACCGGGCGGCGACGTCGGGCTACTGGAAGGCCACCGGC

Hap1 TACCCCAACGGCGCGCGGCCGAACCGGGCGGCGACGTCGGGCTACTGGAAGGCCACCGGC

Hap2 TACCCCAACGGCGCGCGGCCGAACCGGGCGGCGACGTCGGGCTACTGGAAGGCCACCGGC

Hap3 TACCCCAACGGCGCGCGGCCGAACCGGGCGGCGACGTCGGGCTACTGGAAGGCCACCGGC

Hap4 TACCCCAACGGCGCGCGGCCGAACCGGGCGGCGACGTCGGGCTACTGGAAGGCCACCGGC

Hap5 TACCCCAACGGCGCGCGGCCGAACCGGGCGGCGACGTCGGGCTACTGGAAGGCCACCGGC

Hap6 TACCCCAACGGCGCGCGGCCGAACCGGGCGGCGACGTCGGGCTACTGGAAGGCCACCGGC

Hap7 TACGCCAACGGCGCGCGGCCGAACCGGGCGGCGACGTCGGGCTACTGGAAGGCCACCGGC

Hap8 TACGCCAACGGCGCGCGGCCGAACCGGGCGGCGACGTCGGGCTACTGGAAGGCCACCGGC

Hap9 TACCCCAACGGCGCGCGGCCGAACCGGGCGGCGACGTCGGGCTACTGGAAGGCCACCGGC

Hap10 TACCCCAACGGCGCGCGGCCGAACCGGGCGGCGACGTCGGGCTACTGGAAGGCCACCGGC

*** ********************************************************

DQ869678_NAM-1 ACGGACAAGCCTATCCTGGCCTCGGCCACCGGGTGCGGCCGGGAGAAGGTCGGCGTCAAG

Hap1 ACGGACAAGCCTATCCTGGCCTCGGCCACCGGGTGCGGCCGGGAGAAGGTCGGCGTCAAG

Hap2 ACGGACAAGCCTATCCTGGCCTCGGCCACCGGGTGCGGCCGGGAGAAGGTCGGCGTCAAG

Hap3 ACGGACAAGCCTATCCTGGCCTCGGCCACCGGGTGCGGCCGGGAGAAGGTCGGCGTCAAG

Hap4 ACGGACAAGCCTATCCTGGCCTCGGCCACCGGGTGCGGCCGGGAGAAGGTCGGCGTCAAG

Hap5 ACGGACAAGCCTATCATGGCCTCGGCCACCGGGTGCGGCCGGGAGAAGGTCGGCGTCAAG

Hap6 ACGGACAAGCCTATCCTGGCCTCGGCCACCGGGTGCGGCCGGGAGAAGGTCGGCGTCAAG

Hap7 ACGGACAAGCCTATCCTGGCCTCGGCCACCGGGTGCGGCCGGGAGAAGGTCGGCGTCAAG

Hap8 ACGGACAAGCCTATCCTGGCCTCGGCCACCGGGTGCGGCCGGGAGAAGGTCGGCGTCAAG

Hap9 ACGGACAAGCCTATCCTGGCCTCGGCCACCGGGTGCGGCCGGGAGAAGGTCGGCGTCAAG

Hap10 ACGGACAAGCCTATCCTGGCCTCGGCCACCGGGTGCGGCCGGGAGAAGGTCGGCGTCAAG

*************** ********************************************

DQ869678_NAM-1 AAGGCGCTCGTCTTCTACCGCGGGAAGCCGCCCAGGGGCCTCAAGACCAACTGGATCATG

Hap1 AAGGCGCTCGTCTTCTACCGCGGGAAGCCGCCCAGGGGCCTCAAGACCAACTGGATCATG

Hap2 AAGGCGCTCGTCTTCTACCGCGGGAAGCCGCCCAGGGGCCTCAAGACCAACTGGATCATG

Hap3 AAGGCGCTCGTCTTCTACCGCGGGAAGCCGCCCAGGGGCCTCAAGACCAACTGGATCATG

Hap4 AAGGCGCTCGTCTTCTACCGCGGGAAGCCGCCCAGGGGCCTCAAGACCAACTGGATCATG

Hap5 AAGGCGCTCGTCTTCTACCGCGGGAAGCCGCCCAGGGGCCTCAAGACCAACTGGATCATG

Hap6 AAGGCGCTCGTCTTCTACCGCGGGAAGCCGCCCAGGGGCCTCAAGACCAACTGGATCATG

Hap7 AAGGCGCTCGTCTTCTACCGCGGGAAGCCGCCCAGGGGCCTCAAGACCAACTGGATCATG

Hap8 AAGGCGCTCGTCTTCTACCGCGGGAAGCCGCCCAGGGGCCTCAAGACCAACTGGATCATG

Hap9 AAGGCGCTCGTCTTCTACCGCGGGAAGCCGCCCAGGGGCCTCAAGACCAACTGGATCATG

Hap10 AAGGCGCTCGTCTTCTACCGCGGGAAGCCGCCCAGGGGCCTCAAGACCAACTGGATCATG

************************************************************

DQ869678_NAM-1 CATGAGTACCGCCTCACCGGAGCCTCTGCTGGCTCCACCACCACCAGCCGGCCGCCGCCG

Hap1 CATGAGTACCGCCTCACCGGAGCCTCTGCTGGCTCCACCACCACCAGCCGGCCGCCGCCG

Hap2 CATGAGTACCGCCTCACCGGAGCCTCTGCTGGCTCCACCACCACCAGCCGGCCGCCGCCG

Hap3 CATGAGTACCGCCTCACCGGAGCCTCTGCTGGCTCCACCACCACCAGCCGGCCGCCGCCG

Hap4 CATGAGTACCGCCTCACCGGAGCCTCTGCTGGCTCCACCACCACCAGCCGGCCGCCGCCG

Hap5 CATGAGTACCGCCTCACCGGAGCCTCTGCTGGCTCCACCACCACCAGCCGGCCGCCGCCG

Hap6 CATGAGTACCGCCTCACCGGAGCCTCTGCTGGCTCCACCACCACCAGCCGGCCGCCGCCG

Hap7 CATGAGTACCGCCTCACCGGAGCCTCTGCTGGCTCCACCACCACCAGCCGGCCGCCGCCG

Hap8 CATGAGTACCGCCTCACCGGAGCCTCTGCTGGCTCCACCACCACCAGCCGGCCGCCGCCG

Hap9 CATGAGTACCGCCTCACCGGAGCCTCTGCTGGCTCCACCACCACCAGCCGGCCGCCGCCG

Hap10 CATGAGTACCGCCTCACCGGAGCCTCTGCTGGCTCCACCACCACCAGCCGGCCGCCGCCG

************************************************************

DQ869678_NAM-1 GTGACCGGCGGGAGCAGGGCCCCGGCCTCTCTCAGGGTACGTACTTACACGTGTCCATCG

Hap1 GTGACCGGCGGGAGCAGGGCCCCGGCCTCTCTCAGGGTACGTACTTACACGTGTCCATCG

Hap2 GTGACCGGCGGGAGCAGGGCCCCGGCCTCTCTCAGGGTACGTACTTACACGTGTCCATCG

Hap3 GTGACCGGCGGGAGCAGGGCCCCGGCCTCTCTCAGGGTACGTACTTACACGTGTCCATCG

Hap4 GTGACCGGCGGGAGCAGGGCCCCGGCCTCTCTCAGGGTACGTACTTACACGTGTCCATCG

Hap5 GTGACCGGCGGGAGCAGGGCCCCGGCCTCTCTCAGGGTACGTACTTACACGTGTCCATCG

Hap6 GTGACCGGCGGGAGCAGGGCCCCGGCCTCTCTCAGGGTACGTCCTTACACGTGTCCATCG

Hap7 GTGACCGGCGGGAGCAGGGCCCCGGCCTCTCTCAGGGTACGTACTTACACGTGTCCATCG

Hap8 GTGACCGGCGGGAGCAGGGCCCCGGCCTCTCTCAGGGTACGTACTTACACGTGTCCATCG

Hap9 GTGACCGGCGGGAGCAGGGCCCCGGCCTCTCTCAGGGTACGTACTTACACGTGTCCATCG

Hap10 GTGACCGGCGGGAGCAGGGCCCCGGCCTCTCTCAGGGTACGTACTTACACGTGTCCATCG

****************************************** *****************

DQ869678_NAM-1 CACGGTCTATCAGTATTTATTTATTAACTACTCTCGAGCTTAATTATGGTATTGTTGATA

Hap1 CACGGTCTATCAGTATTTATTTATTAACTACTCTCGAGCTTAATTATGGTATTGTTGATA

Hap2 CACGGTCTATCAGTATTTATTTATTAACTACTCTCGAGCTTAATTATGGTATTGTTGATA

Hap3 CACGGTCTATCAGTATTTATTTATTAACTACTCTCGAGCTTAATTATGGTATTGTTGATA

Hap4 CACGGTCTATCAGTATTTATTTATTAACTACTCTCGAGCTTAATTATGGTATTGTTGATA

Hap5 CACGGTCTATCAGTATTTATTTATTAACTACTCTCGAGCTTAATTATGGTATTGTTGATA

Hap6 CACGGTCTATCAGTATTTATTTATTAACTACTCTCGAGCTTAATTATGGTATTGTTGATA

Hap7 CACGGTCTATCAGTATTTATTTATTAACTACTCTCGAGCTTAATTATGGTATTGTTGATA

Hap8 CACGGTCTATCAGTATTTATTTATTAACTACTCTCGAGCTTAATTATGGTATTGTTGATA

Hap9 CACGGTCTATCAGTATTTATTTATTAACTACTCTCGAGCTTAATTATGGTATTGTTGATA

Hap10 CACGGTCTATCAGTATTTATTTATTAACTACTCTCGAGCTTAATTATGGTATTGTTGATA

************************************************************

DQ869678_NAM-1 GTTGATGAAGTTAATTATTGTACGCCGTCTCATCGATCAGTTGGACGACTGGGTGCTGTG

Hap1 GTTGATGAAGTTAATTATTGTACGCCGTCTCATCGATCAGTTGGACGACTGGGTGCTGTG

Hap2 GTTGATGAAGTTAATTATTGTACGCCGTCTCATCGATCAGTTGGACGACTGGGTGCTGTG

Hap3 GTTGATGAAGTTAATTATTGTACGCCGTCTCATCGATCAGTTGGACGACTGGGTGCTGTG

Hap4 GTTGATGAAGTTAATTATTGTACGCCGTCTCATCGATCAGTTGGACGACTGGGTGCTGTG

Hap5 GTTGATGAAGTTAATTATTGTACGCCGTCTCATCGATCAGTTGGACGACTGGGTGCTGTG

Hap6 GTTGATGAAGTTAATTATTGTACGCCGTCTCATCGATCAGTTGGACGACTGGGTGCTGTG

Hap7 GTTGATGAAGTTAATTATTGTACGCCGTCTCATCGATCAGTTGGACGACTGGGTGCTGTG

Hap8 GTTGATGAAGTTAATTATTGTACGCCGTCTCATCGATCAGTTGGACGACTGGGTGCTGTG

Hap9 GTTGATGAAGTTAATTATTGTACGCCGTCTCATCGATCAGTTGGACGACTGGGTGCTGTG

Hap10 GTTGATGAAGTTAATTATTGTACGCCGTCTCATCGATCAGTTGGACGACTGGGTGCTGTG

************************************************************

DQ869678_NAM-1 CCGCATCTACAAGAAGACCAGCAAGGCCGCGGCCGCGGTCGGAGATGAGCAGAGGAGCAT

Hap1 CCGCATCTACAAGAAGACCAGCAAGGCCGCGGCCGCGGTCGGAGATGAGCAGAGGAGCAT

Hap2 CCGCATCTACAAGAAGACCAGCAAGGCCGCGGCCGCGGTCGGAGATGAGCAGAGGAGCAT

Hap3 CCGCATCTACAAGAAGACCAGCAAGGCCGCGGCCGCGGTCGGAGATGAGCAGAGGAGCAT

Hap4 CCGCATCTACAAGAAGACCAGCAAGGCCGCGGCCGCGGTCGGAGATGAGCAGAGGAGCAT

Hap5 CCGCATCTACAAGAAGACCAGCAAGGCCGCGGCCGCGGTCGGAGATGAGCAGAGGAGCAT

Hap6 CCGCATCTACAAGAAGACCAGCAAGGCCGCGGCCGCGGTCGGAGATGAGCAGAGGAGCAT

Hap7 CCGCATCTACAAGAAGACCAGCAAGGCCGCGGCCGCGGTCGGAGATGAGCAGAGGAGCAT

Hap8 CCGCATCTACAAGAAGACCAGCAAGGCCGCGGCCGCGGTCGGAGATGAGCAGAGGAGCAT

Hap9 CCGCATCTACAAGAAGACCAGCAAGGCCGCGGCCGCGGTCGGAGATGAGCAGAGGAGCAT

Hap10 CCGCATCTACAAGAAGACCAGCAAGGCCGCGGCCGCGGTCGGAGATGAGCAGAGGAGCAT

************************************************************

DQ869678_NAM-1 GGAGTGCGAGGACTCCGTGGAGGACGCGGTCACCGCGTACCCGCCCTACGCCACGGCGGG

Hap1 GGAGTGCGAGGACTCCGTGGAGGACGCGGTCACCGCGTACCCGCCCTACGCCACGGCGGG

Hap2 GGAGTGCGAGGACTCCGTGGAGGACGCGGTCACCGCGTACCCGCCCTACGCCACGGCGGG

Hap3 GGAGTGCGAGGACTCCGTGGAGGACGCGGTCACCGCGTACCCGCCCTACGCCACGGCGGG

Hap4 GGAGTGCGAGGACTCCGTGGAGGACGCGGTCACCGCGTACCCGCCCTACGCCACGGCGGG

Hap5 GGAGTGCGAGGACTCCGTGGAGGACGCGGTCACCGCGTACCCGCCCTACGCCACGGCGGG

Hap6 GGAGTGCGAGGACTCCGTGGAGGACGCGGTCACCGCGTACCCGCCCTACGCCACGGCGGG

Hap7 GGAGTGCGAGGACTCCGTGGAGGACGCGGTCACCGCGTACCCGCCCTACGCCACGGCGGG

Hap8 GGAGTGCGAGGACTCCGTGGAGGACGCGGTCACCGCGTACCCGCCCTACGCCACGGCGGG

Hap9 GGAGTGCGAGGACTCCGTGGAGGACGCGGTCACCGCGTACCCGCCCTACGCCACGGCGGG

Hap10 GGAGTGCGAGGACTCCGTGGAGGACGCGGTCACCGCGTACCCGCCCTACGCCACGGCGGG

************************************************************

DQ869678_NAM-1 CATGGCCGGCGCAGGTGCGCATGGCAGCAACTACGTTCAACTGCTCCATCATCACGACAG

Hap1 CATGGCCGGCGCAGGTGCGCATGGCAGCAACTACGTTCAACTGCTCCATCATCACGACAG

Hap2 CATGGCCGGCGCAGGTGCGCATGGCAGCAACTACGTTCAACTGCTCCATCATCACGACAG

Hap3 CATGGCCGGCGCAGGTGCGCATGGCAGCAACTACGTTCAACTGCTCCATCATCACGACAG

Hap4 CATGGCCGGCGCAGGTGCGCATGGCAGCAACTACGTTCAACTGCTCCATCATCACGACAG

Hap5 CATGGCCGGCGCAGGTGCGCATGGCAGCAACTACGTTCAACTGCTCCATCATCACGACAG

Hap6 CATGGCCGGCGCAGGTGCGCATGGCAGCAACTACGTTCAACTGCTCCATCATCACGACAG

Hap7 CATGGCCGGCGCAGGTGCGCATGGCAGCAACTACGTTCAACTGCTCCATCATCACGACAG

Hap8 CATGGCCGGCGCAGGTGCGCATGGCAGCAACTACGTTCAACTGCTCCATCATCACGACAG

Hap9 CATGGCCGGCGCAGGTGCGCATGGCAGCAACTACGTTCAACTGCTCCATCATCACGACAG

Hap10 CATGGCCGGCGCAGGTGCGCATGGCAGCAACTACGTTCAACTGCTCCATCATCACGACAG

************************************************************

DQ869678_NAM-1 CCACGAGGACAACTTCCAGCTAGACGGCCTGCTCACAGAACACGACGTCGGCCTCTCGGC

Hap1 CCACGAGGACAACTTCCAGCTAGACGGCCTGCTCACAGAACACGACGTCGGCCTCTCGGC

Hap2 CCACGAGGACAACTTCCAGCTAGACGGCCTGCTCACAGAACACGACGTCGGCCTCTCGGC

Hap3 CCACGAGGACAACTTCCAGCTAGACGGCCTGCTCACAGAACACGACGTCGGCCTCTCGGC

Hap4 CCACGAGGACAACTTCCAGCTAGACGGCCTGCTCACAGAACACGACGTCGGCCTCTCGGC

Hap5 CCACGAGGACAACTTCCAGCTAGACGGCCTGCTCACAGAACACGACGTCGGCCTCTCGGC

Hap6 CCACGAGGACAACTTCCAGCTAGACGGCCTGCTCACAGAACACGACGTCGGCCTCTCGGC

Hap7 CCACGAGGACAACTTCCAGCTAGACGGCCTGCTCACAGAACACGACGTCGGCCTCTCGGC

Hap8 CCACGAGGACAACTTCCAGCTAGACGGCCTGCTCACAGAACACGACGTCGGCCTCTCGGC

Hap9 CCACGAGGACAACTTCCAGCTAGACGGCCTGCTCACAGAACACGACGTCAGCCTCTCGGC

Hap10 CCACGAGGACAACTTCCAGCTAGACGGCCTGCTCACAGAACACGACGTCAGCCTCTCGGC

************************************************* **********

DQ869678_NAM-1 GGGCGCCGCCTCGCTGGGCCACCTTGCCGCGGCGGCGAGGGCCACCAAACAGTTCCTCGC

Hap1 GGGCGCCGCCTCGCTGGGCCACCTTGCCGCGGCGGCGAGGGCCACCAAACAGTTCCTCGC

Hap2 GGGCGCCGCCTCGCTGGGCCACCTTGCCGCGGCGGCGAGGGCCACCAAACAGTTCCTCGC

Hap3 GGGCGCCGCCTCGCTGGGCCACCTTGCCGCGGCGGCGAGGGCCACCAAACAGCTCCTCGC

Hap4 GGGCGCCGCCTCGCTGGGCCACCTTGCCGCGGCGGCGAGGGCCACCAAACAGTTCCTCGC

Hap5 GGGCGCCGCCTCGCTGGGCCACCTTGCCGCGGCGGCGAGGGCCACCAAACAGTTCCTCGC

Hap6 GGGCGCCGCCTCGCTGGGCCACCTTGCCGCGGCGGCGAGGGCCACCAAACAGTTCCTCGC

Hap7 GGGCGCCGCCTCGCTGGGCCACCTTGCCGCGGCGGCGAGGGCCACCAAACAGTTCCTCGC

Hap8 GGGCGCCGCCTCGCTGGGCCACCTTGCCGCGGCGGCGAGGGCCACCAAACAGTTCCTCGC

Hap9 GGGCGCCGCCTCGCTGGGCCACCTTGCCGCGGCGGCGAGGGCCACCAAACAGTTCCTCGC

Hap10 GGGCGCCGCCTCGCTGGGCCACCTTGCCGCGGCGGCGAGGGCCACCAAACAGTTCCTCGC

**************************************************** *******

DQ869678_NAM-1 CCCGTCGTCCTCAACCCCGTTCAACTGGCTCGAGGCGTCAACCGGTGGCAGCATCCTCCC

Hap1 CCCGTCGTCCTCAACCCCGTTCAACTGGCTCGAGGCGTCAACCGG---------------

Hap2 CCCGTCGTCCTCAACCCCGTTCAACTGGCTCGAGGCGTCAACCGG---------------

Hap3 CCCGTCGTCCTCAACCCCGTTCAACTGGCTCGAGGCGTCAACCGG---------------

Hap4 CCCGTCGTCCTCAACCCCGTTCAACTGGCTCGAGGCGTCAACCGG---------------

Hap5 CCCGTCGTCCTCAACCCCGTTCAACTGGCTCGAGGCGTCAACCGG---------------

Hap6 CCCGTCGTCCTCAACCCCGTTCAACTGGCTCGAGGCGTCAACCGG---------------

Hap7 CCCGTCGTCCTCAACCCCGTTCAACTGGCTCGAGGCGTCAACCGG---------------

Hap8 CCCGTCGTCCTCAACCCCGTTCAACTGGCTCGAGGCGTCAACCGG---------------

Hap9 CCCGTCGTCCTCAACCCCGTTCAACTGGCTCGAGGCGTCAACCGG---------------

Hap10 CCCGTCGTCCTCAACCCCGTTCAACTGGCTCGAGGCGTCAACCGG---------------

*********************************************
